# Genome-wide identification and expression analysis of aquaporin family in *Canavalia rosea* and their roles in the adaptation to saline-alkaline soils and drought stress

**DOI:** 10.1186/s12870-021-03034-1

**Published:** 2021-07-13

**Authors:** Ruoyi Lin, Jiexuan Zheng, Lin Pu, Zhengfeng Wang, Qiming Mei, Mei Zhang, Shuguang Jian

**Affiliations:** 1 Guangdong, Provincial Key Laboratory of Applied Botany & Key Laboratory of South China Agricultural Plant Molecular Analysis and Genetic Improvement, South China Botanical Garden, Chinese Academy of Sciences, Guangzhou, 510650 China; 2 University of the Chinese Academy of Sciences, Beijing, 100039 China; 3 Key Laboratory of Vegetation Restoration and Management of Degraded Ecosystems, Center for Plant Ecology, Core Botanical Gardens, Chinese Academy of Sciences, Guangzhou, 510650 China; 4 Southern Marine Science and Engineering Guangdong Laboratory (Guangzhou), Guangzhou, 511458 China; 5 Center of Economic Botany, Core Botanical Gardens, Chinese Academy of Sciences, Guangzhou, 510650 China; 6 CAS Engineering Laboratory for Vegetation Ecosystem Restoration On Islands and Coastal Zones, South China Botanical Garden, Chinese Academy of Sciences, Guangzhou, 510650 China

**Keywords:** Aquaporin, Drought, Saline-alkaline soil, Water deficit, *Canavalia rosea* (Sw.) DC.

## Abstract

**Background:**

*Canavalia rosea* (Sw.) DC. (bay bean) is an extremophile halophyte that is widely distributed in coastal areas of the tropics and subtropics. Seawater and drought tolerance in this species may be facilitated by aquaporins (AQPs), channel proteins that transport water and small molecules across cell membranes and thereby maintain cellular water homeostasis in the face of abiotic stress. In *C. rosea*, AQP diversity, protein features, and their biological functions are still largely unknown.

**Results:**

We describe the action of AQPs in *C. rosea* using evolutionary analyses coupled with promoter and expression analyses. A total of 37 AQPs were identified in the *C. rosea* genome and classified into five subgroups: 11 plasma membrane intrinsic proteins, 10 tonoplast intrinsic proteins, 11 Nod26-like intrinsic proteins, 4 small and basic intrinsic proteins, and 1 X-intrinsic protein. Analysis of RNA-Seq data and targeted qPCR revealed organ-specific expression of aquaporin genes and the involvement of some AQP members in adaptation of *C. rosea* to extreme coral reef environments. We also analyzed *C. rosea* sequences for phylogeny reconstruction, protein modeling, cellular localizations, and promoter analysis. Furthermore, one of PIP1 gene, *CrPIP1;5*, was identified as functional using a yeast expression system and transgenic overexpression in *Arabidopsis*.

**Conclusions:**

Our results indicate that AQPs play an important role in *C. rosea* responses to saline-alkaline soils and drought stress. These findings not only increase our understanding of the role AQPs play in mediating *C. rosea* adaptation to extreme environments, but also improve our knowledge of plant aquaporin evolution more generally.

**Supplementary Information:**

The online version contains supplementary material available at 10.1186/s12870-021-03034-1.

## Background

*Canavalia rosea* (Sw.) DC. (also called bay bean) is an extremophile halophyte and nitrogen-fixing legume species that is widely distributed in coastal areas of tropical and subtropical regions and is highly adapted to seawater and drought conditions [1]. The seeds of *C. rosea* have nutritional and medicinal value and this species constitutes an important wild plant resource. Notably, *C. rosea* presents better growth potential than most of native species, thereby plays basic and pioneering roles in island greening, sand fixation, and ecological restoration of tropical and subtropical coral islands and coastal zones [2]. Sandy soils, salinization, and seasonal drought are factors that limit growth for many plants in coastal areas or coral reefs. *Canavalia rosea* belongs to the “mangrove associates” group, in which some elaborate mechanisms for adapting to highly saline and alkaline soils and drought stress have evolved at both the morphological and physiological-molecular levels. Understanding the molecular and evolutionary mechanisms of *C. rosea*’s adaptation to the special habitats would help to illuminate extremophile adaptations to adverse conditions. Saline-alkaline soils and drought stress both cause plant cellular water deficits [3] and result in water imbalances, from root water uptake to leaf transpiration [4]. Identification of genes involved in responding to water-deficit stress in *C. rosea* may be valuable for molecular breeding improvement of saline-alkaline and drought-related traits through genetic engineering.

Water is an essential component of any biological system and plants exhibit elaborate adaptations to maintain survival in the presence of water stress. Aquaporins (AQPs) are transmembrane proteins that play critical roles in controlling transmembrane water transport in and out of plant cells by forming water channels [5]. In addition to water transport, AQPs facilitate the transport of small molecules such as urea, H_2_O_2_, and NO, and elements such as boron and silicon across cell membranes [6]. Aquaporins are found in a wide variety of taxa, including microbes, animals, and plants, and are the oldest family of major intrinsic proteins (MIPs). Aquaporins have been traditionally classified into four major subfamilies: plasma membrane intrinsic proteins (PIPs), tonoplast intrinsic proteins (TIPs), Nod26-like intrinsic proteins (NIPs), and small and basic intrinsic proteins (SIPs) [7]. Additionally, in some plant genomes, a small number of AQPs have been identified as a fifth subfamily called X-intrinsic proteins (XIPs), which are absent from monocots and Brassicaceae [8]. Furthermore, GlpF-like intrinsic proteins (GIPs) isolated from a moss (*Physcomitrella patens*) and hybrid intrinsic proteins (HIPs) found in a fern (*Selaginella moellendorffii*) and a moss (*P. patens*), which are rare in most plants, are both classified into the AQP family [9, 10].

Structurally, almost all AQPs consist of six transmembrane domains (α-helices, H1 to H6) with N and C termini facing the cytosol [11]. The six transmembrane domains are joined by five interhelical loops (A–E). The conserved loops (B and E) show extremely hydrophobic characteristic, often containing internal repeats of asparagine-proline-alanine residues (NPA motifs). These conserved, hydrophobic loops seem to be the most important features maintaining AQP function by forming short helices [12, 13]. Aromatic/Arginine regions (ar/R) and Froger’s positions are also conserved in most of AQPs [14]. Generally, AQPs are inserted into membranes in a tetrameric structure comprising four independent pores created by AQP monomers [15]. Besides being water channel proteins, some AQPs are also involved in facilitating the transport of CO_2_ [16], NO [17], glycerol [18], H_2_O_2_ [19], some trivalent elements [20], and a wide range of small uncharged solutes [21]. It is clear that AQPs show versatile functions in water uptake, nutrient balancing, long-distance signal transfer, nutrient/heavy metal acquisition in plant development, and stress responses [22].

Unlike the *AQP* members in yeast (only two genes, *AQY1* and *AQY2*) [23] or animals (only 13 *AQP*s in mammals) [24], plants *AQP*s comprised large, highly diverse gene families that may be linked to plants’ greater adaptability to local conditions given their sessile nature [11, 25]. Many *AQP* gene families have been identified using cDNA and whole-genome analyses in a wide variety of plant species, including *Arabidopsis* (35 members) [26], maize (31 members) [27], and rice (34 members) [28]. Given advances in whole genome sequencing, *AQP*-related research has recently gained traction in studies of plant adaptation, especially for halophyte and drought-tolerant plants. As a close relative of *Arabidopsis*, *Eutrema salsugineum* has been considered a model extremophile used to identify mechanisms of salt tolerance. The AQP family in *E. salsugineum* has been characterized by assessing differential gene expression patterns, with research mostly focused on assessing responses to salt, cold, and drought stress [29, 30]. Chickpea (*Cicer arietinum*) has better drought tolerance than most of leguminous species and its *AQP* gene family has been characterized to further investigate its adaptability to water deficit [31, 32]. Furthermore the *AQP* gene family of cassava (*Manihot esculenta*), a drought-tolerant tuber that is an important food resource in many African countries, has been characterized in terms of its evolution, structure, and expression patterns [33]. *Canavalia rosea* is more tolerant to drought, high salinity, heat, and low nitrogen and phosphorous than most of leguminous plants. It is therefore of particular interest to identify the complete set of *AQP*s within *C. rosea *(*CrAQPs*) and to perform comparative analyses to understand their evolutionary relationships, particularly regarding the adaptability of this species to coastal and coral reef habitats.

In our study, the availability of whole genome sequence data for *C. rosea* facilitated genome-wide analysis to identify the evolutionary relationships between *C. rosea* AQPs and those of related leguminous species. We characterized the structure of *CrAQP*s and their chromosomal locations*.* We also investigated the expression profiles of *CrAQP* genes in various tissues, in response to different abiotic stressors, and in different habitats, along with promoter analyses. Additionally, a single plasma membrane intrinsic protein gene, *CrPIP1;5*, was functionally identified using heterogeneous transgenic assays.

## Results

### Identification of the *C. rosea* AQP family

**Fig. 1:**
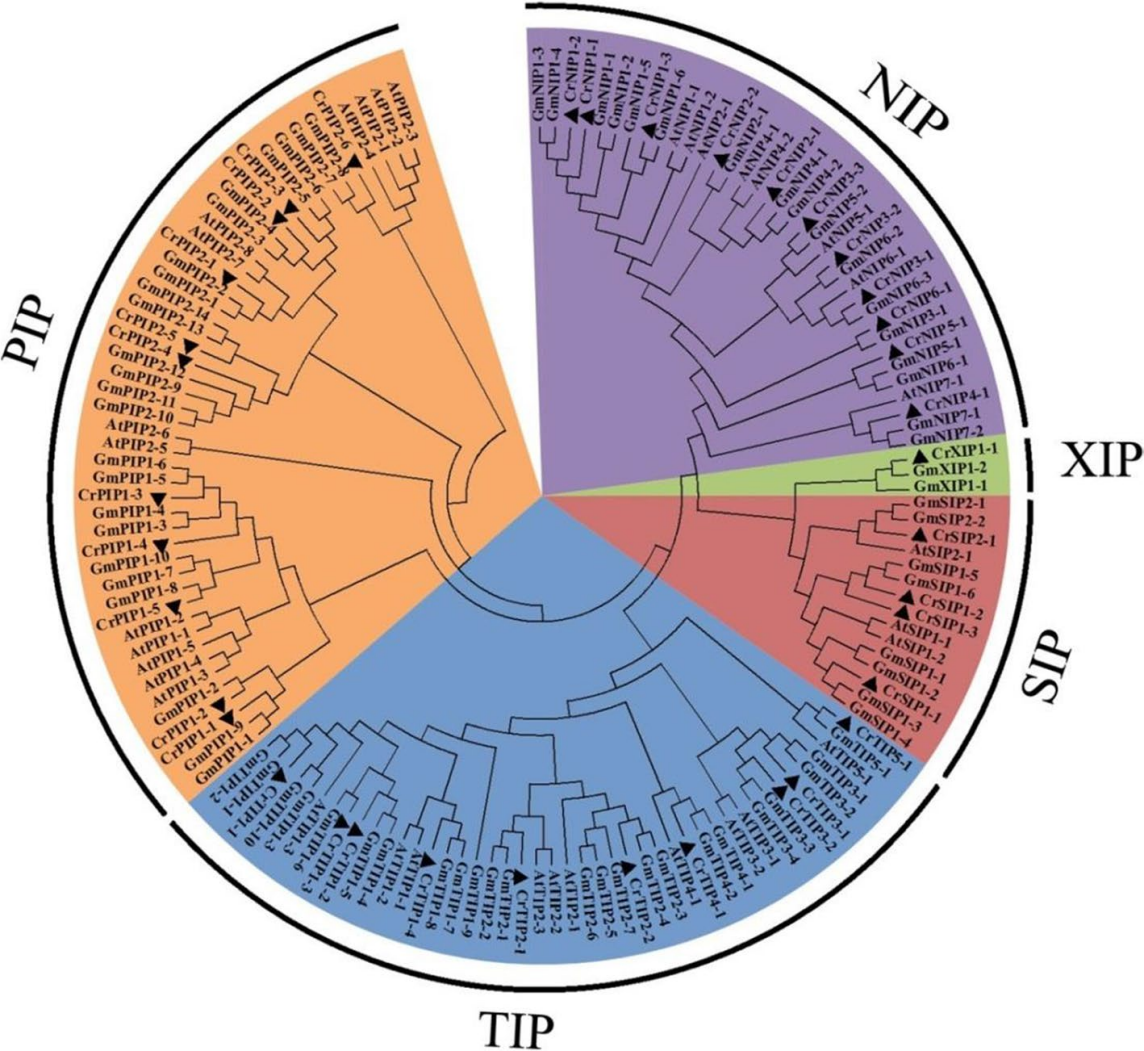
Phylogenetic relationships of the 37 CrAQPs from *C. rosea*, the 75 GmAQPs from soybean, and the 35 AtAQPs from Arabidopsis. The phylogenetic tree is constructed using MEGA 6.0 software, with ClustalW alignment, neighbor-joining (NJ) method, the bootstrap method, and 1000 repetitions. All five subfamilies of *AQP* gene family are well separated in different clades and represented by different color background

Base on the protein BLAST research and Hidden Markov model profile (Pfam ID: PF00230) search, a total of 37 CrAQP members were identified and annotated in the *C. rosea* genome database. The set of CrAQPs includes 11 NIPs, 11 PIPs, 10 TIPs, 4 SIPs, and 1 XIP (Table 1), which were named according to their phylogenetic and sequence identity relationships with AtAQP and GmAQP proteins (Table 1). Based on multiple alignments, a neighbor-joining phylogenetic tree was constructed with the amino acid sequences of AQPs from *C. rosea*, *Arabidopsis*, and soybean (Fig. 1). The clustering results clearly showed that there was only one sequence encoding for the XIP protein in *C. rosea*. In addition, the SIP subfamily in *C. rosea* (CrSIP) had a smaller but more conserved cluster than other three subfamilies in *C. rosea* (CrNIP, CrPIP, and CrTIP). We also compared the number of AQP genes in *C. rosea* with other plant genomes (Table 2), including four Leguminosae species (bean [*Phaseolus vulgaris*, 41 genes], chickpea [*Cicer arietinum*, 40], wild peanut A [*Arachis duranensis*, 32], and wild peanut B [*Arachis ipaensis*, 36]), two Brassicaceae species (Arabidopsis [*Arabidopsis thaliana*, 35] and salt cress [*Eutrema salsugineum*, 35]), and three Gramineae species (rice [*Oryza sativa*, 33], maize [*Zea mays*, 31], and foxtail millet [*Setaria italica*, 39]). The numbers of AQP genes in all of these typical diploid species were similar. The soybean (*G. max*) genome contains 72 AQP genes, which might be due to a whole-genome duplication event in the distant past [34].

**Table 1:** Nomenclature and subcellular localization of AQPs identified from *C. rosea* genome

Name	Locus	Mw (kD)	PI	GRAVY	No. of Phosphor Sites	Prediction for cellular-localization	
						WoLF_PSORT^a^	Plant-mPLoc
CrPIP1;1	02T006326	31.00	9.24	0.304	Ser: 9 Thr: 8 Tyr: 4	plas	plas
CrPIP1;2	03T010068	30.96	9.24	0.265	Ser: 9 Thr: 8 Tyr: 5	plas	plas
CrPIP1;3	01T000438	30.71	8.83	0.405	Ser: 10 Thr: 6 Tyr: 3	plas	plas
CrPIP1;4	10T026210	34.33	7.56	0.286	Ser: 14 Thr: 8 Tyr: 3	plas	plas
CrPIP1;5	06T017675	30.89	8.90	0.361	Ser: 12 Thr: 6 Tyr: 3	plas	plas
CrPIP2;1	02T003848	26.63	8.84	0.429	Ser: 12 Thr: 5 Tyr: 3	plas/vacu	plas
CrPIP2;2	08T022691	30.62	8.24	0.348	Ser: 11 Thr: 9 Tyr: 4	plas	plas
CrPIP2;3	09T024941	31.07	7.66	0.383	Ser: 9 Thr: 10 Tyr: 3	plas	plas
CrPIP2;4	07T020364	30.69	8.28	0.497	Ser: 11 Thr: 7 Tyr: 4	plas	plas
CrPIP2;5	04T013368	30.77	8.28	0.462	Ser: 12 Thr: 7 Tyr: 5	plas	plas
CrPIP2;6	03T008319	30.53	8.82	0.401	Ser: 11 Thr: 6 Tyr: 5	plas	plas
CrTIP1;1	03T010879	26.00	5.32	0.744	Ser: 15 Thr: 2 Tyr: 1	plas/vacu	vacu
CrTIP1;2	08T022781	25.84	5.41	0.864	Ser: 11 Thr: 3 Tyr: 1	vacu/plas	vacu
CrTIP1;3	09T025003	26.06	5.70	0.769	Ser: 7 Thr: 5 Tyr: 1	plas/vacu	vacu
CrTIP1;4	03T008366	25.65	5.78	0.863	Ser: 8 Thr: 5 Tyr: 4	plas/vacu	vacu
CrTIP2;1	10T026350	25.25	4.80	1.005	Ser: 8 Thr: 4 Tyr: 3	vacu	vacu
CrTIP2;2	02T007260	25.16	5.76	0.933	Ser: 11 Thr: 4 Tyr: 3	plas/vacu	vacu
CrTIP3;1	07T020862	27.08	6.34	0.661	Ser: 8 Thr: 4 Tyr: 2	plas/vacu/	vacu
CrTIP3;2	04T012996	27.28	6.74	0.586	Ser: 9 Thr: 6 Tyr: 2	plas/cyto_plas	vacu
CrTIP4;1	02T004418	28.53	5.77	0.798	Ser: 9 Thr: 4 Tyr: 1	plas/vacu	vacu
CrTIP5;1	08T023301	26.44	7.74	0.760	Ser: 13 Thr: 6 Tyr: 2	plas	plas
CrNIP1;1	11T029123	28.33	6.96	0.528	Ser: 11 Thr: 10 Tyr: 1	plas/vacu	plas/vacu
CrNIP1;2	11T029124	28.32	7.01	0.414	Ser: 12 Thr: 7 Tyr: 2	plas/vacu	plas/vacu
CrNIP1;3	01T000942	32.85	9.18	0.460	Ser: 14 Thr: 10 Tyr: 3	plas/vacu/E.R	plas
CrNIP2;1	06T018871	27.82	6.41	0.709	Ser: 15 Thr: 6 Tyr: 5	plas/vacu	plas
CrNIP2;2	01T002720	27.48	5.11	0.650	Ser: 14 Thr: 4 Tyr: 4	plas	plas
CrNIP3;1	09T025384	30.90	7.67	0.465	Ser: 8 Thr: 10 Tyr: 1	plas	plas
CrNIP3;2	02T007404	31.72	8.83	0.460	Ser: 13 Thr: 14 Tyr: 1	plas	plas
CrNIP3;3	04T013473	31.04	8.95	0.459	Ser: 11 Thr: 8 Tyr: 2	plas	plas
CrNIP4;1	04T011155	78.97	9.08	0.092	Ser: 32 Thr: 17 Tyr: 5	plas	plas/chlo
CrNIP5;1	03T010636	30.41	8.57	0.387	Ser: 16 Thr: 11 Tyr: 1	plas	plas
CrNIP6;1	06T018390	30.47	6.83	0.569	Ser: 16 Thr: 11 Tyr: 1	plas/vacu	plas
CrSIP1;1	03T007625	26.53	8.53	0.721	Ser: 4 Thr: 7 Tyr: 2	plas	plas
CrSIP1;2	08T022477	25.88	9.92	0.621	Ser: 4 Thr: 10 Tyr: 2	plas	plas
CrSIP1;3	08T022478	17.13	10.22	0.434	Ser: 6 Thr: 3 Tyr: 3	plas/vacu	plas
CrSIP2;1	03T007754	26.21	9.80	0.547	Ser: 14 Thr: 4 Tyr: 1	vacu/plas/E.R	plas
CrXIP1;1	08T023183	31.59	5.55	0.621	Ser: 11 Thr: 8 Tyr: 0	plas	plas

^a^ The scores predicted by WoLF PSORT equal to or below 3 are ignored

**Table 2:** The numbers of AQP genes in different plant species

Family	Species	Total No	The No. of AQP members in sub-families				
			PIPs	TIPs	NIPs	SIPs	XIPs
Leguminosae	*Canavalia rosea*	37	11	10	11	4	1
	*Phaseolus vulgaris*	41	12	13	10	4	2
	*Cicer arietinum*	40	9	12	16	3	0
	*Arachis duranensis*	32	9	11	8	3	1
	*Arachis ipaensis*	36	9	10	10	3	4
	*Glycine max*	72	24	24	17	8	2
Brassicaceae	*Arabidopsis thaliana*	35	13	10	9	3	0
	*Eutrema salsugineum*	35	12	11	9	3	0
Gramineae	*Oryza sativa*	33	11	10	10	2	0
	*Zea mays*	31	13	11	4	3	0
	*Setaria italica*	39	12	11	13	3	0

The length of CrAQP proteins ranged from 155 aa (CrSIP1;3) to 709 aa (CrNIP4;1), while most were between 230 and 320 aa. The predicted molecular weight and isoelectric points of the CrAQPs ranged from 17.13 kDa to 78.97 kDa and 4.8 to 9.92, respectively (Tables 1 and 3). Thirty four of the 37 CrAQPs included six transmembrane domains and the remaining three members (CrNIP4;1, CrSIP1;3, and CrXIP1;1) possessed seven, three, and five transmembrane domains, respectively (Table 3). The identification of transmembrane regions of CrAQPs is shown in Figure S1.

**Table 3:** Conserved amino acid residues (Asn-Pro-Ala, NPA) motifs, aromatic/arginine (ar/R) filters and Froger’s positions (FPs) and trans-membrane (TM) domains of AQPs in *C. rosea*

Name	AA	TMD	NPA (LB)	NPA (LE)	NPA space	Ar/Rfilters				Froger’s residues				
						H2	H5	LE1	LE2	P1	P2	P3	P4	P5
CrPIP1;1	288	6	NPA	NPA	118	F	H	T	R	Q	S	A	F	W
CrPIP1;2	287	6	NPA	NPA	118	F	H	T	R	Q	S	A	F	W
CrPIP1;3	286	6	NPA	NPA	119	F	H	T	R	E	S	A	F	W
CrPIP1;4	317	6	NPA	NPA	119	F	H	T	R	E	S	A	D	F
CrPIP1;5	288	6	NPA	NPA	119	F	H	T	R	E	S	S	F	W
CrPIP2;1	250	6	\	NPA	\	F	H	T	R	M	S	A	F	W
CrPIP2;2	285	6	NPA	NPA	118	F	H	T	R	Q	S	A	F	W
CrPIP2;3	289	6	NPA	NPA	118	F	H	T	R	Q	S	A	Y	W
CrPIP2;4	288	6	NPA	NPA	118	F	H	T	R	Q	S	A	F	W
CrPIP2;5	287	6	NPA	NPA	118	F	H	T	R	Q	S	A	F	W
CrPIP2;6	284	6	NPA	NPA	118	F	H	T	R	Q	S	A	F	W
CrTIP1;1	252	6	NPA	NPA	111	H	I	A	V	T	S	A	C	W
CrTIP1;2	252	6	NPA	NPA	111	H	I	A	V	T	S	A	Y	W
CrTIP1;3	252	6	NPA	NPA	111	H	I	A	V	T	S	A	Y	W
CrTIP1;4	250	6	NPA	NPA	111	H	I	A	V	T	S	S	Y	W
CrTIP2;1	249	6	NPA	NPA	111	H	I	G	R	T	S	A	Y	W
CrTIP2;2	248	6	NPA	NPA	111	H	I	G	R	T	S	A	Y	W
CrTIP3;1	255	6	NPA	NPA	111	H	I	A	L	T	A	S	F	W
CrTIP3;2	257	6	NPA	NPA	111	H	I	A	R	T	A	A	F	W
CrTIP4;1	272	6	NPS	NPA	111	H	I	A	R	S	S	A	Y	W
CrTIP5;1	253	6	NPA	NPA	111	N	V	G	C	V	A	A	Y	W
CrNIP1;1	268	6	NPA	NPA	109	W	V	A	R	F	S	T	Y	L
CrNIP1;2	269	6	NPA	NPA	109	W	V	A	R	F	S	A	Y	L
CrNIP1;3	306	6	NPA	NPV	109	W	V	A	R	F	S	A	Y	L
CrNIP2;1	261	6	NPA	NPA	109	W	V	A	R	L	S	A	Y	I
CrNIP2;2	256	6	NPA	NPA	109	W	L	A	R	F	S	A	Y	I
CrNIP3;1	299	6	NPA	NPV	108	T	I	G	R	F	T	A	Y	L
CrNIP3;2	307	6	NPA	NPV	108	N	I	S	R	F	T	A	Y	L
CrNIP3;3	299	6	NPA	NPV	108	A	I	G	R	Y	T	A	Y	L
CrNIP4;1	709	7	NPA	NPA	108	A	V	G	R	Y	S	A	Y	I
CrNIP5;1	290	6	NPA	NPA	108	A	S	G	R	F	T	A	Y	F
CrNIP6;1	287	6	NPA	NPA	104	S	I	A	R	Y	S	A	Y	I
CrSIP1;1	247	6	NPT	NPA	112	V	V	P	N	M	A	A	Y	W
CrSIP1;2	239	6	NPS	NPA	113	A	V	P	N	L	A	A	Y	W
CrSIP1;3	155	3	NPS	NLG	79	\	V	P	F	L	A	A	Y	W
CrSIP2;1	236	6	NPL	NPA	108	S	H	G	S	I	V	A	Y	W
CrXIP1;1	296	5	SPV	NPA	127	V	V	V	R	D	C	A	\	\

### Features of AQP proteins

The phosphorylation state of AQP proteins is a key factor regulating the transport of water and other small molecules, or affecting of the protein subcellular localization [14, 22]. In this study, we predicted the possible phosphorylation sites of CrAQPs. In brief, all CrAQPs except for CrXIP1;1 contained all three phosphorylation sites (Ser, Thr, and Tyr; Table 1). We also predicted the subcellular localization of CrAQPs. The two programs used (WoLF_PSORT and Plant-mPLoc) had similar results and most CrAQPs were located in the plasma membrane, although some were located in vacuoles, plastids, and the endoplasmic reticulum (Table 1). The subcellular localizations of CrAQPs showed diverse and broad patterns, indicating that the in vivo compartmentation of CrAQPs is highly variable for each member to regulate transport of water and/or solutes across the plasma membrane and intracellular membrane systems, thereby exercising unique biological functions.

The NPA motifs, ar/R filter, and Froger’s positions of AQPs are critical for their substrate selectivity. A multiple alignment between CrAQPs and other plant AQPs was performed and the conserved NPA motifs and amino acids in ar/R filter and Froger’s positions are characterized in Table 3 and Figure S1. Except for CrPIP2;1, the other 36 CrAQPs all contained two NPA motifs, one in loop B and one in loop E, and most of them were conserved. However, some CrAQPs, such as in CrTIP4;1 and four CrSIPs, displayed a variable third residue in the LB NPA motif, in which the A residue was replaced by S/T/L. In addition, the CrXIP1;1 protein had variable first and third residues in the LB NPA motif (SPV). In loop E, this NPA motif was more conserved and only CrNIP1;3, CrNIP3;1, CrNIP3;2, and CrNIP3;3 showed substitutions of A by V. In CrSIP1;3, the LE NPA motif degenerated into NLG and showed a greater divergence in residues of the two NPA motifs than the other CrAQPs (Table 3). The space between the two conserved NPA motifs varied from 79 to 127 aa and most were between 108 and 119 aa (Table 3). At the ar/R selectivity filters and Froger’s positions, the CrAQPs displayed more differences than in NPA motifs (Table 3). These variabilities determined the substrate specificity of CrAQPs.

### Chromosomal locations and evolutionary characterization of *CrAQP*s

**Fig. 2:**
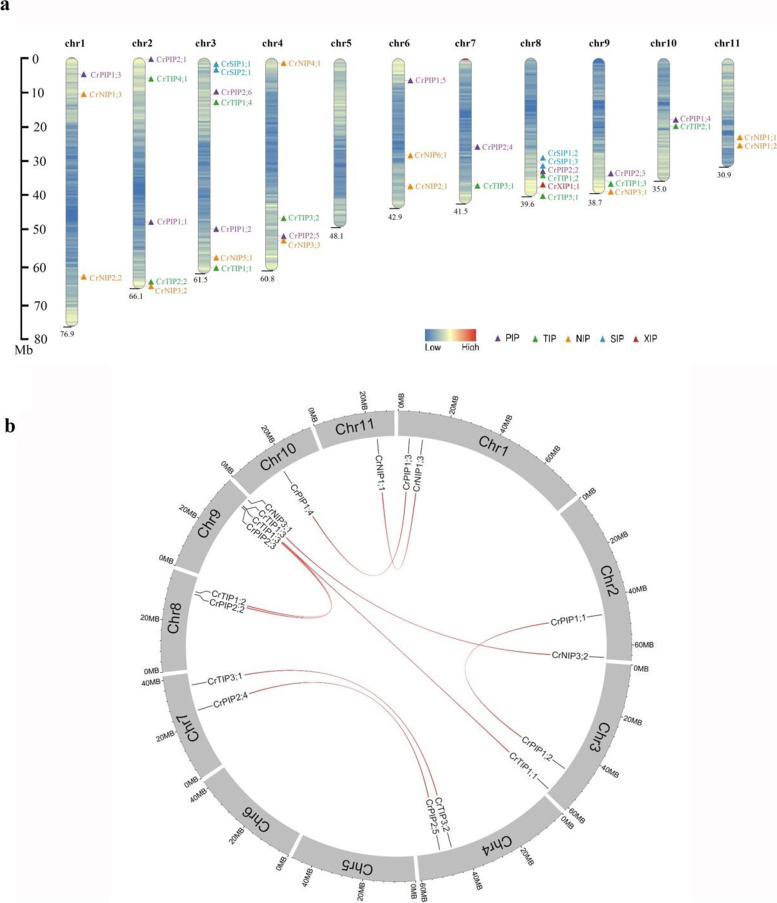
**a** Locations of the 37 *CrAQP*s on 11 chromosomes of *C. rosea*. **b** The distribution of segmental duplication of *CrAQP*s in *C. rosea* chromosomes

To investigate the evolutionary relationship among *CrAQP* genes, chromosome maps were constructed (Fig. 2a). There are eleven chromosomes in the *C. rosea* genome and *CrAQP* genes were found on all except chromosome 5. On the other ten chromosomes, the *CrAQP*s were unevenly distributed. Among them, chromosome 3 had seven *CrAQP* genes, chromosome 8 had six, chromosome 2 had five, chromosome 4 had four, chromosomes 1, 6, and 9 had three, and chromosomes 7, 10, and 11 had two.

Gene duplication events of *CrAQP*s were also investigated. A total of eighteen and four *CrAQP* genes were found to be segmentally and tandemly duplicated, respectively (Table 4). The distribution of segmental duplication of *CrAQP*s in *C. rosea* chromosomes was simply showed in Fig. 2b.The selection pressure acting on *CrAQP* genes was inferred from the ratio of non-synonymous (Ka) to synonymous (Ks) substitution values. Our data indicate that all *CrAQP* genes were under evolutionary pressure, with an average Ka/Ks ratio of 0.1523. All Ka/Ks ratios were well below one (range: 0.0989–0.2738) (Table 4). These results suggest that *CrAQP*s experienced strong purifying selection pressure with limited functional divergence after duplication.

**Table 4:** Ka/Ks analysis and duplication events for *CrAQP* genes

Duplicated pair	Duplicate type	Ka	Ks	Ka/Ks	*P*-value(Fisher)	Positive selection
CrPIP1;1/CrPIP1;2	Segmental	0.073547	0.622897	0.118072	1.87439E-30	No
CrPIP1;3/CrPIP1;4	Segmental	0.111758	0.842367	0.132671	1.34665E-32	No
CrPIP2;2/CrPIP2;3	Segmental	0.06824	0.675207	0.101065	6.37277E-34	No
CrPIP2;4/CrPIP2;5	Segmental	0.048577	0.491175	0.0989	7.70278E-29	No
CrTIP1;1/CrTIP1;3	Segmental	0.140454	1.19439	0.117595	9.9E-36	No
CrTIP1;2/CrTIP1;3	Segmental	0.065171	0.651205	0.100078	2.28E-30	No
CrTIP3;1/CrTIP3;2	Segmental	0.141034	0.515046	0.273827	1.48E-12	No
CrNIP1;1/CrNIP1;3	Segmental	0.170454	0.652004	0.26143	7.81E-16	No
CrNIP3;1/CrNIP3;2	Segmental	0.13405	0.803634	0.166804	1.27E-28	No
CrNIP1;1/CrNIP1;2	Tandem	\	\	\	\	\
CrSIP1;2/CrSIP1;3	Tandem	\	\		\	\

### Gene structures and protein motif compositions

**Fig. 3:**
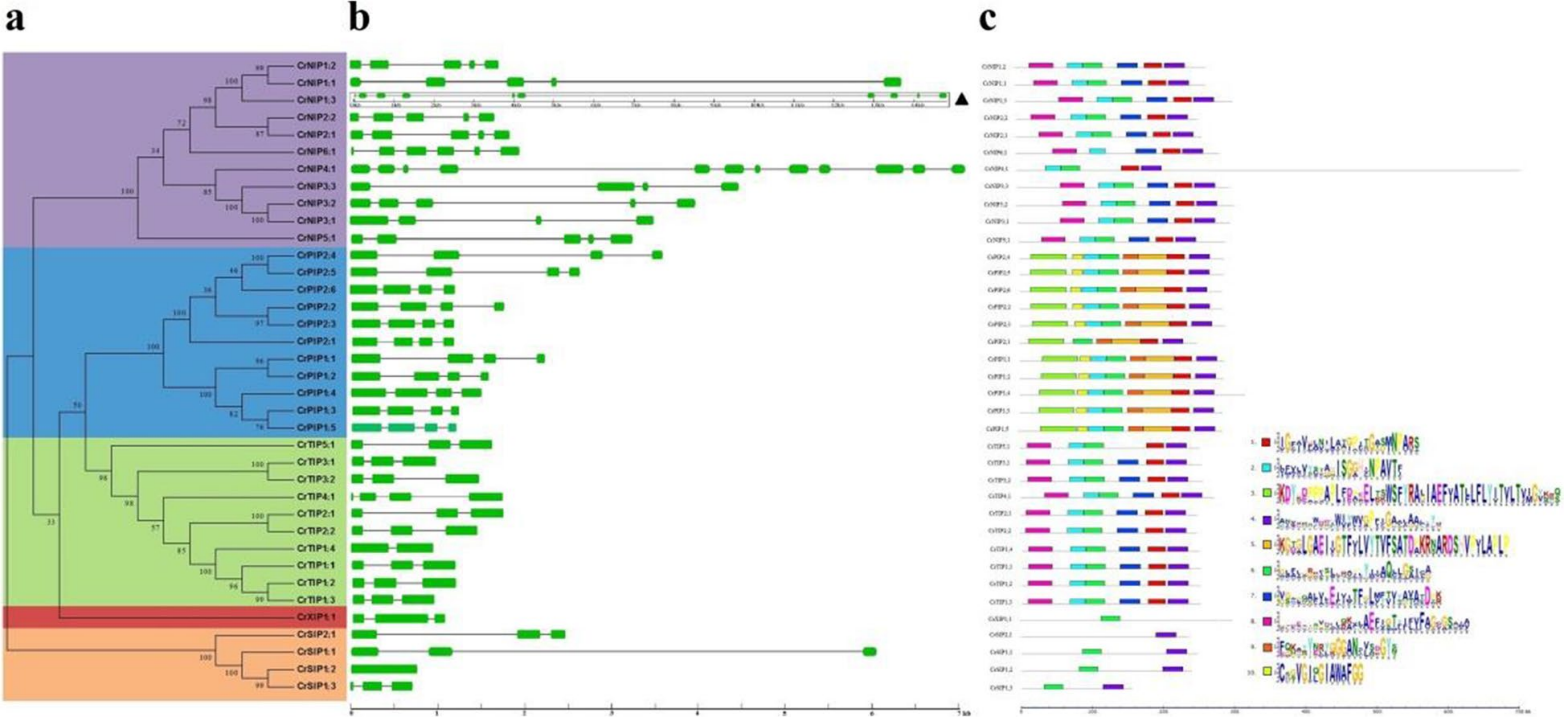
Phylogenetic relationships, genes’ structure, and motif compositions of the *AQP* genes in *C. rosea*. **a** The phylogenetic tree on the left side is constructed using MEGA 6.0. The five major groups are marked with different color backgrounds. **b** The exon–intron organization of the *CrAQP*s is constructed using GSDS 2.0 (in the middle). **c** The conserved motifs of each group on the right side are identified by the MEME web server. Different motifs are represented by different colored boxes, and the motif sequences are provided in Table S4

Gene structure analyses performed using the GSDS tool revealed relatively large variation in the number and length of introns/exons that resulted in *CrAQP*s length variation (720–14,816 bp) across five different *CrAQP* subfamilies (Fig. 3a and b). The number of introns ranged from zero (CrSIP1;2) to eleven (CrNIP4;1). Most *CrNIP*s and *CrPIP*s possessed three to four introns and most *CrTIP*s had two introns, except for CrTIP4;1, which had three introns. Three of four *CrSIP*s had two introns, except for CrSIP1;2, which was intronless. The only *CrXIP1;1* also had two introns.

To further investigate the function of AQPs in *C. rosea*, MEME was used to identify the conserved domains of CrAQP proteins. Among the ten motifs identified, motifs 1, 2, 4, 6, 7, and 8 were widely found in CrNIP and CrTIP subfamilies. Most of the CrPIP members shared conserved motifs 1, 2, 3, 4, 5, 6, 9, and 10. Three of the four CrSIPs shared conserved motifs 4 and 6, all except CrSIP2;1. The CrXIP1;1 protein possessed only motif 6. In general, the motif compositions were similar within each CrAQP protein subfamily (Fig. 3c).

### *Cis*-acting regulatory elements

The regulation of *CrAQP* expression remains a key mediator of *CrAQP* function, especially in response to stress and plant growth and development. The *cis*-acting regulatory elements are a series of nucleotide motifs that bind to specific transcription factors, thereby regulating transcription in plants. In this study, we identified putative *cis*-acting elements in the promoter regions of all of *CrAQP*s by scanning the online PlantCARE program.

**Fig. 4:**
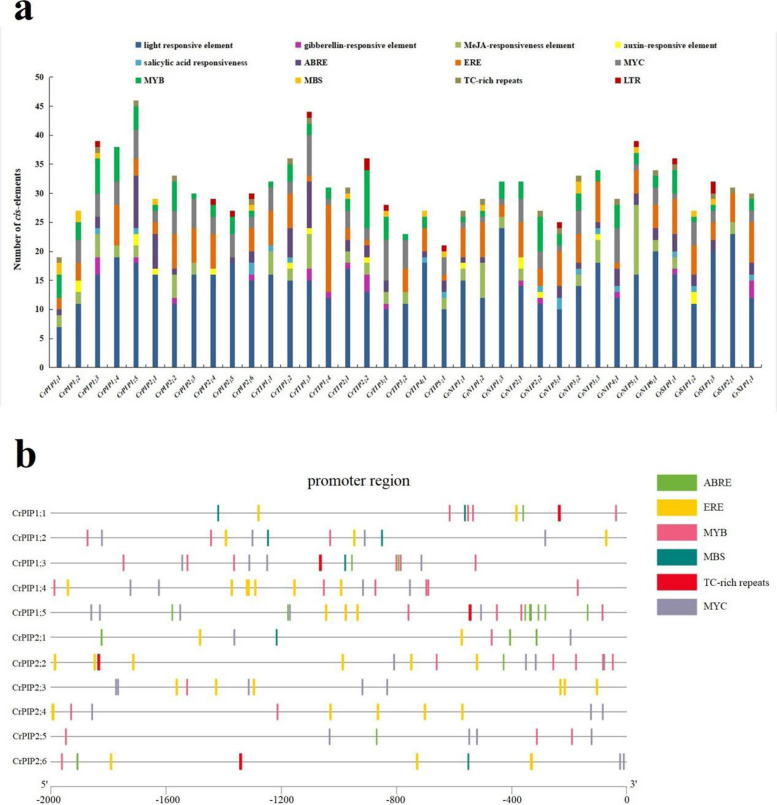
Numbers and distribution of the *cis*-acting elements in the 37 candidate *CrAQP* promoter regions. **A** Summaries of the twelve *cis*-acting elements in the 37 candidate *CrAQP*s promoter regions; **B** Distribution of the six *cis*-acting elements (ABRE, ERE, MYB, MBS, TC-rich repeat, and MYC) in the eleven *CrPIP* promoter regions. The elements are represented by different symbols. The scale bar represents 500 bp

The promoter analyses of all 37 *CrAQP*s identified 68 putative *cis*-acting elements, including 25 light responsive elements, 4 ABA responsive elements, 3 gibberellin-responsive elements, 2 MeJA-responsive elements, 2 auxin responsive elements, 1 ethylene-responsive element, 22 abiotic or biotic stress-related responsive elements, and 18 development-related responsive elements (Table S3). We characterized these elements into 12 categories: light responsive elements, gibberellin-responsive elements, MeJA-responsive elements, auxin-responsive elements, salicylic acid responsive elements, ABRE-, ERE-, MYC-, MYB-, MBS-, and TC-rich repeats, and LTR. The numbers of these elements in each *CrAQP* promoter region are summarized in Fig. 4a. In addition, because PIPs play an important roles in maintaining water balance in plant cells, we summarized the abiotic stress-related *cis*-acting elements (including ABRE, ERE, MYB, MBS, TC-rich repeats, and MYC) within 11 *CrPIP* promoter regions (Fig. 4b). The categories and numbers of these elements suggest that mechanisms regulating *CrPIP* expression are involved in stress responses. However, further functional studies are still necessary to confirm the functions of these *cis*-acting *CrAQP* elements.

### Expression profiles of *CrAQP*s in different tissues and plants residing in different habitats

**Fig. 5:**
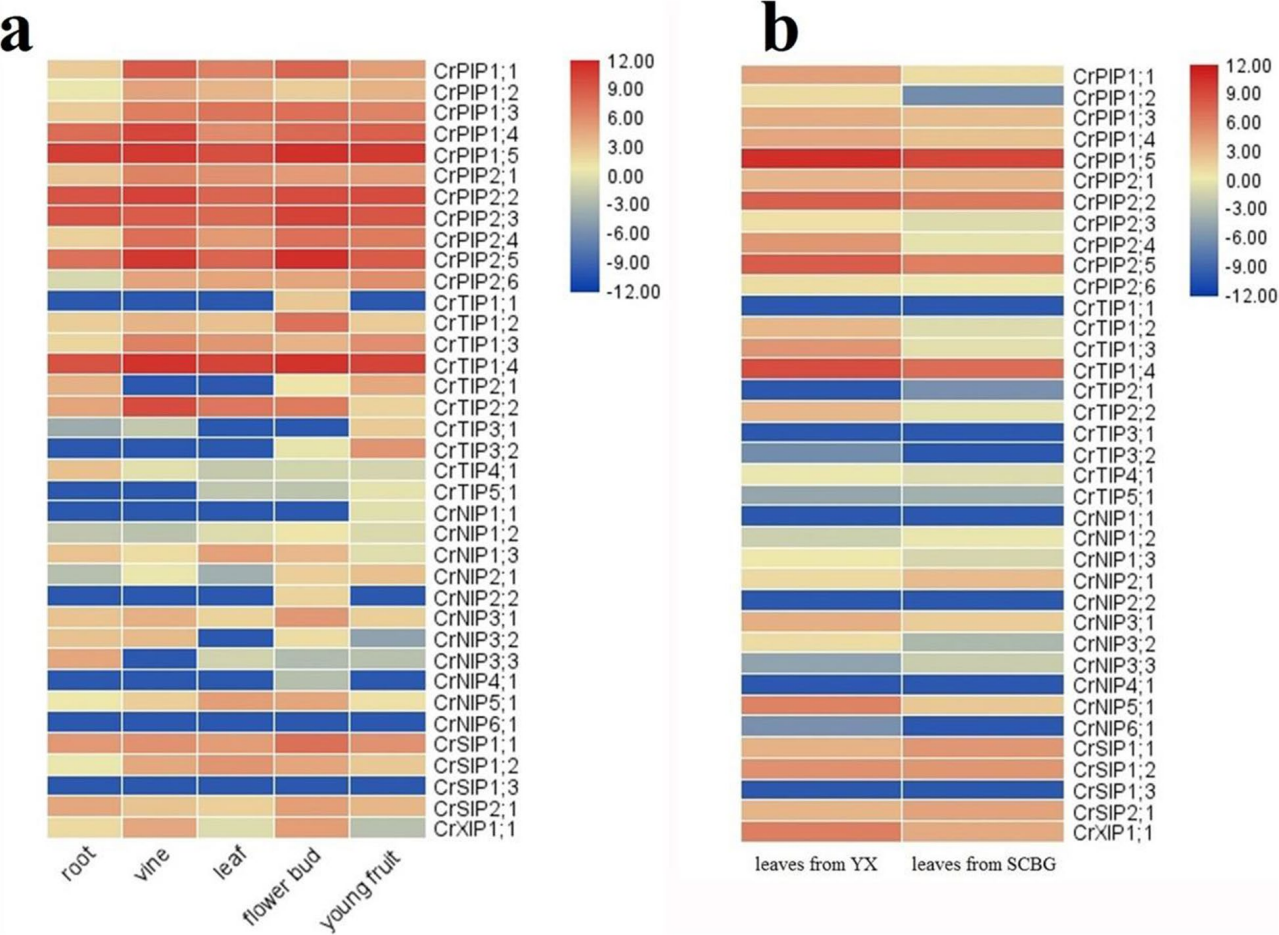
Heatmaps showing (**A**) the expression levels of the 37 *CrAQP*s in the root, vine, leaf, flower bud, and young fruit of *C. rosea* plant and (**B**) expression differences of the 37 *CrAQPs* in mature *C. rosea* leaves planting in South China Botanical Garden (SCBG) and in Yongxing Island (YX)

Tissue- and habitat-specific expression profiles of *CrAQP*s were assessed by examining their Illumina RNA-Seq data representing seven tissue types: roots, vines, young leaves, flowering buds, and young fruits gathered from SCBG, and two mature leaf samples gathered from SCBG and YX Island respectively. Expression of all *CrAQP*s was detected in at least one of the examined tissues, though the transcript level was diverse. Overall, the *CrPIP* members had relatively higher expression in all tissues. The subfamilies *CrPIP* and *CrTIP* also produced abundant transcripts in most examined tissues (Fig. 5).Young flowering buds and young fruits tended to have high levels of *CrAQP* expression across the whole family (Fig. 5a). We also focused on the comparison of *CrAQP*s’ expression levels in adult *C. rosea* leaves collected from various habitats (YX Island and SCBG), and the results indicated that the most of *CrAQP*s expressed higher in the YX sample than in the SCBG sample, particularly the *CrPIP* members (Fig. 5b). These results suggest that *CrAQP*s might play diverse roles in the growth and development of *C. rosea*, and in this extremophile halophyte’s adaptation to coral reef habitats.

### Expression profiles of *CrPIP*s in response to different stressors and the ABA treatment

**Fig. 6:**
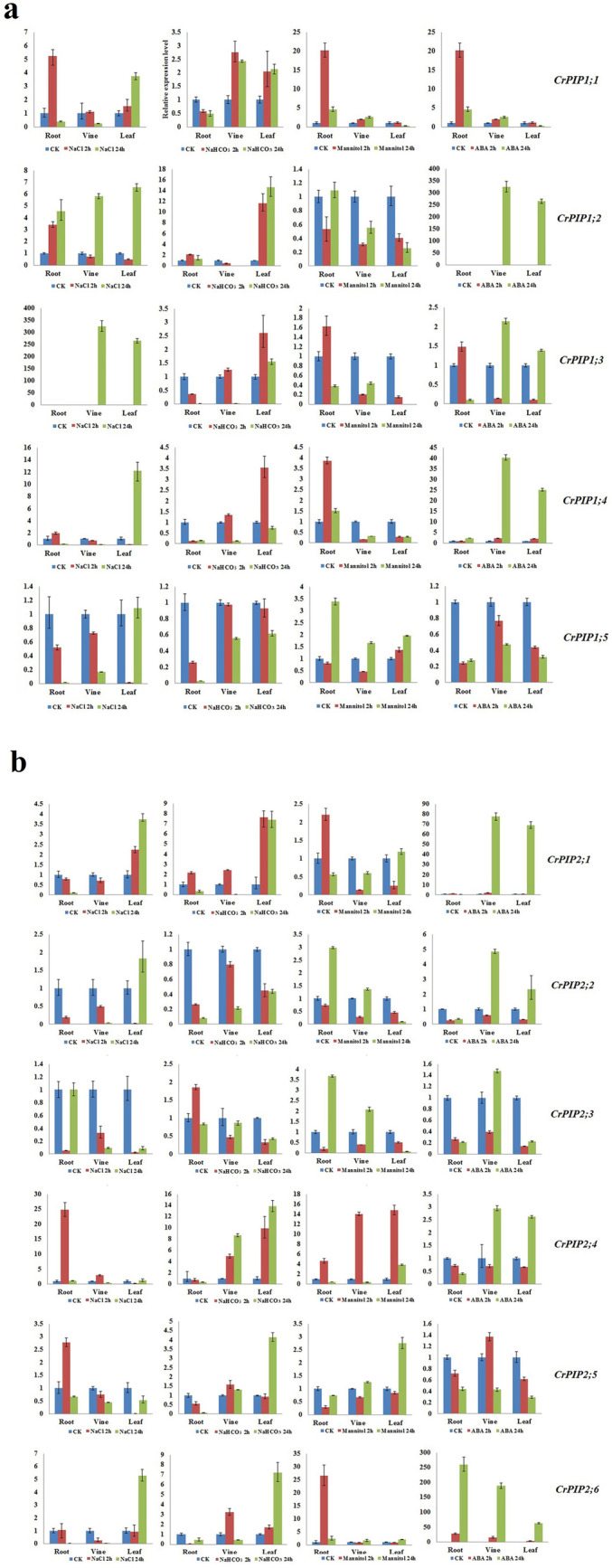
Quantitative RT-PCR detection of the expression levels of the 11 *CrPIP*s responding to different stresses (600 mM NaCl, 150 mM NaHCO_3_, 300 mM mannitol, and 100 mM ABA) in *C. rosea* seedling plants. **a** 5 *CrPIP1*s. **b** 6 *CrPIP2*s. Relative expression values were calculated using the 2 − ΔCt method with housekeeping gene *CrEF-α* as reference gene. Bars show mean values ± SD of *n* = 3–4 technical replicates

We performed a gene expression analysis on different *C. rosea* tissues to examine the expression patterns of *CrPIP* genes under various abiotic stress conditions and an ABA hormone treatment. The purpose of these treatments were to mimic reef and coast adversity as much as possible. We performed qRT-PCR to detect the transcript levels of these subfamily genes. As shown in Fig. 6, expression of all *CrPIP*s was affected by the stressors and hormone application. We also found several *CrPIP* members that showed relatively stable expression patterns, even under the various stressors. These genes included *CrPIP1;5*, *CrPIP2;2*, *CrPIP2;3*, and *CrPIP2;5* (Fig. 6). Combining these results with the RNA-Seq data (Fig. 5), it is evident that these genes maintained higher expression levels than the other *CrAQP* genes across different tissues and habitats, suggesting that they may be involved in maintaining basic and primary water homeostasis during *C. rosea* growth and development. Under high salt stress, *CrPIP1;2* showed all induced expression patterns in roots, vines, and leaves, while *CrPIP1;1*, *CrPIP1;3*, *CrPIP1;4*, *CrPIP2;1*, and *CrPIP2;6* showed elevated expression in vine and leaf, and their expression was downregulated in roots. In general, alkaline stress had a smaller effect on the expression of *CrPIP*s. The genes *CrPIP1;2*, *CrPIP1;3*, *CrPIP1;4*, *CrPIP1;5*, *CrPIP2;1*, *CrPIP2;4*, *CrPIP2;5*, and *CrPIP2;6* were downregulated in root, while *CrPIP1;1*, *CrPIP1;2*, *CrPIP2;4*, and *CrPIP2;5* were slightly upregulated in aerial tissues. High osmotic stress increased the expression of *CrPIP1;1*, *CrPIP2;4*, and *CrPIP2;6*, and the ABA treatment increased the expression of *CrPIP1;2*, *CrPIP1;4*, *CrPIP2;1*, and *CrPIP2;6* (Fig. 6). These results indicate the role these genes play in multiple abiotic stress responses and ABA signaling response in *C. rosea*.

### Interactions between CrPIP1;5 and CrPIP2;3

**Fig. 7:**
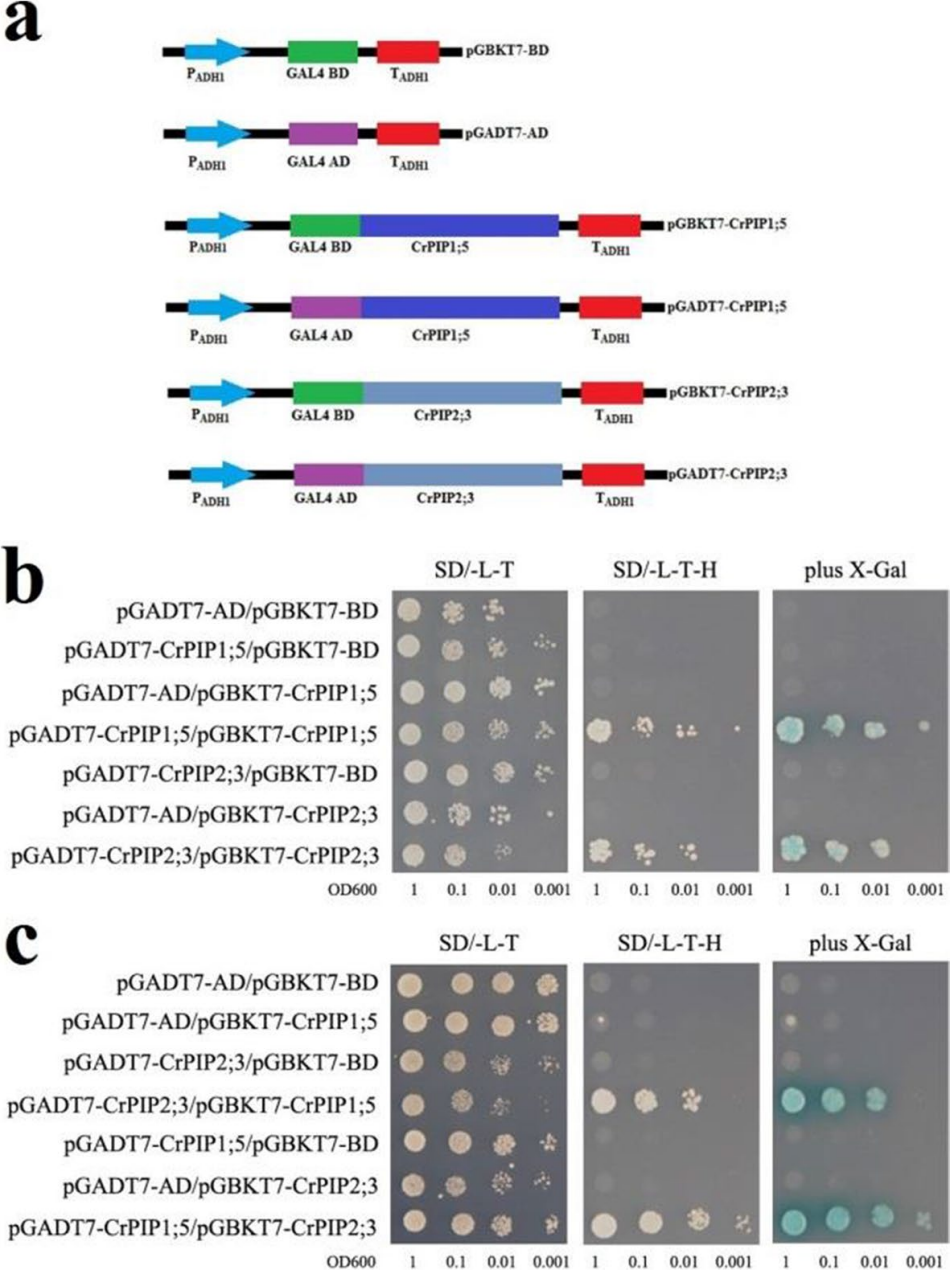
Homodimer or Heterodimer of the CrPIP1; 5 and the CrPIP2;3 detection by yeast two-hybrid assay. **a** Maps of different constructs. **b** Both the CrPIP1;5 and the CrPIP2;3 showed self-interacting. **c** The CrPIP1;5 and the CrPIP2;3 could interact each other

A previous study indicated that plant PIP1 and PIP2 members can associate together in heterodimers and tetramers [35]. In our previous study, we have functional identified a *C. rosea PIP2* gene, *CrPIP2;3*, being involved in drought tolerance in transgenic plant [36]. *CrPIP1;5* and *CrPIP2;3* were both initially isolated from *C. rosea* cDNA library, and these two members showed much higher expression level in different tissues of *C. rosea* than other *CrAQP*s (Fig. 5a), which might indicate their relative importance of maintaining water balance in vivo. In this study we analyzed two PIP members, CrPIP1;5 and CrPIP2;3, to confirm that CrAQPs could form homodimers or heterodimers. To explore CrPIP1;5-CrPIP2;3 interactions, a series of DNA constructs were prepared for a yeast two-hybrid assay (Fig. 7a). BD and AD vectors were co-transformed into yeast AH109. Both CrPIP1;5 and CrPIP2;3 did not self-activate, but both can form homodimers through direct interactions with themselves(Fig. 7b). Furthermore, CrPIP1;5 and CrPIP2;3 can interact with each other (Fig. 7c). Together, these results indicate that, at least in yeast cells, these two CrPIP members can interact with themselves and each other to form active pores for water and small molecule transport across membranes.

### Abiotic stress tolerance of yeast and Arabidopsis heterologously expressing *CrPIP1;5*

**Fig. 8:**
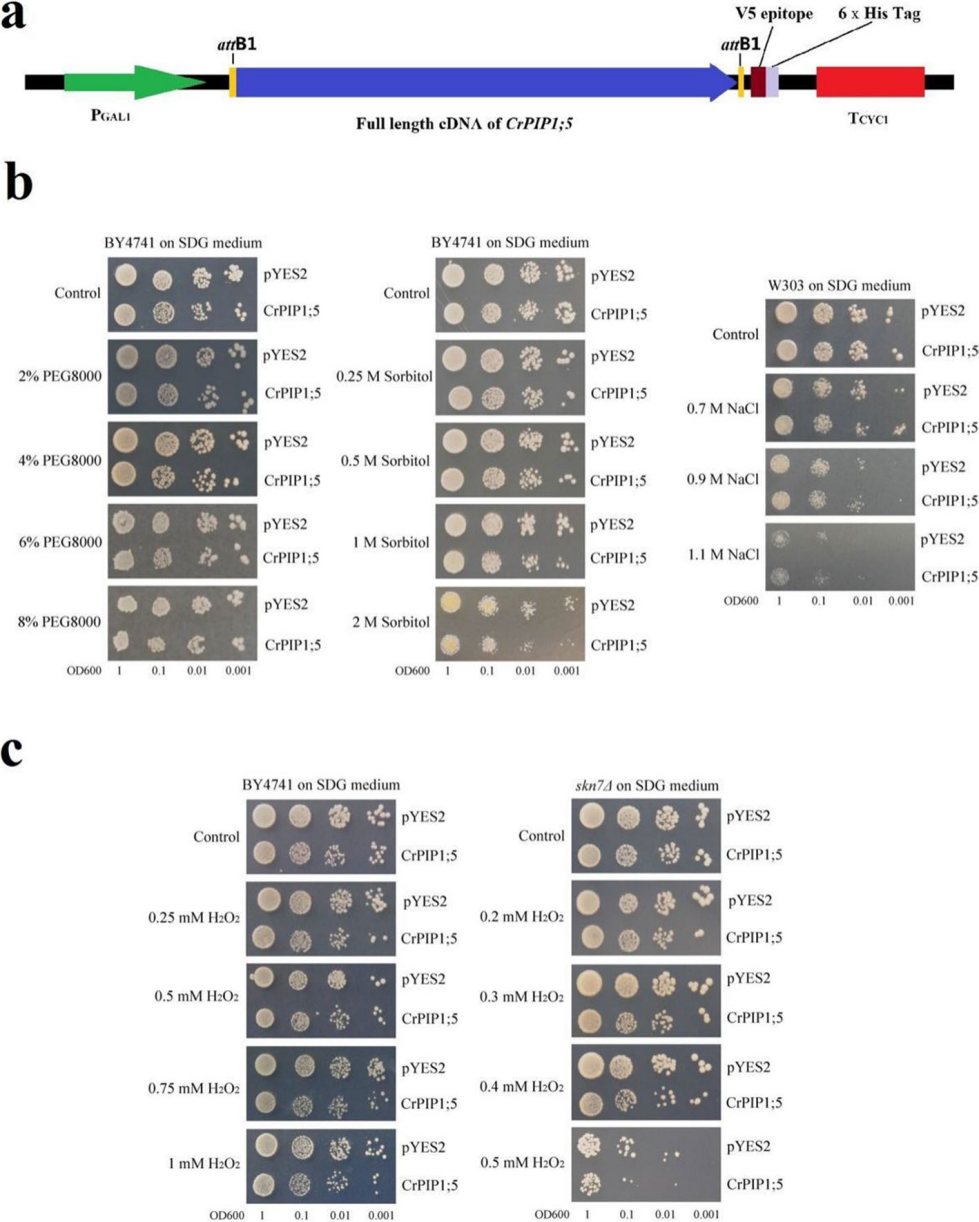
The spot assays for stress tolerance confirmation of the *CrPIP1;5* expression in yeast. **a** Map of the CrPIP1;5-pYES-DEST52 construct. **b** High osmotic stress and salt tolerance confirmation in yeast. The growth state of yeast cells (WT, BY4741) transformed with CrPIP1;5-pYES-DEST52 and empty vector pYES2 with or without PEG8000, sorbitol, or NaCl on SDG-Ura plates. The concentration of these stress factors for each treatment was labeled on the left. **c** H_2_O_2_ oxidative stress tolerance confirmation in yeast. The growth state of yeast cells (WT and H_2_O_2_-sensitive mutant strain *skn7Δ*) transformed with CrPIP1;5-pYES-DEST52 and empty vector pYES2 with or without H_2_O_2_ on SDG-Ura plates. The concentration of H_2_O_2_ for each treatment was labeled on the left

We performed functional identification of *CrPIP1;5* using a yeast expression system, constructing with a *CrPIP1;5*-pYES-DEST52 recombinant vector (Fig. 8a). As seen in Fig. 8b, W303 transformed with either *CrPIP1;5* or pYES2 developed normally and did not differ in growth rate from the SDG control plate. However, with the addition of PEG8000 or sorbitol, W303 transformed with *CrPIP1;5* showed an obvious growth lag compared to yeast containing the pYES2 control. When NaCl was added to the SDG medium, the W303 yeast containing *CrPIP1;5* showed better growth than the control (Fig. 8b). We also checked H_2_O_2_ transport activity using the yeast expression system. *CrPIP1;5* resulted in increased H_2_O_2_ sensitivity of yeast and lower growth rates, while both the BY4741 strain and the H_2_O_2_-sensitive mutant strain *skn7Δ* showed similar growth performance to the SDG control plate (Fig. 8c). These results indicate that, at least in yeast cells, CrPIP1;5 is an active H_2_O and H_2_O_2_ transporter.

To further assess the effects of *CrPIP1;5*, we generated transgenic *Arabidopsis* plants that ectopically expressed *CrPIP1;5* under the control of 35S promoters. The plants were confirmed as transgenic using genomic PCR, RT-PCR, and qRT-PCR (Figure S2). Plants from three homozygous T3 lines (*OX 1#*, *OX 5#*, and *OX 10#*) were subjected to the salt, salt-alkaline, high osmotic, and drought tolerance tests. Although our seed germinating assays indicated that *CrPIP1;5 OX* lines showed no significant difference on germination rates under salt, salt-alkaline, or high osmotic challenges compared with WT seeds (Figure S3), while in the seedling growth assays, *CrPIP1;5 OX* line seedlings showed slightly growth retardation on the salt and salt-alkaline MS plates compared with WT control (Figure S4).

**Fig. 9:**
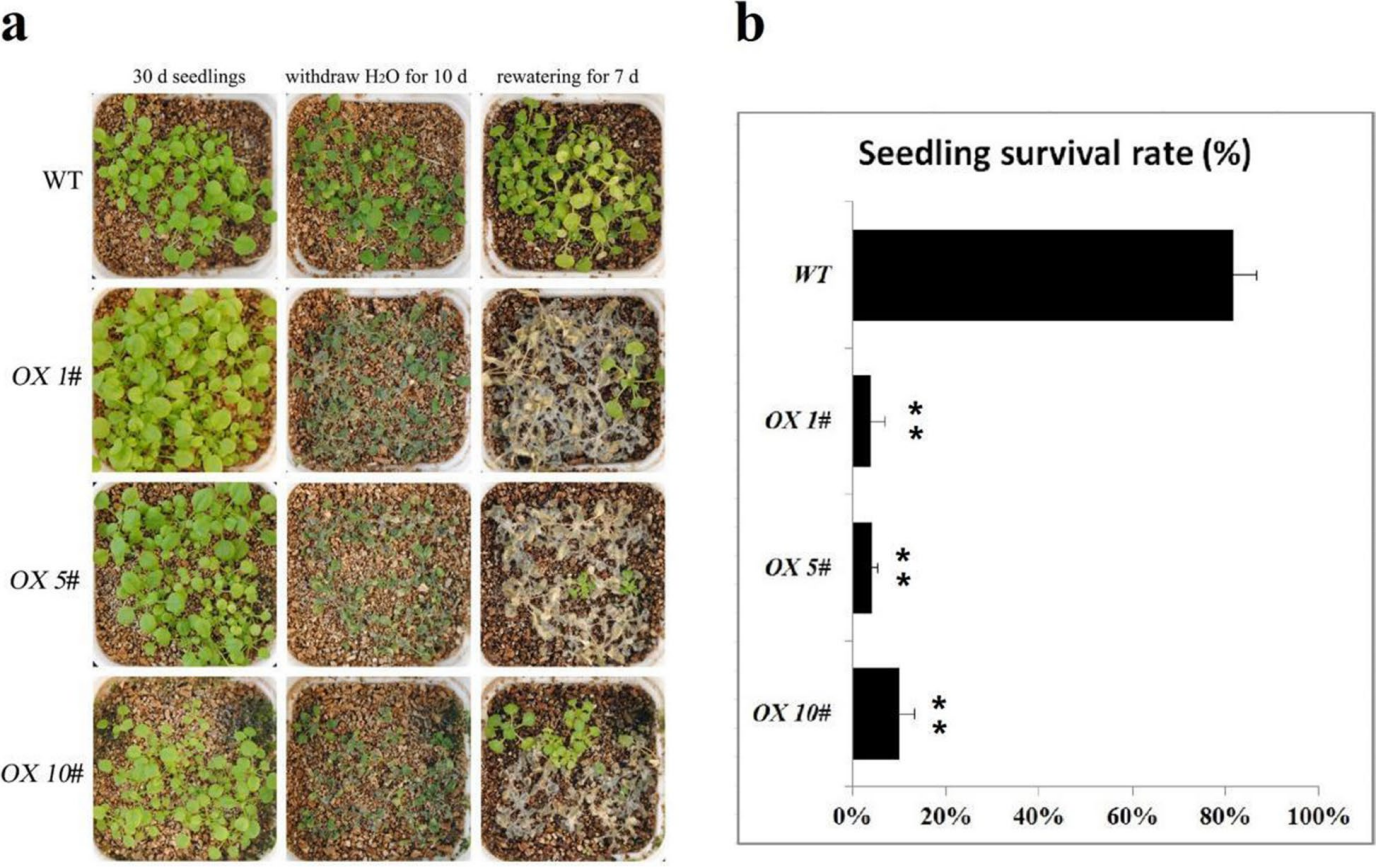
Drought stress treatment on the growth of the transgenic *CrPIP1;5* overexpression lines (*OX 1#*, *OX 5#*, and *OX 10#*) and wild-type (WT) plants under natural growth conditions in vermiculite. **a** Leaf phenotypes of the transgenic line and WT plants under drought treatment. **b** The survival rates of the *OX* lines and WT plants. Bars show mean values ± SD of *n* = 3–4 biological replicates. The significance level was defined as * (*P* < 0.05) and ** (*P* < 0.01)

The seeds of WT and *CrPIP1;5 OX* lines were grown in well-watered conditions for 30 days, and prior to the salt, drought, and alkaline stress treatments, the growth rates of adult plants (WT and three *CrPIP1;5 OX* lines) were relatively consistent. There was no difference in tolerance between WT and transgenic plants (*OX 1#*, *OX 5#*, and *OX 10#*) under salt (200 mM NaCl) and salt-alkaline (100 mM NaHCO_3_, pH 8.2) stressors (Figure S5). Apparently, *CrPIP1;5* resulted in weak sensitivity to drought (Fig. 9a). After 10 days of water withdrawal, all plants wilted to some degree in both WT and the three *CrPIP1;5 OX* lines. After re-watering and growing for another 7 days, most of the *CrPIP1;5 OX* plants did not recover, while most of the WT plants did recover and had a higher survival rate than *CrPIP1;5 OX* plants (Fig. 9b). This result indicates that overexpression of *CrPIP1;5* decreased plant resistance to drought.

We found that *GFP*-fused *CrPIP1;5* was constitutively expressed (under the control of CaMV 35S) in transgenic *Arabidopsis* plants. Root tip fluorescence of roughly three- to four-day-old transgenic *Arabidopsis* seedlings was easily discerned by confocal microscopy; the GFP-CrPIP1;5 protein was visible in the plasma membranes of transgenic plants, while in control plant roots, the GFP signal was distributed evenly in the whole cytoplasm (Figure S6). These results suggest that subcellular localization of CrPIP1;5 was consistent across the PIP1 subfamily and was predominantly localized to the plasma membrane, as well as partly in endomembrane system. Within the plasma membrane, CrPIP1;5 was folded into a specific transmembrane channel and functioned as a water transporter.

## Discussion

Water deficit—caused by drought, high salinity/alkaline, high temperature, cold/freezing conditions or other abiotic stressors—can negatively affect plant growth and survival. However, plants have developed intricate mechanisms to cope with this type stress, including alterations to signal perception and transduction and differential expression of stress responsive genes through complex networks. Aquaporins are a class of integral membrane proteins that facilitate the diffusion of water and other small solutes. Plants often maintain large and diverse AQP families compared to animals and microorganisms. Aquaporins have been reported to play crucial roles in plant water balance and homeostasis under adverse growing conditions [5, 21] and in response to specific biotic challenges [37, 38]. In this study, we performed genome-wide identification and characterization of AQPs in *C. rosea* to understand the evolution of this family and its molecular roles. We were particularly interested in resolving the molecular mechanisms underlying this extremophile halophyte’s adaptation to coral reef habitats and its responses to acute salt, alkaline, and drought stressors.

The AQP protein family within the *C. rosea* genome was characterized and 37 putatively functional CrAQP isoforms (based on Pfam domain sequences) were identified, belonging to the PIP (11 isoforms), TIP (10), NIP (11), SIP (4), and XIP (1) subfamilies (Table 1). We performed whole genome sequencing of *C. rosea*, and our result indicates that this species is diploid, with a 534.94 Mbp genome size (data not published). The number of *AQP*s was similar to other diploid plant species (Table 2) and their protein sequences were highly similar. This indicates that the number of *AQP*s and sequence specificity may not be directly related to the adaptation of *C. rosea* to extreme environments. The roles that *CrAQP*s play in stress tolerance needs to be further studied from other perspectives, such as transcriptional regulation, protein modification, and the regulation of AQP transmembrane transport activities.

Although numerous studied have identified AQPs in model plant species, research on this gene family has increasingly focused on plants that inhabit novel environments. This is largely because *AQP* genes are seen as candidates for use in genetic modification of crops to increase agricultural productivity [39, 40]. The saltbush *Atriplex canescens* is highly tolerant of saline-alkaline soils, drought, heavy metals, and cold, and the *AQP* genes *AcPIP2* and *AcNIP5;1* have been shown to be involved in abiotic stress tolerance in this species, and their overexpression in transgenic *Arabidopsis* caused altered tolerance to drought and salt [41, 42]. Compared with cultivated soybean, the wild *Glycine soja* is relatively salt-alkaline tolerant. Two *AQP* genes from *G. soja*, *GsTIP2;1* and *GsPIP2;1*, minimized tolerance to salt and dehydration stress when overexpressed in *Arabidopsis*, implying they have negative impacts on stress tolerance by regulating water potential [43, 44]. In most functional analyses conducted in transgenic plants, the overexpression of *AQP* genes caused elevated tolerance to salt and drought, such as in *Malus zumi* (gene *MzPIP2;1*) [45], *Sesuvium portulacastrum* (*SpAQP1*) [46], *Stipa purpurea* (*SpPIP1*) [47], *Simmondsia chinensis* (*ScPIP1*) [48], *Thellungiella salsuginea* (*TsPIP1;1*) [49], and *Phoenix dactylifera* (*PdPIP1;2*) [50]. The elevated expression of *AQP* genes in plants can lead to cellular changes in water potential, which cause alterations in water uptake and transpiration, and ultimately modify tolerance to water deficit stress. In this respect, understanding the distribution, expansion, regulation, phylogenetic diversity, and evolutionary selection of *AQP* genes in extremophile plants like *C. rosea* is an important step toward potentially improving the water utilization abilities and drought adaptations of other plant species, including agricultural crops.

Plant AQPs play versatile physiological roles in combatting abiotic stress, not only by regulating water content and potential, but also by transporting certain signaling molecules and nutrients. Generally, AQPs consist of six transmembrane helices connected by five loops (A–E) and cytosolic N- and C-termini. Loops B (cytosolic) and E (non-cytosolic) both contain the highly conserved NPA (asparagine-proline-alanine) motifs that form part of the core of these proteins. The aromatic/arginine (ar/R) constriction is located at the non-cytosolic end of the pore. The substrate specificity of AQPs is closely related to several different signature sequences, including NPA motifs, the ar/R filter, and Froger’s positions (FPs) [51]. In all CrAQP NPA motifs, the first two residues were the most conserved, except for CrSIP1;3 and CrXIP1;1, in which the loop B and loop E NPA motifs degenerated into NLG and SPV. The third residue of NPA motifs was more variable, in which A was frequently replaced by either L, S, T, or V. However compared to the NPA motifs, the 10 amino acid residues at the ar/R filter and Froger’s positions were more variable in all CrAQPs (Table 3). In some subfamilies, the ar/R selectivity filter sequences were similar, such as in CrPIPs (F–H-T-R), CrTIP1s (H-I-A-V), and CrNIP1s (W-V-A-R). We also analyzed Froger’s positions (P1–P5), five conserved amino acid residues that are related to glycerol transport in water-conducting AQPs. The P2, P3, P4, and P5 Froger’s positions in CrPIPs were relatively conserved (S-A-F-W), and in CrTIPs, they were less conserved (S-A-Y/F-W). In CrNIPs and CrSIPs, the P3 and P4 positions mostly stayed A and Y. It is supposed that plant TIPs may transport various small solutes, including H_2_O_2_, NH_4_^+^, and urea, in addition to water [40]. As with other plant TIPs, CrTIPs are mainly located in vacuolar membranes and may be involved in the regulation of water flow across subcellular compartments of organelles [52]. The variation of CrTIPs in ar/R selectivity filter sequences may contribute to their multiple transport functions, and their NPA spacing varies from 79 to 127 amino acid residues, which indicates that CrTIPs might also be involved in the transmembrane transport of multiple small molecules.

Gene structure organization, gene expansion, and gene diversity are critical indicators of the evolution of gene families. The *CrPIP* and *CrTIP* subfamilies exhibit relatively stable gene structure in comparison with other subfamilies (Fig. 3). Most of them possess three (*CrPIP*s) or two (*CrTIP*s) introns, suggesting that they might share a common ancestral origin. Similar to previous reports showing very few or no intronless *AQP* genes in other plant species [30, 31, 53], only one intronless *AQP* was identified in *C. rosea*. The intronless gene, *CrSIP1;2*, might have evolved recently through a retrotransposon process. The *CrAQP* family has undergone a number of duplication events consistent with the highly duplicated nature of plant genomes (Fig. 2; Table 4). The duplication events concerning segmental and tandem duplications identified in this study have also been reported in other plant species [33, 34]. In the present study, some duplicated *CrAQP*s have distinct patterns of expression in different tissues and habitats, and under different stressors and hormone exposure (Figs. 5 and 6). It is likely that these duplicated gene pairs have similar protein functions yet function in different biological processes, probably mediated by transcriptional regulation or posttranscriptional modification.

*Canavalia rosea* is a salt- and alkaline-tolerant and drought-adapted halophyte, and abiotic stressors, such as saline-alkaline soil, seasonal drought, strong solar irradiance, and high temperatures, are the main limiting factors that induce osmotic stress and disturb water balance for this species and other tropical seaside plants. For regulating the water transport under different abiotic stresses efficiently, plant often changes the expression of aquaporin genes through a series of complex steps to control the water uptake or management. However, the water management effectiveness and patterns could always depending on the plant growth conditions or tissue type, as well as the different types or degrees of water stress and the specificity of AQP members [54]. In plant, the *PIP* isoforms were supposed to playing major roles in maintaining plant water homeostasis and responses to abiotic stress [11, 55]. Gene transcript levels are dependent upon the structures of their promoters. Therefore, the *cis*-acting elements in promoter regions might provide the key to understanding genetic factors influencing the responses of signal molecules and environmental elicitors. We summarized the abiotic stress-related *cis*-acting elements in *CrAQP* promoters (Fig. 4) and our findings suggest diversity in *CrAQP* expression patterns, which could be also considered as a part of the adaptation mechanisms to stress conditions. Chickpea (*Cicer arietinum* L.) is an important food legume crop with good drought and salinity tolerance than most of other crops [31, 32]. The promoter analyses of *CaAQP*s also identified a number of *cis*-regulatory elements, including defense and stress responsive elements. This promoter analysis was also partly support by the experimental expression analyses some of selected *CaAQP*s, while was not absolutely consistent with the expression patterns of *CaAQP*s [32]. Furthermore, the expression profiles of *CrAQP*s in different tissues revealed by RNA-Seq indicate that some of the *CrPIP* and *CrTIP* subfamilies had higher expression levels than other subfamilies (Fig. 5a), and habitat-specific RNA-Seq data acquired from leaves further indicated the most of the *CrPIP* members had greater expression levels in coastal *C. rosea* (YX) than in inland *C. rosea* (SCBG; Fig. 5b). Similar results were also observed in leguminous plant, chickpea (*Cicer arietinum* L.) [32]. In the drought-tolerant genotype chickpea, two subfamilies (CaPIPs and CaTIPs) showed high expression in all tissues as compared to other subfamilies, which indicated these two subfamilies were essential for water transportation and carried drought tolerant. The qRT-PCR results also showed some of PIP members showed much higher expression level in drought-tolerant genotype chickpea than in drought-tolerant genotype chickpea [32]. Our results suggest that differential expression of *CrPIPs* might be associated with different water use strategies in different habitats, and the higher expression level of *CrAQP*s in coastal *C. rosea* plants might be an adaptive mechanism to deal with intracellular and extracellular water-deficit signals. Due to the material limitation, and we cannot collect enough and intact root tissues from YX Island for RNA-seq analysis, so we mimicked the stresses in lab, and analyzed the gene expression with qRT-PCR. The expression patterns of *CrPIP*s under salt, alkaline, and drought stress and the ABA hormone treatment were further investigated (Fig. 6). The results showed that *CrPIP* expression was most affected under the saline-alkaline, high osmotic stress, and ABA treatments. Furthermore, some *CrPIPs* showed clearly different and even opposite expression patterns in roots, vines, and leaves. This can be attributed to the fact that in roots the PIP proteins mainly facilitate water absorption from external environments, while in vines and leaves, the PIPs may play a larger role in transpiration. Broadly, our results suggest a role for *PIP*s in regulating *C. rosea* hydraulics and probably adaptation to the challenging environmental conditions found on tropical coral reefs and islands.

In our previous research, we have characterized *CrPIP2;3* as a salt/drought stress related gene [36]. Here we performed protein–protein interaction studies using yeast two-hybrid assays and found that two CrPIP members, encoded by *CrPIP1;5* and *CrPIP2;3*, that were highly expressed in all tested tissues and almost constitutively expressed under the abiotic stress challenges and ABA treatment (Figs. 5a and 6). These two CrPIP members could bind to themselves and each other to form homodimers and heterodimers (Fig. 7). This is consistent with previous findings that some PIP1 and PIP2 members could assemble as homotetramers and heterotetramers, thereby triggering channel activities, influencing substrate specificity, and regulating PIP trafficking [56]. Here, our results on the expression patterns of *CrPIP1;5* and *CrPIP2;3* provide a detailed understanding of their regulatory modes and help to illuminate *CrAQP* functions. These data are especially helpful for characterizing AQP-interacting protein complexes involved in *C. rosea*’s adaptations to harsh environmental conditions such as low water availability and saline-alkaline soils.

Our results from the yeast overexpression system indicate that CrPIP1;5 is an active transmembrane H_2_O and H_2_O_2_ transporter (Fig. 8). We assessed the overexpression of *CrPIP1;5* in transgenic *Arabidopsis*, and *CrPIP1;5* lead to slightly reduced saline-alkaline and drought tolerance, which showed the exact opposite phenotype compared with *CrPIP2;3*’s overexpression in *Arabidopsis* [36]. This also suggests that *CrPIP1;5* could play a key role in water transport. Here we speculated that as a foreign AQP gene, *CrPIP1;5* might be involved in modifying the function of endogenous PIPs of Arabidopsis, or function more in water flowing out than water absorption in transgenic *Arabidopsis* roots, thereby resulted in sensitivity to salt and drought stresses. We also found that high levels of salt, alkaline, and ABA slightly decreased the expression of *CrPIP1;5* in *C. rosea*, this further suggests that this gene is highly important for water movement between cells and tissues, and is indeed involved in a stress response pathway that protects yeast cells or plants from water loss under high salinity conditions and promotes water release under high osmotic stress or drought (Figs. 8b and 9). The overexpression of *CrPIP1;5* in transgenic *Arabidopsis* is in contrast to most previous findings [40, 54], suggesting that overexpression of plant PIPs results in specificities of abiotic stress tolerance or sensitivity, which also might be attributed to the protein interaction, post-translational modifications (PTM), protein trafficking of the foreign AQP. While in *C. rosea*, our results about the expression pattern of *CrPIP1;5* regarding to different tissues or habitats (Fig. 5), as well as the transcriptional changes responding to salinity/alkaline, high osmotic stress, and ABA treatment (Fig. 6a), indicated that *CrPIP1;5* might be boiled down to a “housekeeping gene” for basic water homeostasis, to some degree. Even the *CrPIP1;5* showed higher expression level in YX sample than in SCBG sample (leaves) (Fig. 5b), which might be probably due to the long-term adaptive mechanism that *C. rosea* plants on YX island have to deal with much tougher water-deficit adversities than plants in SCBG, including water absorption from the external environment and water transportation in vivo. The slightly decreased expression of *CrPIP1;5* in *C. rosea* seedlings caused by stress factors or ABA (Fig. 6a), which might be an emergency protection for alleviating the damages caused by water disturbances, since we only checked the *CrPIP1;5*’s expression changes in 24 h challenged by different factors. Many studies also proved that the overexpression of plant PIPs could increase sensitivity to drought stress. For example, tobacco PIP1 member, NtAQP1, caused a decline of root hydraulic conductivity and decreased resistance of plants to water stress [57]. Transgenic tobacco (*Nicotiana tabacum*) plants overexpressing *AtPIP1;4* and *AtPIP2;5* displayed rapid water loss under dehydration stress and showed enhanced water flow under drought stress [58]. The *Glycine soja* gene *GsPIP2;1* negatively impact salt and drought stress tolerance by regulating water potential when overexpressed in transgenic *Arabidopsis* [44]. In addition, *Arabidopsis* plants overexpressing *AcPIP2* (a PIP gene from saltbush *A. canescens*) exhibited drought-sensitive phenotypes [41]. This can be explained by the nature and intensity of stresses, combining with the cooperation between the over-expressed foreign AQPs and the endogenous AQPs. When the water-deficit stress signals were perceived, the foreign AQPs’ over-expression might affect the expression patterns and distribution of endogenous AQPs, and as a result the plant stress-responsive effects might vary among plant species. In our study, the over-expression of *CrPIP1;5* showed some sensitivity both in yeast and in Arabidopsis, which only reflect *CrPIP1;5* did involve in water stress responses in vivo. That, combined with our previous related research about *CrPIP2;3* [36], suggested that overexpression of these two PIP genes in transgenic Arabidopsis promoted plant responses to abiotic stressors by maintaining water homeostasis, as well as decreasing the damage caused by water-deficit stress. Overall, as an active water channel protein with high expression levels in *C. rosea* different tissues or challenged by different factors, the *CrPIP1;5*, as well as the *CrPIP2;3*, might be basic sustainers for water homeostasis during the development of *C. rosea* plants.

## Conclusions

The leguminous nitrogen-fixing plant, *C. rosea*, presents extreme saline-alkaline and drought resistance and is used as pioneer species on islands and reefs for artificial vegetation construction. In the present study, we conducted a genome-wide analysis and characterization of *AQP*s in *C. rosea*. Our results will be helpful for understanding the involvement of this gene family in adaptation to stressful abiotic conditions, particularly through its impact on water balance. We determined that the *CrAQP* family consists of 37 members distributed across five subfamilies. Each member had subtle variations in gene and protein structures, transcriptional regulation, subcellular localization, substrate-specificity, and post-translational regulatory mechanisms. Expression profiling of *CrAQP*s revealed higher expression of PIP-associated genes in almost every tissue of *C. rosea* plants, suggesting that this subfamily likely plays important roles in developmental processes and abiotic stress responses. As predicted, the two PIP1 and PIP2 members, CrPIP1;5 and CrPIP2;3, formed homodimers and heterodimers through protein interactions. We also functionally identified one of the *CrPIP1* members, *CrPIP1;5*, given its highest expression levels in different tissues of *C. rosea*. Although our results showed that overexpression of *CrPIP1;5* could increase sensitivity to saline-alkaline and drought conditions in yeast and plants, combining the high and regulatable expression of *CrPIP1;5* in *C. rosea*, it can be inferred that *CrPIP1;5* is one of basic and important functional genes for facilitating water transportation in vivo, and might be involved in the *C. rosea* ecological adaptation to tropical coral reef. The identification of CrAQPs in this study will be useful for further investigation of the roles that *AQP*s play in the various developmental stages and physiological processes of *C. rosea*, as well as elucidating the possible ecological adaptation mechanisms of *C. rosea* to extreme environments, and identification candidate genes for potential introduction into transgenic agricultural crops.

## Methods

### Plant materials

*Canavalia rosea* plants growing on Yongxing Island (YX, 16˚83′93′′ N, 112˚34′00′′ E) and in the South China Botanical Garden (SCBG, 23˚18′76′′ N, 113˚37′02′′ E) were used in this study. The *C. rosea* plants growing in SCBG were introduced gradually from coastal area in Hainan Province since 2012, and grew steadily for one to seven years with regular water and fertilizer supply in Guangzhou, China. To analyze tissue-specific transcriptional patterns of the identified *CrAQPs*, roots, stems, leaves, flowers, and fruits were gathered from *C. rosea* plants grown in SCBG. In addition, to investigate the involvement of the *CrAQPs* in adaptation to different habitats, adult leaves were gathered from *C. rosea* plants growing in both YX and SCBG. In brief, the *C. rosea* tissues were collected outdoors and immediately frozen in liquid nitrogen, then the samples were stored at − 80 °C for subsequent RNA-seq analysis. Three independent biological replicates were used.

### Identification of *CrAQP* genes and gene duplication analysis of the CrAQP family

To identify all putative *CrAQP* genes, the genome database of *C. rosea* (data not published) was used to obtain DNA and protein sequences (Table S1). In brief, DIAMOND [59] and InterProscan (https://www.ebi.ac.uk/interpro/search/sequence/) were used to identify all *C. rosea* proteins with conserved domains and motifs (e < 1e^−5^), and all proteins were annotated using InterPro and Pfam databases (http://pfam.xfam.org/). The Pfam ID (MIP, PF00230) was used to search the CrAQP protein family, and putative sequences of CrAQP proteins were identified and submitted to SMART (http://smart.embl-heidelberg.de/) and the NCBI Conserved Domain Database (https://www.ncbi.nlm.nih.gov/Structure/cdd/wrpsb.cgi) to confirm presence of the AQP domain. Next, the selected *CrAQPs* were named based on their sequence homology with known AQPs and *C. rosea* genome annotation.

Gene segmental and tandem duplications were assessed using MCScanX software (http://chibba.pgml.uga.edu/mcscan2/), and tandem duplications were also checked manually according their gene loci. The number of synonymous substitutions per synonymous site (Ka), the number of non-synonymous substitutions per non-synonymous site (Ks), and the P-value from a Fisher's exact test of neutrality were calculated using the Nei-Gojobori model with 1,000-bootstrap replicates [60]. A Ka/Ks ratio < 1 indicates purifying selection, a Ka/Ks ratio = 1 indicates neutral selection, and a Ka/Ks ratio > 1 indicates positive selection.

### Multiple sequence alignment and phylogenetic analysis of CrAQP family proteins

A bootstrap neighbor-joining phylogenetic tree was constructed based on multiple alignments of the identified AQPs from *C. rosea* with AQPs from Arabidopsis and soybean using MEGA 6.0 with 1,000 bootstraps. The sequences of GmAQPs (from soybean; *Glycine max*) and AtAQPs (from *Arabidopsis*; *Arabidopsis thaliana*) were downloaded from the phytozome database (https://phytozome-next.jgi.doe.gov/). Aquaporins were mapped on *C. rosea* chromosomes according to positional information of the *CrAQP* genes in the *C. rosea* genome database and displayed using MapInspect software (http://mapinspect.apponic.com/).

The gene structure for each *CrAQP* was illustrated using the Gene Structure Display Server 2.0 (http://gsds.cbi.pku.edu.cn/). To identify the biochemical features of all CrAQPs, the ProtParam (http://web.expasy.org/protparam/) was used to predict molecular weights (MW) and isoelectric points (pI) of the *candidate* CrAQP proteins. The transmembrane domains (TMDs), NPA motifs, and other conserved amino acid residues were recognized by the sequence alignment of CrAQPs with AtAQPs [26] and GmAQPs [34]. The numbers of phosphorylation sites within CrAQPs were predicted using NetPhos 3.1 (http://www.cbs.dtu.dk/services/NetPhos/). Subcellular localization was predicted using the WoLF PSORT server (https://wolfpsort.hgc.jp/) and Plant-mPLoc (http://www.csbio.sjtu.edu.cn/bioinf/plant-multi/). Conserved CrAQP motifs were analyzed using MEME suite (http://meme-suite.org/), with the maximum number of motifs being 10 and the optimum width of motifs ranging from 11 to 50.

### Promoter sequence profiling of *CrAQP*s

Putative *CrAQP* promoter sequences (2,000 bp upstream of ATG) were retrieved from the *C. rosea* genomes database (Table S1). Sequences were then uploaded into the PlantCARE database (http://bioinformatics.psb.ugent.be/webtools/plantcare/html/) for *cis-*acting regulatory element analysis. The *cis*-acting elements were classified as either hormone-specific (gibberellin-responsive elements, MeJA-responsive elements, auxin-responsive elements, salicylic acid-responsive elements, EREs, and ABREs) or abiotic stress-responsive (light responsive elements, MYCs, MYBs, MBSs, TC-rich repeats, and LTREs). The different elements were summarized and several selected *CrPIP* promoters were visualized using TBtools [61].

### Expression analysis of *CrAQP*s

A transcriptome database of *C. rosea* was constructed using Illumina HiSeq X sequencing technology. The quality of the RNA-Seq datasets created from seven different tissues (roots, vines, young leaves, flowers, and young siliques collected from *C. rosea* growing in SCBG; mature leaves from *C. rosea* growing in SCBG and on YX Island) was examined using FastQC (http://www.bioinformatics.babraham.ac.uk/projects/fastqc/), which produced 40 Gb clean reads. In brief, five different tissue samples were collected from similar young *C. rosea* plants (two-year-old with flowers and young siliques planted in SCBG), then these samples were cleaned and frozen quickly with liquid nitrogen for organ-specific RNA-Seq analysis. As to the habitat-specific RNA-Seq analysis, the mature leaves gathered separately from *C. rosea* plants growing in SCBG (seven-year-old plants) or native plants in YX Island, then the mature leaf samples were frozen with liquid nitrogen for future use. The samples mentioned above were collected with 3 independent replicates for each group. Clean reads were mapped to the *C. rosea* reference genome using Tophat v.2.0.10 (http://tophat.cbcb.umd.edu/). Gene expression levels were calculated as fragments per kilobase of transcript per million mapped reads (FPKM) according to the length of the gene and the read counts mapped to the gene: FPKM = total exon fragments/[mapped reads (millions) × exon length (kb)]. Expression levels (log2) of *CrAQPs* were visualised as clustered heatmaps using TBtools.

To investigate the involvement of the CrAQP genes in abscisic acid (ABA) and in various stress responses, *C. rosea* was germinated from seed and 30-day-old seedlings were exposed to stressors. In brief, for the high osmotic stress treatment, seedlings were removed from their pots and carefully washed with distilled water to remove soil from the roots, and then transferred into a 300 mM mannitol solution. For high salt stress, seedlings were soaked in a 600 mM NaCl solution. For alkaline stress, seedlings were soaked in a 150 mM NaHCO_3_ (pH 8.2) solution. For ABA treatment, a freshly prepared working solution of 100 μM exogenous ABA was sprayed on the leaves of seedlings. The second and/or third mature leaves from the seedling apexes were collected at 0, 2, and 24 h during the previously described stress treatments, with the 0-hourtime point used as the control. All samples were immediately frozen in liquid nitrogen and stored at − 80 °C for subsequent gene expression analysis. Three independent biological replicates were used. Transcript abundance of several *CrAQP*s' transcript was investigated using a qRT-PCR assay. The extraction and isolation of RNA and the synthesis of first strand cDNAs was performed as the previous report [36], with 3 replicates for each group. In brief, total RNA was extracted from *C. rosea* seedling tissues under the stress/ABA treatments and reverse transcribed to cDNA. Quantitative RT-PCR was conducted using the LightCycler480 system (Roche, Basel, Switzerland) and TransStart Tip Green qPCR SuperMix (TransGen Biotech, Beijing, China). All of the gene expression data obtained via qRT-PCR was normalized to the expression of CrEF-α (Table S2). The primers used for qRT-PCR (CrEF-αRTF/CrEF-αRTR for the reference gene and other CrAQP-specific primer pairs) are listed in Table S2.

### Detection of *CrPIP1;5* and *CrPIP2;3* homodimers and heterodimers using a yeast two-hybrid assay

The full-length cDNAs of *CrPIP1;5* (GenBank accession number MT787665) and *CrPIP2;3* (GenBank accession number MT787666) were isolated from the cDNA library of *C. rosea* seedlings, in which all cDNAs were inserted into a yeast expression vector (pYES-DEST52) using Gateway® techniques (Life Technologies). The recombinant plasmids containing *CrPIP1;5* and *CrPIP2;3* cDNAs were designated as CrPIP1;5-pYES-DEST52 and CrPIP2;3-pYES-DEST52 and used as template DNA in the following PCR assays. The open reading frame (ORF) regions of *CrPIP1;5* and *CrPIP2;3* were PCR-amplified using the primer pairs PIP1-5BDF/PIP1-5BDR and PIP2-3BDF/PIP2-3BDR, respectively (Table S2), and then inserted into a pGBKT7 vector using InFusion® techniques (In-Fusion HD® Cloning System, Clontech) to construct the pGBKT7-CrPIP1;5 and pGBKT7-CrPIP2;3 bait plasmids. The prey plasmid, pGADT7-CrPIP1;5, and pGADT7-CrPIP2;3, were generated by cloning the *CrPIP1;5* and *CrPIP2;3* ORFs into the pGADT7 vector after amplification (using the primers PIP1-5ADF/PIP1-5ADR, PIP2-3ADF/PIP2-3ADR as above; Table S2). Constructed vectors including activation domain (AD) and binding domain (BD) were co-transformed into AH109-competent yeast cells in pairs, and transformants were plated on SD/-Leu-Trp and SD/-Leu-Trp-His mediums to test protein interactions. Transformant spots on SD/-Leu-Trp-His medium were also supplemented with 40 µg/mL 5-bromo-4-chloro-3-indoxyl-α-d-galactopyranoside (X-α-Gal, 2 µL per spot) to further confirm interactions of the different co-transformants. Each experiment was independently repeated three times.

### In vivo stress tolerance assay for *CrPIP1;5* overexpression in yeast

The recombinant plasmids CrPIP1;5-pYES-DEST52 and the empty vector pYES2 (a negative control) were transformed into the *Saccharomyces cerevisiae* wild type strains W303 (MATa; *his3-11_15; leu2-3_112; ura3-1; trp1Δ2; ade2-1; can1-100*), WT (BY47471; MATa; *his3Δ1; leu2Δ0; met15Δ0; ura3Δ0*), and H_2_O_2_-sensitive mutant *skn7Δ* (BY4741; MATa; *ura3Δ0; leu2Δ0; his3Δ1; met15Δ0; YHR206w::kanMX4*). The yeast strain W303 was provided by Zhou et al. [62]. The WT (Y00000) and *skn7Δ* (Y02900) strains were obtained from Euroscarf (http://www.euroscarf.de/index.php?name=News). Plasmids were introduced into yeast strains using a standard polyethylene glycol (PEG)-lithium acetate-based transformation protocol. The yeast spot assay for NaCl, PEG, sorbitol, and H_2_O_2_ tolerance were performed as previously described [63].

### Functional identification of *CrPIP1;5* in transgenic Arabidopsis plants

The coding sequence (CDS) of the *CrPIP1;5* cDNA was PCR-amplified using the primer pair PIP1-5OXGF/PIP1-5OXGR (Table S2) and then inserted into plant expression vector pEGAD to generate *CrPIP1;5*-pEGAD. Thus, transgenic *Arabidopsis* plants (col-0 genotype, three overexpression lines, *OX 1#*, *OX 5#*, and *OX 10#*) were generated. After confirmation with genomic PCR and quantitative RT-PCR, these T3 homozygous transgenic lines were tested for their stress tolerance according to their seed germination rate as well as seedling and adult plant growth rate. These tests were thereby meant to evaluate the biological functions of *CrPIP1;5*.

In brief, seed germination rate of the *CrPIP1;5* transgenic *Arabidopsis* (*OX 1#*, *OX 5#*, *OX 10#*, and WT) was measured under the following stress treatments: NaCl (175 mM, 200 mM, and 225 mM; salt stress); 5 mmol/L NaHCO_3_ plus 95 mmol/L NaCl (pH 8.2), 7.5 mmol/L NaHCO_3_ plus 92.5 mmol/L NaCl (pH 8.2), and 10 mmol/L NaHCO_3_ plus 90 mmol/L NaCl (pH 8.2; alkaline stress); mannitol (200 mM, 300 mM, and 400 mM) stress. The goal of these treatments was to detect the effect of the overexpression of *CrPIP1;5* on affecting the salt/alkaline/osmotic tolerance of transgenic *Arabidopsis* seeds during germination. Additionally, root length was calculated to evaluate the influence of the overexpression of *CrPIP1;5* on transgenic *Arabidopsis* seedlings under abiotic stress (100 mM, 150 mM, and 200 mM NaCl for salt stress; 0.5 mmol/L NaHCO_3_ plus 99.5 mmol/L NaCl, 0.75 mmol/L NaHCO_3_ plus 99.25 mmol/L NaCl, 1 mmol/L NaHCO_3_ plus 99 mmol/L NaCl, pH 8.2 for alkaline stress; 200 mM, 300 mM, and 400 mM mannitol for osmotic stress. Wild-type *Arabidopsis* and Murashige&Skoog medium (MS) or MS plus 100 mM NaCl (pH 8.2) medium were used as controls. The seed germination and seedling growth experiments were both performed on MS plates with or without stress factors, in the same greenhouse environment used to grow the *Arabidopsis* plants. Drought and salt/alkaline tolerance assays were also performed on transgenic adult *Arabidopsis* plants. Both WT and transgenic seeds (*OX 1#*, *OX 5#*, and *OX 10#*) were grown on MS medium. Ten-day-old seedlings were transplanted into square pots filled with nutrient solution soaked vermiculite, with identical soil moisture. For drought tolerance assay, thirty to forty plants of each *OX* lines and WT control were cultured in growth chamber as described above without watering for another 20 days, since the original vermiculite humidity could ensure regular growth of Arabidopsis plants. The water content of vermiculite in the pots was reduced over this timeframe but did not induce drought stress. The plants were then subjected to a drought tolerance assay by continuous withdrawing water, whereby WT and transgenic plants (*OX 1#*, *OX 5#*, and *OX 10#*) turned to continuous drought conditions for 10 days and then rewatered adequately and recovered for another 7 days. Survival rates were then calculated according to the number of living plants at the end of the experiment. For salt tolerance assay, the ten-day-old seedlings of WT and transgenic Arabidopsis (*OX 1#*, *OX 5#*, and *OX 10#*) growing in square pots were irrigated with 200 mM NaCl solution (50 mL each pot) and the phenotype was recorded after 7 days. For alkaline tolerance assay, the same seedlings were irrigated with 100 mM NaHCO_3_ solution (50 mL each pot) and the phenotype was recorded after 4 days.

Subcellular localization of CrPIP1;5 in *Arabidopsis* was also detected using GFP fusion protein in seedling roots. The *OX* homozygous lines of CrPIP1;5-pEGAD and the control (pEGAD) transgenic plants were sterilized and spotted in MS plates to generate seedlings. Then, three- to four-day-old seedlings were detected using a camera fitted to a confocal laser scanning microscope to record the GFP fluorescence of different tissues. To confirm it was the cell membrane that was fluorescing, seedling roots were stained by using a propidium iodide solution (1 mg/mL in phosphate buffer solution).

### Statistical analysis

All the experiments in this study were repeated three times independently and results are shown as mean ± SD (n ≥ 3). Pairwise differences between means were analyzed using Student's *t*-tests in Microsoft Excel 2010.

## Supplementary Information

**Additional file 1: Figure S1**. The structural features of the CrAQPs. **a** CrNIPs. **b** CrPIPs. **c** CrTIPs. **d** CrXIPs. **e** CrSIPs.

**Additional file 2: Figure S2**. The overexpression analyses of *CrPIP1;5* in the three transgenic Arabidopsis lines (*OX 1#*, *OX 5#*, and *OX 10#*). **a** RT-PCR and genomic DNA PCR analysis of *CrPIP1;5* in the transgenic Arabidopsis lines and WT plants. **b** Quantitative RT-PCR analysis of *CrPIP1;5* in the transgenic Arabidopsis lines and WT plants.

**Additional file 3: Figure S3**. Overexpression analyses of *CrPIP1;5* in the transgenic Arabidopsis lines (*OX 1#*, *OX 5#*, and *OX 10#*) and stress tolerance analyses of transgenic plants with regards to seed germination rates. **a** Photographs of the transgenic lines and WT seeds germinated on MS medium or MS medium with NaCl, NaCl plus NaHCO_3_ (pH 8.2), or mannitol for 7 d. **b‒d** The seed germination rates in WT and transgenic lines under NaCl (**b**), NaCl plus NaHCO_3_ (pH 8.2) (**c**), and mannitol (**d**) stresses after 7 d.

**Additional file 4: Figure S4**. Salt, salt-alkaline, and high osmotic stress analyses of the transgenic plants with *CrPIP1;5*’s overexpression based on seedling root lengths. Four-day-old seedlings were transplanted into MS medium containing NaCl, NaCl plus NaHCO_3_ (pH 8.2) or mannitol and then grown for 7 d before measuring the root length. **a** Photographs of the transgenic lines (*CrPIP1;5OX 1#*, *OX 5#*, and *OX 10#*) and WT seedlings on MS medium or MS medium with NaCl, NaCl plus NaHCO_3_ (pH 8.2), or mannitol; **b‒d** The seedling root lengths (mm) in WT and the transgenic lines under NaCl (**b**), NaCl plus NaHCO_3_ (pH 8.2) (**c**), or mannitol (**d**) stresses after 7 d. Error bars indicate the SD based on over three replicates (*n* ≥ 3). Asterisks indicate significant differences from the control (Student’ s *t*-test P values, * *p* < 0.05 and ** *p* < 0.01).

**Additional file 5: Figure S5**. Salt and alkaline stress analyses of the transgenic plants with *CrPIP1;5*’s overexpression based on the growth of adult Arabidopsis. **a** Leaf phenotypes of the transgenic Arabidopsis *OX* lines and WT plants under 200 mM NaCl stress for 7 days. **b** Leaf phenotypes of the transgenic Arabidopsis *OX* lines and WT plants under 100 mM NaHCO_3_ (pH 8.2) stress for 4 days.

**Additional file 6: Figure S6**. Subcellular localization of the CrPIP1;5 protein. Arabidopsis roots expressing 35S:GFP-CrPIP1;5 fusion proteins (upper two lines) and 35S:GFP (lower line) were observed under a laser scanning confocal microscope.

**Additional file 7: Table S1**. The obtained *CrAQP*s’ nucleotide and protein sequences information in this study.

**Additional file 8: Table S2**. Primers’ information used in this study.

**Additional file 9: Table S3**. The categories of *cis*-acting elements identified in the *CrAQP*s’ promoter regions.

**Additional file 10: Table S4**. The conserved motif sequences of CrAQPs identified by the MEME web server.

## Data Availability

All data generated or analyzed during this study are included in this article and its supplementary information files. However, the sequence data in this study can also be accessed at http://doi.org/10.13140/RG.2.2.21213.74727.

## Abbreviations

AQP: Aquaporin
RNA-Seq: RNA sequencing
PIP: Plasma membrane intrinsic protein
NIP: Nod26-like intrinsic protein
TIP: Tonoplast intrinsic protein
SIP: Small and basic intrinsic protein
XIP: X-intrinsic protein
GIP: GlpF-like intrinsic protein
HIP: Hybrid intrinsic protein
MIP: Major intrinsic protein
NPA: Asparagine-proline-alanine
ar/R: Aromatic/Arginine region
FPs: Froger’s positions
GSDS: Gene Structure Dispaly Server
ABA: Abscisic acid
SDG: Synthetic dropout medium plus galactose
qRT-PCR: Quantitative Reverse Transcription Polymerase Chain Reaction
OX: Over-expression
WT: Wild type
GRAVY: Grand average of hydropathicity
MS: Murashige and Skoog medium
CK: Control check
SCBG: South China Botanical Garden
YX: Yongxing Island
MW: Molecular weight
pI: Isoelectric point
TMD: Transmembrane domain
FPKM: Fragments per kilobase of transcript per million mapped reads
AD: Activation domain
BD: Binding domain
CDS: Coding sequence
