# Supplementary figures and images for: Genome-wide identification and expression analysis of aquaporin family in Canavalia rosea and their roles in the adaptation to saline-alkaline soils and drought stress
Source: BMC Plant Biol. 2021 Jul 13;21:333. doi: 10.1186/s12870-021-03034-1 (PMC8278772; doi:10.1186/s12870-021-03034-1)

**Figure S1**


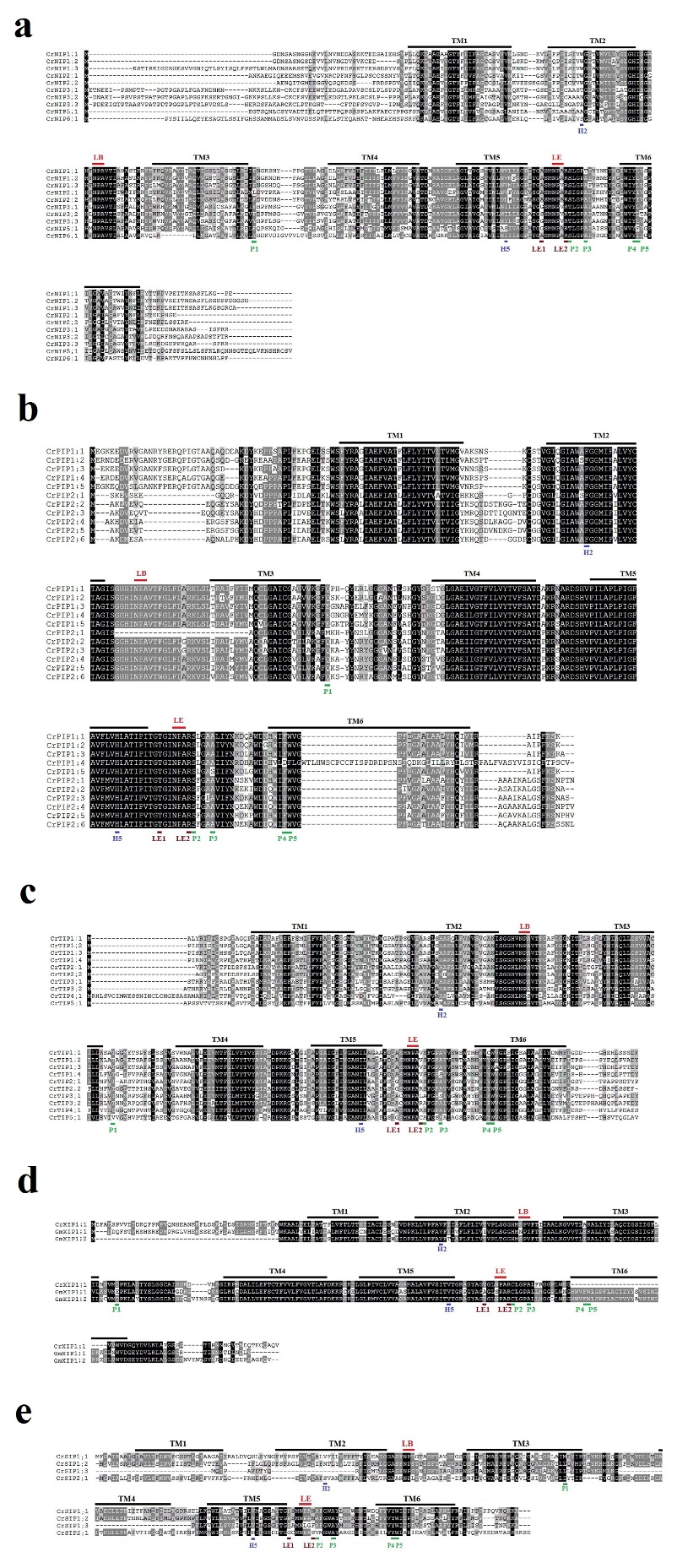

Supplement: Supplementary file 1 — Additional file 1: Figure S1. The structural features of the CrAQPs. a CrNIPs. b CrPIPs. c CrTIPs. d CrXIPs. e CrSIPs. [file 12870_2021_3034_MOESM1_ESM.docx]

Figure S2


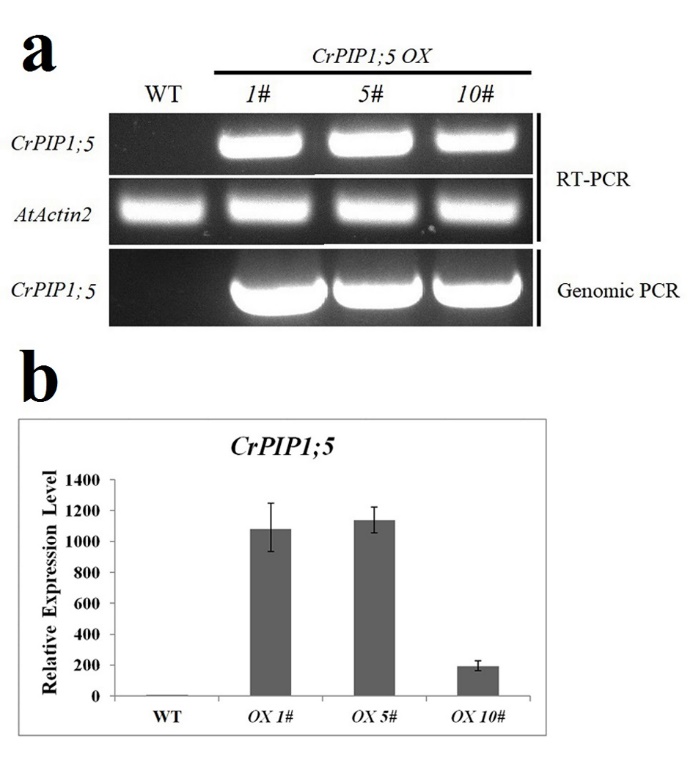

Supplement: Supplementary file 2 — Additional file 2: Figure S2. The overexpression analyses of CrPIP1;5 in the three transgenic Arabidopsis lines (OX 1#, OX 5#, and OX 10#). a RT-PCR and genomic DNA PCR analysis of CrPIP1;5 in the transgenic Arabidopsis lines and WT plants. b Quantitative RT-PCR analysis of CrPIP1;5 in the transgenic Arabidopsis lines and WT plants. [file 12870_2021_3034_MOESM2_ESM.docx]

Figure S3


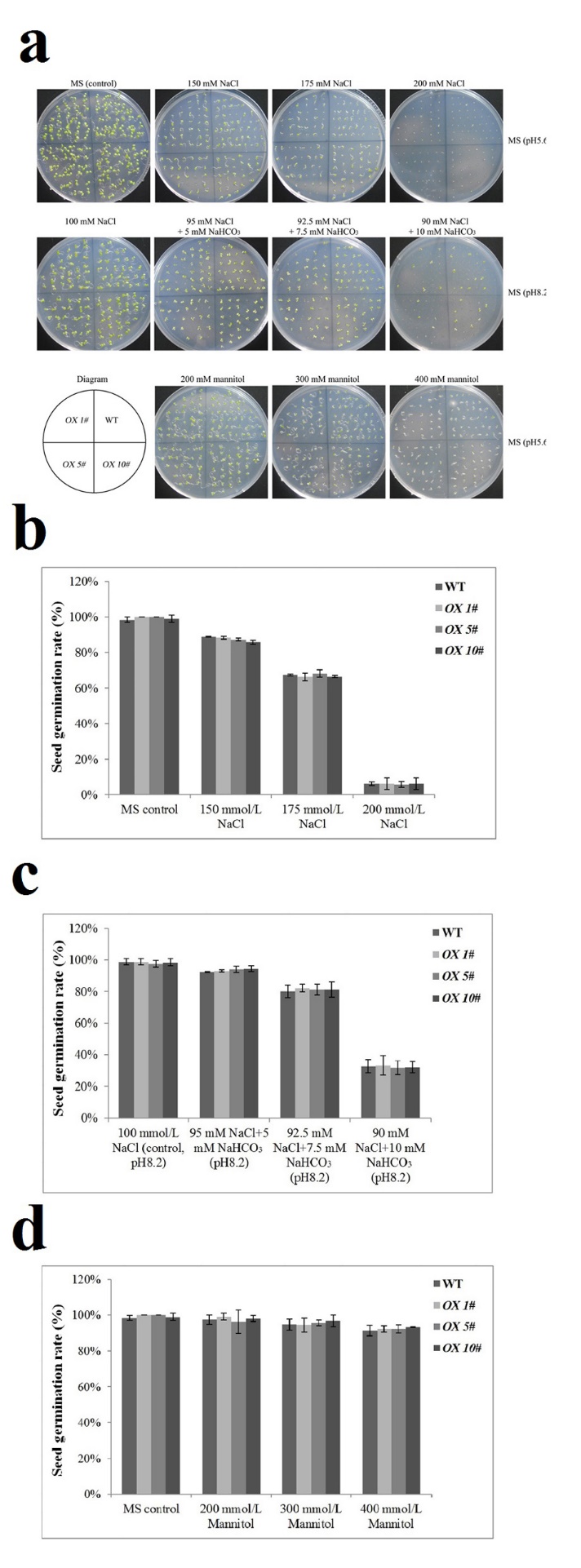

Supplement: Supplementary file 3 — Additional file 3: Figure S3. Overexpression analyses of CrPIP1;5 in the transgenic Arabidopsis lines (OX 1#, OX 5#, and OX 10#) and stress tolerance analyses of transgenic plants with regards to seed germination rates. a Photographs of the transgenic lines and WT seeds germinated on MS medium or MS medium with NaCl, NaCl plus NaHCO3 (pH 8.2), or mannitol for 7 d. b‒d The seed germination rates in WT and transgenic lines under NaCl (b), NaCl plus NaHCO3 (pH 8.2) (c), and mannitol (d) stresses after 7 d. [file 12870_2021_3034_MOESM3_ESM.docx]

Figure S4


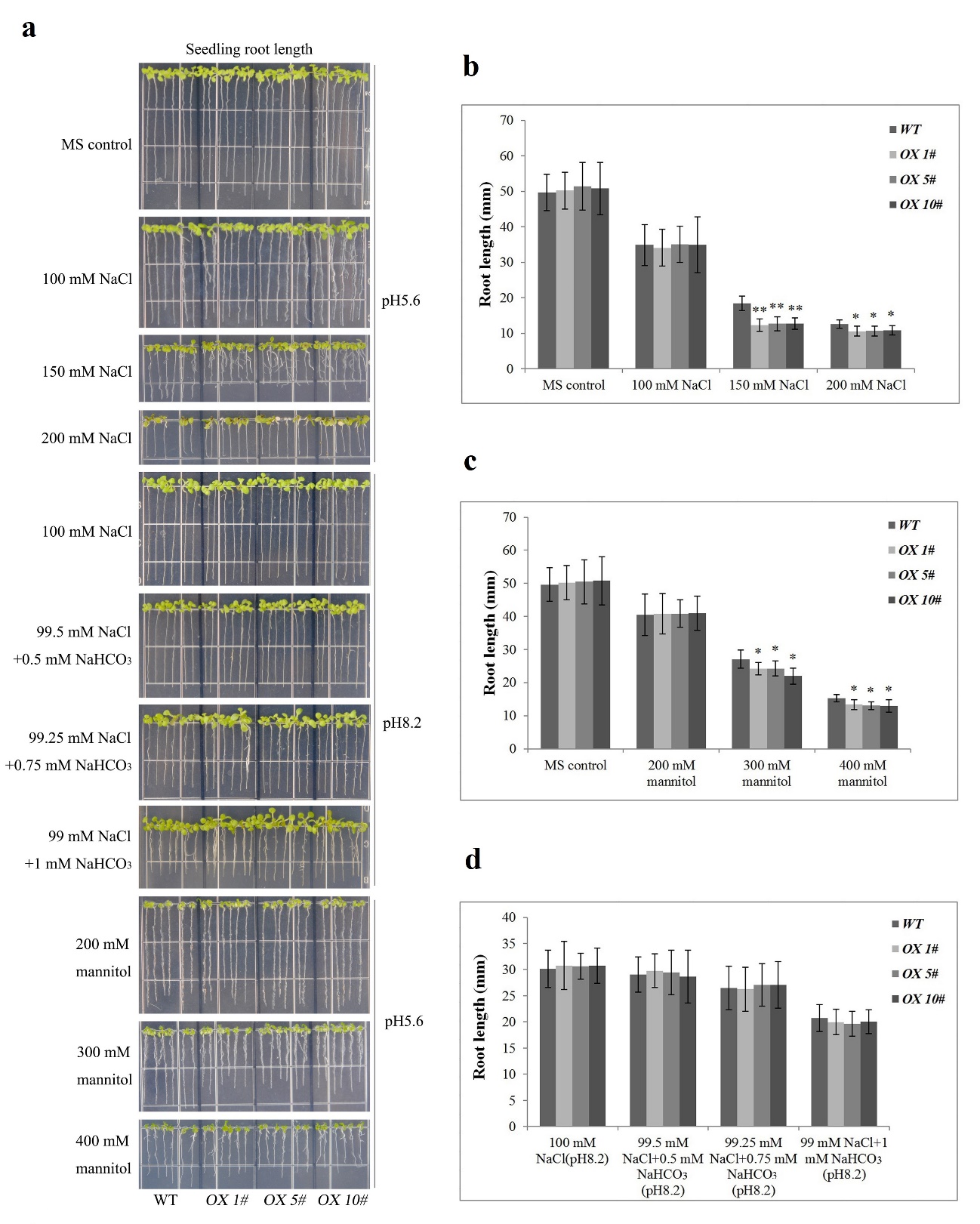

Supplement: Supplementary file 4 — Additional file 4: Figure S4. Salt, salt-alkaline, and high osmotic stress analyses of the transgenic plants with CrPIP1;5’s overexpression based on seedling root lengths. Four-day-old seedlings were transplanted into MS medium containing NaCl, NaCl plus NaHCO3 (pH 8.2) or mannitol and then grown for 7 d before measuring the root length. a Photographs of the transgenic lines (CrPIP1;5OX 1#, OX 5#, and OX 10#) and WT seedlings on MS medium or MS medium with NaCl, NaCl plus NaHCO3 (pH 8.2), or mannitol; b‒d The seedling root lengths (mm) in WT and the transgenic lines under NaCl (b), NaCl plus NaHCO3 (pH 8.2) (c), or mannitol (d) stresses after 7 d. Error bars indicate the SD based on over three replicates (n ≥ 3). Asterisks indicate significant differences from the control (Student’ s t-test P values, * p < 0.05 and ** p < 0.01). [file 12870_2021_3034_MOESM4_ESM.docx]

Figure S5


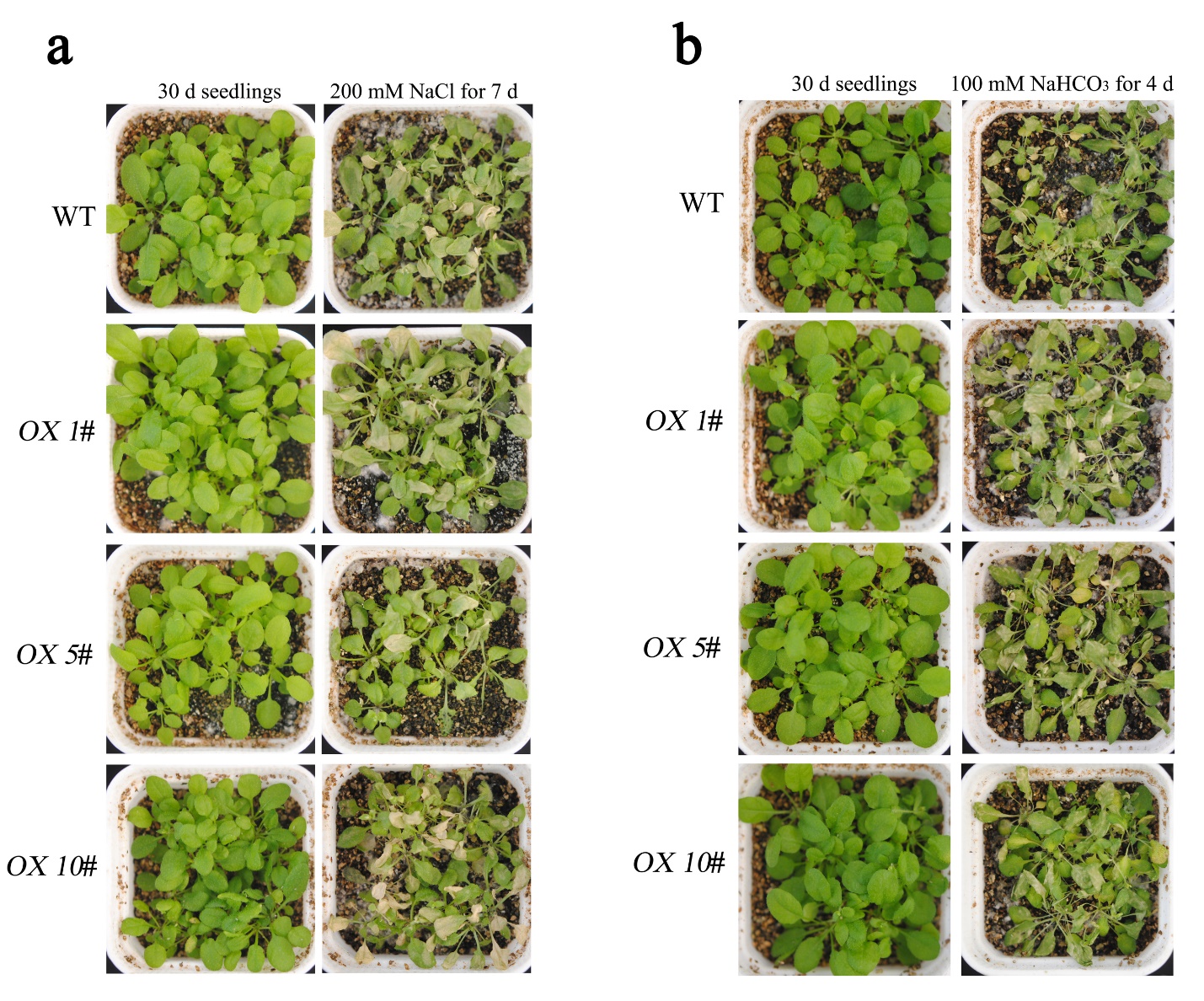

Supplement: Supplementary file 5 — Additional file 5: Figure S5. Salt and alkaline stress analyses of the transgenic plants with CrPIP1;5’s overexpression based on the growth of adult Arabidopsis. a Leaf phenotypes of the transgenic Arabidopsis OX lines and WT plants under 200 mM NaCl stress for 7 days. b Leaf phenotypes of the transgenic Arabidopsis OX lines and WT plants under 100 mM NaHCO3 (pH 8.2) stress for 4 days. [file 12870_2021_3034_MOESM5_ESM.docx]

Figure S6


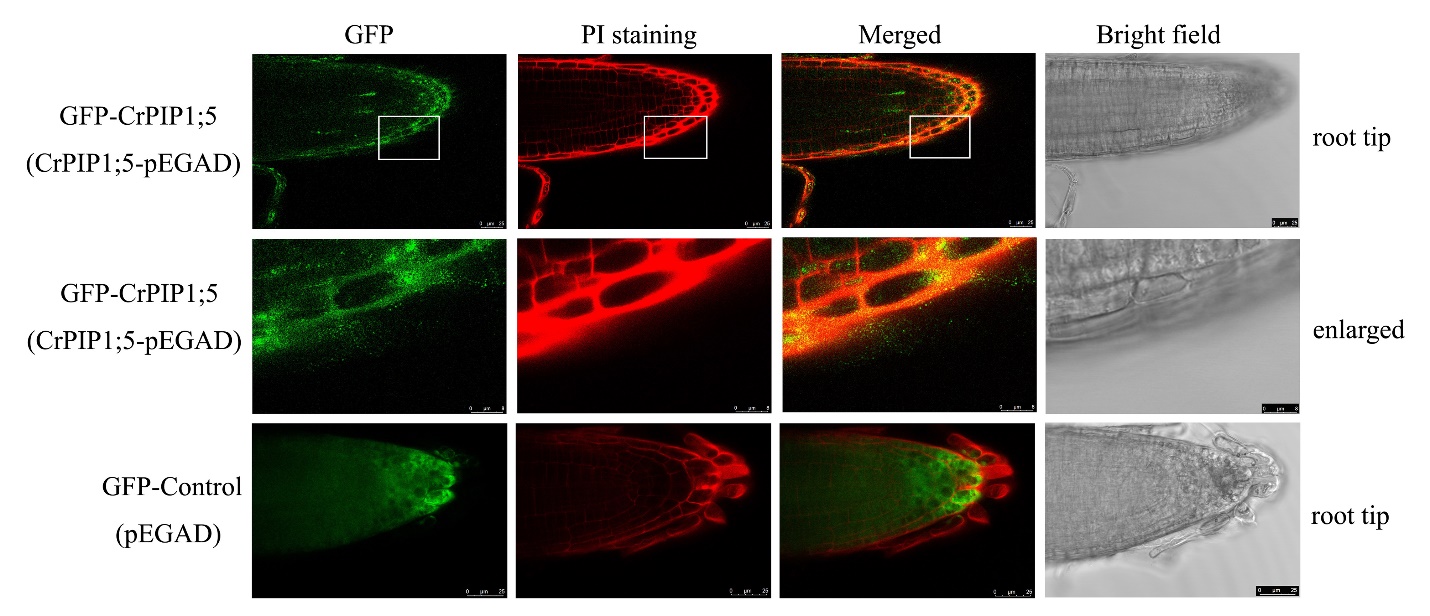

Supplement: Supplementary file 6 — Additional file 6: Figure S6. Subcellular localization of the CrPIP1;5 protein. Arabidopsis roots expressing 35S:GFP-CrPIP1;5 fusion proteins (upper two lines) and 35S:GFP (lower line) were observed under a laser scanning confocal microscope. [file 12870_2021_3034_MOESM6_ESM.docx]
