# Supplementary material for: Genome-wide identification and expression analysis of aquaporin family in Canavalia rosea and their roles in the adaptation to saline-alkaline soils and drought stress
Source: BMC Plant Biol. 2021 Jul 13;21:333. doi: 10.1186/s12870-021-03034-1 (PMC8278772; doi:10.1186/s12870-021-03034-1)
Supplement: Supplementary file 7 — Additional file 7: Table S1. The obtained CrAQPs’ nucleotide and protein sequences information in this study. [file 12870_2021_3034_MOESM7_ESM.docx]

**Table S1**

| **The sequence information of the CrAQP family** |
| --- |
| **Protein** |
| >CrPIP1;1  MEGKEEDVRVGANRYRERQPIGTAAQAQDDAKDYKEPPSAPLFEPGELSSWSFYRAGIAEFVATFLFLYITVLTVMGVAKSNSKCSTVGIQGIAWAFGGMIFALVYCTAGISGGHINPAVTFGLFLARKLSLTRAIFYIIMQCLGAICGAGVVKGFQPHQYERLGGGANTLSKGYSKSTGLGAEIVGTFVLVYTVFSATDAKRNARDSHVPILAPLPIGFAVFLVHLATIPITGTGINPARSLGAALIYNKDQAWDNHWIFWVGPFIGAALAALYHQIVIRAIPFRSK |
| >CrPIP1;2  MERNDEDERVGANRYGERQPIGTGAQSQDGKEYREAAPAPLFEARELTSWSFYRAGIAEFVATFLFLYITVLTLMGVAKSPTKCSTVGVQGIAWSFGGMIFALVYCTAGISGGHINPAVTFGLFLARKLSLTRTVFYMIMQCLGAICGAAVVKGFQSKQYERLGGAANTLNKGYSKGDGLGAEIVGTFILVYTVFSATDAKRNARDSHVPILAPLPIGFAVFLVHLATIPITGTGINPARSLGAAIVYNKDQAWDGHWIFWVGPFIGAALAALYHQIVMRAIPFKSK |
| >CrPIP1;3  MEKEEDVKVGANKFSERQPLGTAAQSDKDYKEPPAAPLFEPGELKSWSFYRAGIAEFVATFLFLYITILTVMGVNNSSSKCSSVGIQGIAWAFGGMIFALVYCTAGISGGHINPAVTFGLFLARKLSLTRAVFYIVMQCLGAICGAGVVKGFEGNARYELFKGGANFVNPGYTKGDGLGAEIVGTFILVYTVFSATDAKRNARDSHVPILAPLPIGFAVFLVHLATIPITGTGINPARSLGAAIIFNRDLAWDDHWIFWVGPFVGAALAALYHQIVIRAIPFKTRA |
| >CrPIP1;4  MERDEDVKVGANKYSERQALGTGAQGEKDYKEAPPAPLFEPGELKSWSFYRAGIAEFVATFLFLYITVLTVMGVNRSPSKCSSVGIQGIAWAFGGMIFALVYCTAGISGGHINPAVTFGLFLARKLSLTRAVFYIVMQCLGAICGAGVVKGFEGNGRYEMYKGGANFVSHGYTKGDGLGAEILGTFILVYTVFSATDAKRNARDSHVPILAPLPIGFAVFLVHLATIPITGTGINPARSLGAAIIYNRDHAWDDHVLDFLGWTLHWSCPCCFISPDRDPSNSFQDKGLILLRYELSTRPALFVASYVISICFTPSCV |
| >CrPIP1;5  MEGKEQDVSLGANKFPERQPIGTAAQSQDDGKDYQEPPPAPLFEPSELTSWSFYRAGIAEFVATFLFLYITILTVMGVNRSPKCQSVGIQGIAWAFGGMIFALVYCTAGISGGHINPAVTFGLFLARKLSLTRALFYMVMQVLGAICGAGVVKGFEGKTRYGLYKGGANFVAPGYTKGDGLGAEIVGTFILVYTVFSATDAKRSARDSHVPILAPLPIGFAVFLVHLATIPITGTGINPARSLGASIIFNKDLGWDDHWIFWVGPFIGAALAALYHQVVIRAIPFKSK |
| >CrPIP2;1  MSKEVSEEGQQRKDYVDPPPAPLIDLAEIKLWSFYRALIAEFIATLLFLYVTVATVIGHKKQSGPCDGVGLLGIAWSFGGMIFVLVYCTAGISAQCLGAICGVGLVKAFMKHPYNSLGGGANSVSSGYNKGTALGAEIIGTFVLVYTVFSATDPKRNARDSHVPVLAPLPIGFAVFMVHLATIPITGTGINPARSFGAAVIYNNSKVWDDHWIFWVGPFVGALAAAAYHQYILRAAAIKALGSFRSNPTN |
| >CrPIP2;2  MAKEVEVQEQGEYSAKDYQDPPPTPLFDPEELTKWSFYRALIAEFIATLLFLYVTVLTIIGYKSQTDSTKGGTECDGVGILGIAWAFGGMIFILVYCTAGISGGHINPAVTFGLFLGRKVSLIRALLYMVAQCAGAICGTGLAKGFQKAYYNRYGGGANSVADGYNKGTALGAEIIGTFVLVYTVFSATDPKRNARDSHVPVLAPLPIGFAVFMVHLATIPITGTGINPARSFGAAVIYNKEKIWDDQWIFWVGPIVGAAVAAIYHQYILRASAIKALGSFRSNA |
| >CrPIP2;3  MAKDVEQVTEQQGEYSAKDYHDPPPAPLIDVEELTKWSLYRAAIAEFIATLLFLYITVLTIIGYMRQSDTTIQGNTECDGVGILGIAWAFGGMIFILVYCTAGISGGHINPAVTFGLFVGRKVSLIRALLYMIAQCAGAICGAGLAKGFQKAYYNRYGGGVNLVSDGYNKGTALGAEIIGTFVLVYTVFSATDPKRSARDSHVPVLAPLPIGFAVFMVHLATIPVTGTGINPARSFGPAVIFNKDKAWDDQWIYWVGPFVGAAVAAFYHQYILRAAAIKALGSFRSNAS |
| >CrPIP2;4  MAKHDVEIAERGSFSAKDYHDPPPAPLIDAEELTKWSFYRALIAEFIATLLFLYITVLTVIGYKSQSDLKAGGDVCGGVGILGIAWAFGGMIFILVYCTAGISGGHINPAVTFGLFLARKVSLIRAIMYMVAQCLGAICGVGLVKAFQKAYYNRYGGGANELSDGYSTGVGLGAEIIGTFVLVYTVFSATDPKRSARDSHVPVLAPLPIGFAVFMVHLATIPVTGTGINPARSLGAAVIYNQEKAWDDQWIFWVGPFAGAAIAAFYHQFILRAGAAKALGSFRSNPTV |
| >CrPIP2;5  MAKDVEVTERGSFSGKDYHDPPPAPLIDAEELTKWSFYRALIAEFIATLLFLYITVLTVIGYKHQSDVNDKGDVCGGVGILGIAWAFGGMIFILVYCTAGISGGHINPAVTFGLFLARKVSLIRAIMYIIAQCLGAICGVGLVKAFQKSYYNRYGGGANSLADGYSTGVGLGAEIIGTFVLVYTVFSATDPKRSARDSHVPVLAPLPIGFAVFMVHLATIPVTGTGINPARSFGAAVIYNHDKPWDDHWIFWVGPFAGAAIAAFYHQFILRAGAVKALGSFRSNPHV |
| >CrPIP2;6  MAKDMESAAQNALPHKDYHDPPPAPLFDAAELRSWSLYRALIAEFVATLLFLYVTVLTVIGYKHQTDGGDPCNGVGILGIAWAFGGMIFVLVYCTAGISGGHINPAVTWGLFLARKVSLVRALSYMVVQCLGAICGVGLVKAFQKSYYNRYAGGANMLSDGYNKGTGLGAEIIGTFLLVYTVFSATDPKRNARDSHVPVLAPLPIGFAVFMVHLATIPITGTGINPARSFGAAVIYNNEKAWDDQWIFWVGPFIGATIAAIYHQFVLRAQAAKALGSFRSSSNL |
| >CrTIP1;1  MALYRIAIGSPGEAGQPDALRAAFAEFFSMIIFVFAGEGSGMAYNKLTNNGPATPSGVIAASLSHAFGLFVAVSVGANISGGHVNPAVTFGAFLGGNITLLRSILYWIAQLLGSVVACILLKSATGGMETSPFSLSSGVSVWNALVLEIVMTFGLVYTVYATALDPKKGNVGIIAPIAIGFIVGANILAGAAFDGASMNPAVSFGPAVVSWSWTHHWVCWVGPFIGSAIAAIIYDNMFIGDDGHEHLSSSDF |
| >CrTIP1;2  MPISRISIGNPSELGQADALKAALAEFISMLIFVFAGEGSGMAYNKLTNNGSATPAGVVAASLSHAFALFVAVSVGANISGGHVNPAVTFGAFIGGHITLLRGILYWIAQLLGSVVACLLLKLATAGMETSAFSLSSGVGAANALVFEIVMTFGLVYTVYATAVDPKKGNIGIIAPIAIGFIVGANILAGGAFDGASMNPAVSFGPAVVSWTWANHWVYWVGPLIGSAIAALVYEIFFITPSSYEQLPVADY |
| >CrTIP1;3  MPISKIAIGHSSELTQADALKAALAEFISMLIFVFAGEGSGMAYNKLTKNGSATPAGLVAASLSHAFALFVAVSVGANISGGHVNPAVTFGAFIGGHITLFRTILYWIAQLLGSIVACLLLKVATGGLETSAFALSSGVGAGNAFVFEIVMTFGLVYTVYATAVDPKKGDLGIIAPIAIGFIVGANILAGGAFDGASMNPAVSFGPAVVSWTWDNHWVYWAGPFIGSAIAALVYEIFFINQNTHDHLPTTDY |
| >CrTIP1;4  MPIRNIAVGRPEEATHPDTLKAALAEFISTLIFVFAGSGSGIAYNKLTDNGAATPAGLISAAIAHAFALFVAVSVGANISGGHVNPAVTFGAFIGGNISFLRGIVYIIAQLLGSIVASLLLLFVTGLPVPAFGLSAGVGVGNALVLEIVMTFGLVYTVYATAIDPKKGNLGIIAPIAIGFIVGANILLGGAFDGASMNPAVSFGPSVVSWSWNNHWIYWVGPLIGGGLAGLIYEVIFISHTHEQLPTTDY |
| >CrTIP2;1  MVKIALGTFDDSFSIASLKAYLAEFIATLLFVFAGVGSAIAYNELTSDAALDAPGLVAVAVAHAFALFVGVAIAANISGGHLNPAVTFGLAIGGNITLLTGFLYWIAQLLGSIVACLLLNFVTAKSVPTHGVAAGVNVFAGLVFEIVVTFGLVYTVYATAADPKKGSLGIIAPIAIGFIVGANILAAGPFSGGSMNPARSFGPAVVSGNFVDNWIYWIGPLIGGGLAGLIYGDIFIGSYTPAPPSDTYP |
| >CrTIP2;2  MAGIAFGRFDDSFSLSSIKAYIAEFISTLLFVFAGVGSAIAYGKLTSDAALDPAGLVAVAICHGFALFVAVSVGANISGGHVNPAVTFGLALGGQITILTGIFYWIAQLVGSIVACFLLHFVTGGLTTPIHSVAAGVGAVEGVVTEIIITFGLVYTVYATAADPKKGSLGTIAPIAIGFIVGANILAAGPFSGGSMNPARSFGPAVVSGNFHDNWIYWVGPLIGGGLAGLIYGNVFIRSDHAPLSSEF |
| >CrTIP3;1  MSTRRYAFGRADEATHPDSMRATLAEFASTFIFVFAGEGSGLALVKIYQDSAFSAGELLAVALAHAFALFAAVSSSMHVSGGHVNPAVTFGALLGGRISVVLAIYYWIAQLLGAIVAALILRLVTNNMRPSGFHLAPGVGAAHMLILEIVMTFGLMYTIYGTAIDPKRGSVSNIAPLAIGLIVGANILVGGPFDGACMNPALAFGPSLVGWRWHYHWIFWVGPLIGAALAAIIYEYVVIPTEPPHQHQPLAPEDY |
| >CrTIP3;2  MATNRRYAIGRLDEANNPDSMRATLAEFLSTCIFVFVGEGSALALSQIYKDTGTSAGELVVVALAHAFALFAAISATMHVSGGHVNPAVTFGSLLAGKISVLRAVYYWVAQLLGSVVAALLLRLVTNNMRPQGFGVSVGVGAFHSLILEIALTFGLMYTVYATALDPKRGTVGSISPLAIGLVVGANILVGGPFDGACMNPARAFGPALVGWRWHYHWIFWVGPLIGAALAALLYEYVMVQTEPPHAHHQPLAPEDY |
| >CrTIP4;1  MRHLSVCIMWESSNIHCLCNGESASAMAKIALGTTREVTQPDCIQALVVEFIATFLFVFVGVGASMAVDKFVGDALVGLFAVAVAHALVVAVMISSAHISGGHLNPSVTLGLLASGHITLFRSILYWIDQLIASAAASFLLYYLSGGQNTPVHTLASGVGYGQGVIWEIVLTFGLLFTVYATMVDPKKGALAGIGPTLVGFVVGANILAGGAFSAASMNPARSFGPALVSGNWTDHWVYWVGPLIGGGLAGFIYETFFIDRSHVLLFPDAES |
| >CrTIP5;1  MAPSSVTVTSRFHESVTRNALRSYLSEFVCTFFFVFLVVGSQMSSRKLMPDASLNPTSLVVVAMANAFALCSVLYVAWDISGGHVNPAVTFAMAVGGHISVPTALFYWVAQLIASVMACLVLRVIVVGMHVPTYTIAEEMTGFGASVLEGILTFVLVYTVYAARDPRRGPMSSTGPLAVGLIAGANVLAAGPFSGGSMNPACAFGSAAIAGSFRNQAVYWVGPLIGASIAGLLYDNALFPSHTTHSVTQGLAV |
| >CrNIP1;1  MGDNSASNGGHEVVLNVNDDASKKTEDSAIEHSVPLLQKLAAEAAGTYFLIFAGCASVVVNLGNDKVLTFPGISIVWGLTVMVLVYSLGHISGAHFNPAVTIAHASTKRFPFKQVPAYIIAQVIGSLLASGTLRLIFNGKSNYFPGTVPAGSDLQAFVIEFIITLFLMLVISGVATDNRAIGELAGLVVGSTVLLNVLIAGPITGASMNPARSLGPTIVYNEYRGIWIYLVSPILGAVAGTWIYNFLRYTTKPVPEITKSASFLKGPE |
| >CrNIP1;2  MGDNSASNGSDEVVLNVNGDVSVKCEDSVPLLQKLVAEVVGTYFLIFAGCGSVVVNLSKDKMVTQPGISIVWGLTVMVLAYSLGHISGAHFNPAVTIAHASTKRFPFKQVPGYIVAQVVGSTLASGSLRLIFNGKNDHFAGTLPAGSDLQAFVLEFIITFYLMFVISGVATDNRAIGELAGLAIGSTVLLNVMFAGPITGASMNPARSLGPAIVHNEYRGLWIYLVSPTVGAVAGTWAYNFIRYTNKPVREITNSASFLKGSPPEGGSH |
| >CrNIP1;3  MKSTIRKIGDRGESVVGNIQTLSYISQLFPSTLWLMADNSARSETQEVVLNVPKDPSKTYERSDSYVSVPFLQKLVAEIVGTYFLIFAGCASVVVNKNNDNVVTLPGISIVWGLAVMVLVYSVGHISGAHFNPAVTIAFASTRRFPLMQVPAYVAAQLLGATLASGTLKLIFNGSHDQFSGTLPAGSNLQAFVIEFIITFYLMFVISGVATDNRAIGELAGLAIGSTILLNVMIAGPITGASMNPVRSLGPAFVHTEYRGIWIYLLSPVVGAVAGAWVYNIIRYTDKPLREITKSASFLKGSGRCA |
| >CrNIP2;1  MANKAEGIQEEEMSRVEVGVNRCPFNFSGLPSCCSSNYVVTLTQKVIAEFIGTYFVVFAGCGSVAVNKIYGSVTFPGICITWGLIVMVMVYSVGHISGGHFNPAVTITWAIFRRFSYKEVPIYIVAQLLGSILASGTLALMLDVTPKAYFGTVPVGSIGQSLAAEIIITFLLMFVISAVSTDDRAVGDFAGVTVGMTIMLNVFVAGPVSGASMNPARSIGPALIKHVYKGLWVYIVGPIIGAIAGAFVYNFLRSTEEPHSE |
| >CrNIP2;2  MAENQITGMEEGGVQSQKDSSFRDSPDVVQIIQKVVAEVIGTYFFIFAGCCSLVLNKVEESKGSITFPGICVVWGVTLMILVYSLGHISGAHFNPAVTVSLAIYRQFPLKQVPLYIIAQIVGSVLASGTLDLLFDVDDNSYFGTVPTGSYTRSLVFEILTTFLLLFVISSVTTDNGAVGELAGVAIGMTILIDLFIAGNVSGASMNPARSLGPALVMHIYTGFWIYIVGPFLGGILGVTAYNLIRFNEKPLSSIRK |
| >CrNIP3;1  METNEEIPSMPTTPGTPGAPLFGAFNDNHNNKKSLLKNCKCFSVEEWTIEDGALPAVSCSLPLSPPPVSLARKVGAEFIGTFILMFSGTAAAIVNQKTPGSETLIGCAASTGLAVMIVILATGHISGAHLNPAVTISFAALKHFPWKHVPMYIGAQVLASICSAFALKGIFHPFMSGGVTVPSGGYGQAFALEFIIGFNLMFVVTAVATDTRAVGELAGIAVGATVMLNILIAGPATGGSMNPVRTLGPAIAANNYKAIWVYLVAPVLGALGGAGTYTAVKLPEEDDNAKARASISFRR |
| >CrNIP3;2  MDNAEIPSVPSTPATPGTPGAPLFGGFKSERTGNGIGKKPSLLKSCKCFSVEEWTLEDGTLPKLSCSLPPPPVPLAKKVGAEFIGTFILMFAAIGTAIVNQKTHGSETLVGCAAANGLAVMIIISSTGHICGAHLNPAVTISFAALKHFPWKNVPVYIGTQLLASICAAFALKEVFDPFMSGGVTVPSVGYGQAFAIEFTVSFILMFVVTAVATDTRAVGELAGIAVGATVMLNILIAGPATGSSMNPVRTLGPAIATNNYRGIWVYLTAPILGTLCGAGAYTVVKLPDQRFNSQAKAPSAPSTFTR |
| >CrNIP3;3  MPDEEIGTPTAASVPATPDTPGGPLFTSLRVDSLSHERDSFAKARCKCLPTKGHTCFTDFSVGVPIPNVSLTQKIGAEFVGTFILIFASTAGPIVNNKYNGAEGLLGNGATAGLTVMFIILSIGHISGAHLNPALTIAFAAFRHFPWVHVPAYVAAQVSASICAGFALKAVYHPFLSGGVTIPSVTIGQAFATEFIITFNLLFVVTAVATDTRAVGELAGIAVGATVLLNILISGPTSGGSMNPVRTLGPAVAAGNFKHIWIYLVAPTLGGLAGAGVYTLVKLRDKDGEPPRQARSFRR |
| >CrNIP4;1  MAEVVGTFILMFCVCGINASTQFQNGAVGLLEYAATAGLTVIVIIFSIGPISCAHVNPAVTIAFATIGQFPWFKVPVYIIAQTVGSMSATYIGSLVYGIKSDVMMTQPLQGCNSAFWVEVIATFIIMFLIAALTFESQSVGHLSGLVAGIAIGLAVLITGPVSGGSMNPARSLGPAIVSWKFKDIWIYILAPCVGAVAGALMFHVLRLREQHCSPLSSQNIRDVGRPIPLCSKYNDKSTFTQIVVASSIGLILAATMHYNVKRMRDRKIVPRLRFSKTRQIPKLEKFSHYVARQMGFKDKKSCPLLCKLASEYIRKSEGCEDDIYAFFENEPNVDSLFVKLVEEFERCILSYFAFHWSHGDVLISQVLSSEKPKKKLKHIVMAATREQRFERVTKNLKVARVFNTLVEEMKAMGLVSNDDSTCTEVMAPMALSDRSPMLLFMGGGMGAGKSTVLKDILKEPFWAGAASNAVIIEADAFKESDVIYRALSSRGHQDMIRTAELVHQSSTDAASSLLVTALNEGRDVIMDGTFSWVPFVVQTITMARNVHRRRYRMGVGYKVNEDGSVTENYWERIEDEEPEKVGGKRRKPYRIELVGVICDAYLAVIRGIRRAIMCRRAVRVKSQLRSHKRFADAFMTYCHLVDNARLYSTNSLEGPPKIIVVKLIGWKDKDKTLLVDPEEIDCLKRVARLNEDANSIYELYKRPNPTCE |
| >CrNIP5;1  MEGTSQNLCSYVADTIELQTPTTPQPSSSPLAKFAECYPPGFSRKVLAEVIGTYLLVFVGSGSAGLANIDANKVSKLAASLAAGCIVTVMIYSIGHISGAHMNPAVSLAFAAVRHFPWPQVPFYIAAQLIGAISAAYTLRELFQPSKQIGETQPAGSHIQALIMEMVTTFTMVFISMAVATDTKATGTLSGVAVGCSVSIASIVAGPMTGGSMNPARTLGPAIAISSYKGLWVYFVGPITGALLGAWSYNVIKETDQPGFSFSLLSLSFKLRQNNSGTEQLVKNSHRCSV |
| >CrNIP6;1  MPYSIILLQEYESAGTLSSIFGHSSAMMADSLSVNVDSSPKLELSTEQAHKTNHEAEHSPSKFQKAIAELVGTYIIIFAGCGAALVNEKLQLTIVGIAIVSGLALTVALYSVGHISGGHFNPAVTIALAAVRKVQLKLLMGATLATLTLKVLYHDKVDIGVTVLTYLSSTSDLEAIVWESIITAILMLTICGVATDHRGRKELAGVAIGIAVLINIIIAGPITGASMNPARSLGPAIVSGDYRNIWVYIIGPILGAVFASTLYKLLDVTKPAKTVPFHWCNHNHLPF |
| >CrSIP1;1  MFGAIKAAIGDAVLTFLWVFCSSTLGIAAGAIIRALDVQHLSYNGFPYPSFLVTTALVFILVFFFTVIGEAIGGASFNPTGTASFYAVGLGSDTLFSMALRFPAQALGAAGGALAIMEVIPTEYKHMIGGPSLKVDLHTGAVAEGLLTFIITFAMLFIILKGPRSELLKTWLMATATVILVMAGSAYTGPSMNPANAFGWAYLNNWHNTWDQFYVYWICPFTGAILAAWLFRAIFPPPPPQVKQKKA |
| >CrSIP1;2  MGVIKSAIGDAILTSIWVFIISTLRIVTTEVAIFLGLQPFSFAGLVITTIFNTLYVLTISFIGRMLGGASFNPSTTISFYTAGLRPDSSLVSMAIRFPAQAAGGALGAKTLLQVMPTHYKHMLKGPFLKVDLHTGAIAEGLLTFTHNLAILFIMLKGPKNPFLKVYLLSVATVALVIPGSGFTGPSMNPANAFGWAYTNNKHNTLEQFYVYWICPFIGASSAALIYRFLFMSPTKQKKA |
| >CrSIP1;3  MQIPAFDTYQRFIGMTLGGASFNPSTNISFYIARLRPNSSLASMAIRFPSQAAGGAIAAKVLLLVIPTQYKHMLKGPFLKVYLLSVAIVALVILGSGFTGPAMNLGFAFGWAYMNNKHNTREHFYVYWICPFVGSTLAAFVYRFLFISPTNKKKA |
| >CrSIP2;1  MGRIKLLLFDFVLSFMWVSSSVLIRIFVFKFLGFRHDHLGEIVKTAFSVANMFFFAFLVRLTRGAAHNPLTVLAGAISGDFNNFLYCVAARIPSQVLGSIVGVKLLIYTIPEVGRGPSLNIDIHRGALTEGLLTFAIVTISLGLATKIRENFFMKTWISSLSKLTLHILGSDLTGGCMNPASVMGWAYARGDHITKEHILVYWLAPIEATILAVWTFKLLVQPVKEDRTASKRKSD |
| >CrXIP1;1  MDFADSPVVDIDKQFPRPVQNHEANKKFLDSKLLDSIGAHEIFTKEMWKAALTELTATTFLVFTLTTSIIACLDSNEVDPKLLVPFAVFIIAFLFLIVTVPLSGGHMSPVFTIIAALKGVVTLARALIYILAQCIGSIIGFLILNSVMDPKLADTYSLGGCAISGKDVNSGIKPMDALILEFTCTFVVLFVGVTLAFDKKRSKDLGLPIVCLVVAGAMALAVFVSITVTGRAGYAGVGLNPARCLGPALFRGGPLWEGVVWVDGQYDVLKLAFGSSGTIHNNNGVTNDQTECQAQV |
| **CDS** |
| >CrPIP1;1  ATGGAAGGTAAAGAAGAGGATGTGAGAGTTGGTGCCAATAGATACAGAGAGAGACAACCAATAGGAACTGCTGCTCAAGCCCAAGATGATGCCAAAGACTACAAAGAGCCACCTTCAGCACCTCTTTTTGAGCCTGGTGAGCTATCATCATGGTCTTTCTATAGGGCAGGGATTGCAGAGTTTGTGGCAACGTTTTTGTTTCTTTATATCACGGTGTTAACTGTTATGGGTGTGGCCAAATCCAATTCCAAGTGTTCCACTGTGGGGATTCAAGGCATAGCTTGGGCTTTTGGTGGAATGATCTTTGCTCTTGTTTATTGCACTGCTGGGATCTCAGGGGGTCACATTAACCCAGCAGTGACATTTGGGCTATTCTTGGCACGCAAGCTCTCTCTGACAAGGGCAATTTTTTACATAATTATGCAGTGCTTGGGGGCTATATGTGGGGCTGGAGTAGTGAAGGGGTTCCAACCCCACCAATATGAAAGGCTTGGTGGTGGTGCCAACACACTCAGTAAAGGCTACTCCAAAAGTACTGGCCTTGGAGCAGAGATTGTTGGCACATTTGTCCTTGTTTACACTGTCTTCTCTGCCACTGATGCAAAAAGAAATGCTAGAGATTCCCATGTTCCTATTTTGGCACCACTGCCTATTGGTTTTGCTGTCTTTTTGGTGCACTTGGCTACAATTCCTATTACAGGAACTGGTATCAACCCTGCTAGAAGTCTAGGTGCAGCCCTTATATACAACAAGGACCAAGCTTGGGACAACCATTGGATTTTTTGGGTAGGGCCTTTCATTGGAGCAGCACTTGCAGCATTGTACCATCAGATAGTGATCAGGGCCATCCCCTTCAGGTCCAAGTGA |
| >CrPIP1;2  ATGGAGAGGAATGATGAAGATGAGAGAGTTGGTGCCAACAGGTATGGAGAGAGGCAACCAATAGGAACTGGTGCACAGAGCCAAGATGGGAAAGAGTACAGAGAAGCAGCTCCAGCTCCATTGTTTGAAGCCAGAGAATTAACATCATGGTCTTTCTACAGAGCAGGGATAGCAGAATTTGTGGCCACATTCTTGTTTCTTTATATAACAGTTTTGACACTGATGGGTGTTGCAAAATCTCCCACAAAGTGCTCCACAGTGGGTGTTCAAGGCATTGCTTGGTCATTTGGTGGAATGATCTTTGCTCTTGTCTATTGCACAGCTGGTATCTCAGGGGGTCACATTAACCCAGCTGTGACATTTGGGCTGTTCTTGGCACGCAAGTTATCTCTGACTAGGACAGTGTTTTACATGATTATGCAGTGTTTGGGAGCTATATGTGGTGCAGCTGTAGTCAAAGGATTCCAATCAAAACAATATGAGAGGCTTGGTGGTGCTGCCAACACTCTAAATAAAGGGTACTCCAAAGGTGATGGCCTTGGAGCAGAGATTGTTGGCACATTTATTCTTGTTTACACTGTTTTCTCTGCTACAGATGCCAAGCGAAATGCTAGAGACTCACACGTTCCTATTTTGGCACCATTGCCTATTGGTTTTGCTGTCTTTCTAGTGCATTTGGCTACAATTCCTATCACAGGGACTGGCATCAACCCTGCTAGAAGTTTAGGTGCAGCCATTGTATACAACAAGGACCAAGCTTGGGATGGCCATTGGATCTTCTGGGTAGGGCCTTTTATTGGGGCAGCACTTGCAGCTTTGTACCATCAGATAGTGATGAGGGCCATTCCTTTCAAGTCAAAATGA |
| >CrPIP1;3  ATGGAGAAAGAGGAAGATGTTAAGGTTGGAGCAAACAAATTCTCAGAAAGGCAACCATTGGGAACAGCTGCACAGAGTGACAAGGACTACAAAGAGCCACCAGCAGCTCCTTTGTTTGAGCCTGGTGAGTTAAAGTCATGGTCCTTCTACAGAGCTGGAATTGCTGAGTTTGTGGCCACTTTCTTGTTCCTCTACATCACCATTTTGACTGTTATGGGTGTCAACAATTCATCCTCCAAGTGTTCTTCTGTTGGCATTCAAGGCATTGCTTGGGCTTTTGGTGGCATGATATTTGCCCTTGTCTACTGCACTGCTGGAATATCAGGAGGACACATAAACCCAGCTGTGACCTTTGGTCTATTTTTGGCTAGGAAGCTGTCCTTGACAAGGGCAGTGTTTTACATTGTGATGCAGTGTCTTGGAGCTATATGTGGTGCTGGTGTGGTTAAGGGCTTTGAGGGTAATGCTCGGTATGAGTTGTTCAAAGGTGGAGCCAATTTTGTGAACCCTGGATACACCAAAGGTGATGGCCTTGGAGCTGAGATTGTTGGTACCTTCATTCTTGTCTACACCGTTTTCTCTGCCACTGATGCTAAGAGAAACGCTAGAGACTCTCATGTTCCTATTTTGGCTCCTCTTCCCATTGGATTTGCTGTGTTTTTGGTCCATTTGGCCACCATTCCCATCACTGGAACCGGCATTAACCCAGCTAGAAGTCTTGGAGCTGCCATCATCTTCAACAGGGACCTTGCATGGGATGACCATTGGATTTTCTGGGTTGGACCTTTTGTTGGAGCTGCCCTTGCTGCTTTATATCACCAGATAGTCATCCGAGCCATTCCTTTCAAGACAAGGGCTTAA |
| >CrPIP1;4  ATGGAGAGGGATGAAGATGTTAAGGTTGGAGCAAACAAATACTCAGAAAGACAAGCGTTGGGGACAGGAGCTCAGGGTGAGAAGGACTATAAGGAAGCACCCCCAGCACCATTGTTTGAGCCAGGGGAGTTGAAGTCATGGTCTTTTTACAGAGCTGGGATTGCAGAATTTGTGGCAACGTTCCTGTTCTTGTACATCACAGTCTTAACTGTGATGGGTGTGAACAGGTCACCAAGCAAATGCTCCTCTGTTGGCATTCAAGGAATTGCTTGGGCTTTTGGTGGCATGATTTTTGCACTTGTTTACTGCACAGCTGGAATATCAGGTGGACACATAAATCCAGCAGTGACCTTTGGTCTGTTTCTGGCTAGGAAGCTGTCACTCACAAGAGCGGTATTCTACATTGTGATGCAGTGCCTTGGAGCTATATGTGGTGCGGGTGTGGTGAAGGGGTTTGAGGGTAATGGTAGGTATGAGATGTACAAAGGTGGAGCTAATTTTGTGAGTCATGGATACACCAAAGGTGATGGTCTTGGAGCTGAGATTCTTGGCACTTTTATTCTTGTTTACACCGTTTTCTCCGCCACCGATGCCAAGAGAAACGCCAGAGACTCTCATGTTCCGATCCTGGCCCCTCTTCCTATCGGATTTGCTGTTTTCTTGGTCCACTTGGCCACCATTCCGATCACCGGAACCGGCATTAACCCGGCCAGGAGTCTAGGTGCTGCTATAATATACAACAGAGACCATGCTTGGGATGACCATGTATTGGATTTTCTGGGTTGGACCCTTCATTGGAGCTGCCCTTGCTGCTTTATATCACCAGATCGTGATCCGAGCAATTCCTTTCAAGACAAGGGGTTAATCCTCCTTCGTTATGAATTATCTACCCGGCCTGCTCTTTTTGTCGCTTCATATGTAATATCTATTTGTTTTACTCCATCATGTGTGTAA |
| >CrPIP1;5  ATGGAGGGGAAGGAACAGGATGTTTCATTGGGAGCCAACAAGTTCCCGGAGAGGCAGCCTATTGGAACGGCGGCGCAAAGCCAGGACGACGGAAAGGACTACCAGGAGCCACCACCGGCGCCGCTGTTTGAACCGTCCGAGCTTACATCGTGGTCGTTTTACAGAGCCGGGATAGCAGAGTTCGTCGCCACTTTTCTTTTTCTCTACATCACCATCTTAACTGTGATGGGCGTGAACAGGTCCCCCAAGTGCCAGTCAGTTGGTATTCAAGGAATTGCTTGGGCTTTCGGTGGCATGATCTTCGCTCTTGTTTACTGCACCGCTGGAATCTCAGGGGGTCACATAAATCCGGCGGTGACGTTTGGACTGTTTTTGGCGAGAAAATTGTCGTTGACAAGAGCGCTGTTCTACATGGTGATGCAGGTGCTGGGTGCTATCTGTGGTGCCGGTGTGGTGAAAGGTTTCGAGGGAAAAACCAGGTACGGATTATACAAAGGTGGTGCCAACTTTGTTGCTCCCGGTTATACCAAAGGTGATGGCCTTGGTGCTGAGATTGTTGGCACCTTTATTCTTGTTTACACCGTCTTCTCAGCCACCGATGCTAAGCGTAGCGCCAGAGACTCTCACGTTCCTATTTTGGCACCTCTACCCATTGGGTTCGCTGTTTTCTTGGTGCACTTGGCCACCATTCCTATTACCGGAACTGGTATCAACCCTGCTCGTAGTCTTGGAGCCTCCATCATCTTCAACAAAGACCTTGGTTGGGATGATCACTGGATCTTCTGGGTGGGACCATTCATTGGTGCTGCTCTTGCCGCTCTCTACCACCAAGTCGTCATCCGAGCCATTCCTTTCAAGTCCAAGTAA |
| >CrPIP2;1  ATGTCGAAGGAAGTGAGCGAAGAAGGACAGCAAAGGAAGGACTACGTGGACCCTCCTCCAGCACCTCTTATCGACTTGGCTGAGATTAAGCTCTGGTCCTTCTACAGAGCCCTCATCGCCGAGTTCATCGCCACCCTTCTCTTCCTCTACGTCACCGTCGCCACCGTCATAGGCCACAAGAAACAGAGCGGTCCATGCGACGGCGTTGGCCTTCTCGGCATAGCTTGGTCCTTCGGTGGCATGATCTTTGTCCTCGTCTACTGCACAGCTGGCATCTCCGCACAGTGTCTTGGTGCTATCTGCGGTGTGGGGTTGGTGAAGGCCTTCATGAAGCATCCCTACAACTCTCTTGGTGGCGGTGCTAACTCCGTGTCTTCTGGGTACAACAAAGGCACGGCTCTTGGCGCTGAGATAATCGGGACATTTGTGCTTGTCTACACCGTTTTCTCCGCCACAGACCCCAAGAGAAACGCCCGTGACTCCCATGTCCCTGTTTTGGCCCCGTTGCCAATTGGCTTTGCCGTTTTCATGGTTCACCTGGCTACCATACCCATCACCGGTACCGGAATTAACCCCGCCAGGAGCTTCGGTGCTGCTGTTATCTACAACAACTCCAAAGTTTGGGATGACCATTGGATCTTCTGGGTTGGGCCCTTCGTGGGAGCGTTGGCAGCAGCTGCTTATCACCAGTACATACTTAGAGCAGCAGCTATCAAGGCATTGGGATCGTTCCGAAGCAACCCAACCAACTAG |
| >CrPIP2;2  ATGGCTAAAGAAGTTGAGGTTCAAGAACAAGGAGAATACTCAGCTAAGGACTATCAAGACCCACCTCCAACACCTTTGTTTGATCCTGAGGAGCTCACAAAGTGGTCCTTCTATAGAGCCCTCATTGCTGAGTTCATAGCAACCCTTCTCTTCCTTTATGTCACTGTGTTAACCATTATTGGCTACAAAAGCCAAACTGATTCCACCAAAGGTGGCACAGAGTGTGATGGGGTTGGCATTTTGGGCATAGCTTGGGCCTTCGGTGGCATGATTTTCATCCTTGTTTACTGCACTGCTGGTATTTCTGGAGGACACATAAACCCGGCTGTGACATTTGGGCTTTTCCTAGGACGCAAGGTGTCACTGATAAGGGCGTTGCTATACATGGTAGCACAGTGTGCTGGTGCAATCTGTGGCACTGGATTGGCAAAGGGGTTCCAAAAAGCATACTACAACAGGTATGGAGGTGGTGCCAATTCTGTGGCTGATGGCTACAATAAGGGTACTGCTTTAGGTGCTGAGATTATTGGTACCTTTGTTCTTGTCTACACTGTCTTCTCTGCCACTGATCCTAAGAGGAATGCTAGGGACTCTCATGTTCCTGTACTGGCACCACTACCCATTGGATTTGCTGTGTTCATGGTTCACTTAGCAACAATCCCAATCACTGGTACTGGCATTAACCCTGCAAGGAGTTTCGGAGCAGCTGTAATATACAACAAGGAGAAAATTTGGGATGACCAGTGGATTTTCTGGGTTGGACCAATTGTTGGAGCAGCAGTGGCTGCAATCTACCACCAATACATTCTTAGAGCATCAGCTATCAAAGCTCTTGGATCCTTCAGGAGCAATGCTTAA |
| >CrPIP2;3  ATGGCTAAAGACGTTGAGCAGGTTACGGAGCAACAAGGGGAATACTCGGCCAAGGACTACCACGACCCTCCTCCGGCGCCGTTGATCGACGTGGAGGAGCTCACAAAGTGGTCTTTATACAGAGCCGCCATAGCAGAGTTCATAGCAACCCTTCTCTTCCTTTACATCACCGTCTTGACCATTATCGGGTACATGAGACAGAGCGATACCACAATTCAAGGTAACACCGAATGTGACGGTGTTGGCATTTTGGGCATTGCTTGGGCCTTTGGTGGCATGATTTTCATCCTTGTTTACTGCACCGCCGGTATCTCAGGTGGACACATAAACCCTGCGGTGACATTCGGGTTGTTCGTGGGACGCAAGGTGTCTCTGATAAGAGCGTTACTTTACATGATAGCACAGTGTGCGGGTGCTATATGCGGTGCTGGATTGGCGAAGGGATTCCAAAAAGCATACTACAACAGGTATGGAGGAGGGGTTAACCTTGTGAGCGATGGTTACAACAAAGGTACTGCTTTGGGTGCTGAGATCATTGGTACCTTCGTTCTTGTTTACACTGTTTTCTCCGCCACCGATCCTAAGAGGAGCGCCAGAGACTCTCATGTTCCTGTATTGGCACCACTTCCTATTGGATTTGCGGTGTTCATGGTCCACTTGGCTACAATTCCTGTGACCGGTACCGGTATTAACCCTGCAAGGAGTTTCGGACCCGCTGTTATCTTCAACAAGGACAAAGCCTGGGATGACCAGTGGATTTACTGGGTTGGACCATTTGTTGGAGCTGCTGTGGCTGCATTCTATCACCAATACATTCTGAGAGCAGCAGCTATCAAAGCTCTTGGATCCTTCAGGAGCAACGCTTCATGA |
| >CrPIP2;4  ATGGCTAAGCATGATGTTGAGATTGCTGAGCGTGGTTCCTTTTCTGCAAAGGATTATCATGACCCTCCTCCAGCACCTTTGATTGATGCTGAGGAACTCACAAAGTGGTCCTTCTACAGGGCTTTGATTGCTGAGTTTATTGCCACACTTCTCTTCCTTTACATTACTGTCCTCACTGTGATTGGATACAAAAGCCAGAGTGATCTCAAAGCTGGTGGTGATGTTTGTGGTGGTGTTGGCATTCTTGGCATTGCTTGGGCCTTTGGTGGCATGATCTTCATCCTTGTTTACTGCACTGCTGGAATTTCAGGGGGTCACATAAACCCAGCAGTGACATTTGGGCTGTTCTTGGCTCGCAAGGTGTCTTTGATTAGAGCTATAATGTACATGGTGGCTCAGTGCTTGGGGGCCATATGTGGAGTTGGGTTGGTTAAGGCCTTCCAAAAGGCTTATTACAATAGGTATGGTGGTGGGGCCAATGAACTCAGTGATGGGTACAGCACAGGTGTTGGATTGGGTGCTGAGATCATTGGAACCTTTGTTTTGGTATACACTGTATTCTCTGCCACTGACCCCAAGAGAAGTGCTAGAGATTCTCATGTGCCGGTTTTGGCTCCACTTCCAATTGGATTTGCTGTGTTCATGGTTCATTTGGCAACCATCCCAGTGACTGGCACTGGTATTAATCCTGCTAGGAGTCTTGGAGCTGCTGTCATCTACAACCAAGAGAAGGCATGGGATGATCAATGGATCTTTTGGGTAGGACCATTTGCTGGTGCAGCCATTGCAGCCTTCTACCACCAATTCATCTTGAGAGCAGGTGCAGCTAAGGCTCTTGGATCATTCAGGAGTAACCCCACTGTTTGA |
| >CrPIP2;5  ATGGCGAAAGACGTTGAGGTTACTGAGCGTGGTTCATTCTCTGGCAAGGACTACCATGACCCTCCTCCTGCACCCCTCATTGATGCAGAGGAACTCACCAAGTGGTCCTTTTACAGGGCACTCATTGCTGAGTTCATTGCCACTTTGCTTTTCCTTTACATTACTGTGCTCACCGTTATTGGGTACAAGCACCAGAGTGATGTTAACGATAAGGGTGATGTTTGTGGTGGCGTTGGCATTCTCGGAATTGCATGGGCCTTTGGTGGCATGATCTTCATCCTTGTTTACTGCACCGCTGGAATTTCAGGAGGTCACATTAACCCAGCAGTGACATTTGGGTTGTTTTTGGCTCGCAAGGTGTCTTTGATCCGTGCTATCATGTACATAATAGCTCAGTGCTTGGGGGCTATCTGTGGAGTTGGGTTGGTCAAGGCCTTCCAAAAGTCTTACTACAACAGGTATGGTGGTGGGGCCAATTCTCTTGCTGATGGCTACAGCACAGGCGTTGGATTGGGTGCTGAGATCATTGGCACCTTTGTTTTGGTATACACTGTCTTCTCTGCCACTGACCCTAAGAGGAGTGCTAGAGATTCCCATGTTCCGGTTTTGGCACCACTTCCCATTGGATTTGCTGTGTTCATGGTTCACTTGGCCACCATCCCAGTCACCGGCACCGGCATTAATCCTGCCAGGAGTTTTGGAGCTGCTGTTATATACAACCATGATAAACCCTGGGATGACCATTGGATCTTTTGGGTTGGACCATTTGCCGGAGCAGCCATTGCAGCCTTCTACCACCAGTTCATCTTAAGAGCAGGTGCAGTCAAGGCTCTTGGATCCTTCAGGAGTAACCCTCATGTTTGA |
| >CrPIP2;6  ATGGCAAAAGACATGGAAAGTGCGGCACAAAATGCGTTACCACACAAGGACTACCATGACCCTCCACCCGCACCACTGTTCGACGCCGCCGAGCTCCGTAGTTGGTCCTTATACAGAGCCCTCATCGCGGAGTTTGTCGCTACCCTGCTTTTCCTCTATGTCACCGTTTTGACCGTCATCGGTTACAAACATCAGACCGACGGCGGAGACCCTTGCAACGGCGTCGGGATTCTGGGCATCGCCTGGGCCTTCGGAGGCATGATTTTCGTCCTCGTTTACTGCACCGCGGGAATATCCGGGGGTCACATAAACCCGGCGGTGACGTGGGGGTTGTTCCTTGCTCGGAAAGTGTCGCTGGTTAGAGCATTGAGTTACATGGTGGTTCAGTGCTTGGGAGCTATATGCGGTGTTGGACTCGTGAAGGCTTTTCAGAAGAGCTACTACAACAGATACGCAGGAGGTGCCAACATGCTCTCCGATGGATACAACAAAGGAACCGGGTTGGGCGCCGAGATTATCGGCACCTTCCTTCTTGTCTATACCGTCTTCTCCGCCACCGATCCCAAAAGAAATGCCAGAGATTCCCATGTTCCCGTGTTGGCACCGCTTCCAATTGGCTTTGCGGTTTTCATGGTTCATTTGGCAACCATCCCTATCACCGGCACCGGCATCAACCCTGCCAGAAGCTTCGGCGCTGCCGTCATATACAACAATGAAAAGGCATGGGATGACCAGTGGATATTCTGGGTTGGACCTTTCATCGGTGCTACCATTGCTGCAATCTATCACCAGTTCGTGTTGAGAGCACAGGCGGCAAAGGCTCTGGGATCTTTCAGGAGCTCCTCAAACCTCTAA |
| >CrTIP1;1  ATGGCTCTCTATAGAATTGCAATTGGGTCCCCTGGAGAGGCTGGTCAACCTGATGCACTTAGAGCAGCATTTGCAGAATTTTTCTCCATGATCATTTTTGTTTTTGCTGGAGAAGGATCTGGCATGGCTTATAACAAACTTACCAATAATGGACCTGCAACACCTTCTGGTGTCATAGCTGCATCCCTATCTCATGCATTTGGTCTTTTTGTGGCTGTTTCTGTTGGGGCAAACATTTCTGGTGGTCATGTTAACCCTGCAGTTACATTTGGTGCCTTCTTAGGAGGAAACATAACCCTCTTAAGAAGTATTTTGTATTGGATTGCACAGTTGCTTGGTTCAGTTGTTGCTTGTATTCTTCTCAAGTCTGCTACTGGTGGAATGGAAACATCACCTTTTTCTCTATCCTCTGGTGTGTCTGTTTGGAATGCATTAGTTTTGGAAATTGTGATGACTTTTGGCTTGGTATATACAGTTTATGCCACAGCATTGGATCCAAAGAAAGGGAATGTTGGCATTATTGCCCCAATTGCAATTGGTTTTATTGTGGGTGCCAACATCTTAGCTGGTGCTGCTTTTGATGGTGCATCTATGAACCCTGCTGTGTCCTTTGGCCCTGCTGTGGTTAGCTGGTCATGGACTCATCATTGGGTCTGTTGGGTTGGTCCATTCATTGGTTCAGCAATTGCTGCCATTATCTATGATAACATGTTCATAGGTGATGATGGTCATGAACACCTTTCAAGCAGTGACTTCTAG |
| >CrTIP1;2  ATGCCGATTTCTAGAATTTCCATTGGAAATCCTTCAGAGTTAGGCCAAGCTGATGCGCTTAAAGCAGCTCTCGCTGAGTTCATCTCAATGCTAATCTTTGTCTTTGCAGGAGAAGGCTCTGGCATGGCTTATAACAAGCTCACAAACAATGGCTCAGCAACACCGGCTGGGGTGGTGGCAGCATCATTGTCTCATGCCTTTGCTCTTTTTGTTGCGGTCTCTGTGGGTGCTAACATCTCTGGTGGTCATGTTAACCCTGCTGTCACATTTGGTGCCTTTATTGGGGGCCACATTACCCTTCTTAGAGGCATTTTGTACTGGATTGCTCAGTTGCTTGGCTCTGTTGTAGCTTGCTTGCTCCTTAAACTTGCCACTGCTGGAATGGAAACATCTGCGTTCTCATTATCTTCTGGGGTGGGAGCAGCAAACGCGCTTGTGTTTGAGATTGTGATGACTTTTGGTTTGGTTTACACGGTGTATGCTACTGCAGTGGATCCAAAGAAGGGTAACATTGGGATAATTGCTCCAATTGCAATTGGTTTCATTGTGGGTGCTAACATCTTGGCAGGGGGTGCCTTTGACGGGGCATCAATGAATCCAGCAGTCTCTTTTGGGCCCGCTGTTGTTAGCTGGACATGGGCCAACCACTGGGTCTACTGGGTCGGCCCATTAATTGGATCCGCTATTGCTGCGCTTGTCTACGAGATTTTCTTCATCACCCCAAGTTCTTATGAACAGTTACCTGTCGCCGATTATTAG |
| >CrTIP1;3  ATGCCGATTTCTAAAATTGCCATTGGACATTCTTCTGAGTTGACACAAGCCGATGCGCTTAAGGCTGCACTTGCTGAGTTCATCTCAATGCTCATTTTTGTTTTTGCCGGAGAAGGCTCTGGCATGGCTTATAATAAACTAACAAAAAACGGTTCAGCAACACCAGCTGGATTGGTGGCAGCCTCACTTTCACATGCCTTTGCTCTTTTTGTGGCGGTTTCTGTTGGCGCTAACATTTCTGGCGGTCATGTCAACCCTGCTGTCACTTTCGGTGCCTTTATTGGTGGCCACATTACTCTCTTTAGAACCATTTTGTATTGGATTGCTCAGTTGCTTGGCTCTATCGTTGCTTGCTTGCTCCTTAAAGTTGCCACTGGAGGATTGGAAACATCTGCATTTGCGCTGTCTTCAGGAGTGGGTGCGGGGAATGCGTTTGTGTTTGAGATTGTGATGACTTTCGGTTTGGTTTACACGGTGTATGCAACTGCGGTGGACCCAAAGAAGGGTGATCTTGGAATAATTGCTCCAATTGCAATTGGTTTCATAGTTGGTGCTAACATCTTGGCGGGTGGAGCATTTGATGGTGCATCCATGAACCCTGCGGTCTCCTTTGGGCCTGCTGTTGTTAGCTGGACATGGGATAATCATTGGGTCTATTGGGCCGGCCCATTCATTGGTTCTGCTATTGCTGCTCTTGTTTACGAAATTTTCTTCATTAACCAAAACACCCATGACCACCTCCCCACCACAGATTATTAG |
| >CrTIP1;4  ATGCCGATCAGAAATATCGCCGTTGGAAGGCCTGAGGAGGCCACTCACCCAGATACCTTGAAGGCTGCGTTGGCTGAGTTCATCTCCACCCTCATCTTCGTCTTCGCTGGCTCAGGTTCCGGCATCGCCTACAACAAGCTCACCGACAACGGTGCAGCCACCCCCGCCGGTCTCATCTCTGCCGCCATAGCCCATGCATTCGCCCTCTTTGTTGCCGTCTCCGTCGGCGCCAACATCTCAGGCGGCCACGTAAACCCCGCCGTCACCTTCGGTGCCTTCATCGGTGGCAACATCAGCTTCCTCCGCGGTATCGTGTACATCATCGCCCAGCTCCTGGGCTCCATCGTGGCCTCCTTGCTCCTGCTCTTCGTCACCGGATTGCCTGTTCCAGCATTCGGACTCTCTGCAGGAGTTGGAGTGGGAAACGCTTTGGTGTTGGAAATCGTGATGACTTTCGGATTGGTGTACACGGTGTACGCCACAGCCATTGATCCTAAGAAGGGTAATTTGGGAATTATCGCCCCCATCGCTATCGGTTTTATCGTTGGCGCTAACATTTTGTTGGGTGGGGCCTTCGATGGAGCATCCATGAACCCGGCCGTGTCATTCGGACCTTCAGTAGTGAGCTGGAGCTGGAACAACCACTGGATCTACTGGGTTGGGCCTCTCATCGGTGGTGGGCTTGCTGGGCTTATCTACGAGGTCATCTTCATCAGCCACACCCACGAGCAGCTCCCTACCACTGACTATTAG |
| >CrTIP2;1  ATGGTGAAGATAGCACTTGGTACCTTTGATGATTCCTTTAGTATTGCCTCCCTTAAGGCTTATCTAGCAGAGTTCATTGCCACTTTGCTTTTTGTGTTCGCTGGTGTTGGATCAGCCATCGCTTATAACGAGCTGACATCAGATGCAGCCTTGGATGCACCGGGCCTGGTGGCAGTAGCTGTGGCCCATGCATTTGCACTATTTGTAGGTGTGGCCATCGCAGCCAACATCTCAGGTGGCCATTTGAATCCAGCTGTCACATTTGGATTGGCTATTGGAGGCAACATCACACTCCTCACTGGTTTCTTGTACTGGATTGCCCAGTTGCTGGGCTCAATCGTCGCTTGTCTCCTCCTAAATTTTGTCACCGCTAAGAGCGTTCCAACCCACGGAGTGGCTGCTGGTGTGAACGTTTTTGCAGGTTTAGTGTTTGAGATTGTTGTGACTTTTGGATTGGTTTACACTGTGTATGCCACTGCAGCTGACCCCAAAAAGGGCTCACTGGGCATCATTGCACCCATTGCTATTGGGTTTATTGTGGGTGCCAACATCTTAGCTGCTGGCCCATTCAGTGGTGGTTCTATGAACCCGGCTCGTTCATTTGGACCGGCTGTGGTTAGTGGAAACTTCGTTGATAACTGGATCTACTGGATTGGGCCACTGATTGGAGGAGGTTTGGCTGGGTTGATTTATGGTGACATCTTCATTGGTTCCTATACCCCTGCCCCACCCTCTGATACCTATCCTTGA |
| >CrTIP2;2  ATGGCTGGCATTGCATTCGGACGCTTCGATGATTCTTTCAGTTTGAGCTCAATCAAGGCCTATATTGCTGAGTTCATCTCAACCTTACTCTTTGTTTTTGCCGGTGTTGGTTCAGCCATAGCCTATGGTAAATTAACTTCAGATGCAGCACTAGATCCAGCTGGATTAGTAGCAGTTGCTATTTGTCATGGTTTTGCTCTATTTGTTGCTGTTTCTGTGGGTGCCAACATCTCTGGTGGCCATGTCAACCCTGCTGTGACCTTTGGATTGGCTCTTGGTGGCCAGATAACCATCCTCACTGGCATCTTCTACTGGATTGCACAGCTTGTTGGCTCCATTGTGGCATGTTTTCTCCTCCACTTTGTCACAGGAGGCTTGACAACTCCCATCCATAGTGTGGCTGCAGGGGTTGGAGCTGTTGAAGGAGTTGTTACAGAGATCATCATCACATTTGGTTTGGTGTACACAGTGTATGCCACAGCAGCTGATCCCAAGAAAGGTTCATTAGGTACCATTGCACCCATTGCCATTGGTTTCATTGTTGGTGCCAACATCTTGGCAGCAGGACCATTCTCTGGAGGGTCAATGAACCCAGCACGCTCCTTTGGCCCTGCAGTTGTTAGTGGTAACTTCCATGACAATTGGATCTACTGGGTTGGACCTCTCATTGGTGGTGGTTTGGCTGGCCTTATCTATGGCAATGTTTTCATTCGCTCTGACCATGCACCTCTTTCTAGTGAATTTTGA |
| >CrTIP3;1  ATGTCAACCCGTAGATATGCATTTGGAAGGGCAGATGAGGCCACACACCCAGACTCCATGAGGGCTACTTTAGCTGAATTTGCCTCCACTTTCATCTTTGTCTTTGCTGGAGAAGGCTCTGGCCTTGCTTTGGTTAAGATTTACCAGGATTCAGCTTTCTCAGCTGGTGAATTATTGGCAGTTGCACTTGCACATGCCTTTGCTCTATTTGCTGCTGTATCTTCTAGCATGCATGTATCAGGTGGCCATGTCAACCCGGCTGTGACATTCGGTGCTCTCCTTGGGGGAAGGATCTCAGTGGTCCTTGCCATATACTACTGGATAGCTCAACTTCTTGGTGCTATTGTGGCTGCTCTCATACTCAGGCTTGTCACTAATAACATGAGACCATCGGGGTTCCATTTGGCACCTGGTGTTGGAGCGGCACACATGCTTATACTTGAGATTGTCATGACATTTGGGCTGATGTACACTATATATGGTACAGCAATTGATCCCAAAAGGGGTTCAGTTAGCAACATTGCACCTTTGGCAATTGGACTTATTGTTGGGGCAAACATCCTTGTTGGTGGGCCATTTGATGGAGCATGCATGAACCCTGCTCTTGCTTTTGGGCCATCCTTGGTGGGCTGGAGATGGCACTACCACTGGATCTTCTGGGTGGGTCCATTGATTGGGGCTGCACTGGCAGCAATCATATATGAATATGTTGTGATCCCAACTGAACCCCCTCATCAACATCAACCATTGGCTCCTGAAGATTACTAG |
| >CrTIP3;2  ATGGCAACAAACCGAAGATATGCAATTGGAAGGTTGGATGAGGCTAACAACCCTGATTCCATGAGAGCCACCTTAGCTGAATTCCTCTCCACTTGCATTTTTGTGTTTGTTGGAGAAGGCTCTGCCCTTGCTTTGAGCCAGATTTACAAGGATACAGGTACATCAGCTGGTGAGCTAGTGGTTGTTGCACTAGCTCATGCTTTTGCTCTATTTGCTGCTATTTCTGCTACCATGCATGTCTCTGGTGGACATGTCAACCCTGCTGTCACTTTCGGTTCTCTTCTTGCTGGCAAGATCTCTGTCCTTAGAGCCGTTTACTACTGGGTTGCTCAACTTCTCGGTTCTGTAGTCGCTGCCCTTTTGTTGAGGCTTGTCACCAACAATATGAGACCACAGGGGTTCGGTGTGTCTGTGGGTGTTGGAGCGTTTCACAGCCTTATTCTTGAGATTGCCTTGACATTTGGTCTGATGTACACGGTGTATGCTACTGCTCTTGATCCTAAAAGGGGCACCGTTGGTTCAATTTCGCCCTTAGCAATAGGACTTGTTGTTGGGGCAAACATCCTTGTTGGTGGGCCGTTTGATGGAGCATGCATGAACCCAGCTCGGGCTTTTGGGCCTGCCTTGGTGGGCTGGAGATGGCACTACCACTGGATCTTCTGGGTGGGTCCATTGATTGGGGCCGCCCTGGCAGCACTGTTATATGAATATGTTATGGTCCAAACTGAGCCTCCTCATGCTCATCACCAACCTTTGGCTCCTGAAGATTACTAG |
| >CrTIP4;1  ATGCGCCATTTGTCCGTTTGTATTATGTGGGAATCAAGTAATATTCATTGCTTGTGCAATGGAGAATCAGCCTCAGCCATGGCCAAAATCGCTCTTGGAACCACCCGAGAGGTCACTCAACCAGATTGCATTCAGGCACTCGTCGTTGAATTCATCGCCACCTTCCTCTTTGTCTTTGTTGGCGTAGGTGCTTCTATGGCCGTTGACAAGTTTGTTGGGGATGCACTGGTGGGCTTGTTTGCTGTAGCAGTGGCACATGCTCTTGTGGTGGCTGTAATGATCTCCTCCGCCCACATTTCCGGTGGCCACCTCAACCCCTCCGTCACTCTTGGTCTCCTTGCCAGCGGTCACATCACCCTCTTCCGCTCCATCCTTTATTGGATTGATCAATTAATAGCATCTGCAGCCGCTTCGTTTCTGCTTTACTACCTTTCAGGAGGACAGAATACTCCAGTTCATACGCTGGCGAGTGGAGTGGGGTATGGTCAGGGAGTAATTTGGGAGATTGTGTTGACGTTTGGTTTGTTGTTCACCGTGTATGCGACAATGGTGGATCCAAAGAAAGGAGCACTTGCTGGGATTGGACCAACGCTGGTTGGGTTTGTAGTGGGTGCCAATATCCTTGCCGGTGGGGCATTCTCTGCTGCTTCTATGAACCCAGCAAGGTCTTTTGGCCCTGCCTTGGTTTCTGGCAACTGGACTGATCATTGGGTTTACTGGGTTGGACCTCTCATTGGTGGTGGCCTTGCAGGTTTCATCTATGAGACTTTCTTCATTGACCGATCTCATGTTCTACTTTTCCCTGATGCAGAAAGTTAA |
| >CrTIP5;1  ATGGCTCCTTCTTCTGTCACTGTCACTTCCCGCTTTCATGAATCCGTTACCCGAAATGCACTTCGCTCCTATCTCTCCGAGTTTGTCTGCACTTTCTTCTTTGTCTTTCTTGTGGTTGGCTCTCAAATGTCCTCAAGGAAATTGATGCCTGATGCTTCATTGAACCCAACGAGTCTGGTGGTGGTTGCGATGGCGAATGCTTTTGCTTTATGTTCTGTTTTATATGTCGCATGGGATATTTCCGGTGGACACGTGAATCCGGCGGTGACGTTTGCAATGGCAGTAGGAGGTCACATTAGTGTACCAACCGCTCTCTTCTATTGGGTTGCTCAGCTTATAGCCTCAGTTATGGCTTGCCTTGTCCTAAGGGTCATTGTTGTTGGAATGCATGTGCCAACATACACCATTGCGGAAGAAATGACAGGATTTGGAGCATCCGTATTAGAGGGTATCCTCACATTTGTTTTGGTGTACACTGTGTATGCTGCTAGGGACCCTAGACGTGGGCCTATGAGTTCTACAGGCCCACTTGCGGTTGGGCTAATAGCTGGGGCAAATGTGTTGGCTGCAGGCCCATTCTCTGGAGGGTCAATGAACCCAGCATGTGCTTTCGGCTCAGCCGCTATTGCTGGCAGTTTCAGGAACCAAGCAGTGTATTGGGTTGGACCTTTGATTGGTGCTTCAATTGCTGGCCTCCTTTATGACAATGCACTCTTCCCTTCTCACACTACACATTCAGTTACCCAAGGACTTGCTGTGTAA |
| >CrNIP1;1  ATGGGTGATAATTCAGCAAGCAATGGAGGCCACGAGGTGGTTTTAAACGTAAACGATGATGCTTCCAAAAAGACTGAGGACTCAGCTATCGAACATTCTGTGCCTCTTTTGCAAAAGTTGGCAGCTGAGGCGGCGGGGACGTACTTCTTGATATTTGCTGGTTGTGCATCGGTGGTAGTGAACCTTGGAAACGACAAGGTACTGACATTTCCAGGAATTTCCATTGTTTGGGGACTCACTGTTATGGTATTGGTTTACTCTCTTGGTCATATTTCCGGTGCTCATTTCAACCCTGCTGTCACCATTGCTCATGCTTCCACCAAAAGGTTTCCTTTCAAGCAAGTACCTGCGTATATAATAGCTCAGGTCATTGGATCCCTACTTGCTAGTGGAACTCTCAGACTTATATTCAATGGTAAGAGTAACTATTTTCCAGGAACAGTACCGGCTGGTTCTGACTTGCAAGCCTTTGTGATTGAATTCATAATCACTTTGTTTCTCATGCTAGTCATTTCTGGAGTCGCCACCGATAATAGAGCGATTGGTGAGTTGGCAGGGCTTGTAGTTGGGTCTACGGTGCTGTTAAATGTGTTGATTGCCGGGCCAATTACTGGAGCATCAATGAATCCAGCAAGAAGCTTAGGCCCTACTATTGTGTACAACGAGTACAGAGGAATATGGATATATTTGGTGTCACCGATTCTGGGAGCTGTGGCGGGTACATGGATCTACAATTTCCTCAGGTACACCACAAAGCCTGTGCCTGAGATCACCAAGAGTGCCTCTTTCCTCAAAGGACCAGAATGA |
| >CrNIP1;2  ATGGGAGATAATTCAGCAAGCAATGGAAGCGACGAGGTGGTTTTAAACGTGAACGGTGATGTTTCTGTAAAGTGTGAGGACTCTGTGCCTCTTCTGCAGAAGTTGGTAGCAGAGGTGGTGGGAACGTACTTCTTGATATTTGCAGGATGTGGTTCGGTGGTGGTGAACCTTAGCAAGGACAAGATGGTGACGCAGCCAGGAATTTCTATTGTTTGGGGACTCACCGTTATGGTATTGGCTTACTCTCTTGGTCATATCTCCGGTGCTCATTTCAACCCTGCTGTTACCATTGCTCATGCTTCCACCAAAAGGTTTCCCTTCAAGCAGGTACCGGGTTACATAGTAGCTCAAGTCGTTGGATCCACACTTGCTAGCGGAAGTCTCAGACTTATATTCAATGGCAAGAATGACCATTTTGCAGGAACACTACCCGCTGGTTCTGACTTGCAAGCCTTTGTGCTCGAATTCATAATCACTTTCTACCTCATGTTCGTCATTTCTGGAGTCGCTACCGACAACAGAGCGATTGGTGAGTTGGCAGGGCTTGCAATTGGGTCTACGGTACTTCTAAATGTGATGTTTGCCGGGCCAATAACAGGAGCATCAATGAATCCAGCAAGAAGCTTAGGACCTGCTATTGTGCACAATGAGTACAGAGGATTATGGATATATTTGGTGTCACCAACGGTAGGAGCGGTGGCGGGTACATGGGCCTACAATTTCATCAGATACACCAACAAGCCTGTGCGTGAGATCACCAATAGTGCCTCTTTCCTCAAAGGATCACCACCTGAGGGTGGATCCCACTGA |
| >CrNIP1;3  ATGAAGAGTACAATTAGGAAGATTGGAGACAGAGGAGAGAGTGTTGTGGGCAATATACAAACCCTGAGTTATATTTCTCAGTTGTTTCCTTCTACTCTGTGGCTGATGGCTGATAATTCAGCAAGAAGTGAAACCCAAGAGGTAGTTTTAAATGTCCCAAAGGACCCCTCCAAAACATATGAACGCTCAGACTCCTATGTTTCTGTGCCTTTCTTGCAGAAGTTGGTAGCAGAGATAGTGGGGACATATTTCTTGATATTTGCAGGGTGTGCTTCAGTGGTGGTTAATAAGAATAATGACAATGTTGTCACACTTCCTGGGATCTCAATTGTTTGGGGACTGGCTGTGATGGTCTTGGTTTACTCCGTTGGTCACATCTCTGGTGCCCATTTCAACCCTGCTGTCACCATTGCTTTTGCCTCCACCAGAAGGTTTCCGTTGATGCAGGTACCGGCTTATGTAGCTGCTCAGCTTCTTGGAGCCACACTTGCAAGTGGAACTCTGAAACTGATATTTAATGGGAGCCATGACCAGTTTTCAGGAACACTCCCAGCTGGATCTAACCTTCAAGCTTTTGTGATTGAATTCATAATCACTTTCTATCTTATGTTTGTCATATCTGGGGTTGCCACCGATAACAGAGCGATTGGCGAGTTGGCGGGGCTTGCAATTGGGTCTACAATACTGCTGAATGTGATGATTGCAGGGCCAATCACAGGAGCATCAATGAACCCAGTTAGAAGCTTAGGACCTGCTTTTGTACACACTGAATACAGAGGAATATGGATATACCTGTTATCACCGGTTGTTGGGGCAGTGGCTGGAGCATGGGTCTACAACATCATAAGGTACACCGACAAGCCCTTGCGTGAGATCACCAAAAGTGCCTCTTTTCTCAAAGGATCAGGCCGGTGTGCTTAG |
| >CrNIP2;1  ATGGCCAACAAAGCTGAAGGCATCCAAGAAGAAGAAATGTCAAGAGTGGAAGTGGGTGTTAATCGCTGCCCTTTCAACTTTTCTGGCTTGCCTAGCTGTTGTTCATCAAACTATGTTGTAACCCTGACACAAAAGGTGATCGCAGAATTCATTGGCACATATTTTGTGGTATTTGCTGGTTGCGGTTCTGTGGCAGTGAATAAGATCTATGGCTCTGTCACATTTCCTGGCATTTGTATCACATGGGGGCTTATTGTAATGGTCATGGTCTACTCCGTTGGTCATATCTCTGGAGGTCACTTTAATCCTGCGGTCACTATCACTTGGGCCATTTTTCGCCGATTCTCATACAAAGAGGTGCCAATATACATTGTTGCTCAGTTGCTGGGGTCGATACTTGCTAGTGGCACATTAGCCCTAATGTTGGACGTCACACCTAAAGCTTATTTTGGAACGGTACCAGTTGGATCTATTGGCCAGTCTTTAGCTGCAGAAATCATCATCACTTTTCTCTTAATGTTTGTCATATCTGCCGTATCTACAGATGACAGAGCGGTAGGTGACTTTGCAGGAGTTACAGTGGGAATGACTATAATGTTGAATGTCTTTGTTGCAGGGCCCGTATCAGGAGCTTCCATGAACCCTGCAAGAAGTATTGGTCCCGCGCTCATCAAGCATGTTTACAAAGGGTTATGGGTGTATATAGTTGGTCCGATTATTGGAGCCATAGCTGGAGCATTTGTCTATAACTTTCTTAGATCCACGGAAGAGCCACACTCTGAATAA |
| >CrNIP2;2  ATGGCTGAAAACCAGATCACAGGAATGGAAGAAGGTGGAGTCCAGTCACAGAAGGATTCTAGCTTTCGTGATTCTCCTGATGTAGTTCAAATTATACAAAAGGTAGTTGCAGAGGTGATAGGGACATATTTCTTCATATTTGCAGGGTGTTGCTCTTTGGTTTTGAATAAAGTTGAAGAGAGTAAAGGGTCAATAACGTTTCCTGGAATTTGTGTGGTATGGGGTGTAACCCTAATGATCTTGGTTTATTCTCTCGGTCACATTTCTGGTGCTCATTTCAATCCTGCAGTTACTGTTAGCTTAGCCATCTATCGCCAATTCCCTTTAAAACAGGTGCCTCTATATATTATTGCACAAATAGTAGGATCGGTCCTTGCTAGTGGGACATTGGACCTTCTCTTTGATGTAGATGATAATTCTTATTTTGGAACAGTACCAACAGGATCTTATACTCGATCTCTTGTTTTTGAGATACTCACAACATTTCTCTTATTGTTTGTTATTTCTTCAGTTACCACGGACAATGGAGCGGTTGGAGAGTTGGCAGGTGTGGCTATTGGTATGACAATCTTAATAGACCTCTTCATTGCAGGAAATGTGTCAGGAGCATCTATGAACCCAGCCAGAAGTCTAGGACCAGCATTGGTGATGCATATTTACACAGGATTTTGGATTTATATAGTTGGGCCATTTCTTGGTGGTATATTAGGTGTCACAGCCTACAATTTGATTAGATTCAATGAGAAACCACTAAGCTCAATCAGAAAATAA |
| >CrNIP3;1  ATGGAAACAAATGAGGAAATTCCATCAATGCCTACAACACCAGGCACTCCTGGTGCACCTCTTTTCGGTGCCTTCAATGACAATCATAACAATAAGAAATCTCTCCTCAAGAATTGTAAGTGCTTCAGTGTGGAAGAATGGACGATTGAAGATGGAGCGTTACCAGCTGTATCATGTTCATTACCGTTGTCACCTCCTCCTGTGTCTCTTGCAAGAAAAGTCGGAGCTGAATTCATCGGAACCTTCATTCTCATGTTCTCTGGCACTGCCGCTGCTATTGTCAACCAAAAAACACCTGGCTCCGAGACTCTCATTGGATGTGCTGCCTCCACCGGCCTCGCCGTCATGATCGTCATTCTCGCCACCGGCCATATATCCGGCGCTCATCTCAACCCTGCCGTCACCATTTCCTTTGCTGCATTAAAGCACTTCCCATGGAAACATGTGCCTATGTATATCGGTGCTCAGGTTTTGGCATCTATATGCTCTGCCTTTGCTCTGAAAGGGATTTTTCATCCTTTCATGAGTGGTGGAGTCACCGTTCCTTCCGGAGGATATGGCCAAGCTTTTGCTTTAGAGTTCATTATTGGCTTCAATCTCATGTTCGTTGTAACTGCAGTCGCCACCGACACCAGAGCTGTGGGAGAACTCGCGGGAATCGCGGTGGGAGCAACTGTAATGCTCAATATACTCATTGCAGGGCCAGCTACGGGAGGGTCAATGAACCCAGTGAGAACACTGGGTCCAGCTATCGCCGCGAACAACTACAAAGCCATATGGGTCTATCTGGTGGCTCCCGTTCTCGGAGCTTTAGGGGGGGCAGGTACCTACACTGCAGTCAAGCTGCCAGAAGAAGATGATAACGCCAAGGCAAGGGCTTCAATCAGCTTCAGAAGGTGA |
| >CrNIP3;2  ATGGATAATGCAGAAATTCCATCAGTTCCTTCAACACCTGCTACACCAGGAACTCCTGGTGCTCCTCTTTTTGGAGGGTTCAAGTCTGAGAGAACTGGGAATGGTATTGGTAAGAAACCATCCCTTCTCAAAAGTTGCAAATGTTTCAGTGTTGAAGAATGGACCTTAGAAGATGGAACCTTGCCTAAACTCTCTTGCTCTTTGCCACCCCCTCCTGTGCCTCTTGCAAAAAAGGTTGGAGCTGAGTTTATTGGCACCTTCATTCTCATGTTTGCTGCAATAGGCACTGCTATTGTGAACCAAAAGACACATGGCTCTGAGACTCTGGTTGGATGTGCTGCAGCTAATGGACTTGCAGTCATGATCATCATTTCCTCCACTGGCCATATCTGTGGTGCTCATCTCAACCCTGCTGTCACCATTTCCTTTGCTGCATTAAAGCACTTCCCGTGGAAAAATGTGCCAGTGTATATAGGTACACAACTTTTGGCATCAATATGTGCTGCATTTGCTCTGAAGGAGGTTTTTGACCCCTTTATGAGCGGTGGAGTGACGGTCCCTTCAGTAGGATATGGCCAAGCATTTGCTATAGAGTTCACTGTCAGCTTTATTCTCATGTTCGTTGTCACTGCCGTCGCCACTGATACAAGAGCTGTGGGAGAGCTCGCAGGAATCGCGGTGGGAGCCACTGTCATGCTCAACATACTCATAGCCGGGCCAGCAACTGGAAGTTCAATGAACCCTGTAAGAACACTAGGTCCAGCAATTGCTACAAACAACTACAGAGGAATATGGGTCTATCTCACTGCTCCAATACTTGGAACTCTATGTGGGGCTGGTGCTTACACTGTTGTCAAGTTGCCTGATCAACGTTTTAATTCTCAGGCAAAGGCACCTTCAGCTCCTTCCACATTCACTAGGTGA |
| >CrNIP3;3  ATGCCGGACGAGGAGATAGGGACGCCGACGGCCGCGTCGGTTCCGGCAACGCCGGATACTCCGGGAGGACCACTATTCACGTCGCTGCGAGTTGACTCACTATCACACGAACGTGACTCGTTTGCAAAGGCTCGGTGCAAGTGCTTGCCGACCAAAGGTCATACCTGCTTCACCGATTTCTCAGTTGGGGTTCCAATTCCCAATGTATCTCTCACTCAGAAGATTGGAGCAGAGTTTGTGGGGACATTCATATTGATATTTGCATCAACGGCTGGACCGATAGTGAACAACAAGTACAATGGAGCAGAGGGTTTGTTAGGGAATGGAGCTACTGCAGGATTAACAGTAATGTTCATTATTCTCTCCATTGGCCACATCTCAGGTGCACATCTCAACCCGGCTCTCACCATTGCATTTGCAGCTTTTCGACATTTCCCTTGGGTCCATGTCCCTGCTTATGTAGCTGCACAAGTCTCTGCCTCCATCTGTGCTGGTTTCGCTCTCAAAGCTGTTTACCATCCTTTCCTCTCTGGTGGTGTCACCATCCCTTCTGTCACCATTGGCCAAGCTTTTGCAACCGAGTTTATTATCACTTTTAATCTCTTGTTTGTCGTCACTGCTGTTGCTACCGATACTCGTGCGGTTGGTGAATTGGCAGGTATTGCTGTTGGGGCTACAGTTTTGCTCAACATTCTCATATCAGGGCCAACAAGCGGTGGTTCGATGAATCCGGTGCGCACCTTAGGTCCAGCAGTTGCAGCAGGAAATTTCAAGCATATATGGATATATTTGGTGGCTCCTACGCTGGGTGGACTCGCTGGTGCTGGCGTTTATACGCTTGTAAAGCTGCGTGACAAGGATGGTGAACCGCCGCGACAAGCTAGGAGTTTCCGTCGCTAG |
| >CrNIP4;1  ATGGCAGAGGTGGTGGGTACTTTTATTTTGATGTTCTGTGTATGTGGAATCAATGCAAGCACACAATTCCAAAATGGTGCAGTGGGCCTTCTGGAGTATGCAGCTACAGCAGGATTAACAGTGATAGTGATAATTTTCTCTATAGGGCCAATATCTTGTGCGCATGTTAACCCAGCTGTCACAATAGCCTTTGCAACAATTGGTCAATTTCCATGGTTCAAGGTACCAGTTTACATAATAGCACAGACAGTAGGTTCTATGTCGGCAACATACATAGGTAGCCTTGTGTATGGCATAAAATCAGATGTTATGATGACACAGCCACTCCAAGGGTGCAACTCTGCCTTCTGGGTGGAGGTTATTGCAACTTTCATCATCATGTTCCTCATTGCTGCTTTGACGTTTGAATCTCAATCAGTAGGCCATTTATCTGGTCTAGTAGCTGGAATAGCAATTGGGCTTGCTGTACTAATCACAGGCCCTGTCTCAGGTGGATCAATGAATCCTGCAAGATCTTTAGGTCCAGCAATTGTGTCATGGAAATTTAAGGACATATGGATATACATCCTAGCTCCTTGTGTTGGAGCTGTAGCTGGAGCTCTAATGTTTCATGTTCTACGTCTTCGAGAACAACATTGTAGTCCTTTGTCCTCCCAAAACATTAGAGATGTTGGTCGTCCCATACCCTTATGCTCAAAGTATAATGACAAGTCCACCTTCACACAAATCGTCGTGGCCTCCTCTATTGGGTTGATTTTGGCTGCAACAATGCATTATAATGTTAAAAGAATGAGAGATCGAAAGATTGTTCCACGTTTGAGATTTTCAAAGACAAGACAAATTCCAAAGCTTGAGAAGTTCTCTCATTACGTAGCTAGGCAAATGGGGTTCAAAGACAAGAAAAGTTGTCCTCTTCTTTGTAAATTGGCTTCTGAATACATAAGGAAATCTGAAGGATGTGAAGATGATATATATGCTTTTTTCGAGAATGAACCAAATGTGGATTCACTTTTTGTGAAGCTTGTAGAGGAGTTTGAGAGATGCATTCTTAGTTACTTTGCATTCCATTGGAGCCATGGTGATGTATTGATAAGTCAGGTGTTAAGCTCAGAGAAGCCAAAAAAGAAGCTCAAGCACATAGTTATGGCAGCAACTAGGGAACAAAGGTTTGAGAGAGTAACAAAGAATCTGAAGGTGGCTAGAGTTTTTAATACATTAGTTGAAGAGATGAAAGCAATGGGACTTGTATCAAATGATGATTCTACATGCACAGAGGTGATGGCTCCAATGGCTCTTAGTGATAGGAGCCCAATGCTTCTTTTCATGGGAGGTGGTATGGGAGCTGGAAAGAGCACTGTTCTTAAGGATATTTTGAAAGAACCCTTTTGGGCAGGAGCAGCAAGCAATGCAGTCATCATTGAGGCAGATGCCTTCAAAGAATCAGATGTCATATATAGAGCTCTTAGTTCAAGAGGGCATCAGGACATGATTCGAACAGCTGAATTGGTACACCAATCATCAACAGATGCAGCCTCATCCCTGTTGGTAACAGCATTAAATGAGGGGAGGGATGTAATTATGGATGGCACATTCTCTTGGGTACCATTTGTTGTGCAGACCATAACAATGGCCAGAAATGTGCATCGCCGCCGTTACCGTATGGGAGTTGGCTATAAGGTGAATGAGGATGGAAGTGTAACAGAAAACTATTGGGAAAGAATTGAGGATGAAGAACCTGAAAAAGTTGGAGGTAAAAGGAGAAAACCATATAGGATAGAGCTAGTTGGAGTAATATGTGATGCTTACCTTGCAGTCATTAGAGGCATAAGGAGAGCTATCATGTGTAGAAGAGCAGTGAGAGTGAAGTCACAGTTGAGATCCCATAAAAGATTTGCTGATGCATTTATGACTTATTGTCACCTAGTAGACAATGCTAGACTATACAGTACAAACTCTTTAGAAGGCCCACCTAAGATAATTGTTGTAAAGTTGATAGGGTGGAAAGATAAGGACAAAACACTGCTTGTTGATCCAGAAGAAATTGATTGTTTGAAGAGGGTTGCTAGGTTGAATGAAGATGCCAATTCCATATATGAGCTTTACAAGCGTCCTAATCCAACTTGTGAA |
| >CrNIP5;1  ATGGAAGGGACCAGCCAAAATCTGTGCAGCTATGTCGCTGACACTATTGAGCTGCAAACTCCCACCACCCCCCAGCCATCATCATCCCCTCTTGCAAAATTTGCAGAGTGTTACCCTCCTGGGTTTTCTAGAAAGGTACTGGCAGAGGTCATAGGGACATACCTATTGGTGTTTGTGGGAAGTGGGTCTGCTGGTCTTGCAAATATTGATGCAAACAAAGTGTCAAAACTTGCAGCTTCACTTGCAGCGGGATGCATAGTAACGGTGATGATTTATTCGATTGGGCATATCTCTGGAGCGCACATGAATCCAGCAGTGTCTTTAGCTTTTGCCGCCGTGAGGCATTTTCCATGGCCACAGGTTCCATTTTACATTGCAGCTCAACTCATAGGAGCCATTTCTGCGGCATATACACTGCGAGAGCTATTTCAGCCATCCAAGCAGATTGGGGAAACACAACCTGCTGGATCACACATTCAAGCACTAATCATGGAAATGGTGACCACATTCACCATGGTCTTCATCTCCATGGCCGTGGCCACCGACACAAAAGCGACGGGAACCCTATCAGGAGTAGCAGTAGGTTGTTCTGTTAGCATAGCAAGCATTGTTGCCGGACCAATGACAGGGGGATCAATGAACCCAGCAAGGACATTAGGTCCTGCAATTGCAATTTCATCCTACAAGGGACTTTGGGTCTATTTTGTTGGGCCAATCACTGGGGCACTTTTAGGGGCATGGTCTTATAATGTGATTAAGGAGACAGATCAGCCAGGTTTTTCATTTTCACTACTCTCCCTTTCCTTCAAGCTACGCCAAAACAATAGTGGAACTGAACAACTTGTCAAAAACAGCCACCGATGCTCGGTGTGA |
| >CrNIP6;1  ATGCCATATAGCATTATATTGTTGCAGGAATACGAATCTGCAGGTACACTTTCATCTATTTTTGGGCATTCAAGTGCTATGATGGCTGATTCACTGTCAGTTAATGTTGACTCTTCACCTAAGCTTGAATTATCCACCGAACAAGCACATAAAACAAACCATGAGGCTGAACACTCTCCTTCTAAATTCCAAAAGGCCATTGCCGAACTCGTGGGTACATACATTATTATATTTGCGGGTTGTGGAGCTGCCCTTGTCAACGAAAAGTTGCAACTTACAATAGTAGGTATAGCAATTGTTTCGGGTCTTGCTCTCACAGTTGCATTATATTCGGTTGGTCATATTTCTGGTGGTCATTTTAATCCTGCAGTCACAATTGCTTTGGCTGCGGTCAGAAAAGTTCAACTGAAACTTTTGATGGGTGCTACATTGGCCACTCTCACTCTCAAAGTGTTGTATCATGACAAGGTGGATATTGGAGTAACAGTGCTTACATACTTAAGCTCAACTTCTGATCTTGAAGCCATAGTGTGGGAATCTATAATCACTGCCATTTTGATGCTCACTATTTGTGGTGTAGCAACTGATCACAGAGGGAGAAAAGAACTCGCTGGAGTTGCAATAGGCATTGCCGTTTTGATTAACATCATCATTGCCGGGCCAATTACCGGAGCTTCCATGAATCCTGCAAGGAGTTTAGGCCCTGCTATAGTATCTGGTGATTATAGAAACATTTGGGTTTATATCATAGGCCCAATTTTGGGAGCAGTGTTCGCAAGTACACTTTACAAACTCCTAGATGTAACCAAACCAGCTAAAACTGTACCATTTCACTGGTGTAATCATAATCATTTACCTTTCTAA |
| >CrSIP1;1  ATGTTTGGGGCTATAAAAGCAGCAATTGGAGATGCAGTGTTGACTTTCTTGTGGGTGTTCTGTTCCTCCACGTTGGGGATAGCTGCAGGGGCCATAATCAGAGCCCTTGACGTTCAACACCTCTCTTACAACGGTTTCCCTTACCCTTCTTTTCTCGTCACTACTGCACTCGTCTTCATTCTAGTTTTCTTCTTTACCGTCATCGGCGAGGCCATTGGTGGCGCCAGCTTCAACCCCACCGGCACTGCTTCCTTTTACGCTGTTGGTCTCGGTTCCGACACTCTCTTCTCAATGGCTTTACGTTTCCCTGCCCAGGCGCTTGGTGCTGCGGGTGGTGCATTGGCGATTATGGAGGTGATTCCTACTGAATATAAGCACATGATTGGGGGTCCTTCTTTGAAAGTGGACTTGCATACTGGGGCTGTAGCTGAAGGGCTGTTGACTTTTATTATCACATTTGCTATGCTCTTCATCATTCTTAAGGGTCCTCGTAGTGAGTTACTGAAGACTTGGTTGATGGCCACTGCCACCGTCATTCTGGTCATGGCCGGATCTGCTTACACTGGGCCATCCATGAATCCTGCCAATGCCTTTGGTTGGGCATACTTAAACAATTGGCACAACACATGGGACCAATTCTATGTATACTGGATTTGCCCCTTCACTGGAGCAATATTGGCTGCCTGGCTGTTCCGTGCTATCTTTCCCCCACCACCACCTCAAGTAAAACAGAAGAAAGCATGA |
| >CrSIP1;2  ATGGGGGTGATAAAGTCAGCTATTGGAGATGCAATTTTGACCTCAATATGGGTTTTCATCATCTCAACTTTGAGGATTGTCACAACTGAAGTAGCTATATTTCTTGGTCTTCAACCTTTCTCATTTGCAGGCCTAGTCATCACCACAATCTTTAACACCCTTTATGTCCTCACTATAAGCTTCATTGGTAGGATGTTAGGTGGTGCTAGTTTCAACCCTTCAACCACTATTTCATTCTACACTGCAGGGTTAAGGCCTGATTCATCTCTTGTATCCATGGCTATTAGATTCCCTGCTCAAGCAGCTGGTGGAGCTCTTGGTGCCAAGACTTTGCTGCAAGTGATGCCAACCCATTACAAGCACATGTTGAAAGGACCTTTCTTGAAGGTGGATTTGCATACAGGTGCTATAGCTGAGGGGTTGTTAACTTTTACTCATAATTTAGCTATCCTTTTTATCATGCTCAAGGGTCCTAAAAACCCTTTTCTGAAGGTCTATTTGCTCTCTGTGGCTACTGTGGCTTTGGTTATTCCGGGTTCTGGCTTCACTGGGCCTTCCATGAACCCAGCCAATGCCTTTGGATGGGCTTATACTAACAACAAACACAACACTTTGGAGCAATTTTATGTCTATTGGATATGTCCTTTCATAGGGGCTTCCTCTGCTGCTTTGATTTACCGGTTTCTTTTTATGTCTCCAACTAAGCAGAAGAAAGCTTGA |
| >CrSIP1;3  ATGCAGATCCCAGCCTTTGACACGTATCAACGCTTCATTGGTATGACCTTAGGTGGTGCTAGCTTCAATCCTTCAACCAATATTTCCTTTTACATTGCAAGGCTAAGGCCTAATTCATCACTTGCATCTATGGCTATTAGATTTCCTTCTCAAGCAGCTGGTGGAGCCATTGCTGCCAAGGTGCTCTTGCTAGTGATACCAACCCAATACAAGCACATGTTGAAAGGGCCTTTTTTGAAGGTGTATTTGCTTTCAGTGGCAATAGTGGCTTTGGTCATTCTAGGTTCTGGGTTCACAGGGCCTGCCATGAATCTGGGCTTTGCTTTTGGATGGGCTTATATGAACAATAAGCACAACACTCGGGAGCATTTTTATGTCTATTGGATATGTCCTTTCGTAGGGTCTACCTTAGCTGCTTTTGTATATCGATTTCTGTTTATCTCACCAACCAATAAGAAGAAAGCTTGA |
| >CrSIP2;1  ATGGGACGAATCAAGCTTCTCCTCTTCGATTTCGTTCTATCTTTCATGTGGGTATCTTCTTCCGTTCTCATTCGCATATTTGTCTTCAAATTTCTCGGCTTCCGCCATGACCATCTCGGCGAGATTGTCAAGACAGCCTTCTCCGTCGCCAACATGTTCTTCTTTGCGTTCCTTGTCAGGCTTACACGTGGCGCCGCCCACAACCCCCTTACCGTTCTCGCTGGTGCTATCTCCGGGGACTTTAACAACTTCCTTTACTGTGTTGCTGCTAGAATCCCCTCTCAGGTGCTTGGATCTATTGTTGGAGTTAAACTTCTTATTTATACAATTCCTGAAGTAGGACGGGGACCAAGTCTGAATATTGACATTCATCGGGGTGCACTGACAGAAGGATTGCTAACATTCGCAATTGTAACCATTTCACTTGGACTTGCCACAAAAATTCGTGAAAATTTCTTCATGAAGACTTGGATCTCCAGCCTCTCCAAGCTAACACTTCATATACTTGGCTCTGATCTGACTGGTGGTTGTATGAACCCTGCATCTGTAATGGGATGGGCTTATGCTAGAGGGGATCACATAACAAAGGAACACATCCTTGTATACTGGCTTGCCCCCATAGAAGCAACTATTTTAGCAGTGTGGACGTTCAAATTGCTAGTCCAGCCTGTAAAAGAAGATAGAACAGCCTCAAAAAGAAAATCGGATTGA |
| >CrXIP1;1  ATGGATTTCGCTGATTCCCCAGTAGTTGACATTGATAAACAATTTCCGAGGCCTGTTCAAAATCATGAGGCTAATAAGAAATTTTTAGACTCCAAACTTCTTGACTCAATCGGTGCCCATGAAATTTTCACAAAAGAGATGTGGAAAGCAGCTCTAACAGAGTTAACAGCAACTACATTTCTAGTGTTCACCTTAACAACTTCCATTATTGCATGCTTGGACTCAAATGAGGTTGATCCTAAGCTTCTTGTTCCCTTTGCAGTCTTCATCATTGCTTTCTTGTTCCTAATTGTGACAGTTCCTCTATCTGGGGGTCATATGAGTCCTGTTTTCACAATCATTGCTGCTTTAAAGGGTGTTGTGACTCTTGCACGTGCTCTTATTTACATATTAGCACAATGTATTGGCTCAATAATTGGTTTCTTAATACTTAACAGTGTAATGGACCCAAAATTAGCTGATACATATTCCTTGGGAGGCTGTGCCATTAGTGGCAAAGATGTGAATTCTGGTATAAAGCCTATGGATGCTTTGATATTGGAATTCACTTGCACATTTGTGGTACTCTTTGTGGGTGTCACATTGGCATTTGATAAGAAAAGGTCAAAGGATTTGGGCTTACCAATTGTATGTTTGGTGGTGGCAGGGGCCATGGCACTGGCAGTTTTTGTGTCCATAACTGTAACTGGGCGGGCTGGCTATGCAGGTGTTGGGTTGAACCCAGCAAGATGTTTAGGCCCAGCATTGTTTCGTGGAGGCCCATTGTGGGAGGGTGTGGTTTGGGTGGATGGACAATATGATGTGTTAAAGTTGGCTTTCGGTTCCAGTGGGACCATCCATAATAACAATGGTGTTACAAATGATCAAACAGAATGCCAAGCTCAAGTATGA |
| **Genomic DNA** |
| >CrPIP1;1  ATGGAAGGTAAAGAAGAGGATGTGAGAGTTGGTGCCAATAGATACAGAGAGAGACAACCAATAGGAACTGCTGCTCAAGCCCAAGATGATGCCAAAGACTACAAAGAGCCACCTTCAGCACCTCTTTTTGAGCCTGGTGAGCTATCATCATGGTCTTTCTATAGGGCAGGGATTGCAGAGTTTGTGGCAACGTTTTTGTTTCTTTATATCACGGTGTTAACTGTTATGGGTGTGGCCAAATCCAATTCCAAGTGTTCCACTGTGGGGATTCAAGGCATAGCTTGGGCTTTTGGTGGAATGATCTTTGCTCTTGTTTATTGCACTGCTGGGATCTCAGGTAAAATTTTAACACTCATGTTTTATCTTTCTATAATTTCTTCTAGGTAATCATTTTAATATTTTATTATCAAAAACAAATAAATATTTTGTAATCTAAAAGGGTCTCCGTCTTGCTGTTTATTAAAAAAGAAAATAAAAAAATTTCTATTAGAAATCATGATGAGTTTATTATAAAATATAAATCACAGAAGAAGTACGTTAGTAAAATTTTTAATATAAGTTTATAATTTTTAATTAGTATTTTTATAATATTGAGTGGCCTTTTTAATTAAAAAAAAGTTGCTTTATTATACTGATTAAGATATTAAAAACTAAAAGAAATTATTAATATAATTTTAATTAAAAATTTACTTTTAATTCTTTAATCAGTGCTTTAGATTCATGTTAACAAGATCTTATAAAAATATGTTTTGACATATTTTATTTTTCATTCTAAGTAAGGATATATTGTACAATGATTTAGAATTATTCAAATTCAAATGCAACTGATAAATGAAATGGGTACCAACGTCTTCCAATTTGGAAGTAGCCCCAAAATCTCTAATACGAGATCAAGGGAAAATTTTATATGATAAATTTTATATTTGTTTCTCTTTTAAATAACATTAAATAGGTAAAAATTATCAAAATTTGTGAAAATGTAAAGAATTAATATTATTTTTTTATATCTTCTTTCCTATCACACAATTATATTTTTCTTTTTTTTCTTCCTACATCTCTTTCTTTTAATCACATCCCATTAACAATAATTTTTTTAATATATATAACTTGTGTGAATTTGGTAATGCAGGGGGTCACATTAACCCAGCAGTGACATTTGGGCTATTCTTGGCACGCAAGCTCTCTCTGACAAGGGCAATTTTTTACATAATTATGCAGTGCTTGGGGGCTATATGTGGGGCTGGAGTAGTGAAGGGGTTCCAACCCCACCAATATGAAAGGCTTGGTGGTGGTGCCAACACACTCAGTAAAGGCTACTCCAAAAGTACTGGCCTTGGAGCAGAGATTGTTGGCACATTTGTCCTTGTTTACACTGTCTTCTCTGCCACTGATGCAAAAAGAAATGCTAGAGATTCCCATGTTCCTGTAAGTATATATAAATCATTCCAAATATGCACCTTTTTAATTATAGTTATATCAGTATTATTATAATGACTTGTAAGTTGTGTTATTTTGATAATAAATTTGATTATAAACTTCTTAATGGGTCAGATTTTGGCACCACTGCCTATTGGTTTTGCTGTCTTTTTGGTGCACTTGGCTACAATTCCTATTACAGGAACTGGTATCAACCCTGCTAGAAGTCTAGGTGCAGCCCTTATATACAACAAGGACCAAGCTTGGGACAACCATGTGAGTCTTCGTTCTTAATTTTTTTTTCTTTTTCTTTATTCTTTCCTAATTTTACATGTGCAAATTAACAGCATTATTTCTAGCATATTTTTTTAAAAGAATATAACACCCATATAAGTAAGTCATAATAAATTTCAGTTAATAATAAAATATATTTGAAAATATGTAATAAAATTTGTTACTAATATCCTAAATCTATTTTGACTGCATTTGAACAATTAGATTAATTAAGCACTTATTCAATAATTTTTTTTATGTAAGAGCTTATAACATAAGTTTGATCATAAAATTGTTATTAAAATATGTTAAAAAATTATTTTATTAAATTACATTAAAATAAAAAATTAAACTTATAAACTATTGATACAAATTTCTTGCAAGTCCTACCACACCTTGATCTATAGCATTTGTAGATGATAGCAATTGACTTTTGCTTGGTGAAAAAAAGTTAAAATTTCCCAAATTATATTATTTTGGTGCAGTGGATTTTTTGGGTAGGGCCTTTCATTGGAGCAGCACTTGCAGCATTGTACCATCAGATAGTGATCAGGGCCATCCCCTTCAGGTCCAAGTGA |
| >CrPIP1;2  ATGGAGAGGAATGATGAAGATGAGAGAGTTGGTGCCAACAGGTATGGAGAGAGGCAACCAATAGGAACTGGTGCACAGAGCCAAGATGGGAAAGAGTACAGAGAAGCAGCTCCAGCTCCATTGTTTGAAGCCAGAGAATTAACATCATGGTCTTTCTACAGAGCAGGGATAGCAGAATTTGTGGCCACATTCTTGTTTCTTTATATAACAGTTTTGACACTGATGGGTGTTGCAAAATCTCCCACAAAGTGCTCCACAGTGGGTGTTCAAGGCATTGCTTGGTCATTTGGTGGAATGATCTTTGCTCTTGTCTATTGCACAGCTGGTATCTCAGGTAATAGAAATTATATTATTAATTAATACAAAATATCATGTCTCTTTTAAGTATATGATACTAATAGGCTCTATATATCAACAGCTTTTCACTAACAAAATTATTTATTAGAATTAAACATATTTTTAATCCATGTAAAATTAGTAAATTTTTTAACCCTTATATTTAATTAAACATATTTTATAAAAATTACATATAAATAAAATTTAATTAAATATCAAAATAAAGACCAGAAATATATTTAACTTAATTTATTAATATGCATCAGATTACTAATGTAAAGAAGTGTAGATCCATCATATCAACCAAAAATAAGCTTAATGTGTAATATTTTTGTTTTGATTAATCTATCTATTATGAGATGGTTATTGAGTGTTGTGTAATTGTTTGAAATGCAGGGGGTCACATTAACCCAGCTGTGACATTTGGGCTGTTCTTGGCACGCAAGTTATCTCTGACTAGGACAGTGTTTTACATGATTATGCAGTGTTTGGGAGCTATATGTGGTGCAGCTGTAGTCAAAGGATTCCAATCAAAACAATATGAGAGGCTTGGTGGTGCTGCCAACACTCTAAATAAAGGGTACTCCAAAGGTGATGGCCTTGGAGCAGAGATTGTTGGCACATTTATTCTTGTTTACACTGTTTTCTCTGCTACAGATGCCAAGCGAAATGCTAGAGACTCACACGTTCCTGTATGTAAACTCTTTCCTAATTTGCATCAATTTTGAAATGTTTAAAAACTGAATTATTCTGATATATTATAATCTAATCATACACATTTCAATGTGTTAGATTTTGGCACCATTGCCTATTGGTTTTGCTGTCTTTCTAGTGCATTTGGCTACAATTCCTATCACAGGGACTGGCATCAACCCTGCTAGAAGTTTAGGTGCAGCCATTGTATACAACAAGGACCAAGCTTGGGATGGCCATGTGAGAATTCATCTTAATTTTACTTGGGATTTTTTCTTTTGGTTACAATTTTTTGAAATTGATAACTGTTTGTGATGGTTAATAAACTGACAAAAATATAGATGTAAATGGATGAATGGTATTGTGCATATCTAAAATAATTGAAATGTTTCTTAATTTATATAAGTCCAAGTTTTTTAAATTTTTACAGAAAAAATAAATCAAACGATTTAGTGGACTTATGCAAATCGTATTTTATTTTGGTGCAGTGGATCTTCTGGGTAGGGCCTTTTATTGGGGCAGCACTTGCAGCTTTGTACCATCAGATAGTGATGAGGGCCATTCCTTTCAAGTCAAAATGA |
| >CrPIP1;3  ATGGAGAAAGAGGAAGATGTTAAGGTTGGAGCAAACAAATTCTCAGAAAGGCAACCATTGGGAACAGCTGCACAGAGTGACAAGGACTACAAAGAGCCACCAGCAGCTCCTTTGTTTGAGCCTGGTGAGTTAAAGTCATGGTCCTTCTACAGAGCTGGAATTGCTGAGTTTGTGGCCACTTTCTTGTTCCTCTACATCACCATTTTGACTGTTATGGGTGTCAACAATTCATCCTCCAAGTGTTCTTCTGTTGGCATTCAAGGCATTGCTTGGGCTTTTGGTGGCATGATATTTGCCCTTGTCTACTGCACTGCTGGAATATCAGGTATATTATTATGTTATATATAACTTTCAATTTTCATAATATTTTTTAATACCCTAAGTAACATAGATGTGTGTGTTGGTTGTAACAGGAGGACACATAAACCCAGCTGTGACCTTTGGTCTATTTTTGGCTAGGAAGCTGTCCTTGACAAGGGCAGTGTTTTACATTGTGATGCAGTGTCTTGGAGCTATATGTGGTGCTGGTGTGGTTAAGGGCTTTGAGGGTAATGCTCGGTATGAGTTGTTCAAAGGTGGAGCCAATTTTGTGAACCCTGGATACACCAAAGGTGATGGCCTTGGAGCTGAGATTGTTGGTACCTTCATTCTTGTCTACACCGTTTTCTCTGCCACTGATGCTAAGAGAAACGCTAGAGACTCTCATGTTCCTGTATGTCTTCTTCTTCTTTCCATTAATTTGCCATTTCCTCATGTTTACCAAAAATATGAGTTTCTCTAATAAATTCACCTATATCTATGAATTATGGTGTGAGAAATATAACAAAAAAATTGTGTACTTGTTTTAATGTTTCTGGTAAATGGATATTCCAATTAATTAATTAATGTGGTGAATTTATGATATAACATGATTGTAGATTTTGGCTCCTCTTCCCATTGGATTTGCTGTGTTTTTGGTCCATTTGGCCACCATTCCCATCACTGGAACCGGCATTAACCCAGCTAGAAGTCTTGGAGCTGCCATCATCTTCAACAGGGACCTTGCATGGGATGACCATGTATTTTCTTAATCTTCACTTTCAATTTTGTTGTATTTCCATTACCCTTGCTACAAAAATAAATAATAATACAATTTTTAATTTTAATGTTTTGCAGTGGATTTTCTGGGTTGGACCTTTTGTTGGAGCTGCCCTTGCTGCTTTATATCACCAGATAGTCATCCGAGCCATTCCTTTCAAGACAAGGGCTTAA |
| >CrPIP1;4  ATGGAGAGGGATGAAGATGTTAAGGTTGGAGCAAACAAATACTCAGAAAGACAAGCGTTGGGGACAGGAGCTCAGGGTGAGAAGGACTATAAGGAAGCACCCCCAGCACCATTGTTTGAGCCAGGGGAGTTGAAGTCATGGTCTTTTTACAGAGCTGGGATTGCAGAATTTGTGGCAACGTTCCTGTTCTTGTACATCACAGTCTTAACTGTGATGGGTGTGAACAGGTCACCAAGCAAATGCTCCTCTGTTGGCATTCAAGGAATTGCTTGGGCTTTTGGTGGCATGATTTTTGCACTTGTTTACTGCACAGCTGGAATATCAGGTAATAAACATATCCTTCTTTCATATTTCATGTTAAAATTAAATGTTATTTTTCTTTTGTGATGTTGGTGTTGGTGTTGGTTGGCACAGGTGGACACATAAATCCAGCAGTGACCTTTGGTCTGTTTCTGGCTAGGAAGCTGTCACTCACAAGAGCGGTATTCTACATTGTGATGCAGTGCCTTGGAGCTATATGTGGTGCGGGTGTGGTGAAGGGGTTTGAGGGTAATGGTAGGTATGAGATGTACAAAGGTGGAGCTAATTTTGTGAGTCATGGATACACCAAAGGTGATGGTCTTGGAGCTGAGATTCTTGGCACTTTTATTCTTGTTTACACCGTTTTCTCCGCCACCGATGCCAAGAGAAACGCCAGAGACTCTCATGTTCCGGTATCTATTCTTTCTCCTCTCTTCTCACTACTTTGCATAGTGAATAACCATACAATATAATATATACTTGCAATTAATTTTAGATCCTGGCCCCTCTTCCTATCGGATTTGCTGTTTTCTTGGTCCACTTGGCCACCATTCCGATCACCGGAACCGGCATTAACCCGGCCAGGAGTCTAGGTGCTGCTATAATATACAACAGAGACCATGCTTGGGATGACCATGTATGTACTTCTTTCTCCTTATGTGGCTTTAGAGTTAGTATTTTTTTTTATGCTTAAAATTAATAAAATGACTTATTTATAATTTTGTGGGTTACAGTGGATTTTCTGGGTTGGACCCTTCATTGGAGCTGCCCTTGCTGCTTTATATCACCAGATCGTGATCCGAGCAATTCCTTTCAAGACAAGGGGTTAATCCTCCTTCGTTATGAATTATCTACCCGGCCTGCTCTTTTTGTCGCTTCATATGTAATATCTATTTGTTTTACTCCATCATGTGTGTAA |
| >CrPIP1;5  ATGGAGGGGAAGGAACAGGATGTTTCATTGGGAGCCAACAAGTTCCCGGAGAGGCAGCCTATTGGAACGGCGGCGCAAAGCCAGGACGACGGAAAGGACTACCAGGAGCCACCACCGGCGCCGCTGTTTGAACCGTCCGAGCTTACATCGTGGTCGTTTTACAGAGCCGGGATAGCAGAGTTCGTCGCCACTTTTCTTTTTCTCTACATCACCATCTTAACTGTGATGGGCGTGAACAGGTCCCCCAAGTGCCAGTCAGTTGGTATTCAAGGAATTGCTTGGGCTTTCGGTGGCATGATCTTCGCTCTTGTTTACTGCACCGCTGGAATCTCAGGTGAAATCCCTCTCTGGTTTTGATGAAATAAATTCAAATTAAATTATTGAAAATTAACAAGGTTGGGTGCGATGCAGGGGGTCACATAAATCCGGCGGTGACGTTTGGACTGTTTTTGGCGAGAAAATTGTCGTTGACAAGAGCGCTGTTCTACATGGTGATGCAGGTGCTGGGTGCTATCTGTGGTGCCGGTGTGGTGAAAGGTTTCGAGGGAAAAACCAGGTACGGATTATACAAAGGTGGTGCCAACTTTGTTGCTCCCGGTTATACCAAAGGTGATGGCCTTGGTGCTGAGATTGTTGGCACCTTTATTCTTGTTTACACCGTCTTCTCAGCCACCGATGCTAAGCGTAGCGCCAGAGACTCTCACGTTCCTGTAAGTCCCCCACTCCTCTATGTATCTATCGATCTTAAATTAGTCTTTAAAATTATACGGTAATTTACTTATTAAAAATGTTTGTTGAACTAACTCTATGGTTTGAATGAGTAGATTTTGGCACCTCTACCCATTGGGTTCGCTGTTTTCTTGGTGCACTTGGCCACCATTCCTATTACCGGAACTGGTATCAACCCTGCTCGTAGTCTTGGAGCCTCCATCATCTTCAACAAAGACCTTGGTTGGGATGATCACGTACGTTAATGATTATTAATTAATTAATTAATTAATTCCTCTTTTAGATAATATTTTAGAATTTTCCAGAAAATGAATCGCAACTGTTGATTCTAGTGTTAATTGATTGCCACTATTTGTGCAGTGGATCTTCTGGGTGGGACCATTCATTGGTGCTGCTCTTGCCGCTCTCTACCACCAAGTCGTCATCCGAGCCATTCCTTTCAAGTCCAAGTAA |
| >CrPIP2;1  ATGTCGAAGGAAGTGAGCGAAGAAGGACAGCAAAGGAAGGACTACGTGGACCCTCCTCCAGCACCTCTTATCGACTTGGCTGAGATTAAGCTCTGGTCCTTCTACAGAGCCCTCATCGCCGAGTTCATCGCCACCCTTCTCTTCCTCTACGTCACCGTCGCCACCGTCATAGGCCACAAGAAACAGAGCGGTCCATGCGACGGCGTTGGCCTTCTCGGCATAGCTTGGTCCTTCGGTGGCATGATCTTTGTCCTCGTCTACTGCACAGCTGGCATCTCCGGTAACCCCAACTAACTAACTAACTAACTCCCTCATTCCCTACGTTCATTGTGCTTTTAAATAAACTTTTTCAACATTCTGAAGCGAACAAACAAATGCAGGTGGACATATAAATCCTGCGGTGACTTTTGGGCTGTTCTTGGCTCGGAAGGTGTCTCTCATACGAGCTGTGCTGTACATGGTAGCACAGTGTCTTGGTGCTATCTGCGGTGTGGGGTTGGTGAAGGCCTTCATGAAGCATCCCTACAACTCTCTTGGTGGCGGTGCTAACTCCGTGTCTTCTGGGTACAACAAAGGCACGGCTCTTGGCGCTGAGATAATCGGGACATTTGTGCTTGTCTACACCGTTTTCTCCGCCACAGACCCCAAGAGAAACGCCCGTGACTCCCATGTCCCTGTATGTTTCTATTATATAATCACATTCATTCATTCCACCACTTGGGCCTTAATTGGCTGATAATTTACAGGTTTTGGCCCCGTTGCCAATTGGCTTTGCCGTTTTCATGGTTCACCTGGCTACCATACCCATCACCGGTACCGGAATTAACCCCGCCAGGAGCTTCGGTGCTGCTGTTATCTACAACAACTCCAAAGTTTGGGATGACCATGTATGTATACGTACATGTATATGTATATATATACATGCCTTTTTGTTTCTTTCTAGCTTCGAATCTGATAGATTATATATTATATGCGGTGCATGGCTGTATTATCTGATGTGTGTGTGGTTGCAGTGGATCTTCTGGGTTGGGCCCTTCGTGGGAGCGTTGGCAGCAGCTGCTTATCACCAGTACATACTTAGAGCAGCAGCTATCAAGGCATTGGGATCGTTCCGAAGCAACCCAACCAACTAG |
| >CrPIP2;2  ATGGCTAAAGAAGTTGAGGTTCAAGAACAAGGAGAATACTCAGCTAAGGACTATCAAGACCCACCTCCAACACCTTTGTTTGATCCTGAGGAGCTCACAAAGTGGTCCTTCTATAGAGCCCTCATTGCTGAGTTCATAGCAACCCTTCTCTTCCTTTATGTCACTGTGTTAACCATTATTGGCTACAAAAGCCAAACTGATTCCACCAAAGGTGGCACAGAGTGTGATGGGGTTGGCATTTTGGGCATAGCTTGGGCCTTCGGTGGCATGATTTTCATCCTTGTTTACTGCACTGCTGGTATTTCTGGTATGATTTTTGTCCCCTTTTCTCTCTTACAAAACCACAACATACTCTTCATGTATTATATAGCTACAAACATGAAACACACACTATAATATTCGGAATAATTTTTTTAATAAATTTGGAAGTTAATTTTATGAAATAGTGTGTAATTAAGTTGGTTCCCTTGGCTTGTGGCATGACCTTCCAAAAGTTGTAAGTAATATGCATGAAAGAAGAAATTAAAAGTTAATAATTTGTGGGCTTGGTGATGCAGGAGGACACATAAACCCGGCTGTGACATTTGGGCTTTTCCTAGGACGCAAGGTGTCACTGATAAGGGCGTTGCTATACATGGTAGCACAGTGTGCTGGTGCAATCTGTGGCACTGGATTGGCAAAGGGGTTCCAAAAAGCATACTACAACAGGTATGGAGGTGGTGCCAATTCTGTGGCTGATGGCTACAATAAGGGTACTGCTTTAGGTGCTGAGATTATTGGTACCTTTGTTCTTGTCTACACTGTCTTCTCTGCCACTGATCCTAAGAGGAATGCTAGGGACTCTCATGTTCCTGTAAGAATATATATATATTTACCATATTCCAAAATATATATATATATATATATATTTTTTTGTTCATCATAAATATATATAAATACACCACCTCCTCTCTCATTTTTCTAATTATTCTCTTGTTTGGTTTATGGTTGCATGGATTGTTAAAAAAAATATAGGTACTGGCACCACTACCCATTGGATTTGCTGTGTTCATGGTTCACTTAGCAACAATCCCAATCACTGGTACTGGCATTAACCCTGCAAGGAGTTTCGGAGCAGCTGTAATATACAACAAGGAGAAAATTTGGGATGACCAGGTAAATATCCATTTATAAACTAAATTCATGCTTTGTTTCCTTTTTTTTTCACATGTAAACTATTCTAACCATTAAGTATTAAGATAATTTTAGGGAATTGTTTATATTTTTTTTAAATTACCAATTTTTGTTAATTTATTGACAAAATATTTAAAAGAAACTATTTTCTAAAATAAATGGTTTTCAAACATGAATTATTAAATTAGTTTGTTAGTTTAGTCTTGAAAATTTTAAAAATAATTTATGATTATTATTTTGCATTAAAAAGAAATATAACAAATGTTATTTTGCAAATCTCGAGAACTAAAATATCATAAGTTATAATGTTTGGTCCATACAATATGATAGACTACTCAAAATTTGGTTTTTTTTATTATTCAATTTTTACCAAAGAAAAGAAAAATGATGCTGAAAGTTATCTATATATTTGATAAGGGGTCTAATTTTGATGAATTGTTTGATGAACAGTGGATTTTCTGGGTTGGACCAATTGTTGGAGCAGCAGTGGCTGCAATCTACCACCAATACATTCTTAGAGCATCAGCTATCAAAGCTCTTGGATCCTTCAGGAGCAATGCTTAA |
| >CrPIP2;3  ATGGCTAAAGACGTTGAGCAGGTTACGGAGCAACAAGGGGAATACTCGGCCAAGGACTACCACGACCCTCCTCCGGCGCCGTTGATCGACGTGGAGGAGCTCACAAAGTGGTCTTTATACAGAGCCGCCATAGCAGAGTTCATAGCAACCCTTCTCTTCCTTTACATCACCGTCTTGACCATTATCGGGTACATGAGACAGAGCGATACCACAATTCAAGGTAACACCGAATGTGACGGTGTTGGCATTTTGGGCATTGCTTGGGCCTTTGGTGGCATGATTTTCATCCTTGTTTACTGCACCGCCGGTATCTCAGGTAACTAACTATATATACACCTTCCTACTACTATTAATTAATTAATTACATACTTAATTATTACTAATGAAATTTGTATGTTATAGGTGGACACATAAACCCTGCGGTGACATTCGGGTTGTTCGTGGGACGCAAGGTGTCTCTGATAAGAGCGTTACTTTACATGATAGCACAGTGTGCGGGTGCTATATGCGGTGCTGGATTGGCGAAGGGATTCCAAAAAGCATACTACAACAGGTATGGAGGAGGGGTTAACCTTGTGAGCGATGGTTACAACAAAGGTACTGCTTTGGGTGCTGAGATCATTGGTACCTTCGTTCTTGTTTACACTGTTTTCTCCGCCACCGATCCTAAGAGGAGCGCCAGAGACTCTCATGTTCCTGTAAGCATTCATTTGTTTGTTTGTTTTTTTTTTTTTTCATTTATAATTAATGATGAATTTTGAAATAATAAAAAATAAAAACAGGTATTGGCACCACTTCCTATTGGATTTGCGGTGTTCATGGTCCACTTGGCTACAATTCCTGTGACCGGTACCGGTATTAACCCTGCAAGGAGTTTCGGACCCGCTGTTATCTTCAACAAGGACAAAGCCTGGGATGACCAGGTAACTAACTGCATATACATACATATATATATAAATAAGGACTTAAAATATTTGTAAAGGAAAGTTAGAATAAGTTATTGTTTTGACTTTTCAGTGGATTTACTGGGTTGGACCATTTGTTGGAGCTGCTGTGGCTGCATTCTATCACCAATACATTCTGAGAGCAGCAGCTATCAAAGCTCTTGGATCCTTCAGGAGCAACGCTTCATGA |
| >CrPIP2;4  ATGGCTAAGCATGATGTTGAGATTGCTGAGCGTGGTTCCTTTTCTGCAAAGGATTATCATGACCCTCCTCCAGCACCTTTGATTGATGCTGAGGAACTCACAAAGTGGTCCTTCTACAGGGCTTTGATTGCTGAGTTTATTGCCACACTTCTCTTCCTTTACATTACTGTCCTCACTGTGATTGGATACAAAAGCCAGAGTGATCTCAAAGCTGGTGGTGATGTTTGTGGTGGTGTTGGCATTCTTGGCATTGCTTGGGCCTTTGGTGGCATGATCTTCATCCTTGTTTACTGCACTGCTGGAATTTCAGGTGCTTTTCACTTTTCATCTTCTTCACATACACATTCTTTATGTAATTACTTCATTTCTGATCAAATTAGTTTTGAATTCTTGAACATATTTATGATACGTGTATAATTAATTTGAATCATATATTTTTACTAGAGTGCGTGTTTTTCATTAGTAAGTGTGAAAAAGTCTTAATTTGCTTGAATGGACTTCAATCTCTTTTGTTTGTTGTTGTTCAAAGTTCTCATTTTTCTCTAATCTTATACATTATTTGATTGATTTCCTTCTTTCTGATATAGTTTCTTAGCATTCAAACAAGAAAAGTTTGCTCTGTTTAAGAAGCATCAAGGGTACCCCCATATAGCCAATTAGCTAGCATGTTTGGTTTTAAGTGTTTATCAAATGATATAATGATGCTCTTAGTTCAAGTTTAAGTGGCCATTCTTGTTTAATTTACATAATTCTCTCAATTACTCCGCCTACTGCTTAGTTGCTTCACTTCAAATTGAGGATGATGGTTTGGTGTGTTTGGACTTGTTTTCTTGGTTGAAAAGTAAAAAAACATATACATAAAAAAAAAAAAAAAAAACCCTTGATTTGCTAGCAACTTTATGCCTCTCAGTTCTAATTATATGAAAATTGTAACTTTTTTTAAAAATTTAACGCAGGGGGTCACATAAACCCAGCAGTGACATTTGGGCTGTTCTTGGCTCGCAAGGTGTCTTTGATTAGAGCTATAATGTACATGGTGGCTCAGTGCTTGGGGGCCATATGTGGAGTTGGGTTGGTTAAGGCCTTCCAAAAGGCTTATTACAATAGGTATGGTGGTGGGGCCAATGAACTCAGTGATGGGTACAGCACAGGTGTTGGATTGGGTGCTGAGATCATTGGAACCTTTGTTTTGGTATACACTGTATTCTCTGCCACTGACCCCAAGAGAAGTGCTAGAGATTCTCATGTGCCGGTATGAATTCAATCATATATTCATTGTATTATAGTCTTGTTTGTGCTTTTTTGGTTCTCTTATTAATACATTAATTTAATACTCTATGTACCAAGTTACCAACTGTGTTGTCATACCCTATTTTTAAGCATGGATCACTCTTAGTTATTTCAGGTTTTTAAATAGAAAATTAATTTCGAGAAATAATTTTAGAATGGTTATTGGTAAAGAAAAAAAAAATTGTGCTTCTTGTAACCATAAACAGATAAAGTTGAGGGGAAGTGACTAAGCTATTTTCAGCTATCTCAACTATCATGCATCTAGTTTTACTTTTTAAATGAAATTAGGTAAAGGGGCATTGAAGTAATTAAGAAGCTTTAATAAACTTTTTCTATTGTCATGGATAATTAATTGTAGTTCTTCAAATGGTGATGGCATGCTATCAGTCTAATCATTCACGCCAATTGGATATTTCTAAGTCAGTGAACTTCATAATTATTTTAAAAAAAATCCATTCATTTCTGATGATATGTACATGCTATCAAAAATTTTACAGCTTATGCCATCCTGCAGTTTCCATTTTCATAAAATGTTCTAGCCCAGTCAATATGTGCTTAATAACAATAGTAATAACACCTTTATTTTTAATAATAATTTTATAATAATCTGAACTAGTCAATCCTTCATGAAACAGTTTAAAGATCTTAAGTCTTAGCCAGTTGGTTTTCTTTTAATATTGGAATCTAAATCTTCTTTTCAGTACTTTATCTTGAGACTTCAATATTGAACATGAAATTTTCCACTTTTCTTGTTCCTTGCTTTCTACTACTACTTTAATTGTTTTGTCCTCAAAACAAGAAAGTGGGTAACCTTTTCATGTCATAATGGTTACATCTTTTATTCATCTAGGCATCCTAAAGGGGTGAACCTATTTCTTTCTTTTCACCAAAATTACCAAATACGTGATAGTTTTTTAGTAAAGAAAAATGTTACCATGCGCTTAAGGAATATATATGGACCCATTACCATATATGATTTTGTGGTGACTTTTGATTTTTCTCATAGCCTTATCATAGGAATGTTATCTAATCTTTTTCCTCTTTCAAGAAAAGTGGATTCTTCTGAAGTTTTCATGCATTTTTTTTTTTTGCCTTGCAAGCAAAATGCATCTTTGGTAGTGAATAACTTTGTGTGTTTTAAGGTCAAAAATTAATGCTTAATATCATTTTCTCCCAAGCTGGCAAATCTCGTGAGAGTTTCCAGCATGGGTCATGCTCATTTGTCATTATCAGTTTTGGGTTCCTTCAAACACTTTCTCAGATTCTCCTAATTAATAAAGAAAAGAAACCATGAGCCTCACATGGAAGCATGTCCCATCTGTCCCAAGTGTTTATTGTTTTTCAATTTTTCTTTTTCTTTTTTATTTTTATAATGATTAATTTTAATTTGTATGTTTGGCATAACTTGTGATGGACTTTTTTTTAATTAAAATACAAGTTTTTATTTTGATTTTGTTGTGTATTTGAAGGTTTTGGCTCCACTTCCAATTGGATTTGCTGTGTTCATGGTTCATTTGGCAACCATCCCAGTGACTGGCACTGGTATTAATCCTGCTAGGAGTCTTGGAGCTGCTGTCATCTACAACCAAGAGAAGGCATGGGATGATCAAGTGAGTAACTATTTTCTAAAACTATTACATTTTTATATTATAAGAAATATTATTAAATATTAAAAATTATAAAATAGAAAAATAAATAAAAACCCATGTTACTTCCCTCTTCATTCCTCTCACTTTAGAGGAAAGATATGGAAAATATTAAAAATCATTTGAGTTATGAGAATATTTGTTGTTTTTATTTTTTTATTTATAAAATTATCATACCATATTATTTTTATTTTTATAGAAATAAGCTATTTTTAATGTTTTGTGTACAAAATTTTAAAAAAAGGAAACAAAATAAAAATAAAATAAATTTACAAATCAAATAATACCTTAGCAAAATATTTTTTTAAACATATTTTTTAATATATATTCTTTTATGGTTGATCAGTTTTATGTCAAAACACATATCTTATAAATTGATTATTTTTTAAAATTACTTCATTTTTCAAAAAAAAAATGACTTGCTAGTTTTTTTTTTATTATAATCAAATTTATATTTGCCATATCAAGCAAAGGAGGATAAAAAAAATGGCAACTTTTATCTTATATTTTGTTCCTTCTCTATGTTCAGTGGATCTTTTGGGTAGGACCATTTGCTGGTGCAGCCATTGCAGCCTTCTACCACCAATTCATCTTGAGAGCAGGTGCAGCTAAGGCTCTTGGATCATTCAGGAGTAACCCCACTGTTTGA |
| >CrPIP2;5  ATGGCGAAAGACGTTGAGGTTACTGAGCGTGGTTCATTCTCTGGCAAGGACTACCATGACCCTCCTCCTGCACCCCTCATTGATGCAGAGGAACTCACCAAGTGGTCCTTTTACAGGGCACTCATTGCTGAGTTCATTGCCACTTTGCTTTTCCTTTACATTACTGTGCTCACCGTTATTGGGTACAAGCACCAGAGTGATGTTAACGATAAGGGTGATGTTTGTGGTGGCGTTGGCATTCTCGGAATTGCATGGGCCTTTGGTGGCATGATCTTCATCCTTGTTTACTGCACCGCTGGAATTTCAGGTTACTTTTCTCTCTTCTTTTCCTTCCTTCAGATCCACTTCCTTCAATGCTAAAATCACACAACTTGTTGTTTCTTGTTTCTGCCTTTCCATTGCTTGCTTTGCAATTTTTTTTTTTTTACCAAACTTCCTGATTTCTTGTGTTGTGTTGGGTGTCTGAAAAAATGTTTCCTGAAGAAGATTTTTTGGTTGAGTTTTTGTGTTTGATTTTAAGTTACTTATCCTCCCAGTAGAAAAAACTTGCACTGTGGGAACCTCCTTAGCTTGGTTTTTCTTTTTCCTCTTTTTTTTTTTTCTCTTAAGTTTCTAGGGGCGTGTTTGTTTTGTTTATCCGTTTCTGTTTTTATTTCCTGTTTTTAGTTTTAATTACAAAGATGTATTTTTAAAAAAAAATTGTAAAGAGAAAAGCCAAGATCGAAAATGGATGAAGATAATGACTTTTTAGACACACCAAACATGTCCTAAGCCTCCAACCGTTGTTTGAGTATTTGTTTAGAGTTACTAGTATGGAAGATTTAACTCATTAACTGTGGTGGACTAAGTTTGAAGAATTTGTGGGGTTGCAGGAGGTCACATTAACCCAGCAGTGACATTTGGGTTGTTTTTGGCTCGCAAGGTGTCTTTGATCCGTGCTATCATGTACATAATAGCTCAGTGCTTGGGGGCTATCTGTGGAGTTGGGTTGGTCAAGGCCTTCCAAAAGTCTTACTACAACAGGTATGGTGGTGGGGCCAATTCTCTTGCTGATGGCTACAGCACAGGCGTTGGATTGGGTGCTGAGATCATTGGCACCTTTGTTTTGGTATACACTGTCTTCTCTGCCACTGACCCTAAGAGGAGTGCTAGAGATTCCCATGTTCCGGTACGTTTTCAATCATTTACCTTATTATTACATTCTCTTCTCTTCCATGTATCAACTCTGCAACAACACCATGTTGATGCCTGAGTTTTGACGAAATTACTATGTGTGGAGCAACTAGGGGGAGGCATAGGTATTATGAAATGTTCCAAGTTAACCAGTTTCATCTGGTTTAGTCCTTTGGTTAAACATTGATTCCCTTTTAGTTTATGTTGTTTATAAAATTTGGTAAATAGTTTTCAACTGTGATCTCGAATTACCTATTGATTGAAGAAAGAAAAAGAAAATGGTGTTTCAATAATAGATATGTGTATTTTAGGTAGGGTTCAATATCCATGATTTTGCCCCCACTTTTCTTTGCTTGGCTTATTTAGTCAACTTCTTAAGGTTAGATTTCAGAATACGTAAGTGCCTTAATTAATTTCTCAGTGGATGCATCTTTGATTTGTCTAGGCATCCTAGGGTGGTGAAGCTATCTTTTTCCTTCACCATAATTATTTCCAAATGAAATGATGATGTTAATGAAGTATAACAGTGGCGTGCGCTTGTGGAAAATGGGTCCCTTTTACCTTGTATATGAGTTTGTGGTGGTGGTTGAATTTCATGCCCAACACATGATTATTTGGATATATAATTTCTTCCCAATTTTGAGAAAAGTGGATTCTACTTTGTTCCTTGAAAGTAAACCATATCTATGTTATCTGATAGTAGAGGACTTGGAGTGTCTCAAGTTTAAGATTAAAGTGTTCATAATTATGTCTGTTATGCCTATGTCATTTTCCAAAAGCTGGCAAAATCTGGTGGGAATTTCTAGCCTCATGGCAATTTGTCAATATCAAGCTTGGGTTTTTGTATTCACCTTCCAAATACCTTTAACAGATTATTGTAATTGAAAAAGGTAAAATATGGGCTTCACATGTATGCATGCCCTATCTGTCCCAACTCAATTGTCTATTGTTTTGTCACTTCCATTTACTTTTTCTAATAATTTGGATTCATGCGTTTGGAATAACTTTGCATCAACAGCATCTACTTTAATTTAGTTCAATCACATCATTTTCTTATCATGCTTTATTCTATTCTGCATTTGAAGGTTTTGGCACCACTTCCCATTGGATTTGCTGTGTTCATGGTTCACTTGGCCACCATCCCAGTCACCGGCACCGGCATTAATCCTGCCAGGAGTTTTGGAGCTGCTGTTATATACAACCATGATAAACCCTGGGATGACCATGTAAGTGTTGTTACTATCTCTAAATTTACAACCTGGTTATGTCAAGTTGTTAAAAAGACTATTAGAAGTGAATATTATAGCTTTATCTGATAAATTGTTCATTCTGTTTGTTCAGTGGATCTTTTGGGTTGGACCATTTGCCGGAGCAGCCATTGCAGCCTTCTACCACCAGTTCATCTTAAGAGCAGGTGCAGTCAAGGCTCTTGGATCCTTCAGGAGTAACCCTCATGTTTGA |
| >CrPIP2;6  ATGGCAAAAGACATGGAAAGTGCGGCACAAAATGCGTTACCACACAAGGACTACCATGACCCTCCACCCGCACCACTGTTCGACGCCGCCGAGCTCCGTAGTTGGTCCTTATACAGAGCCCTCATCGCGGAGTTTGTCGCTACCCTGCTTTTCCTCTATGTCACCGTTTTGACCGTCATCGGTTACAAACATCAGACCGACGGCGGAGACCCTTGCAACGGCGTCGGGATTCTGGGCATCGCCTGGGCCTTCGGAGGCATGATTTTCGTCCTCGTTTACTGCACCGCGGGAATATCCGGTATGTGGGTAATTGAATTCAATTAATTTGTAAGTGAATAAAATTAAAATGTGATGTGAGGTTACAGGGGGTCACATAAACCCGGCGGTGACGTGGGGGTTGTTCCTTGCTCGGAAAGTGTCGCTGGTTAGAGCATTGAGTTACATGGTGGTTCAGTGCTTGGGAGCTATATGCGGTGTTGGACTCGTGAAGGCTTTTCAGAAGAGCTACTACAACAGATACGCAGGAGGTGCCAACATGCTCTCCGATGGATACAACAAAGGAACCGGGTTGGGCGCCGAGATTATCGGCACCTTCCTTCTTGTCTATACCGTCTTCTCCGCCACCGATCCCAAAAGAAATGCCAGAGATTCCCATGTTCCCGTATGCAATTAATTAACCACCCACCCACTCTCTGCATAATAATCATTCATTTCTTCTACTTCCTCAATCTTTCTTGTTTTTATCTCCAGGTGTTGGCACCGCTTCCAATTGGCTTTGCGGTTTTCATGGTTCATTTGGCAACCATCCCTATCACCGGCACCGGCATCAACCCTGCCAGAAGCTTCGGCGCTGCCGTCATATACAACAATGAAAAGGCATGGGATGACCAGGTACCTACCTCCTATATATATATATATATATATATATATATATACACTATACTATGCTCTCTTCATTTTCCTACCTATCTGCTATATATATATATATATGGAAATTGATTGATTCATTGTATTGGCTTAGTGGATATTCTGGGTTGGACCTTTCATCGGTGCTACCATTGCTGCAATCTATCACCAGTTCGTGTTGAGAGCACAGGCGGCAAAGGCTCTGGGATCTTTCAGGAGCTCCTCAAACCTCTAA |
| >CrTIP1;1  ATGGCTCTCTATAGAATTGCAATTGGGTCCCCTGGAGAGGCTGGTCAACCTGATGCACTTAGAGCAGCATTTGCAGAATTTTTCTCCATGATCATTTTTGTTTTTGCTGGAGAAGGATCTGGCATGGCTTATAGTGAGCACTATTTCACACAAAAATGTGTTTTAGAACCTTATCTTTGTCCCATTTAATGTTACATTGCCGTTTAGATTTAGAATAATTTAAAGGAAAGTGTCACACATAATGAACTTGTATTTTGATTTGTGTAAATGTAAATGTCAACTCGATCTTCCACCATGGCATGTTATAGTTCCATTCTAAATACTTGATTTGAAACAGAATTGGGTAGAATAGGTTTAAGAATTGTGTCCTCTATTTTGATGCTGTTTTGGTCATGGCAGTGTGAGCATTTTAGGATGCTATAGAAATGACTAATTATTGGTTGTTGTGGCAGACAAACTTACCAATAATGGACCTGCAACACCTTCTGGTGTCATAGCTGCATCCCTATCTCATGCATTTGGTCTTTTTGTGGCTGTTTCTGTTGGGGCAAACATTTCTGGTGGTCATGTTAACCCTGCAGTTACATTTGGTGCCTTCTTAGGAGGAAACATAACCCTCTTAAGAAGTATTTTGTATTGGATTGCACAGTTGCTTGGTTCAGTTGTTGCTTGTATTCTTCTCAAGTCTGCTACTGGTGGAATGGTAACCATTAATCTTCAACTTTAAACACTACTTACTAACTCATATTCATAATTGATAGTAGTAATCTTTTGCACTTAGTACCCTTGAAACTCTAATTCTGTAATTTGCACCATGCAGGAAACATCACCTTTTTCTCTATCCTCTGGTGTGTCTGTTTGGAATGCATTAGTTTTGGAAATTGTGATGACTTTTGGCTTGGTATATACAGTTTATGCCACAGCATTGGATCCAAAGAAAGGGAATGTTGGCATTATTGCCCCAATTGCAATTGGTTTTATTGTGGGTGCCAACATCTTAGCTGGTGCTGCTTTTGATGGTGCATCTATGAACCCTGCTGTGTCCTTTGGCCCTGCTGTGGTTAGCTGGTCATGGACTCATCATTGGGTCTGTTGGGTTGGTCCATTCATTGGTTCAGCAATTGCTGCCATTATCTATGATAACATGTTCATAGGTGATGATGGTCATGAACACCTTTCAAGCAGTGACTTCTAG |
| >CrTIP1;2  ATGCCGATTTCTAGAATTTCCATTGGAAATCCTTCAGAGTTAGGCCAAGCTGATGCGCTTAAAGCAGCTCTCGCTGAGTTCATCTCAATGCTAATCTTTGTCTTTGCAGGAGAAGGCTCTGGCATGGCTTATAGTAAGCCCTAATTTAATTTGTTCAATTTCATAAATTAACCTTCTGTCTAGTTTTCATTGACAATTAAAAGCATGGAGTAATTGTATCAACTCTTGTGGTGTTTTTCTCAGACAAGCTCACAAACAATGGCTCAGCAACACCGGCTGGGGTGGTGGCAGCATCATTGTCTCATGCCTTTGCTCTTTTTGTTGCGGTCTCTGTGGGTGCTAACATCTCTGGTGGTCATGTTAACCCTGCTGTCACATTTGGTGCCTTTATTGGGGGCCACATTACCCTTCTTAGAGGCATTTTGTACTGGATTGCTCAGTTGCTTGGCTCTGTTGTAGCTTGCTTGCTCCTTAAACTTGCCACTGCTGGAATGGTAATTAGTAATCACATTTTACATATCTTCATTCAATGTATCTTTGGATAAACGTTTCTCAAATTACTTTTAGTTAAAAGTAAATTAAAGCAACCACATTTATGTTTTGAATTTTTTTCTTTACAGCCAACACTGTCCTCTATTATTGGGGCTCTCTACAATTTGTTTAACTTTACATCAAATTAATTTTTGTTAATAAAACTATAAAATTAAATATTATTATGTGATATTAAAATGAAACAAGGCATGCTAGTTTAATTGATATTGATATTTTTCTTGTGAATGTCAATTGCAGGAAACATCTGCGTTCTCATTATCTTCTGGGGTGGGAGCAGCAAACGCGCTTGTGTTTGAGATTGTGATGACTTTTGGTTTGGTTTACACGGTGTATGCTACTGCAGTGGATCCAAAGAAGGGTAACATTGGGATAATTGCTCCAATTGCAATTGGTTTCATTGTGGGTGCTAACATCTTGGCAGGGGGTGCCTTTGACGGGGCATCAATGAATCCAGCAGTCTCTTTTGGGCCCGCTGTTGTTAGCTGGACATGGGCCAACCACTGGGTCTACTGGGTCGGCCCATTAATTGGATCCGCTATTGCTGCGCTTGTCTACGAGATTTTCTTCATCACCCCAAGTTCTTATGAACAGTTACCTGTCGCCGATTATTAG |
| >CrTIP1;3  ATGCCGATTTCTAAAATTGCCATTGGACATTCTTCTGAGTTGACACAAGCCGATGCGCTTAAGGCTGCACTTGCTGAGTTCATCTCAATGCTCATTTTTGTTTTTGCCGGAGAAGGCTCTGGCATGGCTTATAGTAAGAAGCCCTTTCAATTTATTTATTTAACTCTATCCATTGCTTCCATTTCAACATATAATGATACTAATAATTAATTGGCAGATAAACTAACAAAAAACGGTTCAGCAACACCAGCTGGATTGGTGGCAGCCTCACTTTCACATGCCTTTGCTCTTTTTGTGGCGGTTTCTGTTGGCGCTAACATTTCTGGCGGTCATGTCAACCCTGCTGTCACTTTCGGTGCCTTTATTGGTGGCCACATTACTCTCTTTAGAACCATTTTGTATTGGATTGCTCAGTTGCTTGGCTCTATCGTTGCTTGCTTGCTCCTTAAAGTTGCCACTGGAGGATTGGTAACAAACACTATATATAGTTAATTAGTTAATGTTAACGATGATGAACATGTTTCTAGTTTTGTGCTAAGCAGCATCTCTTAATTTGGTGTGTTGCATTGTAGGAAACATCTGCATTTGCGCTGTCTTCAGGAGTGGGTGCGGGGAATGCGTTTGTGTTTGAGATTGTGATGACTTTCGGTTTGGTTTACACGGTGTATGCAACTGCGGTGGACCCAAAGAAGGGTGATCTTGGAATAATTGCTCCAATTGCAATTGGTTTCATAGTTGGTGCTAACATCTTGGCGGGTGGAGCATTTGATGGTGCATCCATGAACCCTGCGGTCTCCTTTGGGCCTGCTGTTGTTAGCTGGACATGGGATAATCATTGGGTCTATTGGGCCGGCCCATTCATTGGTTCTGCTATTGCTGCTCTTGTTTACGAAATTTTCTTCATTAACCAAAACACCCATGACCACCTCCCCACCACAGATTATTAG |
| >CrTIP1;4  ATGCCGATCAGAAATATCGCCGTTGGAAGGCCTGAGGAGGCCACTCACCCAGATACCTTGAAGGCTGCGTTGGCTGAGTTCATCTCCACCCTCATCTTCGTCTTCGCTGGCTCAGGTTCCGGCATCGCCTACAACAAGCTCACCGACAACGGTGCAGCCACCCCCGCCGGTCTCATCTCTGCCGCCATAGCCCATGCATTCGCCCTCTTTGTTGCCGTCTCCGTCGGCGCCAACATCTCAGGCGGCCACGTAAACCCCGCCGTCACCTTCGGTGCCTTCATCGGTGGCAACATCAGCTTCCTCCGCGGTATCGTGTACATCATCGCCCAGCTCCTGGGCTCCATCGTGGCCTCCTTGCTCCTGCTCTTCGTCACCGGATTGGTAAACCCGTTATTCCGTTACACATGTTGGTTTTGATTAACGAACTTTATAATTAATGAGTTTTGTTACGGTGCTTTGCAGCCTGTTCCAGCATTCGGACTCTCTGCAGGAGTTGGAGTGGGAAACGCTTTGGTGTTGGAAATCGTGATGACTTTCGGATTGGTGTACACGGTGTACGCCACAGCCATTGATCCTAAGAAGGGTAATTTGGGAATTATCGCCCCCATCGCTATCGGTTTTATCGTTGGCGCTAACATTTTGTTGGGTGGGGCCTTCGATGGAGCATCCATGAACCCGGCCGTGTCATTCGGACCTTCAGTAGTGAGCTGGAGCTGGAACAACCACTGGATCTACTGGGTTGGGCCTCTCATCGGTGGTGGGCTTGCTGGGCTTATCTACGAGGTCATCTTCATCAGCCACACCCACGAGCAGCTCCCTACCACTGACTATTAG |
| >CrTIP2;1  ATGGTGAAGATAGCACTTGGTACCTTTGATGATTCCTTTAGTATTGCCTCCCTTAAGGCTTATCTAGCAGAGTTCATTGCCACTTTGCTTTTTGTGTTCGCTGGTGTTGGATCAGCCATCGCTTATAGTAAGTGCTCCAGCGTGACTAGTAATAGTAGTTTCATATTTGAATTATACAACATATATAGAATTTTCATGCATGTTTCTGTGCTGCTTTAATCCTTGGATGGCATGCATGGACACTCCTCCGATTGAGAATTTCCGGAATTCAAGTTGGTTATATGATTCTACGAGAATGAAGGGATAATCACCTGAAATTGGAATATAATGACTTGAATGTTTTTAGATTTTATAAATATATTTTTTGCTTTTAATTATCATAACTTTTTTCTTGAACAATTTATGTCATCACATAGCATTGTCTTTTAAAAAATCAAATTTTATAAGGATAAAAAATAAAATTTCTTTAAGAGATTAAAACTATATTGAACCATATTTACATGGATTAAAAATATATTTTATTCTAATATAAATATTAAAAAAATCTATACATACTTTTCGAATTTTAGAACAGTTACAAGTATCACTATATACTTTTACGTTAGCTAAACTCCCAAACAACACTTTCTATTAATCATTAGTTGATACTATACTTACATAACAATGTGAATTTTTAAAATATTACATTTCGAACCCATAATATGAACATTTGAAGTTACTTTATTAAATTTGATTTAATGTAACCAATGCAATCTGTACGTTCAAATTAAATGACAGTAAGTTTTCTTAGTTATCTGATTACACTATGAATTGAACAATTAAAAAAGTTGGCGAGAATATTCATAAAAGAAGGAATTATATAATATTTTTCATAATGGACACATTTGTAATGTAGTATATAATATCATGTTTATTAAGAAACAGCTATAATAATGGCACAAACGTACAAGGAATAATGAAGTGGTTGTTTTGTATTATGTAGACGAGCTGACATCAGATGCAGCCTTGGATGCACCGGGCCTGGTGGCAGTAGCTGTGGCCCATGCATTTGCACTATTTGTAGGTGTGGCCATCGCAGCCAACATCTCAGGTGGCCATTTGAATCCAGCTGTCACATTTGGATTGGCTATTGGAGGCAACATCACACTCCTCACTGGTTTCTTGTACTGGATTGCCCAGTTGCTGGGCTCAATCGTCGCTTGTCTCCTCCTAAATTTTGTCACCGCTAAGGTATTTCTTCATATACGTTATACGCTAGCAAATTCTTTTGGGCTTCAATTACACGTATTAATAAATAATTCAATTAAATTGGTTAATGATAAATTGTATATTGATAGATAAAAAAATTTCTGATATGATATTCTTTTGGATATTGGCAGAGCGTTCCAACCCACGGAGTGGCTGCTGGTGTGAACGTTTTTGCAGGTTTAGTGTTTGAGATTGTTGTGACTTTTGGATTGGTTTACACTGTGTATGCCACTGCAGCTGACCCCAAAAAGGGCTCACTGGGCATCATTGCACCCATTGCTATTGGGTTTATTGTGGGTGCCAACATCTTAGCTGCTGGCCCATTCAGTGGTGGTTCTATGAACCCGGCTCGTTCATTTGGACCGGCTGTGGTTAGTGGAAACTTCGTTGATAACTGGATCTACTGGATTGGGCCACTGATTGGAGGAGGTTTGGCTGGGTTGATTTATGGTGACATCTTCATTGGTTCCTATACCCCTGCCCCACCCTCTGATACCTATCCTTGA |
| >CrTIP2;2  ATGGCTGGCATTGCATTCGGACGCTTCGATGATTCTTTCAGTTTGAGCTCAATCAAGGCCTATATTGCTGAGTTCATCTCAACCTTACTCTTTGTTTTTGCCGGTGTTGGTTCAGCCATAGCCTATGGTTAGTTTTGTCTGCCTTACATTAGAATTGAACCTAAGATCTTTGTCTCAAGTATCAACCCCTACACTCCTTTATCACATTACACTTACATTCAACTAGTTAAAGGTGTTACACCATGCTTAAACATAAAAATAACCCTTACTTAAAATAAATCAGAAAAGGATGAAACATAGTTTTTTTTTTCTTTTCTTTTGGCCTATAAGTTATATGTAGCATTTTTTTTTTAATTGTCTCCATGCACAAGTTTACAAAACACTGAAATCATGGTACCTTTTTAGTCTCAACACGTTTGGCTTTTATTTATTTATTTATTTTATTTATTTGCAGGTAAATTAACTTCAGATGCAGCACTAGATCCAGCTGGATTAGTAGCAGTTGCTATTTGTCATGGTTTTGCTCTATTTGTTGCTGTTTCTGTGGGTGCCAACATCTCTGGTGGCCATGTCAACCCTGCTGTGACCTTTGGATTGGCTCTTGGTGGCCAGATAACCATCCTCACTGGCATCTTCTACTGGATTGCACAGCTTGTTGGCTCCATTGTGGCATGTTTTCTCCTCCACTTTGTCACAGGAGGCTTGGTAAGTTTAAACTGAACTCATCTTTATGGTTCAAAATCACTTGTGGGATCAACATGGTTATATGCCTAATCTTTTTCCTTGATAAGATGCTTCAGCATAAACCGATTACATACATGCCTTTATCTACCTATAATTCTATTAAGGTGGCCATAGGTATAATTCTGCTACCGTTAAGATGGAACATACTACATAGAATCATAATTTAAGATACCCTCCATTTAATATTGATCTTGTTCATAAAAGATTAAATAAAAAATAATTACAATACAGGATAATTCAGACAACCCCTACTATATATTATATACAAAGTAAAAGAACAAATATAACAGCATAATGTTGAGTTCATTTTTGTTGAAATTATAAACTGAATTGGTGTTATTTGTTGTCAGACAACTCCCATCCATAGTGTGGCTGCAGGGGTTGGAGCTGTTGAAGGAGTTGTTACAGAGATCATCATCACATTTGGTTTGGTGTACACAGTGTATGCCACAGCAGCTGATCCCAAGAAAGGTTCATTAGGTACCATTGCACCCATTGCCATTGGTTTCATTGTTGGTGCCAACATCTTGGCAGCAGGACCATTCTCTGGAGGGTCAATGAACCCAGCACGCTCCTTTGGCCCTGCAGTTGTTAGTGGTAACTTCCATGACAATTGGATCTACTGGGTTGGACCTCTCATTGGTGGTGGTTTGGCTGGCCTTATCTATGGCAATGTTTTCATTCGCTCTGACCATGCACCTCTTTCTAGTGAATTTTGA |
| >CrTIP3;1  ATGTCAACCCGTAGATATGCATTTGGAAGGGCAGATGAGGCCACACACCCAGACTCCATGAGGGCTACTTTAGCTGAATTTGCCTCCACTTTCATCTTTGTCTTTGCTGGAGAAGGCTCTGGCCTTGCTTTGGGTCGGTACATAGTCAATCTCTCCTTGATGCTTAACTAGCTTAGTTCATACTTACATATGTTTCTTGTCATTTTATATATATTTTAACTTTTTCAGCTTTATTGTAAGATTTGATAAACAGTTCAACAAGTTCAATAATCTTCTTTCACACGAGTACTTATAAGATATAAAATTTATTAAAATGAGCTAAAAAAAAAAACTTTTTTTGACAACTTACATCAAAATGTTTAAGTTCAGCAAGTTAAAAAGAGTTTATAAGAATATTTTCAAATATCTCTAGGATATTTGTGCTTATGTTAACCTAACTCATTTATGATATTATGTTATACATGCATGTTATGAATATGCTAGTTATTTTTGGAGCATTTATGGTGATTGGTGAGGGTGATTTGTGTGCTTTGTCTTTGTTGCAGTTAAGATTTACCAGGATTCAGCTTTCTCAGCTGGTGAATTATTGGCAGTTGCACTTGCACATGCCTTTGCTCTATTTGCTGCTGTATCTTCTAGCATGCATGTATCAGGTGGCCATGTCAACCCGGCTGTGACATTCGGTGCTCTCCTTGGGGGAAGGATCTCAGTGGTCCTTGCCATATACTACTGGATAGCTCAACTTCTTGGTGCTATTGTGGCTGCTCTCATACTCAGGCTTGTCACTAATAACATGGTAATAGTATTTTCCGAAATAAATAAACCTATGAAAATGTTTTCAGGTATTTTAAGCATCAAAAGTGGTTCAATAAAAAGTTTATTACTTTTCAAGTTTAAAATTAGACTATGTTAACAAATCCAAACTCATATTTTACTTGTTAGATCATTAAAATAAGAAAAGACATACCCATCACTAATTTTTTTTATGGTGCCATTCATTTATAAAGCACATACATCATTTAACAACCAAACCACTCGGTGGATAAAACTGATATGTTATTGTGGCTGTTTATTGTAGTTAAATGTGGTAACTACAAAATTGGATAATAGTGACATGATCAAAAATTGAACTTTCAATGAAATCTGACATATTGTTGTGTGTGTTTGTTTTGTAGAGACCATCGGGGTTCCATTTGGCACCTGGTGTTGGAGCGGCACACATGCTTATACTTGAGATTGTCATGACATTTGGGCTGATGTACACTATATATGGTACAGCAATTGATCCCAAAAGGGGTTCAGTTAGCAACATTGCACCTTTGGCAATTGGACTTATTGTTGGGGCAAACATCCTTGTTGGTGGGCCATTTGATGGAGCATGCATGAACCCTGCTCTTGCTTTTGGGCCATCCTTGGTGGGCTGGAGATGGCACTACCACTGGATCTTCTGGGTGGGTCCATTGATTGGGGCTGCACTGGCAGCAATCATATATGAATATGTTGTGATCCCAACTGAACCCCCTCATCAACATCAACCATTGGCTCCTGAAGATTACTAG |
| >CrTIP3;2  ATGGCAACAAACCGAAGATATGCAATTGGAAGGTTGGATGAGGCTAACAACCCTGATTCCATGAGAGCCACCTTAGCTGAATTCCTCTCCACTTGCATTTTTGTGTTTGTTGGAGAAGGCTCTGCCCTTGCTTTGAGTCCGTACCATAACATTACATTACATGCTTAACTACTAATCAAATAGTATATGAATATGGTATTTTATGTTGATGAGTGTGTGATTCAATTGTGTGGTTGCAGGCCAGATTTACAAGGATACAGGTACATCAGCTGGTGAGCTAGTGGTTGTTGCACTAGCTCATGCTTTTGCTCTATTTGCTGCTATTTCTGCTACCATGCATGTCTCTGGTGGACATGTCAACCCTGCTGTCACTTTCGGTTCTCTTCTTGCTGGCAAGATCTCTGTCCTTAGAGCCGTTTACTACTGGGTTGCTCAACTTCTCGGTTCTGTAGTCGCTGCCCTTTTGTTGAGGCTTGTCACCAACAATATGGTACCTTAAATCCCTTCTACTGCTATTTGATTCCAGACAGGATTTCCTCCAGTACTAGAGGAATTCATTTTTCTGGGATCCGTTTCATATATATGGAATCTGACTGCATGTAAGAGAAAAAAAGTATAGAGGGAAGAAATTGAGAGAAATTTTACCTTTTTTATTCGTTAATAATTTAAGACAAATTTTACCTTTGTTTATGCAAACATGAATAATACAAGATAAGATTTATCCTTCCTCTAGGTAGTATGAAAGGATTCATGAATAATACAAGATAAAACTTAACCTTTGTTTATGTAAACTTGAATAATTCAAGATGAGACTTAGCTTTCCTTCAGGTAGTCTGAAAGGACCAAATTTAGATATAGTATGATAAATGTTGTTGGTACTTTTCTTCAATTAAAATGAAATTTTGCTTTGATAATTAATATTAATTTTAGTAGCATTAAAGTTAATAACAATTATTGAGATAATTTTTTTAAAATTTTTTAATTGAAGAAAAGTACATAGATATAGATATTGTATTTAAGTTTTGTCGAAACTGAGAATATGAAAAATCAAAGTAATGAGTGTGTTGTGGTTGCCTATTACAGAGACCACAGGGGTTCGGTGTGTCTGTGGGTGTTGGAGCGTTTCACAGCCTTATTCTTGAGATTGCCTTGACATTTGGTCTGATGTACACGGTGTATGCTACTGCTCTTGATCCTAAAAGGGGCACCGTTGGTTCAATTTCGCCCTTAGCAATAGGACTTGTTGTTGGGGCAAACATCCTTGTTGGTGGGCCGTTTGATGGAGCATGCATGAACCCAGCTCGGGCTTTTGGGCCTGCCTTGGTGGGCTGGAGATGGCACTACCACTGGATCTTCTGGGTGGGTCCATTGATTGGGGCCGCCCTGGCAGCACTGTTATATGAATATGTTATGGTCCAAACTGAGCCTCCTCATGCTCATCACCAACCTTTGGCTCCTGAAGATTACTAG |
| >CrTIP4;1  ATGCGCCATTTGTCCGTTTGTATTAGTGAGTTGTAAAAGGTTTGGACGTAAATTTTGTTTGCATATATAATTGGTACCTAGCTAGCATTATGATTAGTGTGGGAATCAAGTAATATTCATTGCTTGTGCAATGGAGAATCAGCCTCAGCCATGGCCAAAATCGCTCTTGGAACCACCCGAGAGGTCACTCAACCAGATTGCATTCAGGCACTCGTCGTTGAATTCATCGCCACCTTCCTCTTTGTCTTTGTTGGCGTAGGTGCTTCTATGGCCGTTGGTATGTAGCAACTACCAGCATATCACTCATCACCTTCTTTCATCTAATGACATCTTCATCTTTATTTATTTATTTATTTATTTCAAAAACTGTCGTTTAACGAATAGAATGATGTATTAAATTTATTTTGATTTGGTGTTACTGTATTAGACAAGTTTGTTGGGGATGCACTGGTGGGCTTGTTTGCTGTAGCAGTGGCACATGCTCTTGTGGTGGCTGTAATGATCTCCTCCGCCCACATTTCCGGTGGCCACCTCAACCCCTCCGTCACTCTTGGTCTCCTTGCCAGCGGTCACATCACCCTCTTCCGCTCCATCCTTTATTGGATTGATCAATTAATAGCATCTGCAGCCGCTTCGTTTCTGCTTTACTACCTTTCAGGAGGACAGGTAATTAATTATGCAATAAAATAAGATATTTTTTTATCATTAAAATTTAGTATAATTGATAAATAATTGTGTTTTTTTTTTAAATGTAATAAAGAAGTTGACTTCTAATCAGTATGTTTTGAAAGAAAAAATTTATTTTAATAATCTTTATATTTTGAAGAAATTAATTTTTATTGTTAGTAAAAATATTTTTAATATACACAAAAAAACTATTTCTATAATTTTATAATTTTTTTTTTCAAATTAAACTACATCCTAATCTATACTAATATTGAAACTGCTCGACTCAATAATATATCTCATTTTTCCTCTTAAAATTTCCTCTTATAGATATATTATTTGTACATCTATTAAACTGTGTTATTATTGTAGCAATCGAAACGGGGAAAACATGATGCTGCTTCAGCACACGATTTATACTAGAGTTTTACTAATCAGTGTTTTTTAATTAAGAAATCAAAATAAGCTTTTATTCAAAAATCATGTTATGATATATTTAATTTAAAATTAGTGAAAATGGTAAATTAACAACTTTTTGAATAAAAAAGATTCCCCTTTAATTTGTTAATTGGGATTTAATATGTTGCCTCAAATCATAGTATGATTGTTTACATAATATGTGTAAATTATGGTTTGCAGAATACTCCAGTTCATACGCTGGCGAGTGGAGTGGGGTATGGTCAGGGAGTAATTTGGGAGATTGTGTTGACGTTTGGTTTGTTGTTCACCGTGTATGCGACAATGGTGGATCCAAAGAAAGGAGCACTTGCTGGGATTGGACCAACGCTGGTTGGGTTTGTAGTGGGTGCCAATATCCTTGCCGGTGGGGCATTCTCTGCTGCTTCTATGAACCCAGCAAGGTCTTTTGGCCCTGCCTTGGTTTCTGGCAACTGGACTGATCATTGGGTTTACTGGGTTGGACCTCTCATTGGTGGTGGCCTTGCAGGTTTCATCTATGAGACTTTCTTCATTGACCGATCTCATGTTCTACTTTTCCCTGATGCAGAAAGTTAA |
| >CrTIP5;1  ATGGCTCCTTCTTCTGTCACTGTCACTTCCCGCTTTCATGAATCCGTTACCCGAAATGCACTTCGCTCCTATCTCTCCGAGTTTGTCTGCACTTTCTTCTTTGTCTTTCTTGTGGTTGGCTCTCAAATGTCCTCAAGTGAGTACCCTTTTGCATATTTTCTCAATTAGCTTAAGTTTCTTTTCATTAATAGATAAGTACAAATGTCACATGCATACTTATGTTTGTCCTTGTGTTCCCACACAAAATTGATGTATAACCATAACCATACCCTTTAAAAAAAAATAATGTTTACTTTTTGGCTAATGTTATGCATGAAAAGTATTCAGATTGCAAGTTTTGTTTTACACAGTAAGCGTTTCTGCATTGAGCATTTAATTCTATATTCGGTGGATAACTTGTAACAATGGAGATCAGAGAAACGCTGGGGTCCTCGTTGTTTTTATTGCGAACCGAAATGTAAAATCATAACTTATATATAAACATAATTTTTAAACTGCACGATCTCACGAGTCTAAATTATTTTCTGGCATTCTATATACCCATTTAATTTGTTTTAGTATTTTTTTTCCCAAATACTGATAGTTTTAAGAAACACGTATCTTTATTGTTTTGAAGAACAATTTTGTCTTATATTGTTTCTTACAAATAAAGGCATTAAATGGTTTCTTTTCTCTTTGCATATGGCTTTTAAAGTGATTACGACATGAATTTTCACATATTGTTTACAATCTCCATTTGTTAGACAGAGTAAACCATTTGAAATGCATTTTATTTTCAACAAGAAGTTCAAGCAATAGAACAATTGCATGAGTCACACCCCATTAATGTTTTTATTAAAGGGATATGCATTAAACTAAAACAATGAAAAATCATGATGTTTGCCAAATGACAGGGAAATTGATGCCTGATGCTTCATTGAACCCAACGAGTCTGGTGGTGGTTGCGATGGCGAATGCTTTTGCTTTATGTTCTGTTTTATATGTCGCATGGGATATTTCCGGTGGACACGTGAATCCGGCGGTGACGTTTGCAATGGCAGTAGGAGGTCACATTAGTGTACCAACCGCTCTCTTCTATTGGGTTGCTCAGCTTATAGCCTCAGTTATGGCTTGCCTTGTCCTAAGGGTCATTGTTGTTGGAATGGTACCTTTCATATTCACTAATTAAGCATAAGATAGCTATCTAATATCTAAATTTTTCAATTTTCATTCACTTTGTTCCCTTTTTATATTATGTTGTTTTTTCAGCATGTGCCAACATACACCATTGCGGAAGAAATGACAGGATTTGGAGCATCCGTATTAGAGGGTATCCTCACATTTGTTTTGGTGTACACTGTGTATGCTGCTAGGGACCCTAGACGTGGGCCTATGAGTTCTACAGGCCCACTTGCGGTTGGGCTAATAGCTGGGGCAAATGTGTTGGCTGCAGGCCCATTCTCTGGAGGGTCAATGAACCCAGCATGTGCTTTCGGCTCAGCCGCTATTGCTGGCAGTTTCAGGAACCAAGCAGTGTATTGGGTTGGACCTTTGATTGGTGCTTCAATTGCTGGCCTCCTTTATGACAATGCACTCTTCCCTTCTCACACTACACATTCAGTTACCCAAGGACTTGCTGTGTAA |
| >CrNIP1;1  ATGGGTGATAATTCAGCAAGCAATGGAGGCCACGAGGTGGTTTTAAACGTAAACGATGATGCTTCCAAAAAGACTGAGGACTCAGCTATCGAACATTCTGTGCCTCTTTTGCAAAAGGTGTCTCCCTTATTCCCTTCTTTCCTTCGCTATCCATAACTCGCTCACAACTTCAATTTTTTTAATTATTGTTTTTTTACAAAGATGAAAATAACAAAATTAAAACTAAAAATTTCATGTAAGTTATTTCAATTTCTTTGTACTAAACTAATCTTAATGGCTTAAAATTAATTATTTTTTAAAAAATGTACGTTGAATTTGTTTCAATAAAAAATTATACATATACTTAATGTTCTTTTGACAACTAGTGCACCAATTCTTTTAATGCAAAATTTATTGTTAAAATTAATTTAATATACATTTATTAGGCTTATCGGTTTAAAATTAAAATCCTGAATTTTAAAACAATTCTACCCAATTTGAAAGTATATACTCGTATCTATTGTTTTTGATAAAATTTTGAACCTTTTTTATGTGTTTTAAAATCTATGAAGTTAAATGTATCAAAAAGAATTTAAGATGTATCTAAAGAGAATTATAAATTGTATAGTCACTTCAAGAATAAATTTATTGATTATATTGAACCCGAAAATTTGATTCAAAATGATAAAAAAAAAAATACTACTTCCATATATGTCCAAGAAAGAAGTGGAAAACAAGATAAATGATCATATAAAATTTTATTATATATAAATGTAGTGTTGCACAAATGGTTTGTAACGTGTTTGGATCAGTGGATCGAGCTGAGAGAATAATAGTTTTTACATTCAAAATTAATGTTGAACCTTTATGATTGGTTTGTTCTTTGTCGAATGTAGTTGGCAGCTGAGGCGGCGGGGACGTACTTCTTGATATTTGCTGGTTGTGCATCGGTGGTAGTGAACCTTGGAAACGACAAGGTACTGACATTTCCAGGAATTTCCATTGTTTGGGGACTCACTGTTATGGTATTGGTTTACTCTCTTGGTCATATTTCCGGTGCTCATTTCAACCCTGCTGTCACCATTGCTCATGCTTCCACCAAAAGGTTTCCTTTCAAGCAAGTGAGTGAGTCAACCAATTACACCCTTTTCTCCCTTTCATTAAATTCATGGAGCTACTATACTGCGCAATATGCTCATTTGGTCAAATATACTGTGTCGTTTTCCTTCAACACATTTTTTGGTACCTCATTTAAAGCTAGCTGTGGTTGAATTGAAGTAAAAAATTGTTAGTTGCATATCTAAAAATAGATAGATTATTGTGTAAAGATGAAAAAAAAAATGTGATGTATTATATAATATGATGATAAGAGACTGAAAAAAAATAAAAAATATTATAATTAAAATGTTTATGAAATATTTAATAATAAAAGTAATTTATAAACATAATTATAATAAATTTATTTATTTATATATCAAAATCTATTTTTAATAATTAATTTTTAAGAATGCATAATTAATTATGTAATCAGTTATATAATTAAGTTAATTATTATTTTATATTAAAATAATCCTAATATTATTTAATTGATTACTACTTATATTAACTTAGTTATATAATTAATTACATAATCGATATTTAAATTTTAAAATTTTAAATATAAATAAATATGATTAGTATTTATAGGTGGAAAATTATCATTAATATAATAATTTAAGATTAACTCAAAACACCTAAACAAGATATTTCTCTCGAGTATTCACTACAGTTTTTAATAGTTCAAGTTTATCTTAAGTGATTGAATCTCCCTTCATTTTAATTAATTAATGTTGCAGGTACCTGCGTATATAATAGCTCAGGTCATTGGATCCCTACTTGCTAGTGGAACTCTCAGACTTATATTCAATGGTAAGAGTAACTATTTTCCAGGAACAGTACCGGCTGGTTCTGACTTGCAAGCCTTTGTGATTGAATTCATAATCACTTTGTTTCTCATGCTAGTCATTTCTGGAGTCGCCACCGATAATAGAGCGGTAACATTATTCTTTCCTGTTCTTAATTAATCAAATAATAAAAAAAAAAAACAACTTGCTTGTTCGGAGATGCTTGGGTCTTAATCTGTTGAACAATTTTTACTATGATATGTTTAGCAAATAGGGGTTCGCAAATCTCTTTCAAAACATCAAATATGGTTACTAAGTTAGCTAACTCAATATAAACATATTTGTAATGTTTCGCACTAGTTGGAATTATTGCTACTAGAAGTTTACATCACTAGACAGTAGACACATCCATGAGTACCAAAATCTATATCCTTTTGTTACTTGATTTTATTTTAAAATCTTGCAGATTGGTGAGTTGGCAGGGCTTGTAGTTGGGTCTACGGTGCTGTTAAATGTGTTGATTGCCGGGTATTAACTTATCTCAAAACCCAATTAAATATAGAAAAAACGATTCATATATATCCTAATAATACTACCAGGCATTAAACATTTTTCTTCTCTCTATTTTTTCCTTTTATCACGTTATATTATCCAGTACATTATGACTTTTTTCTCATTTTCTTTTGCATGTATAAATTTTAGAAAAAAATAGTATTTTTATAAATTTGAGAAAATTAAATCTTGACTGATATAAATTAATTAAATATTACGTGTATTTACATATTTCATGTAGTCAAAATATTAATATCTAAGTATATTTTTTTTATTTAAAAATTTGTTATAGTAAAAAATTGTTGTTTGATCTTTATAATTTATTATTTGATCTTTTATTTTTATTTTTATTTTATTTTATTTTTTAATAACTTATAATTTTTTTTTGAAATATTTTATATATGTATGTAATATAATTTGTTAAAAAATATAATAAAAGTTAGAAGAAATTAAAATTTTTATTAATTACATTACGTTAAAATATAAATTTTATTTTATTAAATAATTATTATAATTAATAGAAAACATTTGACATTCTGATTTCATAAATAAATTATATAAATAAATTAATGTTATAATAATTTAAAGATTTAATAAAATAATTTCATATTTCAATCTTTGCTAATAACGTAAATGAAAGATAATATTCTAATTTTGAACTTTTTTAACTTATTTAAATTTTCAATTAGATTTTTAAAAAAAATAATTATTAACTTTACCTACTTTAATAAAAATTATATCATATTATATATTATATATTTATTTTTTAGATCGATTGTTAAAATAACTATTGATTTTAAATGCAAAGAAAAAATAATATTATAATTTTATAATGTTTTAACGAAAATAATATTTTTTATTTTTTATAATTGAATTTAAAAATTTATATAAAATTATTTTGTGGATAAAATTATAATTAATTTATGATAAAATAGGTTATAATTTATTGTAAAAAGAAACAATTTAGATTTTTGAAAGAAAATATAAGCAAATTAGTGTTTTTTACAATTTAAATAATTTTGATGAGGAATAAAATTTAATTTAAGATTAATAACTATAATTTATTTATCAGTTAAATTTAGTAAATATTATTTTAAATATAATTTTAAATAAAATATGTAAATTATTTTTAATTTTATTTATAAAAAATTATATAATTAGTTTAGGGTTAAAAGTTTATTAAATGATTATAAAAATTAAATTATTTTATAATTAAAATAAAAATCACAAGACCATAATTTTATATATTTTTATATCATTCAATATAATGTAAAGGTTAATAATTTATTTTAAAAATTAATTTAATTATTGTGGAAAATAAATCTAATAAATTAATTAAGATAATAGTTTAATTTTAAATTTTAATTTAAATATTATAAATGATTTAGATAATAATTTAATTTAAAATTTATTTTAAATATTATAGATAAATATGTAAAATTTTATTTATATATTATAGGTTTAGGATTTTTAAGTTAATTAAGTTGATAGGTTTAGAGTTTTAAATTAATTAAGGAATAGTTTAATGTAAGATAATAAATTTATTTAAAAATTAGTTTAAATATTATATCAAATTAATGATTAAATTAAGTATAGCAATGATTTAATGTAAAATTTAATTCTAATGCAATAGGTAATTAAGAGAATAAGTAAATTTTGAAGCTAATTTATAATTTAATTTAAATAGTATAAGTAAATTAGAATATATAAATTTAAAAATTAATATTATAGGTATTAGATAATTAAGATAAGAATTTAACTTTAAATTTAATTTAAATATTATAAATAATTTAAATAATAATTCAATTTAAAATTTATTTTAAATATTATAGATAAATCAGAATATGTAAATTTTATTTACATATTGTAAATTTAAAGTTTTAAGTTAATTACGATAATAAGTTAATTTTAAATTTCATTTCAATATTTTACCTAATTAAAATAGTAATGTATTTTAAATTTTAATTTAAAAATTTAGATAATTTTAACAATAAAATTTTTAAAATTGTATGTCTATTTAAAATTTAATTTAAATTATATATAGAATTAACGTGTTAATATAAATTTTTTGATAATAAGTTAATATATAATTTAATTTAAATATAATAAATAATTAAGATAATAATTTAATTTTAAAATTAGTTAATTATTATTTGAAATTAATGTAATAAATTAATTAAGATAAGAATTAAATTTAAAATTTTATTTAAATATTTTAGGTAATAATTAAAATTAAATTTAATTGAAAAACAAATTAAATAATATAATATTCATTTAAATAATATTATATTCATTTTTTAAAATTATATTAAATTATTTTTTATCTACTTATAAAAATATATTTTTTAAAAGATTAATGTACAATAATAGTATTTTAAAGTCTTACAAATCAAATTTTTTAAAAAGGGTTAAATATGTTTTCAGTCCTTCTAAAATATTCAAATTTTGGTTTTGGTCCCTTATATTTAAAATCATTGGTTTAGGTCCTTCATTTTTTAAAATACATTGATTATAGTCCCTATATTAACTTAAGTTATATTAAATCAAAATATATTAATCATGATTTAAGAATTATAATCAAAACATTTCATATATTATACGGTTCAATATATTTATATTAAGTATCATATTTATAATTAATGTTGTCAAAATATAATTAATATAATAAGAATATTCCATATTTTGATATATTTATTTTTAAGAATCTTCTAAAATTATAATTTATAAATTGATTTTCTAATAATTATTATTATCTAAATAATTATTTATAAATTTTAAATGATATCAATTTTAGAATGACTCTTTTATTATTTTAGTAACAAAAATTTATTCTATTGTTTTCAATTTAAAATTAACATCATAATCTTAATTATATAAGATATAAAATTTAAATATTTTAATATTATTGTATAATTAATTTTAGGTTTTTAAAATTTTTAATATTAAATTGTGTATATCATAAATTTTAATTAGTGGAAATAATTTTTTATATAAAAACATTACATATTTTCTAAATTCTAATAATTTCCAATTATTTTATGTTAAATTAAATGTTAAATAAATCAAGAGAATTCATAAAAGTATATTATTAGATATTAAATTTAATATATAAAAAAATTTAAGCTTTATTATAAATTTAAATTAGTCAAAATTAAACTGAATTATTAAATTTTATAATCAATTTAAATATAAATTAGCTTTAAAAATAATAAAAACAAATTAATACTAAATTATGTTAGTATTTTAAATTAGTTGTATTGCAATCTTTCATATATTTTTTTCATATAATAAAATAAAAGGTTAAATATGTTTTTAGTCCCTTTAAAATATTTAAATTTTGGTTTAATATAACTTAAGTTAATATAGGGATTATAATCAATGTATTTTAAAAAATGAAGGATCTAAACCAAATGTTTTAAATTTAAGGAATCAAAATCAAAATTTAAAAATTTTAGAGAAACTAAAAATATATTTAACCCTTTTAAAAATATTAACTAAGGTTTGACTGCATTAATTATAAAATCATACATGACATTATTTTATTAATCTAAATCAACAAAAATGTACTATTCTCATATATACAAAGATATCCAAGCAAATACGTACTATCCTAAAAATATCTATCAATCTTTATTATTAAATATAAAATCTTATTGCAAGCCTTTATGTGAATAATATTACATTATTCCATAGTAAGATGTTACAATCTTAATTTTGGAAGCAAGGTCAAACATAAAACTAATTTGTGCGTCAAATTATATTTTGTGACAGGCCAATTACTGGAGCATCAATGAATCCAGCAAGAAGCTTAGGCCCTACTATTGTGTACAACGAGTACAGAGGAATATGGATATATTTGGTGTCACCGATTCTGGGAGCTGTGGCGGGTACATGGATCTACAATTTCCTCAGGTACACCACAAAGCCTGTGCCTGAGATCACCAAGAGTGCCTCTTTCCTCAAAGGACCAGAATGA |
| >CrNIP1;2  ATGGGAGATAATTCAGCAAGCAATGGAAGCGACGAGGTGGTTTTAAACGTGAACGGTGATGTTTCTGTAAAGTGTGAGGACTCTGTGCCTCTTCTGCAGAAGGTATATATCTCTCTCTTAATTAATAATATTCCCTTCTTTAATTTCTTTAACCACAACTCATTTAGATTTAAGAATTGCTGTGTTTCCAAACCGATATGGATTTGTTGACGAAATCAGTTGTTGTCTATGATAAAATTGATTCTACTTTTTTTTTTTTGTTTAAATTAAATCCAACCTTTTTGGATTGAAAATGAGTAAAAAAAATGGTGTGGCTGTTTGATGAATGTAGTTGGTAGCAGAGGTGGTGGGAACGTACTTCTTGATATTTGCAGGATGTGGTTCGGTGGTGGTGAACCTTAGCAAGGACAAGATGGTGACGCAGCCAGGAATTTCTATTGTTTGGGGACTCACCGTTATGGTATTGGCTTACTCTCTTGGTCATATCTCCGGTGCTCATTTCAACCCTGCTGTTACCATTGCTCATGCTTCCACCAAAAGGTTTCCCTTCAAGCAGGTCACTCATTATCTTCTTTTCTCTCTTCTCTTTCCATCTATTTTCACCTTTTCCTTTGCCTAAATGTATCATGTCATTTTCCTTCCATACAAGAAAGGCACCTTTTTTGGATTGAAATACCTCTTTTCATGTCCTTCTCTTAAACTCTTAGCATTTTTATTCTGCTTTCTTAAAACCATACTTCATATCCAAACTAAACACATTATTGTCCTAGATTGTGTTAAATTGTATTGAATATTTTCTCCATTTGATGTTGCAGGTACCGGGTTACATAGTAGCTCAAGTCGTTGGATCCACACTTGCTAGCGGAAGTCTCAGACTTATATTCAATGGCAAGAATGACCATTTTGCAGGAACACTACCCGCTGGTTCTGACTTGCAAGCCTTTGTGCTCGAATTCATAATCACTTTCTACCTCATGTTCGTCATTTCTGGAGTCGCTACCGACAACAGAGCGGTAATCTAATTCTTTCCTGTTCTTGTTTGGTGAATTGGGGATGGTTGGGTTTTAATTTTCGTTGACCATTTTGACTATCATTTTGAGTATTAATCAATGATTTTCTACCTTCTCTACTTATAAACATATAAACAGATATATCAAATTTAGTGGTTAGAATTTTTTTATCTTTTTAAAATATAAAAAAATTGTATGATCATAAATAAAATATAGATGGATATATACAAAAATTATTTCACTTATGTGTTATGGATTATCTAATCCAATCTCAATAACTTAATATTTTAATTAATTTTTTAAAAAAATATTTTTTTCAAATTTAATTGAATAAAATATTTATTAAATCAAAATCTATTTGAATAATTCATAATTTTATATAATAATTTTGTTTCATTCAAGAATTCCTCCCTTAGCAAATTAGGGGATAATAACTTTGGTCTTTGGACATATAAAAAATGGTTACTTAAATTGACTAACATAATATGAACATACTTATTATGTTTCGTATCAATTGGAATAATTGCTAGAGGTTAACATCACAGGACACATCCCGAGCGTATTAAAATCTATATTTGAATATTCTACAATCCTATTTGGTATCTTCTGTTAATTTGTTTTATTTAAAAATGCTGCAGATTGGTGAGTTGGCAGGGCTTGCAATTGGGTCTACGGTACTTCTAAATGTGATGTTTGCCGGGTATTAAGAATATTATTAACCAATTGATTAAAATTAAGGTGTTACACTCTTTTGGAAACATTGGTCAAAGTTGGATGTTATTTGTGTATTAATTTGACAGGCCAATAACAGGAGCATCAATGAATCCAGCAAGAAGCTTAGGACCTGCTATTGTGCACAATGAGTACAGAGGATTATGGATATATTTGGTGTCACCAACGGTAGGAGCGGTGGCGGGTACATGGGCCTACAATTTCATCAGATACACCAACAAGCCTGTGCGTGAGATCACCAATAGTGCCTCTTTCCTCAAAGGATCACCACCTGAGGGTGGATCCCACTGA |
| >CrNIP1;3  ATGAAGAGTACAATTAGGAAGATTGGTTATACTATAATATAAAATTATTGTCTCTTTACGAACAACTTTTTGAATGCCTATAAATTGGGGTTGTTTGTTGAGTCTTCTTCATTAGTAGTTGAGGAGACAGAGGAGAGAGTGTTGTGGGCAATATACAAACCCTGAGTTATATTTCTCAGTTGTTTCCTTCTACTCTGTGGCTGATGGCTGATAATTCAGCAAGAAGTGAAACCCAAGAGGTAGTTTTAAATGTCCCAAAGGACCCCTCCAAAACATATGAACGCTCAGACTCCTATGTTTCTGTGCCTTTCTTGCAGAAGGTGTCTCCTATATCCTAATCTTTTTTATTTTGTACACCAAAAACCCATCAAACTCCTCTTTTCATAATTGATTAGGTAAAATGGATTGTTATCTCTTAGACACTGAATGTTTATTGTTATTGTGGAATACTAGAGTATGTGTGTAGTATTTTTCTGAACTAATTAGTATATTGATCATATTGAAGTTAAAAAATTGAGATTAGAAATGAGAAAATGGTTTTGGTTATTGATGGATGCAGTTGGTAGCAGAGATAGTGGGGACATATTTCTTGATATTTGCAGGGTGTGCTTCAGTGGTGGTTAATAAGAATAATGACAATGTTGTCACACTTCCTGGGATCTCAATTGTTTGGGGACTGGCTGTGATGGTCTTGGTTTACTCCGTTGGTCACATCTCTGGTGCCCATTTCAACCCTGCTGTCACCATTGCTTTTGCCTCCACCAGAAGGTTTCCGTTGATGCAGGTGAACCAATCAGACCCTTTATTTCCTCTAAAAACTAGGTTAATATAAATACAATTTTATTTTAATTTTTAATGTGAATTTTATTTTTTAAAATAATTATTAGAAATTAATAATAATAATTTATAATTAAATGATAGTATAAAAACCGTCAATATGTTTTAGTTAAGTTTCTGCAAATAGGTAAATGTGCTAACTCTCCAGCTCCACAAGAAAGGAGAATTATTTAAAGGTCATTTTTCTTGAAATTGAAATTATGAAATACCTCTTGATTTTGTTTTTCCCTCTTTGGTCTTCTCACAAGATCATTGTCTAAAAAGGTAGAAAATTTATGGAATCAAAGAAAGGGTATTCTTGTATGCAGTATTGTCTTAATTAGTGTTAAAATAATTGGTTTGGCGATGTGATGCAAATCCTCTTCCAATAGGTACCGGCTTATGTAGCTGCTCAGCTTCTTGGAGCCACACTTGCAAGTGGAACTCTGAAACTGATATTTAATGGGAGCCATGACCAGTTTTCAGGAACACTCCCAGCTGGATCTAACCTTCAAGCTTTTGTGATTGAATTCATAATCACTTTCTATCTTATGTTTGTCATATCTGGGGTTGCCACCGATAACAGAGCGGTAATGTCTCTCTCCTTAAACTCACATCATATTTGGACACCTTCATACACATAGGAATATCTTATTATTATTTTTAACGTCGAAGGAATGTCTTTAAACTTTATTAAATCAAGATATGATATTTTTGGTACATAATCAAGTGATGATATTATTTATGTGAAATGATTGTGATATATATATATATATATATATATATGTTATATTTATTTTTATTTATTTATAATTATATGATTATTTTTTATATTTTTAAAAAATAAAAAATAAAAAATAATTAAATTTGTTATAAGATAAATGTAAGATAATTTTTATACATAAATTTATCATTTCTCTATTCGTAGTTTATTTGTTAATTAAATTGCATTTAATTGGGTAATAATTGGATTTAATTTTTTAATTTATATTCGTAATCAAGTATAATAGAATAATATGTATTTAAGATTAACCCTAAGCATAAAATTTCTAATTTTATAATATTAAATTTATAATTAAAACAGATTTTAATTGAAACGAATTTAAATATGATATAATATTTATAATATGTTGTTTAGTTCTTAAGGTCTTAAAGCAAGGTCCTACTAGGTTGCTTTTTATTTTTATTTTTCTAAAGGTTTCAAACCGATTTGACTGGTTTTTTAACAGTTTTTTGAACCGTTAATGCTTATAGTCATATAAGTTGAATACTTAATTGTCTCATTCAATTTTTTGTCAAAAGTGACCAATCCAATCCAAATTTTATAATAATAACACGAAGAAATGGAGACGGGCATGGCGAAACTTAGAGGTCAATGCTTTTGAAATGATGATATTGGCACATGAAAATTTGCATTATTCTCACACTACTTTTTTTTTCAATAATAAATAATATATAAATTTTAAATCATTCTTACATCATTTCTTTTCTAAATGATGAAAGGAGAAAATTACACAATATAGGTCAGCCCCATGTGTAACTTGGGTTTAAAACGAGTTTTAAGATAAAATCTTCTAAATTGATTAAAATTACTATAATTTTATTTCACATTTATTTTCAGATTCTTAAAATATAAACTTATATCAAAATAGGTAATAAGTATTTTGATGACTTTACTCTATTTTACTTTTTATAATAATATTTATAATAAAATATAAAGAAAGATATGCATTTTAACTCGGATTAATATAATAAAAAAACTCAACCAACAAAATAAATAAGATAATTGCTATTTATTAATTAAGAAAGAAAATCATAGAATGATAAGGTATTTTTTTTAAAAAAATGTTTATTCCAAATATTAGTCATTTGGACTAATTAATGATATTCTTTAATCTTTTTAGAAGTGAATATTGATGAAAACAATGATTTTTATGTTTTCTCCATCATATGCATGAGACAAAAGAATAAACAAGAAAAGCTTTTGTTTTAAAAAAAATTCATTACATTAATAATAGAAAAGTTATTGTATATTTTTAAAATATTTTGATAACTTAAGATGTGTTCTTAGTTTATGAGATAAAAAAAATTAAATTAAGAATTGGTAAGAATCTAGATAACTTATTAAAAAAATAAAGATAAACCTCAATTATAAAGGTACAACTTTGCAGAGCTACATGATCTATAACATCTCAATGTTCAAAAGCTTCAATTTTTTTTCACAAATAACAATATATTCTTTCTTTTGATTTCTTTTATTATTTATAGATAAGACTGCCTTGATTTTTTTTTCTATATCAACCCAATTAACTAAAAAAAAATAGTAACTAACCTCATCCATTATTGATTGTAAAACAAAATCATAACTAACAACTAATGAATTTCTATCATAATCTTTTTTTGGGACTTATTGTATATTTTTACAACAATTTTAAAACTATGAAACTTTTAAAAATGTGGCTTAAAGGAAGGGTTTTAAGTGCTTGACCCTATGATTCATCCTGTTCACAAAGCTTAATTACCATTTTTTTTATATTTAATTACAATGAGTATGGTTGTTGTGGGAGGGTAAATTATGGTACGGGAAAGGCTTCTCTTGTTTTTCATTTCTGTATTGATTATACACTACGGGCCTATACCCTATAATATTTTATTACGACAATATTATTTCAAATTTGAGAGGTGAAAATATAAGAGTAAGTTAACCATATAATCAAGATAAATGACCATAAGATTAATTTATTTTACTTCACTCATTTTCATTAAATACATCCCTTATGAGAGATTGGCAATTTTTAAAAATAAAAATATCCCCAAGACTTACCTAGAAGGCTAGAACTTGTAATAATAAACTTTGAAATAAAGTTAGCTGAGTTTTTGGTTAACGCTTTTCGATTTTATTAGTTAATCAGACAAACCACGGCCCAATGCATTGTGGCGCAGTTAGAACTTTGAATTTCCTAAATTTTTTAGCAAACAGGGGTTCGGAAATGGAAAAGAAAAAATGGTAGGAAATTGTATCCTTATGAATCACTTGCAAAAATGCTTGAAATATCAGAAGCGAATCAATAATCCATCCAAGAAATATTCTTGTAAAAGTATTTATTTATTTATTAAAATGAATTTAAAATTGATGTAGATTGGCGAGTTGGCGGGGCTTGCAATTGGGTCTACAATACTGCTGAATGTGATGATTGCAGGGTATGAACTTATCCAAATCCTAATTAAATGCAAAATGAAGAGTATTGTTTTAACAGTATTGGAATTGACAGGCCAATCACAGGAGCATCAATGAACCCAGTTAGAAGCTTAGGACCTGCTTTTGTACACACTGAATACAGAGGAATATGGATATACCTGTTATCACCGGTTGTTGGGGCAGTGGCTGGAGCATGGGTCTACAACATCATAAGGTACACCGACAAGCCCTTGCGTGAGATCACCAAAAGTGCCTCTTTTCTCAAAGGATCAGGCCGTGCTGCCACCAATTAGTTTAAGCAATTCCACAATTGTTCCCACTTATCTGTTCTCAGAAGAGTCAATAACTTCTGCCATGTAGTTCTCTTTCTTAGAAAGAAAATAAAAAAAAAATATTCTCTATCAAGTTATTAAGAAAAGCTACATGATTTTATTTCCTTGTTTTGTGCAAGGTAATGCTTTTTTATCTTGGAATTTTGTATGTTGTTCCTATGAAAACGTAATTAATAGTAAAGTAGGAGAGTAATTGTGGATGAAAGTCTAAAGGTCTCTCCAAACTATGTTATAAATAGATTTTTGAGTCTTGAGAAAAAAAAGATTTTTTTTCATAAATTTTTATGCTGCTTTCAACTAAATAATATATCAGAGCTTAAATAGAGTGACTGGAATTTTTTGGAGTTGATAAATGGTGAATGATAACTTGGATCAATTTAGATTTTTCCATTTCACCTAAGAAAGTTATGATAAGTGATATTATAGGAGAGCTATGTACAGATTCTCAAGATACTTAAGATGTTGTATAAAAAAGGTGACGCTTTGATTCAAATTGAGGCTTCCTTTTTACTAGATGAGAAGGAAACTTTATTCAAGTTACAGGAGAAGAGATCAATAAACACTTGCATTCATCTCATCCATTATTGTTTATATGAAGTCTTGTTCAAGAAAGTGTTTAATAAAATAATCTAAACAAGTTTGGGGATATTGAAAACCGTCAATTAAAGATGTTGGCAAAGTGAACAAGCAGATAAACCTTCAAACTCCACATGCATGGTGAGCTTATTTTTGTAAAAATGAACAATAATCAATTAGTTTATGGAAGATATAAAATTAATTATATTAGTTTTGTAAAATATGAAAATTAAGTTTGAATATTTCTAAACGAATATCCCTTTATTCAAATTAATCTACATCTTAATCTGATGTTAGCTAAAGTTACCGTACATGTATACGTGGTACATCAAATCATGAAGTGTCTTGTTGGATTTACATGTGGTAATGCAAATCCCTCCACCCTCCGATAGTGAAACTGCCCTAGTTTTTGCTCTATCATTTTGATGGTGTTAAATTGAACGTTGATGGTGTTAAATTGAACGTTGGTTCATAGGATAGCCTCTTTAATTTTAGTTTTAGCTTAAAGTTATGAACCTATTTAAAAGTAATTTTTTTTAAATTTAAATTTATATAATTTTTTTTAAAATTTAAAAGGATTTATGTAGGTTAGTCTATAAATTAATTTATTTAATTTAATTTAACTTTATAATTTATTTAAAAATAAAATTTTTAAATTCTAATTAAATAGATTAAGTAGAACTAGACTTTGAATGTTTAACTCTTGTTTATTTAAAAATTTAAACTTTGAGTCTAGATATATTACCTGTTAAAGATATTTTGTTTTTCAGTTGATTTAAAATCTATAAGATCTACAAAATTATTTAAAAGTCTAAATTATAAAAAAATATTAATAAATAAGTTTAAAGTCTCTACAAATGATTGATTAAAAAGGTTTTTAAATTTAAAATTTACAAGATAAATAGACTATTAAATAAGCCTAAAACATATATATATATTATATAAATATAATAAATATAAATATAAAATATATAATGATAAATAAATTTTTGAATAAGTTAAACAAACTTTTTTGTAAGCTCAAGTTTGACTTATTTGTCTAAACTAAAAAGACTTTTTAATAAGTTTGTATCTATCTATTTATTAAATAAGTCAAGTTAAGTTAGATTTTAAACAAGTTGAATTGGACAACTTTATTCACTTTCATTCCCAATTCTAACTCACGTAACCTTCAAATTAACTCATTCAATCCTATCTTATCATCTTTTGTTTTGTAACTTGTTTAAAAGTAAGATTTTATATACTTCCATTGATGTAATCCTTAAATTAAGTAAAATGAATATGAGTTAAATCATTTAATCTTATCTTATAACATTATATTTTGTAGCTAAGTTAGAAGTAGGACTTTTAAACCTTAACTCAAGTAACCCTCAAATTAAATGGAATGGATGTAGGCTAGCTCATATATTTTGTAACTTGTATAAAACTAATATTTTTAAACTTTAATCCATATAATTCTCAAATTAAGTAAAATGTGGGAATTAACTTTTACAATACTATCTTGTATTTGACTTTTTTTCTCTAAAATTTCTTTCTCTCTCCTTTTTTTTCTCTCTTAAAATTTTTATATTTAATATTTTTATTGTACTAAAGTATTCTTTAATTTATTTATGTTTCTTTTTAAGATTTTTATCTTTCTTTAAATATTTTTTCATTTATTTTTAGAATTTTCTTCTTCTTTAAATTTTTTCTTTCTCTCTCAACTTTTCTCAATCTTTGTATAGTTTTATCTTTTTTTTTTTTAAATTTTTTAAATATATTTACCTCTCTATCTTTAAACTTATATTTTCTACCAATTTTTCTTTATCTCTCTAAATTTTCTTAATTATTTTAATTTTTCATCCATGTTTTATTCTGAAAGAGGAAAAGAAAGTAGAGGAAAAAAATTATAAAAAAAAATAAAAGAGAAGATTTGAAGAAATAAAAGTAAGCTTATGGATTAATTCTAACCTTGAGGGATTAATGAACTTTTAGCTTTATACACTTTACAAATGTGGGCTTGTGGACTATTTTGACCCTAGCCCACATGACTAGGATTGAGTTGGGCCAAATTGATACCTATAATATTTATCTTTTCTAACAAATTTATCTTAAGCTAGAGGCATCATCAATAACATAAGTTTAACTACAAGAATAATCAACCTTTTATTATTAGGTTGTTATTATAAGCTCTTATATATTTAACAATTATTTTAATTTCTTTTCATTCTATTTTATTTATCATCCAAACAATCGAACATTATACCTTTAATAAGTAAACTAATAGAGTTTCACCAATCATAGAAATGTACGTGAGAAGAAAATTACAAAATATAGAAGATAATTTTACTTACCATTCTATTATAAAGAAAGGTGAATGAGTTGGTAGATAGAAAAAATAAATTGGCATAGAAATTGATAATTTAGTTACGAGAGAATCATTGTTAAGTATTAAAATGGAATAAAGGATAGGTAGACAAAAATTTGGATTAGACTCTTGGATGAAAAAAGACACTTATTAACTTTTCATTTCTCTTTTTCTTTCGTTGTTGTCGCCATTTTTTTTCTTCATTGATATTCAACTAGCTATTATTCTATTATTTAGTTTTTTTAATCATTTTTTCTTGAGTTATATGGTTTGGTATCAAATCTCTCAATCTGAGAAGTTTAAATGATTACCACGCACGCTAAAATAGTTTTGAGAATAGATAATCTAAAGAGGAGCTTTCAATGGAGGTTGGAAAATCAGAATAAGCATGAAGTTCATAGAAAATTGTTTAAAGGAGATGATGACATTGGAAGGACGAGCACTGAACTAGTTTCACGAAGCTTTGCTATGCAGGTTCCGACCATGTATTGTGCAGAACTTATATAAACCTTTATTAAAGGTGAAACAAAATGGATCTTTTATGAATTACAAAGAGAAGTTTGAGTATTTTGGAGCTCTTTATAAATGCTGATCCAACTATTCTAAAGAAAATTTTTCAGAACAAACTCAAAGAGAAGATACAAGTAGTATTATTTTCTACATTTCATAATTTTTTTCTTATGTATATCCTTGTGATAGGTGAACCCTATCATAAACTTAATTTTTTTATTTTAATTTTATATAATATTTTAAATTTATTTTTTTACATTTAGTATCATACTAAATTTATATAATTTAAAATTCAATTAATTGAATGTAATTTGTTGTGATCCATATAGTTTTGATAAAAATAAAAGTCTTTTATGATTTTGTTGTTTAAGTTAAATGGTTAGTTCGTATATATTTACATAAATAAGTTTGATTAAATAATTTTAACTCTTAACACCATATGATGAAAATAAAGGTCTTTTATAAAAAAAATAAAGTTCTTTTATGATTTTATTGTTTAAGTTGAGTGATTAGTTTGTATATATTTACATAAATAAATTTGATTGAATAATTTTAACTCTTAACATCAATCGAATGGACTCAATTTTAATGTTTTATAATTTGAAAATTAGTTTTTATTTTTTACCAAAATAAAAAATTTAATGTCTAATTTGATGTTTTTCAAATAGTAATATATTAATCAATCAATTATGACATATTTTTAAAAAGATTAAATTAGTTAAATTTTCTGAGGAATTATTTAATGGATTAACATCTTACCTTATCCTAATTGATTAATATAAACATTTTTTTCTGGTTGGTTGCATAGTTCCATTTAATCAATTACAGATTAACCATAACTGGTTCAATTGTGAAAAACCAGTTTCTAGATAATTAATGAACCAAATAATTAATTTATTAATTAAAATTTTAATTGATTAAATTGTTTCTAAAATACTTTCTTAATTAATTAATGTGCATTCTAATCAAGGTTTTCTTTAATAGTACTCACGTTTTTTATTATCTATGTAAACATTTAAGATAAAAAAATAATAGTAACATAGACAAAAAAAATTACTTAGATTTAAAAGTTAAAATATTAACCTGTTCATCATCCTTTTTCTTGAGATCTTTGACAACAATTGAGAATTTTTGAGTTTTAATGTGGTGTTACTTTACTTTGAGTGCGAGATACTTTTTTTTTAAAAAAAAATTTAAATGAATTTTTTTAAAAAAAAATCATGACATTTTTTTGGAGATTCTTGATTTATGATAGATTAGTAAAATTGTAAGTTGTTATTAGTGTTATTTAATTTTAGAGAAAAATTTGTAAGTTATAATGGAAGTTTCAAGTTAGTTGGCTAAAGAATTAGATGTAATATTATGATCCATTTGGAGATATACTAGGATGACTTGTTCCATTTTATTAGCTAAAAAAAATATGATAGGCGTATTTTATTATTATTATTATTAAAATAATAATAATAATAATAATAATAATAATAATAATATAATTAGATATAATAACAGAACAGAACAAACAGATATTTGCAAATTTAAATTTTACTATTATGTTTAAATATAATAATAATATTAAAAATATTAGTGATTATTATAATATATTAATTGTGATAATAATAATAATAAAATTAATAACATATTAATAATAATAATAATTAATATCTAATTTTTATAATAATATTATCATTTGAATATAATTTATATTATATTTTATTATTAATTAATTCATTTTAAACATAAAGTAAGATAATATAATATTATTTAATTTGTTATATTAATATACAAATAAATATAAAATAAAATAAATTATATCTTATCATATCTTATTTCTATCATATTATGATATTTTATTTTATCTTATTGTCCAAATAAACACTTTTAGTAAAAAATATGAACTAATATAAATCAAGTATGATATTTTTAATCCTTATTTCTTTAATTTTATTCTTATATTTATCATTACATATTTTTTATACATTTATTTATATCATCATGCATCACTTTTAATATTATTTTTATCATCAATCATCACCACATCAATAACTTTCTTTGTTCAAAATTTTTGCTCTTATATTTTAAATAATTTTAAAATGACTTCTTGTTTCATAATTAAAAGGTTTTTTTTTTTAAATTTAAAACATATTTTGACAATTCAATGCGTTCTCCTCTTGAATTAAAACCATTCGCTTAACATTATTGTGATCTTTATTTTTTATGTATATATATAATTTGATACATTTTTTATTAATTATAAATAAAATAAATTATATTATGATTTTTGTAATTCTTACGACAAATTTTATTAATAATGAATATAAATATGTTTTCCATGTTTGATAATACAAAATTATTTTTTTATCGTTCTTCTCTTTAATCTATTTTAAAGGTTATTTTATACAGATTAAAAAAATTAATTAATATTCTCATTTATATTAAAACTATCTTGTATTTTTTATTTTTTTCATAATTAATATCCTTGTAAAAAGCAAAGATTTAAAGGATGAAAATAATAAATATTCTTAAAATTTTAAAGTGATAATTAAAAAAGTAAGTCAAAATGAAAGAATCATAAGTAATTTTTTTGTTAAAACATATATTTAATATTATCTACATAAATTTATCATACTTTTAAATACAAAATGGTACTTATTAATTTATTAATCATTGTCTTAAAAAAATTAAATATTAATTAATATTAATCAAAGATTGATTTAACCAATAATATGCACACATTCGGAAACATTAAGTTTAATTTTCATCATATTTACGAAATATATGATGGCATCATATCTTTTAGAATTAATTTGATTATTTACTTGATACAAGTTTCTTCAGCAGCCTAGTTAAGTGAGTTGAGCATAGATTTTTTTTGCAAGGTATATATTTTATTATTTTCAGTAATATAGATGGCATTAGTCAAGCACTTTTGTATTCATAAGTATGCGGTAATATTTTCCATTTTTTATGTTTTATTGTTGCATTGACTTCCTATATTTTTAATTGTATTTTATCCAGGTACATATTGTCTACGAAATAGTGGATTTTTTTTTCTCTTTAACTTTCAATAAAAACTTAGTTTATTGAAAATGTTCGTCATATTTCTCTTTAATAATAAAAAGATGAATCAATAGCTATATTATAGTGTTCTAGTTTTTTTAACTTCAAATTAGTCCCTTACTATACTAAAAAAACTCTTCTGAAACTCCTAAAATTATCATTTAGTGTTCTAGTTTTATACAGTGAGTAATATGAAGTTAGTAAAACAAATTTGGTAGACTAAATTAAGGACTCATTACTTTTAAAATCTACGGCACAAGAGAGAGATAACTATGCAATTATTGAACATGACCAAGAAGTGTTTATAGGAACTATAAATGTTGTGTTAGAGCAAGGAAGATAAAGACGTCCTTGTCTTAAACAATTGTCCTTTTGAGATTTGTAGACGGACGAAACCATAATCATATATTTATCATATGATTACATTTTCCTTTTTTTTTTTGTTTCAAAAAGAAATCACAAAGTTAAGGTCCGGTTTGTTATAAGTTTTGATAGGTAAGAATAAGATGACAAAAGTTTAAATTGCAAATATATATGCATTATTATACCGCTGATAAATCATTTCATGATATTTTAATTTTATGAAATTATCTGTTTGAATTTAATTAAACATTAACATTTGGTTTCCATCTATCATCCTTTATGTTATCAATGACAAACATATTATTTTTATTTTCCAATCCAAAATATCAAGCCCAACATTTTCTCGCGAATAAAATTTAAAACTTAAAAGGGATTTGTAAAAATCTCAAATCAAAGCATATATATTGCCATATAATATTTACAAAAATCTAAAATAGGTTACCTTGTTTCTTTCTATAAATAAATTAGTAAATGTGAAGATTTTAACTAAAGACTTCCGCTTTTGAATTTTGAGATCAAACTTTACACCTAATCACCGATCGTTGGGTTAATGAAGCTGATTCAATATAATTATTATTATTTTTAAAATATTAAGGTGGCATAGTATGAGAATCCTTTCATTTTAAACTAAACTACTTCTTCCCTAATAATAAGATTCTTAGAAATAGAATTGTTGGAAAAACAATTGTAAAATATATATAGTTATTATTTAACTTTATATTCAATCATTGCAGAGAAGTTTTATTTCTATGTATTTATAAACATATGTGAAAGTTTCACTCACAACTTATTCATAAATTAATAGTCTCATTTATCAATAATATTTTATATTCGAAAAATATTTTTATTCTCTTCCACAAATGTAAAAAAATCTTAATTCTCATTAATATTGCCACTTAAATCGTAAGTATTTAGGTAAGAATTAGGCATGGATGTACGGAACCTTGGAATTGATCATCGTCCAAGTGTTAAAATAGAGTTGAAACCAACGCAATTAATTAAAAAAGCTGAAATATGATTCATATGTTGTTGCCAGCCACATCATTATATAAAGAGTTCACATTGACCCAAAATAAGCTTAAGATTGTATATCAGAATTATATGAAAGCTATTCACAAACTGCATAGAGGAAGGAGCACAAAGTTTATTCGGTCTGTAAGAACAGAAGGGTTTGTTTGATTCATCTTTGTCATCTGTCGGAAACAGGTTTCCTTGTTTTTCTGTTGGTAATGATGGAAGAGAATTCAGCAACAAATAGAACTCATGAGGTGGTTTTAAGTATGGACAAGGATGTCTCTAGAACAAGTGAAGACTCAGGCACCTGTATCACTGTTTCTTTCTTGCAGAAGGTGTGTCCTAATCTTTCTTCTGTGCCCAAAAATCCATTGAAATGATCTGTTTACACACTCTTTGGATTTGATTAGGTTAAATGGATTGTTATCTCTTAGACACTGAATGTTTATTGTTATTGTGGAATACTAGAGTATGTGTGTAGTATTTTTCTGAACTAATTAATATATTGATCATATTGAAGTTAAAAAATTGAGATTAGAAATGAGAAAATGGTTTTGATTATTGATGGATGCAGTTGGTAGCAGAGGTAGTGGGGACATATTTCTTGATATTTGCAGGGTGTGCTTCAGCGGTGGTTAATAAGAATAATGACAATGTTGTCACACTTCCTGGGATCTCAATTGTTTGGGGACTGGCTGTGACGGTCTTGGTTTACTCAGTTGGTCACATCTCTGGTGCCCATGTCAACCCTGCTGTCACCATTGCTTTTGCCTCCACCAAAAGGTTTCCCTTGAAGCAGGTGAACCATAAACAACAATTTTCCTCTATAACATTGGTAATTGGTTAAACTAGGTAGGAAATTAGAGGTCAAATTAGTCTATCTATCATTCACTTTTCTTGTAAGTTAAAATTCAGATACTCCTTGCCTATTTGCCTTGACCTTTTACACACTAGTTTATTGAAATTACCAAACAAAACACATCATAGAACATGTAGCTAGAGAAATACCGGATTATGTTTAACTTATTATTTTATTGAAGTAATGGCTTGGTTATATCAGTTTTGTAATGAACTTTTTAAACAATAAGCTGTTATTTCTAAGATTTATTCCTCTATTGTTCTATCCAATTGTCTTAAAATTATTAGCATTGGTACGGTGATATTGTATGGTCTATTTAACTTTGTAGGTACCAGTTTATATAGCAGCTCAGGTGCTTGGATCCACACTTGCAAGTGGGACTCTGAGACTGATGTTTAGTGGGAAGCATGACCACTTTATAGGAACACTCCCAACTGGGTCTAACCTTCAAGCTTTTGTGATTGAATTCATAATCACTTTCTATCTTATGTTTGTCATATGCGGGGTTGCCACCGATAACAGAGCGGTGACATTTCCTTATTCCATAAGTTTTGTTTTCTTGCTTCTTTGCTTATAATTAACTCATCTTTTAATACAATACCAATTGCAAGGTTGGCAAATCAGCTTGCCAAAATTTGACTTAAAAAAACTCTGCATTAATTGACCATTTGTCTCATATATTTATAACGGTTTGAAAATTTTGTAAGAAATAAAATTTAGGCCTGGTATTATAGATTTTTTATCCTGTTTTTTAATTAAAATTGGAAATTTATTTTGATAAGTAATATTGACTTTAATATTCATCAATTGTCAAGATAAGTATTTAATTTTAATTTAAAAAGACCAAAAAAATTATAATATTAAATCTAAGTTTTATCCCCCAAAGAATGTTCCAAGTTTGCTTCACATGATAGATTATATACGAAACAAAAATTCATATTTGAATATTTGTTATCCGTGGCTTTTATTTATTCATTTTATTTTTGTATCTTGCAGATCGGTGAGTTGGCTGGGATTGCCATTGGGTCTACGGTGCTGTTGAATGTGATGTTTGCAGGGTATTAACTTATCCCAAACCTAATTAATTAAATATAAGTGGCGATACACATTAAAAAAATTGCTTCATTCACGCTAAGGTGTGTTACATATTTTGGAAGCAAAATATAGGTCAAACTATGATCTCATTTGCGTGATTGAATTATATTGGAGCATAGATAATGGTTGAAATAAAGAATTTATTTTTAGGTCATATATTTTTATGTGTTTTTATTTTTATATTTTTCAGTTTAAATATTTTAGTACCTTTTAATAATATGGTCCTACAAATAATTGATCATTAGAGATTAGAAATGCTAATTCTGAAAAAATGGAAGATTAAAATTGTAAAAAAAAAATAGACAAACCTAAACAAAAAAAAAATTGTATAAATATAAACATAAAAAAGGAAAAGCATATTTAATCAAAATTTCTAACACTTCATTATCTCAGTTATTTACGTTTGTGCTAATGTTATGTAATGAATATTAACATGGTTGATTACATGACAGGCCAATCACAGGGGCATCAATGAATCCAGCAAGAAGCATAGGGCCTGCTTTTGTATACCATGAATATAGAGGAATATGGATATATTTGGTGTCTCCAGTTCTTGGGGCTGTGGCTGGAGCATGGGTCTACAACACCATTCGCTTCACTGACAAGCCATTCCTCAAAGGAGCTGCCTGTAGTGGTGCCTCCATGTGA |
| >CrNIP2;1  ATGGCCAACAAAGCTGAAGGCATCCAAGAAGAAGAAATGTCAAGAGTGGAAGTGGGTGTTAATCGCTGCCCTTTCAACTTTTCTGGCTTGCCTAGCTGTTGTTCATCAAACTATGTTGTAACCCTGACACAAAAGGTCAGTAAAGTTTTTTCGTCTCTCAAGAATTAACAATCGCACACATAAAGACACAATTTATAGGAGTAAAATAAAATGGGGGATGTATTGTTTTTTCTCACAAGGTGATCGCAGAATTCATTGGCACATATTTTGTGGTATTTGCTGGTTGCGGTTCTGTGGCAGTGAATAAGATCTATGGCTCTGTCACATTTCCTGGCATTTGTATCACATGGGGGCTTATTGTAATGGTCATGGTCTACTCCGTTGGTCATATCTCTGGAGGTCACTTTAATCCTGCGGTCACTATCACTTGGGCCATTTTTCGCCGATTCTCATACAAAGAGGTAATTTTCTTTTGTGGAACATTTTGAACTGCTGCCACTCATCAAAGTCAAATTTCACCTATCTTTTACTGAAGAAAGAACAGGTAAAAACTCAAATTGTAGAGATGCCTTGAATTTAATGGCTCGATCCAAATAGTAACTTGACCCGATATTTTGGAACCCAAAAATGATATGGACTAGACCCGGGTTTTTGTATTTTCTTGCATTCTGGATTAATTTATTGGGTTACAGATTACGGTAAAAATGAAGGAGTTCCGACAGGTTCATTATAACATTCGTCATATTTTGAGACTCTTCCTTTCCTCTAAAAGAAAATAAAAATCTCTTAACTGCCTCTAAATATATGTAGGTCACATTGGGCAAACCACATAATTCTGTGTTCCTTGTTCTCTCTCTCCTTTTCGTCTATCCTGTAACACAAGCTAATAACACTTCATGCTAATTACAGGGGAAAAGGAGTCCGGGTTTTTTTATAGATGTGAGCCTTCACTGTTTAAATTTCTAAATGAAGCTGTAAATTTGAAAAAAACAAACATTGATAGATCTTGATGTAATGGTTTGAATGCTTAGAATTACATGTGAACAGTAATAATTTTTAAGAATTATTTGATAATCCTTGAAATTGGGTCTTATTATGATGGCTTTGCGCATGTAGGTGCCAATATACATTGTTGCTCAGTTGCTGGGGTCGATACTTGCTAGTGGCACATTAGCCCTAATGTTGGACGTCACACCTAAAGCTTATTTTGGAACGGTACCAGTTGGATCTATTGGCCAGTCTTTAGCTGCAGAAATCATCATCACTTTTCTCTTAATGTTTGTCATATCTGCCGTATCTACAGATGACAGAGCGGTTTGCACTCATACACTACCTATATATGTGGCTTTAACAATTTCTCTTGATTTTTTTAATCCAAGATTCTCATAAGCATTTTCGCTTGGCTTGATCAATTGGCAGGTAGGTGACTTTGCAGGAGTTACAGTGGGAATGACTATAATGTTGAATGTCTTTGTTGCAGGGTAATTAGTTATGGGTTGATAGGTTTTGAATATTGGTTTTAGGCATGAGTTACAATGGTCCTTGAGGCTGGTGTTGGTTTCTTATTAAAATTGAAATTGGTTTTGCTTGCTTGCAGGCCCGTATCAGGAGCTTCCATGAACCCTGCAAGAAGTATTGGTCCCGCGCTCATCAAGCATGTTTACAAAGGGTTATGGGTGTATATAGTTGGTCCGATTATTGGAGCCATAGCTGGAGCATTTGTCTATAACTTTCTTAGATCCACGGAAGAGCCACACTCTGAATAA |
| >CrNIP2;2  ATGGCTGAAAACCAGATCACAGGAATGGAAGAAGGTGGAGTCCAGTCACAGAAGGATTCTAGCTTTCGTGATTCTCCTGATGTAGTTCAAATTATACAAAAGGTATATTCATATCATTATCTACATTTTACTACATAATTAGTTTCAAACAATTATTGTCTCACTAAAGTATGTAATTTTTGTTTAGTTTATATATATTATTTTGATGCATAGAGAGAAAATTGTTATTACCAATGACCAAAACAAAAATAAAAATTAACGTATGTGTAACAGGTAGTTGCAGAGGTGATAGGGACATATTTCTTCATATTTGCAGGGTGTTGCTCTTTGGTTTTGAATAAAGTTGAAGAGAGTAAAGGGTCAATAACGTTTCCTGGAATTTGTGTGGTATGGGGTGTAACCCTAATGATCTTGGTTTATTCTCTCGGTCACATTTCTGGTGCTCATTTCAATCCTGCAGTTACTGTTAGCTTAGCCATCTATCGCCAATTCCCTTTAAAACAGGCATGCCATTAATCACTTATTCACTATATTTTATTTGGTTATTATTCGTTTATTAATTTGATACACCACATATTATTAAATGAAAGGAACTCCTAAGATATAAATATAGTTGTTGATATTTTAATTTGAAATTGTGTGCACAATTGTTTTTTTCAGGTGCCTCTATATATTATTGCACAAATAGTAGGATCGGTCCTTGCTAGTGGGACATTGGACCTTCTCTTTGATGTAGATGATAATTCTTATTTTGGAACAGTACCAACAGGATCTTATACTCGATCTCTTGTTTTTGAGATACTCACAACATTTCTCTTATTGTTTGTTATTTCTTCAGTTACCACGGACAATGGAGCGGTTAGTATACTCAGTTAGCTATAGTTTTATTTTAATTGTATCATGTTTTTGCTGCAATTCTTTTCATTTTAACACTCTTGTTCTTCTTTATTGGAATTAAATCACCTATTTTTTTTATGTCTTCGTCCTCGTTCTTCTAATTAAGAAATGCCACCTTTCTCCTTTTTAAAAAAAAAAACTTTAAATAATAATATATGATCTCCTTAAGTTGTTTTTTTTTTCAATGAATTGTCATTTTGTAATCTGCGAGATAGGTGAAAAATATGTCCAAGCTATAAGAATTTTTATAGTGGAAAATTGCTAAATGGTGGGTGGTTATTTAATGAATCATGATATAACACTTGTTTATAATCATATTTATCATTTTAAAAAGTTCCACTTATGAAAATGTCATAATATGTTGATATATACTCTCAATATCCCCTTGTGTGAAATAACTATAAGTTCCCATTTTTAACAGGTTGGAGAGTTGGCAGGTGTGGCTATTGGTATGACAATCTTAATAGACCTCTTCATTGCAGGGTAATGTATTGTTATGTGTGTTTGTGTGTGTACACATACATGCATGAGAAGATATTATTATTTACCACAAGTGTATATATAACATCATTTTAACTTAAGTTTGATAATGATGAACAGAAATGTGTCAGGAGCATCTATGAACCCAGCCAGAAGTCTAGGACCAGCATTGGTGATGCATATTTACACAGGATTTTGGATTTATATAGTTGGGCCATTTCTTGGTGGTATATTAGGTGTCACAGCCTACAATTTGATTAGATTCAATGAGAAACCACTAAGCTCAATCAGAAAATAA |
| >CrNIP3;1  ATGGAAACAAATGAGGAAATTCCATCAATGCCTACAACACCAGGCACTCCTGGTGCACCTCTTTTCGGTGCCTTCAATGACAATCATAACAATAAGAAATCTCTCCTCAAGAATTGTAAGTGCTTCAGTGTGGAAGAATGGACGATTGAAGATGGAGCGTTACCAGCTGTATCATGTTCATTACCGTTGTCACCTCCTCCTGTGTCTCTTGCAAGAAAAGTCGGAGCTGAATTCATCGGAACCTTCATTCTCATGTTCTCTGGCACTGCCGCTGCTATTGTCAACCAAAAAACACCTGGCTCCGAGACTCTCATTGGATGTGCTGCCTCCACCGGCCTCGCCGTCATGATCGTCATTCTCGCCACCGGCCATATATCCGGCGCTCATCTCAACCCTGCCGTCACCATTTCCTTTGCTGCATTAAAGCACTTCCCATGGAAACATGTATGTATATATAAAATTCATTTTCCTTCTCTATATAATTTTAATTAATCGATATTCATCCTCTTAACTTTAATTTGATTGAGCTACGTGAAATTGATTATAAAATATTATTAATTGCAGGTGCCTATGTATATCGGTGCTCAGGTTTTGGCATCTATATGCTCTGCCTTTGCTCTGAAAGGGATTTTTCATCCTTTCATGAGTGGTGGAGTCACCGTTCCTTCCGGAGGATATGGCCAAGCTTTTGCTTTAGAGTTCATTATTGGCTTCAATCTCATGTTCGTTGTAACTGCAGTCGCCACCGACACCAGAGCTGTCAGTATTTTATATCCTCCTCTATATTTAACTTCATTTCACTTAATAAATAAACCAATCGATTCTAGCCACTCAGGAAAACAATAACATGATAAAAGAGACAAAACTTCACTAATTCTATATTATTATATCTATAGTTCTTTCTTTTCTTTTAGGTAGAAATATATAGTTCTATAATATGCCTTTTTATATTAATTATTATATCCTTGTATATTATATTAGAGTAAAGTAGTTATTTAGAGTGTTTTGATTTTTTTTTTAATTTATTCTTTGCAAAAAATAATACATGCTATTTTTAAATATTTTGAATTAATTAATTAATTATATAATTTTAATTTGTCGTTAATTATATACTTTTTTTCATGATTTTAAATGTTTTTCAATTTTACCTGAATTTAAAAATACACGGTTATAAAAGAACAATAGCCCATATAATTAGTTGTCCTCTACACCATGCTAGCTTGTTTTCAATGAAGTAACTTATGCCGAGGAATTTTTTGTATTAGACTTATCTTTAATGTAAGCAAGCTCACTTTAAAAGTATAAATTCGTAATTTTAGAAACAAGTTCACTTTAAAAGAATTTTATCTTATTATAATAATACAATAATAACAATGATTTCTCGTTATCTAACCATTTAGTAATTAGTAGACTTCAAGCTAAAGGTCTGTGCTTGCTCCAATAGTAAATCACAATAATTATGGTTATAACCCTTATATAATTAAATCCAATATCAATTAACTTACTTCCTTAAATATTGATAAACGTAATTATCATATATTTTAATAAAATCAACCCAAGCAGGGAGGGGCGGTGGCATTGGGCGACATATCCAAAAGTGTAATTGGTGTGAGAGTCAAAAGGAAAAGATATGTCCGCTGTTTTGTGCCAGTAAATCTACATGAGTTGTCCACGTTTACAAAAACCAATAGGATTTGGAATCTTGCTGGTTGCACCAGACAATAATCTTAGTTTGGTCAATGCAGCAGCTTTAATTAATAATCAGGGACAGATCTAAAAACAATTACCAACAATAAATAAAATTTTAACGTATATGATTGTGTTAATATATTAGAAGATAAATGTAATTGAAGAGTTGCGAGCGCTCATTTCGTTCCTCCTTCCATCCGTTCCCGTTAACAATTCATAGAAGAAATGATGAAGTTGGTCACCAGAATTGCGGAAAACATATTTATTTTTAAATTGCAAAAACCTGATCGTGATTCTTAAACGTTAGATCCATTGACTATGAGTCTGCGACTAATATGGTTTGTAATTTTTTTCAAAATTAAAATGATAGGAAGATCTGCATGTGTTGTGTGTGTGAATGGCATGTGCAGCATCAGCATTTCACTGTGCCCTGATTGATGATTAATATGGTTATGCAGGTGGGAGAACTCGCGGGAATCGCGGTGGGAGCAACTGTAATGCTCAATATACTCATTGCAGGGTACGACTCAGCTCACCACTCACCGTTATCTCCTAAACACAAATTTCCCAAACTATCAAGTTCTTCCTTAAAATTTAGAGGAATATTTCAATTTTATCTTTCGAATTTATATGTATTACTACGCTAGTTTTTTTTAAATATTTTTAAATTAATCTTTCAAATATTTTTTTTTCATTATTTTTATTCTCTAATTTTTCCCATCAACCTATTTTTTAAAACAATTAATCTATCACCGAATTAGTTCTTTAACTTTTCTTATGAATACTTCACTCCTTCAAAAGAACTATGGTACAAGTTATATATATATACACACACACACACACATTTTAAACATTTTTTATTAGACCTTTAACTTTGTTTCACTACTTTAATCCTCCATAATAATTAATAAGACAAAATTTAAATCTTCCTAGAATTTTTAAAGACTTATTTAATGATATATATATATATATATATATATATATTTTAAACATTTTTAATTAGACATTTAACTTTTTTTTCACTATTTTAATCCTCCATAATAATTAATAAGACAGGATTTAAATCTTCTTAGAATTTTTAAAGACTAATTTAGTGATAAATTGGTCTTTAGTTCGGTATAAAAAAGCTAAATTCACCCGTCTCTATTGTGGCTTTGATTTCGGCTACTAAACTTCAGTATGGAAAAAAATAAATAGAAAAGAAAGAGAAATAAAAAAACAGGTCACTCTAAAGTGTATATAAAATGTATATTTATCATTAGGGCATAGTAATTTTGGACTTCTAAAATTTCAGTGTGAAAACGCCCCGTGTTTTTCACTTTAGAAGCAAAGTCATTTTAGAGGATTTTCTTCCGAAGATATATGGTTTTATTCTCAGCCTTATGGTGTGAAAAGAAATAAGTGTCAATTAAAGTTCATTTCATTCCTTCAAGTTAGCATTTTTAAAATTTTCCCAGCATCACCCTGATTGAGAAGGATTTTTAACGGTCAAAAGAACTAATTTCCAACAAATTACAGTACACAGTAGAGCTATGAAAATGGTCCGAGAAAAGCAGACTTTGATAAAATATTCTTAACCAGCAGACTTTGATAAACATTCTTCACCATGTATTGCAGGCCAGCTACGGGAGGGTCAATGAACCCAGTGAGAACACTGGGTCCAGCTATCGCCGCGAACAACTACAAAGCCATATGGGTCTATCTGGTGGCTCCCGTTCTCGGAGCTTTAGGGGGGGCAGGTACCTACACTGCAGTCAAGCTGCCAGAAGAAGATGATAACGCCAAGGCAAGGGCTTCAATCAGCTTCAGAAGGTGA |
| >CrNIP3;2  ATGGATAATGCAGAAATTCCATCAGTTCCTTCAACACCTGCTACACCAGGAACTCCTGGTGCTCCTCTTTTTGGAGGGTTCAAGTCTGAGAGAACTGGGAATGGTATTGGTAAGAAACCATCCCTTCTCAAAAGTTGCAAATGTTTCAGTGTTGAAGAATGGACCTTAGAAGATGGAACCTTGCCTAAACTCTCTTGCTCTTTGCCACCCCCTCCTGTGCCTCTTGCAAAAAAGGTATATAATTATATATACTAATGAAAATACAGACATATGAGAATTTCAACCTGTTTCCATTTTAAGCAAAGTTAATGTTGTTGATTTCAAAACCAGGTTGGAGCTGAGTTTATTGGCACCTTCATTCTCATGTTTGCTGCAATAGGCACTGCTATTGTGAACCAAAAGACACATGGCTCTGAGACTCTGGTTGGATGTGCTGCAGCTAATGGACTTGCAGTCATGATCATCATTTCCTCCACTGGCCATATCTGTGGTGCTCATCTCAACCCTGCTGTCACCATTTCCTTTGCTGCATTAAAGCACTTCCCGTGGAAAAATGTAAACAAGCCTTAATTTAATTTGCCTTTTTTTTTTAACACAAAATCCAATTCAGTGTTATAGGTGGTTTAAGTATTATGTTTAAATTAAAACTAGAGTTTCATTTTAGTAACCTATGCCAATTTTTATTGTTGAACTTTAATGTATGGGTTATGAAAATGAAACATTGCTTAATTTTTTGTCATTTTGTGAATTAATTGCAGGTGCCAGTGTATATAGGTACACAACTTTTGGCATCAATATGTGCTGCATTTGCTCTGAAGGAGGTTTTTGACCCCTTTATGAGCGGTGGAGTGACGGTCCCTTCAGTAGGATATGGCCAAGCATTTGCTATAGAGTTCACTGTCAGCTTTATTCTCATGTTCGTTGTCACTGCCGTCGCCACTGATACAAGAGCTGTAAGTATCTTATTCTGTTAACCACTTTAGTCTCATAATTAAGTAGACCGAATCGGTTGAATTGAAGTATGAAAAAGATGTTTTATCATCAACAAATCTTAATTATGTTCAAAGAGGATGTGAGAAGTGTTAAGTCTTTTTTGTGTGGGGGAGAGTTTAATACAAACTCTAGAGAGTATTTTTGTCTATTTAATTTCTCGGTTTCTATTTGCTTTTGTGCATATGATAACCATCTATCAGATGAAAAGTTTTGTTTTATGGAAAAAACTTTTGATTGATACAAATATAAACATGCATCGTTAAACTAATAAATAAGTGAATGATAGTGTTTATAGATGAAAGAATTATTATCATGTTTTATTAGTGCATGTTTTCAATTTTCAAAGGACATAGGTCCATCAAATTAAATAGGAGGTCCTCATGCTCTGCAAGTCTCGTTCTCAATGAAGTAAAATATGATATTAACAACAATTTCACGTTGTGTCTCCCCAACTAGATCCATGAAGCTCATCATCATTCTGCATGTACCAAGAATCAAACATAAATTAAACTGGCCTTTAAAAAAAGGAAAATTATGAGTGTATATTCAAAATAAGTGAATACATTCAAAAAATGGTAAAATGATAGAGAAGATGAGGGATATAATGCAAAGAAAGAGAGATAAAAAGAAGAATAAAACAATAAAAATTAAGATGGAAAAAATATATATTTATGTTGTTCTGAAAACAAACATAGAGCATTGATATTGTGAGAGTTGATGAAACTCTAAGCATAACTTTATTGACTAATTTTTTTACAAATATAATAGGTTGGCCACTTTAATAATCAGTAGTATAAATAAAATTTATAATATGTTTTAATTTATTAATCAGTATATTTGTTCTTTTAGAAGGTTAGAGTGGTAGCAATAAAAAATTAAATAAAATGGAACTTGTTTTCTATTTTAAATTGATTATCTAGGGACAAAAATTTTATAAACTAATGAAATAATAAGCTGTTATTCGAAGATCAATTAAAGATTTATTCTTAAAAAAATGCAACATGGTCACAAAAGAAGATGTGATGTGATATATTAAATTTTAGATAGATAGATAGTTGGTTCAGAAAAATCATTAGATTTTAACCAATAAATGATCTAGGTCCTCTGCGTGCAAAAGAATTCAGTTGATTAAAACCTTCGAAGTGTATAACTTTTCCCCATGTAAATAATTGATTGATGAAAGATGAAATCCTGACATCACAAGCGGTAAGTGTTTTTTATTCATGAACATGGGAAAACAATATTTTGCTTCATATATATTATTGCGGATGTCATTTTTACAATAAATAAGGATTTAACTACATTTTTCTTTTTGAATTAAAATATAAACGATTTTTTATTTTTATCTTTATAAAAAAATATTTGCTAGTTACGATATTTAATAGCAATAATGTGATTTTAATCTTTTATCAGACTAAAAGTACATGACTTTTATAATTTTTAGAAATTGAAAATAATTATGTTATAATGAAAAAATAAAAAACATCATTATTATTTAAAGACTAGAAATTTACTTATGATATAGATCAATAAGTTTCCTCTTCAATATAGTTGAGCCAAAATAATTGGTTCTCTCTTCCATTGTGATTGCTGCAAATAAAAGGAACCCAGGCAAGGCAGGTGCCCCTTTTGCCAATAATCCAAAGAGAAATTATTCTTGTAAAAAAAATATAAAAATGTAGACAATTATGGAAGACAAAAAGAGAAAAGACACAGATCACAGTAAAAGATGAATAATAATAGAAAAATAAATATAAAAAACAAGTTTAATTTAGTATACATTTTTTTACATCATTAATTGGTTAGAAACTATAGTAAAATAATTTCTCATAATTCAAAAGTTATATTTATCATATTTTCAAATTGATTGATAGTATTAAAAAAAATAGTGAAACTGTATCAAAATTAAATTTTAGTAAATAACTCTAATTCAAAAGGTTATGATTGATGCCACGGCATAGTCAATAAATGTTAAATCATGGCACTTGCTGTTCATTTGCTACTTCTTGTTTTCTCTGCAAAAACAAAAGACATGAACCCGTTTTGCAGTGAGATAACGCTGCGCCTACGAGTGAAAAACGTCAGAAACAGGATTTAGAATCTTGGTAGTTGCACCCAATAATAAGAATTAAGCATAGTTTGTTCGAAGCAGCATCGATTCTTATAGAATATAAGAACGGTAGATATACACATAAAGGTAGAGGTGATTGATTTTGGCATGTTATTATGCTGCTGCAGGTGGGAGAGCTCGCAGGAATCGCGGTGGGAGCCACTGTCATGCTCAACATACTCATAGCCGGGTATGCTATGCCATGCATTATTCAAATAATTCAACGGCATTAACAGGGTTATAATTTACATTTTTTTTTTCTCAACAATTCAGATTTTACACAAATTTAGGTATACATATGATATATGTATATACAAATATCCAGTAAAGTTTTAATTAATCTTAACATACAAGATTCATTCTTTCCAAAAAAAAATATATATATAAATTTTAATTGAAAAATATTTTTGTCACTACAATTGGGAACGCTTTAACACGAGTTGGTTTAGAGTAAGGTAGAATGGGTAGGGAGGGAAATTGAAGGGGTTGGTATGAAAGGGTTAGTTTCAACATGTGGTCCCTGTTTGAATTGGATTAGCAAAACATAATAAGTAGAGATAAGAAGGAACAATATTATGCCATAAGGTATGAAAAAGAAACAAGTATTGAGTGGAATAATATTCTTGTAATATGCGTGACCTTGCTTAATTGATGGTGTACTGTACTAAAGGCCAGCAACTGGAAGTTCAATGAACCCTGTAAGAACACTAGGTCCAGCAATTGCTACAAACAACTACAGAGGAATATGGGTCTATCTCACTGCTCCAATACTTGGAACTCTATGTGGGGCTGGTGCTTACACTGTTGTCAAGTTGCCTGATCAACGTTTTAATTCTCAGGCAAAGGCACCTTCAGCTCCTTCCACATTCACTAGGTGA |
| >CrNIP3;3  ATGCCGGACGAGGAGATAGGGACGCCGACGGCCGCGTCGGTTCCGGCAACGCCGGATACTCCGGGAGGACCACTATTCACGTCGCTGCGAGTTGACTCACTATCACACGAACGTGACTCGTTTGCAAAGGCTCGGTGCAAGTGCTTGCCGACCAAAGGTCATACCTGCTTCACCGATTTCTCAGTTGGGGTTCCAATTCCCAATGTATCTCTCACTCAGAAGGTAAACAACCCACCCCTCTCTCTCTCTCTCTGTTTTGGTCAACGATTTCTATTTCATTTTTCAGAATTTTTTTCTTTCTAGAGCCAGGTATATACATTTGATTTCCAGATAGAATTTTCATAATTGAGTTGTTTTTTTTATGATATTGATGAAAAATGCATGAAATGGAATATGGAATAGTAGTAAAAAAAATGCAAAATAGAAGTTTTCGTTTTTCTGTTATACGAATTCGAAGGCGGCGTGCAATTAATATTCATTGAGGTGTGTGCAATCATACATTGTCGTTATTACACAAATAAACGAACGCGCGGTCGTGTATAAGATTAAGAACAGCTAGGTGTCGCCTTTTTATTTTTATTTAAATATATATATCTTTTTCGTTTTTACATTTTTACGGTTTTTTATTTTAATTTTTAAAACATTTTTATGAGTTATGTTTTTTATCTTTAATAATTAATAAATAATGATGTCTTTAATAAAACAGTTGCTATATATCAATGATATGTAAGCATGGTAGTGAAAGATTAAAATATTTAAATTGTAGAAATATAGAGATAAAAAATCTGAAAATAAATCGGAATTATTAAAGTAAAAAAATTGTAAAAGTATATTGAAGTTTTTAATTATTATAATTGTTTATAAACTTATCCGTAGCAACTAGCATGTATATTTGATATTTTATTATTGTTCTTTAATAATGTCTCTCGTTACAATTTCTTATCTTCGATGCTTTTGAAACAAACAAAATTATGCATTAAATTTTTATTTTAAAAATATGATGGATCAATTTATTTTAAAAATATTTTTTAATAAACTATTTAATTTAATATATTTTTAAAATATAAAATTATATGTTTATAAATTTTAAAATTTAATATATATAATCATCATATTAATAACTTATTATTTAATGATTAAACATGTTAAATTGACCTTTTTATATTAATAGTTTTAGTAAGCGGATATTATATATATATATATATATATATATATATATATATATATATATATATATATATATATATATATATAGTTAGCAGGTCGGATAGTAAAATTACGAGTTAGGAAAGTTTAGATAAAAATATTATTGTCTATTTAAATGTGATTTTTTTACTGTTTAATTAAAATTGTAAGTTAGATGAATTTAAATGTTATTGATATATTTAATATTATAAATTAAATGAGTTAAGTGGATAAGCTTGGTTGGTTAATAAATTCTTTTTTAAATAAAATTTATTTTTAAAATATTTTATTAATTTTTATTTTTTAAATATTTTTTAATAATTAAATAATCTACGAATTAAAATAGCTTAAATGTGAATTATATTTTTTCTAATCCATTTATTTTATATATCAACTCAACCTAATTTGTTTATAACATATCAATATATCAGACTTAAATAACTTATGTTGATTTATATATCCAACTAAAGATTCATTTATTTTAAGAGTAATTTATTTTAAATTTATTTTGTTATATAATTTTTTATAAAATATAAATTTGATAAATTTAATTATTAAATAATAAATATTAATATGATGATTATATATATATATATATATATTAATTTTTTTAAAAAAATGAATTTTTCAAGTATATAATATTATAATTTAAACTAATATTGTTAATAAGTTATTCAATAACTTTTAAAATTTTCAACACTTAATTTTATAGTTGATATTTATTGATATTAAAAAAGATATATTATAAATTTTTAAATAATTTGAGTATAAAATACTGTTAATGTACCCTTCCCTTCCTGTTTGTTTATACATAGGTAGTTAAGGAAGATTTTTTGGAAGGTATTTTATTTGGTACAACTATAAATTTAATAGTATGATAATTAATAGTGTAGTTTAATATATTTATATCATGTATTATATTTTTAATTATTATTATAATTAATATGGTTAATAATTTTGTTTATTTTAATATATTTATTTTTAAAAATATTCTAAAATTATAAATTATTAACAAATATTTTTTTTATACCTTTCTAAATTTAAGGATAGATGTTATGCATTATACTTGTAATTAATACTAGCAGGATATAATTTAATTAACACAATCAACAAGTTTGCATATTTTCATATATTTATTTTTAAAGATATTATAAAATTATAATTTGCAAACTAACCACTCTAATAATTATTAATAAATTTTAAATTATATTAATTTTTAAAAAATTATTATTTTAGCAACCGATTTCTCGATTATAAAAGAGTTTTTTTAAAATTTAAAATTTATAAACAATTAGTTAATTAATAATTATTAGAAGGATCAATTTGTATATTATAATTTTAAAATATCTTTAAAAATAAATATATTAAAGTAGATAAAATTATTAACGGATAATAATAATATTTTGATATTAATTACAAATATAACATATCATATAAATGTATTGAATGACAATATCCCTTATATCTAACCGTATTTGAGATACGAATAAAATACCTTTTTGGGATTAGATTCGAGAAATAAAGGTGAAAAAGAGAAAGGAATTGAATGTGTTGAAATTGAAATGGATATTAAAGATGGTTAGGATATATGAAAACAGATTGGAGCAGAGTTTGTGGGGACATTCATATTGATATTTGCATCAACGGCTGGACCGATAGTGAACAACAAGTACAATGGAGCAGAGGGTTTGTTAGGGAATGGAGCTACTGCAGGATTAACAGTAATGTTCATTATTCTCTCCATTGGCCACATCTCAGGTGCACATCTCAACCCGGCTCTCACCATTGCATTTGCAGCTTTTCGACATTTCCCTTGGGTCCATGTCCCTGCTTATGTAGCTGCACAAGTCTCTGCCTCCATCTGTGCTGGTTTCGCTCTCAAAGCTGTTTACCATCCTTTCCTCTCTGGTGGTGTCACCATCCCTTCTGTCACCATTGGCCAAGCTTTTGCAACCGAGTTTATTATCACTTTTAATCTCTTGTTTGTCGTCACTGCTGTTGCTACCGATACTCGTGCGGTGAGTCAGTCAGTTCATTCATTCATTCATTCGTTCGTTAATTCCTTATCAATTTCAATTTCACCATTTCTATCATTCTTCCTCCCATTCAGGTTGGTGAATTGGCAGGTATTGCTGTTGGGGCTACAGTTTTGCTCAACATTCTCATATCAGGGTAAGGATATTGCTCTTGGAAAATCAACACTACTCACAATCTGTCAACTCTCAAATATGTTAATTTTCAATTTAATAATGATATTATAAAAGATTTTAATTTTAATTTATTACTTTTTTTCATCCGTAAATCCTAGTATTATATGTATAGGCATAGCATATTAAACCTTCAATATATTTTTATCTCTACCGATTATGAGCTTTTATTTAATTTTTAATTTTAAAAAATTGTAAAAATTTAGTTCATCCAAAATTTATCTCTTTCTTATATCAACAATGATTCTTCTGCACATATGAAAAGTATGTTTCACCCATATATCCCTAATTTAATGATAAGATATTTTAAAAAGGAAAAAAAAGAAAGAAAAGTCAATTTTAATATATATATATAGTGTCCGGTAACTTCTATTTTATTTTGTTAGTTAAGTATCAATATTTTAATATTTTTTACTCTTTATAAATATTAATATATTTTTATTCAAACTCTCTAAATTAAATATCATGAGTTTGTCAGATTTTAAATATCAAGAATTTGAGAAATAGCCAACGACTAACGAAGAAATTAAACCACATGCGAAGGAGAAGAAATTCAGGAGAGCTAATTATAAAATTAGGAAGTCGTTTGGTTTATATTTTTTCACCTTTATTATAAAAATGTTTTTTTAAGTTCCACAGAAAACAATAAAAACAGAAAAAAAAAGCTGATATTTTTTAAAGCTCATATTTAATAGAGAAATTAATTTTCTCCTTGAATTGCATAAATAACAAATATTTAAGAAGAAAGTAAGGAGGATAATGAATGAATGGCGTGACATGGTTGATTAGGCCAACAAGCGGTGGTTCGATGAATCCGGTGCGCACCTTAGGTCCAGCAGTTGCAGCAGGAAATTTCAAGCATATATGGATATATTTGGTGGCTCCTACGCTGGGTGGACTCGCTGGTGCTGGCGTTTATACGCTTGTAAAGCTGCGTGACAAGGATGGTGAACCGCCGCGACAAGCTAGGAGTTTCCGTCGCTAG |
| >CrNIP4;1  ATGGCAGAGGTGGTGGGTACTTTTATTTTGATGTTCTGTGTATGTGGAATCAATGCAAGCACACAATTCCAAAATGGTGCAGTGGGCCTTCTGGAGTATGCAGCTACAGCAGGATTAACAGTGATAGTGATAATTTTCTCTATAGGGCCAATATCTTGTGCGCATGTTAACCCAGCTGTCACAATAGCCTTTGCAACAATTGGTCAATTTCCATGGTTCAAGGTGCTAATAAAAAAACAAGAACGTAGCCCTTGTTATAATTTCTGGTACTGTAATTGATTAGTTCATGACTACATACAATTCCCATGTTTCTTCAACAGGTACCAGTTTACATAATAGCACAGACAGTAGGTTCTATGTCGGCAACATACATAGGTAGCCTTGTGTATGGCATAAAATCAGATGTTATGATGACACAGCCACTCCAAGGGTGCAACTCTGCCTTCTGGGTGGAGGTTATTGCAACTTTCATCATCATGTTCCTCATTGCTGCTTTGACGTTTGAATCTCAATCAGTAAGGACTAAGGAACAAAATTACTTATTTAATTAACTTCATTCAAAATTAACTATCTAAATTCCATTTCCCTTTTACCTAAAACTAAAAACACTGTGCAGGTAGGCCATTTATCTGGTCTAGTAGCTGGAATAGCAATTGGGCTTGCTGTACTAATCACAGGGTACCTACCCACAAACCCTCTCTTTTTTTTTTAACAAAAAAAAGCTTAACAAATCTTGTTTCAGTGCATGTTTGAATTAATTTTTTTTAGTTAAATAATTGTTTGTTTTTAAACATTCTTATGTAAATTTAGTGCAAATAAACCATTATTTTCTCAAAACTAGCTGAATCAAACATACTTGATAAATTTGGATCCTTGTATATTATTGAATAACATGATATGGTCATGTTAAAAAGAAAAAGATATAATTTTCGTCAAACTTAATTGATATCATGTTACTTAACAATATGAGAAGATCCAATTTTATTTTTATTATTAGCCTTTTATTTTTCTAATAACAAAGTGGTTTGTTTTCAATTTTGTTTAACTGCAGCCCTGTCTCAGGTGGATCAATGAATCCTGCAAGATCTTTAGGTCCAGCAATTGTGTCATGGAAATTTAAGGACATATGGATATACATCCTAGCTCCTTGTGTTGGAGCTGTAGCTGGAGCTCTAATGTTTCATGTTCTACGTCTTCGAGAACAACATTGTAGTCCTTTGTCCTCCCAAAACATTAGAGATGTTGGTCGTCCCATACCCTTATGCTCAAGTTAGTGCACCAACATGAAAATGCAGAATCAATAGTAATCATTGCCTATTAGCTAGCTTTAGAAGCTGAATTCTTCATTGTTTTTTTTTTTCTTTTTTCTTTCCATAGGGAGACGTGGGCCCATGATTTTGCTGGTAGAGAAAATTTGAGTTTATTATCTGAGAGAGTAGAAGGGTTTAGACAAAGATATATTTTAAGAACAGAAGGGATATCAGATGTTGTTTATCATAAGTTGCCACCGTAATTATAGTAAACTTTGTCTATGTTATGGTTTAAAAATATGCAACAAGATGCAAACTCAACAATGTAATTAAGATAGCACCATATAATCATACCAATCCATATATTAAAATGAGCATTGATAATTTACCACGTGTATAGAATCTAAAGGGTATGTTAGGTACTAACTAAATGGCAATTGGAGTTGGTTTAATAAGAAAATTTTGAGTAATTTGTATGAGATTTTATATTAAATTTTATTATTTTATTATACATAAAACAAATACTTGTTCGTGGTAATTTTTAGAAATAAGGTATGTAATGTGATATATAGAAGTTATTTAAATTTTTATTTTTTTTAAAAAGGATATCCTATAATTAGAGACTCTTTTTATTTAATTCTTTTTTATCATTTTAGTTTGTCATTTGTTCAATTTTGATCCTATACTTTTCATTGCATAGTTTTTGTTTTTTAATTTTTTTAGTTAAGTAAAGGTGGTTCATTTGTTAATTTATCATCCAATTTTTAATGATGTTACTAAAAATTATTGGTCAACTAATGAATAAGATAGTTAAAAATTGAAAATACAGTAGATGTTGAAAAATAAAAATTGAAAGCTGATAAATTATTGATTAAATACATAATCTTTAAATGAAAAGGATAAATTCAATTAATTTTATCCGTATTTTAAACCTGGATTACGTTGTTAACAATATTTGCAAAAATTTTATTTTATAAAGCATATTAATATTGAATTCTCGTTATAAAAATAATTAAATAAATTCTCATTTTAAAGTATTTTGTCTAATTTTTTGATGCAGGAAGTATTTGTCTAATCTAAATACAAAAAAATATTCCTATGTTTTGCTTCATTAAAAGAATGGAATTGCTTGAAATGAATCCGAATTTGTTTGATTTGCTTAGAATTTTTTTTTTCAAAACTCATTCTAAAATATATAAATTGAATTTGATGATATGTTTGGAACATATATTAAAAAAAAACCTGTTTGATTATATTTTATTAACCTTGTGAAGAATTCATACATTAACTAAATTAATAAAAAAACAAAATGACCAATTAAGATAATTAAAAATAAAACAAAAGTAAATTCAATAAAATCAAATACATTATAAATTTGAATCTTCATTTTTAAAATGTAAAATTATTTTATTTTATTTTCCTTATGACCCGTGATAATATCATTATTTAATACTTATTTTTACAATTTTAAAAAATTTTCAAATTGTTATTTTAAAATTTTTTTACAATTAAAATTTTCATTCATATTTTCTTTAAATCTCCCATTTATTTACTTCTAATTATTTTTATATTAAAATTTTCTTTTTCATCTCAAATCTCACCATTTTATATCATTTTTTTCGAATCAATATTATTTTTAAATATTTTACTTCTTTCCTTCCAAATCTAAATTACTTATATTATTATTTATTTTTGAGTTAATTACAATACTCATTTAAATTTAGATTTTTTTTTTTTTGTATTTATCAAGGATATTCTTTAGCCGGTAGCTAAATACTAATCTCTGTGAGATATAGAAATCATTGAAATAGATTTTACCTTCTTTGATAAAATCTTTCTTTATACATACGATTAAAATTAAACTCCCATTAACATGCTTAAGAAACAAAACTATCTATCAATAATATTGAACTTTGGTAATACATTTTTATTTAATTTATATCATAGATTACATTTAAATTAAAATTATACTCCATGCATTGCCCAAAACAAAAGATATTTGCTAGCTAAAGTATATTCAATGTTAACTAGTGTTTCAAAAATAATGGTTAAGAAAACAATAACACAAAAATTATTAACATATATTTGTTATATAAAAAGTACAAAAGTATTCAATACTTATTTTTTTTACATAGTTTAAAGCATATAATAAAACACATCTAATACCTTTCTTAATCAATATCCTTAAATTAATCACTTATTAACAAAAACCCTATCTTACATGCATATACACAAAACAGTAAGAATTTTATAATTAAATCAATTCAAAGGTGTACGCCCAATGAGTATAAATGTATAATCCATTTATACACTCTTAGAAGAAAATATGTTTTATTTAATTATATTTTTGAAACTTCAAAATAATTTTAATACAAATTAATAAAGATATTAATTATAAATTTTAATTTATGCATTATAGTATCTTCTAAAATGGATACATAAAAAGGAAACTGAGGATGTAGATGTAGTATAAACCACTCCCATGCAAAGTGGTAGATCACGTGTCAAGAATTTCCTTCAACGCAATAACAACAAAATCAAATTCTTCGGAAATAAAGAACCCAACAAGACAAAGTAATTCAGATCTCTTATAGCTCTAACAGAGGCCTCTTTCTTGGATTTCTAAGAGAAATGCCGAAAGGTACGTGAAGTGGTGTTGTACAACATAATTCAAGTGCCATACATGACATCATTGTGCTTAATGTTTCTTTTCTTGATATTGATGATCAATGTGTATTCCTCATGCAAAGTTAAAATTGTTTTCATATAATTACCACTAATTCCTCATTATCAATTAATCCTCAATTAATCTTGTTATTCAGAGTATAATGACAAGTCCACCTTCACACAAATCGTCGTGGCCTCCTCTATTGGGTTGATTTTGGCTGCAACAATGCATTATAATGTTAAAAGAATGAGAGATCGAAAGATTGTTCCACGTTTGAGATTTTCAAAGACAAGACAAATTCCAAAGCTTGAGAAGTTCTCTCATTACGTAGGTAATGATATATAAAGATCTAATTTTAATATTTTTTAACAATAATTTTTACAAGAACAAAAGTAAGAGGAGTGCATATTAAGATATTGGTGATATGATTGAGAAATGGTGATTATATATAATCTGAATTGGATTGGTTTGATTCATACGTATATTATAGGTAATTCTCTAATTTGGTGCAGCTAGGCAAATGGGGTTCAAAGACAAGAAAAGTTGTCCTCTTCTTTGTAAATTGGCTTCTGAATACATAAGGAAATCTGAAGGATGTGAAGATGATATATATGCTTTTTTCGAGAATGAACCAAATGTGGATTCACTTTTTGTGAAGCTTGTAGAGGAGTTTGAGAGATGCATTCTTAGTTACTTTGCATTCCATTGGAGCCATGGTGATGTATTGATAAGTCAGGTATTGGGGAGATTTACACGTTTACAGGAATACATATAGAAAATCTCAAAATCATCTACATACTACATTGAATTGTTTGTATAATACTAACTGAAAGCAAATTTTTTCCTTTTTTTTTTTGTTTGGTATTGCAGGTGTTAAGCTCAGAGAAGCCAAAAAAGAAGCTCAAGCACATAGTTATGGCAGCAACTAGGTGTGTACTAATTACTATTATTCTCTAAGAATATTAATTAAGCCATGTTTGAAACCAATGTGGAGATCATATGTTAATAAACATGATGTGAATATCTAATTTTTCTTTAGGCCTCAAATGGCTTAAGAAATCAATGAAATATAATAAGGAAAGAAACAATGATAGGACTTTCTTTCTTCTCCCATTCCTTTCTGAAGTAGCTAGCAAAAAATGAAAGAAAAAAAATTATGTTCATGAATAAATCACTTTTTGTAATTATGTGGTGCCAAAAATGCTTTGTTAGCATTTGAGATTAAAAGAGTGGGCATGTGATTCCTTTTAAGTTAAACAACATCCTTTGCATAGGGAACAAAGGTTTGAGAGAGTAACAAAGAATCTGAAGGTGGCTAGAGTTTTTAATACATTAGTTGAAGAGATGAAAGCAATGGGACTTGTATCAAATGATGATTCTACATGCACAGAGGTGATGGCTCCAATGGCTCTTAGTGATAGGAGCCCAATGCTTCTTTTCATGGGAGGTGGTATGGGAGCTGGAAAGAGCACTGTTCTTAAGGATATTTTGAAAGAGTAAGATAATTAAGTTGCATTTTTTTAACATAATACATCATTTTCCTTATTTATATTATCCTATGATGTGAAATACTTTAATCATCCAAACAAGCAACAAGAATCTAATTTTGTTTTCTTTTATCAATGAAATTTATAGACCCTTTTGGGCAGGAGCAGCAAGCAATGCAGTCATCATTGAGGCAGATGCCTTCAAAGAATCAGATGTCATATATAGAGCTCTTAGTTCAAGAGGGCATCAGGACATGATTCGAACAGCTGAATTGGTGACATGATTAACATTATTCTCTGTTTGTTACTCTATAAATATTCAATACTCAATACTCAATACAAATATGCAAAAGAGAAATGACTAATGTGCATTTGTTATAATGAAATACACTTGGATACATACTGATTTTTATAAAAATCACCACAAAAATAAAAAAAAAAATTGAAGTGTATTCAAAATGTATTTTGCTGTATCATATGTATGTTGATCATTTTTTATATATAGACATACTATTCTGTTTTTTTTTATCATTTTTGTGGGGTAAATACAATGAATCATGACAAAAATTTAATTAAACATTTATCAATAAGTTCATTATATGAGACTTTAATTCAGAAGATTAACAACTTAAAAACTTTAACAAAATAACAAAATTTATTTCAACAAGTTAAAAAGTACTTTGCAAAAAGACCATAAGTCCCTTTCTTAAAACCAAATAAGCTTTGAACAAGCCTTTTCAAGTGCCTCCTAATATTGCACTCATCTAACTGTTACATGATACTTTTTCTTCTTGCATGACAAAATCCTTGTTGCAAATAGGTACACCAATCATCAACAGATGCAGCCTCATCCCTGTTGGTAACAGCATTAAATGAGGGGAGGGATGTAATTATGGATGGCACATTCTCTTGGGTACCATTTGTTGTGCAGACCATAACAATGGCCAGAAATGTGCATCGCCGCCGTTACCGTATGGGAGTTGGCTATAAGGTGAATGAGGATGGAAGTGTAACAGAAAACTATTGGGAAAGAATTGAGGATGAAGAACCTGAAAAAGTTGGAGGTAAAAGGAGAAAACCATATAGGATAGAGCTAGTTGGAGTAATATGTGATGCTTACCTTGCAGTCATTAGAGGCATAAGGTATTTAATCATTGGTTTTTTATTTGAAACAATTAATGATATTCATCTATCATATATAATTTCTTTTCAGTGAAAATGGATTTTGCTAACAATCAAATTTGAATGGTAATAGGAGAGCTATCATGTGTAGAAGAGCAGTGAGAGTGAAGTCACAGTTGAGATCCCATAAAAGATTTGCTGATGCATTTATGACTTATTGTCACCTAGTAGACAATGCTAGACTATACAGTACAAACTCTTTAGAAGGCCCACCTAAGGTGCAAAATATTTCCCTCCTTTTTCTTCTTTCTTCTGACTTTGTTCACAAAAACTTACTTGAACACTTTATTTGAAATAGAGGAAATAAAATCATGTGTTTAGAATGTATAAATGATAGGATACATTTCAATCCATCCCTTTTTAATTTTTTTTGAGATTTATTGTTTTCTCTTCGAAATATATGAATATGGATGAAAAACATTGGTATAACATATTATGATGCTATCAATAGGTATTTACATTACTAATGATTACTATTATCAATGCTTTGGTGGGTATTTTTTGTCCCATGTATTCAATATCTATTATTGACTAGATAATTGTTGTAAAGTTGATAGGGTGGAAAGATAAGGACAAAACACTGCTTGTTGATCCAGAAGAAATTGATTGTTTGAAGAGGGTTGCTAGGTTGAATGAAGATGCCAATTCCATATATGAGCTTTACAAGCGTCCTAATCCAACTTGTGAA |
| >CrNIP5;1  ATGGAAGGGACCAGCCAAAATCTGTGCAGCTATGTCGCTGACACTATTGAGCTGCAAACTCCCACCACCCCCCAGCCATCATCATCCCCTCTTGCAAAATTTGCAGAGTGTTACCCTCCTGGGTTTTCTAGAAAGGTAACTAATTAATTAACAAATATATTCATTTCCCTGTTTTATCATATGGTTCAATTAGTACTCAACACCCGTTGGTAGAGTTAAATTTTATCATATGAAAGAAACATGTATAATTAGATTGTTTATATGTAAAGGAAATGCATAATTAGTAGATGATGATTGTTGACAGGTACTGGCAGAGGTCATAGGGACATACCTATTGGTGTTTGTGGGAAGTGGGTCTGCTGGTCTTGCAAATATTGATGCAAACAAAGTGTCAAAACTTGCAGCTTCACTTGCAGCGGGATGCATAGTAACGGTGATGATTTATTCGATTGGGCATATCTCTGGAGCGCACATGAATCCAGCAGTGTCTTTAGCTTTTGCCGCCGTGAGGCATTTTCCATGGCCACAGGTGCGTCCGTATTTTGTTATTATTATTCTAAACATATTCCTTTCATGTCCACGTGTATTGTTTTCCACGCCTTCCTCATTCAATCAATGGATATAAACATAACAGTACCACCACTCATGCTTCACGAGGCAGATTTTTCTTACTATCTTCGCCTATATTGTTACATACAATTACAATAATTAACAAAATAATGTTATTTGTATGGTTAGTTTAGTTTACTTTCAACAAATCATTTTTTTTTAAATTGGAATATATTTGTAGTATATAATTTTTTATAATATTATTTAATCATATATTATTATATTTTCAAATAGTTAACATCGTAAAAATGTTACATACAATGTATTGAAATTAAATTATTTATTATTTTTACATATGTTTTGATATTTTTAAAAATTATAATTATTCTCCGTAGGCAAGCATCTACCCATAGGAATAAATTTTTGAAAATCAGATTAATTAATTATTCACCTAATGAATGTATTTGTTAATAGTTTATTGATTATAATGAATTAACAACATTTAAATATATATATTGTTAGCATAATAAAAGATAAAATAATAATAAGTTAAATAATATTAACTTATGGATGAAATTTAAATTGAAAGCTTGTATAGATTAGTATTGTCTATTATTTGATAAAAGAAATTATACTAAATAAAAAGTGAAAAATAATTAGTTTTAACTATTTTTCTATCATCAGTTGTGAACCAATCAGACTATACACATGATGAGTTTAATTATTTATTGGGTCTTGAATTAGATAGTCTAATTCAATTTTTAATTTTATGCATTTTACACCTAAAATATGTAATTAATGCTGTTGGAGACAAATGTGTTATTAATGCATCCAATTTTAATCACTAATAATTTAAGGTTGATATCTCATGAGATATTCAGATGCAATGTGAGTGTCATATTAACTGGTCATATTCTCGGTTCTCCAAAAAAAGAAGACAAAAAAAAAAAGTTTATCTTGCCACAAGTGCTCCACCGCCCCTCACCACACATCTTTTTTATTTATTTATAAAAAATCCTTGAAATCTATAAGACATTAGTCATAAATTAGTATAACAAACACAAAATTGAGAGAGTAAGAAATTTATGTATTATTGATTTTAATTATAGTATCTTATAGTTATAAAATTTTAAATTAACAATAGATATATCTTTACTTTTTTTCATTTTCTAAATTTACTTTTCTTTTTAGAGAAACCAAATTTATATTTATTATGCTTGATAGTATTTTAAAAAAGGAGATGATAATTGGGAAAAAAAATCTCCACAAAATGATCATAAAGTCTTTTATAAGCTACTGAATCTATAAAATGGGGGAAAAAATATCTCCTATTAAAATGACCTTGATGAAAACTGAAACCAACATGAGTCAATTTACATTGGTTGAAGTCTGCGTCAAAGCGGTTCAGCCAAGGGAAAAACTGGTACTGGTCAAGTTCACGTCTCACGGAGGACCCACAGTAATGTGTATGCATTTCCATCCAGAAACTATCTGAAATGTTCATTAAAAACCGGCCCACAAATCCTGTATGAATCCCTACATGTGTGATGACCATTTGACCCACCCTTCTGAACTGAAGTAATGCATAATGAATGACCTTAATTACTTGCTGTTTACACAAATTCAAAAATATTTACTAAAATAAAATATGTATTATTTATTTTTAATGAATAATTTTTTGTCATTAAAAGCACAGCTACCAGTCTACCCTCCCTCGGTAAGTTTTGCATTATTGATGTTTGTATTTTGTGAGAAAGCAAAATAATTGGTTTAACTAAAGAAGGGGCCGACACAAGATGACAAGAATGAGAAAATGAAAGAGTCAGCGAAAGAATTCAGTTGGACTTGAACTTTAATTGTGAGAAGAGAAGGTGCCTTAACTGATAGGAGGGATAAAAATGAATAATAATCAAATTCATATTCCATTTTTTTGCAGGTTCCATTTTACATTGCAGCTCAACTCATAGGAGCCATTTCTGCGGCATATACACTGCGAGAGCTATTTCAGCCATCCAAGCAGATTGGGGAAACACAACCTGCTGGATCACACATTCAAGCACTAATCATGGAAATGGTGACCACATTCACCATGGTCTTCATCTCCATGGCCGTGGCCACCGACACAAAAGCGGTAAGAATAACCATAATTCAATTAAAAATTTAATTGAAGTAATTGAATTTCTTTGCCTATTAAATTTTTTCAATGTTGTCATTTGGCAGACGGGAACCCTATCAGGAGTAGCAGTAGGTTGTTCTGTTAGCATAGCAAGCATTGTTGCCGGGTAAGTAAATGAAAAAAGTGTTCCACACACCCCACACACACACACACAAACTCTTTTACATTACAGGAATTACATGACCATTGGCAAAAATAAAGTTAAAAACGGGAGAGTCAAAATTGAGGAAGGTTGAGTTGAAAGTAAGAGACATAAACAGAATCCGATGTTTGAAAATTATATTTATGCTGTCCGTTTATACAGACCAATGACAGGGGGATCAATGAACCCAGCAAGGACATTAGGTCCTGCAATTGCAATTTCATCCTACAAGGGACTTTGGGTCTATTTTGTTGGGCCAATCACTGGGGCACTTTTAGGGGCATGGTCTTATAATGTGATTAAGGAGACAGATCAGCCAGGTTTTTCATTTTCACTACTCTCCCTTTCCTTCAAGCTACGCCAAAACAATAGTGGAACTGAACAACTTGTCAAAAACAGCCACCGATGCTCGGTGTGA |
| >CrNIP6;1  ATGCCATATAGCATTATATTGTTGCAGGTAAGTGCATAAAAAACAAAAGTTCACTAGTTTATATATAAGTCAGGTTAGATCATTAACTTGTAAGAGATTGACAGTTTAATTAATGATATTGAGTTTGAATCTTAGATATGTATTTGCATTAAATGTTTAAAATAGATCTTACCTAATTCGAATAAAATTATTTTTCATGAAAGATACTGTGGTCTCATGCAAAAAAAGTTATACTTATCTTTTTAAAATTAAGGAAAATTCTCCAAGAAATGGTGTATATAACTGCACAGACACAGCATATCCGCACGTAGGAATACGAATCTGCAGGTACACTTTCATCTATTTTTGGGCATTCAAGTGCTATGATGGCTGATTCACTGTCAGTTAATGTTGACTCTTCACCTAAGCTTGAATTATCCACCGAACAAGCACATAAAACAAACCATGAGGCTGAACACTCTCCTTCTAAATTCCAAAAGGTGGCTCTAATGCATAATTGTGTATGTGAAAATAATCTAGATGTACCATGATTATCATAATAGCAATGTTCTTCAAGACCAATTCTCTTACGTGTCTAATGGCTATTCTCTAATTCTGCTTTTGCTTGTGTAGGCCATTGCCGAACTCGTGGGTACATACATTATTATATTTGCGGGTTGTGGAGCTGCCCTTGTCAACGAAAAGTTGCAACTTACAATAGTAGGTATAGCAATTGTTTCGGGTCTTGCTCTCACAGTTGCATTATATTCGGTTGGTCATATTTCTGGTGGTCATTTTAATCCTGCAGTCACAATTGCTTTGGCTGCGGTCAGAAAAGTTCAACTGAAACTTGTAAGCCACGTGTTCTACTAGCTCCCATTATAGCACTATGTAATCTTGCAATTTTCTATCACTCAAACACTGATTTATATATTTATTTATTTTCCAAGGTGCCTATTTATGTGTTGTGCCAGTTGATGGGTGCTACATTGGCCACTCTCACTCTCAAAGTGTTGTATCATGACAAGGTGGATATTGGAGTAACAGTGCTTACATACTTAAGCTCAACTTCTGATCTTGAAGCCATAGTGTGGGAATCTATAATCACTGCCATTTTGATGCTCACTATTTGTGGTGTAGCAACTGATCACAGAGGGGTACCGTTTGTTATACTTTTTCCTATGGATTATCTTAAGAGTAGCTGCTACGTTAAACCTTTTCTTTGCTCCACACTCCACAACCGCAATATTATATAGCAAATTCAGAAATTGTTCCCATTATCTTTAACTCATTCTATGCCTAACTTTTCTAAGACTAAAACTTTGTAGTTTATTTAAATCTGTTTGACAAAGGAGCTAATGTGGTATCCTAATTGATTGGAACAGAGAAAAGAACTCGCTGGAGTTGCAATAGGCATTGCCGTTTTGATTAACATCATCATTGCCGGGTAAACAAACATCCTCTATGGTCCTCTTAACACAATTCTAGTTAGTTTAATTTCCACATAACGACATGGGAATAAGATGCTTTGCTTTTGACTTTTTCTGTCTGTGCTTAGTTTAATTTTCTTTTAAAAAAAATGAGTTATTTTTAAACTTATGAAAATTATGCTAGTAGACAGTATATTAATGTGGAATTTCAAAGTGCTCATAACCGAACCAATCATATTATTGTGCAGGCCAATTACCGGAGCTTCCATGAATCCTGCAAGGAGTTTAGGCCCTGCTATAGTATCTGGTGATTATAGAAACATTTGGGTTTATATCATAGGCCCAATTTTGGGAGCAGTGTTCGCAAGTACACTTTACAAACTCCTAGATGTAACCAAACCAGCTAAAACTGTACCATTTCACTGGTGTAATCATAATCATTTACCTTTCTAA |
| >CrSIP1;1  ATGTTTGGGGCTATAAAAGCAGCAATTGGAGATGCAGTGTTGACTTTCTTGTGGGTGTTCTGTTCCTCCACGTTGGGGATAGCTGCAGGGGCCATAATCAGAGCCCTTGACGTTCAACACCTCTCTTACAACGGTTTCCCTTACCCTTCTTTTCTCGTCACTACTGCACTCGTCTTCATTCTAGTTTTCTTCTTTACCGTCATCGGCGAGGCCATTGGTGGCGCCAGCTTCAACCCCACCGGCACTGCTTCCTTTTACGCTGTTGGTCTCGGTTCCGACACTCTCTTCTCAATGGCTTTACGTTTCCCTGCCCAGGTTCGGTGCTATCATTCGTGTCCAAAAGGATCTTTTCCTTTTACTTTTTCCCTTTTCTGTGTATTGCTTTTGAAAATCATCTTTTTGCCCCCCCCCTTTTTCTTTAATGTATTCCTCCTTTGTTTTGGATAAAATTGATTTTGATGAAATTGATATGTGTTTAATGAATTTGAACTGTGTAGTACTGTTTTTTCATTGGAAAGTGGAAGAATTGATTTTTCAACACCAAAATAAACTAGTACTTTGTGCATAAATTAATTTCAATAGTATATTTTTATTGTAATATGTATTTTTTCAATAATTGATTTACCAACGTCAAGCCAAGTAAGCCAATATCTTGATATTTTTAAACTTTACTAGAACTGATTTTGAACCATTGAATAAGATAGAAATACGCGCATAAATTATTTTGAATTGTATATTTTCATTGGAATATGTATTTTTCATTGGAAAGTGCGAGAATTGGTTTTCTAACTTCAAACCGAAGAAGCCAATCTCACAAGTTTGAATCTTTTGAAGAAATTATTGGTGGGCTGATATGAATTTATGTAGAATGTTTGATGAAATGCTTGAGTTGTGAATAGGCGCTTGGTGCTGCGGGTGGTGCATTGGCGATTATGGAGGTGATTCCTACTGAATATAAGCACATGATTGGGGGTCCTTCTTTGAAAGTGGACTTGCATACTGGGGCTGTAGCTGAAGGGCTGTTGACTTTTATTATCACATTTGCTATGCTCTTCATCATTCTTAAGGGTCCTCGTAGTGAGTTACTGAAGACTTGGTTGATGGCCACTGCCACCGTCATTCTGGTCATGGCCGGATCTGCTTACACTGGGCCATCCATGAATCCTGCCAATGTGAGCGATTACATTTCTGCATTTATCATTTTTTTGTCTTTTGATGTATGAATTATGTTGCATTGCGTCAGTCTAAGTAATAATATCATGATGTCACTAGGGTGACTCACTGATTTGAATTATCATCTTTTCTTGTTATCTTCTTTGTATTTATTTTATACGTGAAACTTTGTAAAGCGCTTTTAATAACTTCTGCCTACGATGTCCTGTTCTTCTCAGCATTCTGGAATTTAACTTTTGCCCCTTGCCTTTGTTTATGAGAATGTCATGCATTGTTTTCATAATTTCATTCAACTATCGAAATGTCTTTTGTATGTAGGCTAAGGGTATCTTCTCCCTCCATCCTCTTCTGTCCCATAGTCATTCTGCTAGTTAATGGTATCAATTCCGGTTGTGACTTGTGTGCCATTTATGTTATTTACTCATTAAATGTGACTTCTTATCTTGCTCTCTCACATGACACGATTTTCATATTTCTCAACATTTCTTGATTAAGAAATCACCAGGGTGATTCACTGATTTGAACTATAATCTTTTTTTTGTCATGGTGTTCAGTATTTATTTTATATGCATTTCAAGTTTGAAACTGTGTAGTGTAAATCGCTTTTAACAACTTCTGCCTAGGATTTCCTGTTCTTCTCAGTGTTCTGGAATTTAATTCTTGCCCCTTCCTTGCCTTTGTTTATGTCTATCATATATATGCTATAGGTTCTTCTCCCCACCCCCGCGCTTCTGTCCCATCAATATTGTGCTATTTATTGTTGTCAGTTCCTGTTGTGACTTGTGTGGCAGACATTATTTACTCATTAAATGTGACTTCTTATCTTGCTCTTTCACGTGTCACGTTTTTCATATTTCTCAACATTTCTTGATTAAGACATCACTTGGGTGATTCACTGATTTGAGTTATCATCTTTTCTTGTTGCCTTCTTCAGTATTTATTTTATATGCCTTTCAAGTTTGAAACTTTGTATTGTAGAGTGCTTTTGACAACTTCTTCCCAGGGTTTCCTGTTTTTCTCAACGTTCTGGAATTTAACTCTTGCCCTTTGACTTTGTTTAAGAGAATATCTTGCATAATTTCATTCAATTCTTGAATATGCCATTCATATGTATGCTTCAGGTATCCTCTGTCCCCTTTCTGTCCTGTTGTTATTCTGCTATTTAATGGCGTCAATTCCTGTTGTGACTCATGTGCCACTGACATTATATTTACTTATTAAATGGGACTTCTTATCTTGCTCTCTCATGTGACACGTTTTTCATATTTCTCAACATACCTTGTTTAAGAAGTCACTAGGGTGATTCGCTGATTTGAATTATCATCTTTTCTTGTCACCTTCTTCAGTATTTATTTTATATGCCTTTTAAATTGGAAACTTTGTATTGTAGACCGTTTTTAACAACTTCTGCCTAGGGTTTCCTGTTCTTTGCAGCGTTCTGGAATTTAACTCGTGCCCCTTCACTTTGTTTATAACAATATCATGCATAATTTCATTTGAAACTTTGTATTGTAAAGCGCTTTTAACAACTTCTGCCTGGGGTTTCCTGTTCTTCTCAGCATTCTGGAATTTAACTCTTGTCCTTTGGCTTTGTTTACGAGAATATCATGCATAATTTCATTCAACTTCTGAATATGCCTTTTTTATGTATGTTACTGTTTTCTCCCCCCCCCCCCCCTCCCTTTCTTTTGTCCCATTGTTATTCTGCTAATTAATGGCATCAATTCCTGTTGTGACTCATGTACTACTGACCTTATTTACTCATTAAATGCGACTTCTTATCTTGCTCTTTCATGTGACATGATTTTCATATTTCTTAAGATATATGGATGATAACAAAGCTCAATAATAAATTTTATAGTTTTTAAATTGCCTTATTTCATCTTCATGGTAATTAATCACAAAAGTCAATCCATGGCAGCTTGATTTGTTTTCTCTTCTTTGTAATCCCCCCCCCCCCCTATATGCCAAGTGTATCTAACTGTGTTTGATAATGTATCAAATGAAAACTTGTGATATTGCTGAATATTATGAGCCAAAGAACATGATGGGAAAATATAGTGGTTAGTTTTACATTATGTTACTTTTACAAATAAAAATTGTGGGTTTGGTGTCATAGGGCATTCATGTTATACAATAAAAGTATATGAAACCATCATGGCCTCTTTGGGAAAAGTGCTTCAACACACTTTAATAGAAGATTGTCAAATTATGGCTTTGACCTTATGGAATATCTACTTTTGCCAGTTTTCCTCGATATCACTTTGACAATTCGACTAAACAATCAAAATTATATTGGCTATCCTATTAGTTAAAACATGAGAATTCGGGAGATAATTCAGATGTTCAGTTAAGGGCAGATTCAGTAGACAAATTGTAAGATTGAGCTATCCATAAATCTTATTGTATAGGACAGTTCGAATTATCTGCTTTTTGTGCATTCACGCATTCTTTCCTCTTTCCCTGGAATTGCTAATAAGGATGTCAAAAGGTTTCTTTCTATTAGGTGAGGTTGACTAAATGGATCAAATGGGATAATTATGTTTTATCAGAACTAATTCTTCACAGGCAATTTAGATCTAAATCCTTGTTAGTGATTTCACTCACCTCTAATTGTTGGGCTTTCCTCTATTTGATGAGTCTTCTAATTGGAGCTTCTATTGGTCTTCCCACATGTCCAAACCATCTTAGGTGGAGATGCAATTCTACCATCTTTTCTCTCAATAAGTATTACTCCTACTCTCCTTCTATTACATTCATTTTACTAATCTTATCCTTTTGTGTTTAACATTCATCCAATGCAATATTTTCAGCCCCACAACAGGTATATCTTATGCTTGTGTATGGCCATTTATCTCCAAACAGTTATGCTTAACATGCACTTGTCTATCACAAATAACATCTAAAATATTCCTCCATTTCATTTGCTAAGTTGAATAGTATGATTTACATCTCCTGTCTTTTCTCCAATGCTTTGTATGATAGATTTAAGATACCTAAACTGGGTGATATGTGGTATGGTATAACTTCCAATATTCACCTCCAAGGCAGTGCTAGCTTGCATTCCATTTACTCTGTTTTCCTTCTGCTCAAAAAGAAACTGTGCGTAGATCTTTTTATTTCCACGGACCCTCCCCATCGAACCTTGCAAAAGGTATATGACTTCCATGGCTGACCTGCCAATCATAAAATGATTTTGGTTCTTGAAATGTGTATCCCTTGTCTTAATCTTCCCAATCAATTACACTCTTCTGTAGTTGTGGTATGGTTCAATCTTAATCCTTCTATAGTTAGCACAATTCTGTATGTTGCTTTGTTGATCAAAGTGCTCCCCTGCTGTTCATCTGCAAGCGAAGCTATACTTTTCTCCCCTACACACTTCCATACTTCTATAGGTATATTATCTAGTCCAATCACTTTTTTGATTGTCTATCCTCTTTAGTGTTTATAATATTGGTCCTCTTCTCTAATGTTTAAAAAGATTGAGTCCATTAAATTTGTTTATCTTTGATTAATTAACTTGTGAAAATAACTCTTCCGTTGCTTCTTGATCCATGACCCTCTTCAGCCTTATGCAAAAAATTTAAACCATGTCTTGATTTGTGTGTCATCCTACCATACTTTATTTATTTCTTACATAATTTCTATATTTCGATGCAATAATGAGCAAGCACCTTTATTCCTGTGAAAATTCCTCCTCCATTATATTTTAGCTCTAACGTGACTTTTCCATCCACTTTAGTTTGGCTCCTAATGGCATTCTTTTAAAATAAAGAGGATTAGACCATGGGTTTGAAATCTGCTAGCCATTTCATACCAAAAAGAGATCCAAATAGATTTGCTGCTATCCTTTCTTTTGCTGCCTGTATGGTATTAATTATTGTTCTCATCTTCATTTTGATTATCTTTATTGGATGCTTCATGCTGAATTATCCAATCTTCTATGAGATGTGTTTATCGGTTGCTCAGCATCAACGTCTTCCTTCCAAGTTACTACATCACGAGCTTATATCATTTAAAATGAGTGATTATTATGATTGATCTGCACTTGAAACTGGATCACATGCCTCCTATTCTCAACAAAAGGACTAGATGTTGATCTATGTTAAATAAAATAGTCATCAAGTTATGCAAATATTCAGCCAAATTAGCTGCTTATGGTATGAGTTGACATAATGGAAGGACTCCTAAGTGAACTGGAATCTTGATTTTGTACCTAACCTTGCCAGACTTCAGCTACTCATACTTGGAATACAGAGAAATGCTAGTGACCTCTCTCTTTAACACTAATTTTGAAATACTCTATTATTAGGTAAAATTTCATGCTGCCCACTAACATTGACCTACACTCTATTGATGCGGGCAATATGAAATTTCACCCTATTGTGTGCTGAAAAGAATGTCACAGTATGTTGCTAGCATTTCTTTTGGAATGTAAACTAGGCTGTGGTTCATATGAATCTCCTTACATGACATGAAAAGTTTCTCTTGCCATCTTTAAACTTTTTTCTGATACAAATATAAATAATAGAGAAACAAATTGGTGGCTTGACATCAAATAGTCAAGTAAACAAATTTGGTGTCACTCATTAACTTCTTTGAAGTCACTATTGTTTATTTCTTCGAAATTCATGTAGTTTATACATACCTTCTTATGTGCATATGACTTGATCCTTCTGATGTTCATTTCAGCTTGATCTGGGTCGGGCTTATAGATGCCAATGAATAACTCTATGATCCCTTATTGTCTCTGCAGGCCTTTGGTTGGGCATACTTAAACAATTGGCACAACACATGGGACCAATTCTATGTATACTGGATTTGCCCCTTCACTGGAGCAATATTGGCTGCCTGGCTGTTCCGTGCTATCTTTCCCCCACCACCACCTCAAGTAAAACAGAAGAAAGCATGA |
| >CrSIP1;2  ATGGGGGTGATAAAGTCAGCTATTGGAGATGCAATTTTGACCTCAATATGGGTTTTCATCATCTCAACTTTGAGGATTGTCACAACTGAAGTAGCTATATTTCTTGGTCTTCAACCTTTCTCATTTGCAGGCCTAGTCATCACCACAATCTTTAACACCCTTTATGTCCTCACTATAAGCTTCATTGGTAGGATGTTAGGTGGTGCTAGTTTCAACCCTTCAACCACTATTTCATTCTACACTGCAGGGTTAAGGCCTGATTCATCTCTTGTATCCATGGCTATTAGATTCCCTGCTCAAGCAGCTGGTGGAGCTCTTGGTGCCAAGACTTTGCTGCAAGTGATGCCAACCCATTACAAGCACATGTTGAAAGGACCTTTCTTGAAGGTGGATTTGCATACAGGTGCTATAGCTGAGGGGTTGTTAACTTTTACTCATAATTTAGCTATCCTTTTTATCATGCTCAAGGGTCCTAAAAACCCTTTTCTGAAGGTCTATTTGCTCTCTGTGGCTACTGTGGCTTTGGTTATTCCGGGTTCTGGCTTCACTGGGCCTTCCATGAACCCAGCCAATGCCTTTGGATGGGCTTATACTAACAACAAACACAACACTTTGGAGCAATTTTATGTCTATTGGATATGTCCTTTCATAGGGGCTTCCTCTGCTGCTTTGATTTACCGGTTTCTTTTTATGTCTCCAACTAAGCAGAAGAAAGCTTGA |
| >CrSIP1;3  ATGCAGATCCCAGCCTTTGACACGTATCAACGTAAGAATATTCCAATCCCACTCTTGCTTTGCTGCTTTATTATTATTATTATTACTATTATTATTATTATTATTATTACTAAACACCTTTTTGGTCCTTATCACAGGCTTCATTGGTATGACCTTAGGTGGTGCTAGCTTCAATCCTTCAACCAATATTTCCTTTTACATTGCAAGGCTAAGGCCTAATTCATCACTTGCATCTATGGCTATTAGATTTCCTTCTCAAGCAGCTGGTGGAGCCATTGCTGCCAAGGTGCTCTTGCTAGTGATACCAACCCAATACAAGCACATGTTGAAAGGGCCTTTTTTGAAGGTAAATTTGCATACAGGAGCAATGGTAGAGGGGGTATTAACTTTTACTCACAACTTAAGCTACCCTTTTTTATCATGCTTAGGGGCCCTAAAAGCCCATTATTGAAGGTGTATTTGCTTTCAGTGGCAATAGTGGCTTTGGTCATTCTAGGTTCTGGGTTCACAGGGCCTGCCATGAATCTGGGCTTTGCTTTTGGATGGGCTTATATGAACAATAAGCACAACACTCGGGAGCATTTTTATGTCTATTGGATATGTCCTTTCGTAGGGTCTACCTTAGCTGCTTTTGTATATCGATTTCTGTTTATCTCACCAACCAATAAGAAGAAAGCTTGA |
| >CrSIP2;1  ATGGGACGAATCAAGCTTCTCCTCTTCGATTTCGTTCTATCTTTCATGTGGGTATCTTCTTCCGTTCTCATTCGCATATTTGTCTTCAAATTTCTCGGCTTCCGCCATGACCATCTCGGCGAGATTGTCAAGACAGCCTTCTCCGTCGCCAACATGTTCTTCTTTGCGTTCCTTGTCAGGCTTACACGTGGCGCCGCCCACAACCCCCTTACCGTTCTCGCTGGTGCTATCTCCGGGGACTTTAACAACTTCCTTTACTGTGTTGCTGCTAGAATCCCCTCTCAGGTTCTGCTTTTCTTCTGTTGTAAACTCCTTTTTTATAAGATAATTTTTTTAGCGAAGGTTTGAAGAAGAATTGTTACGAACCTTTTGGAGAAGTGCTTAATTAAATTTTTACCATAAGACTTCATATGATAGAAGTTTATTGAATATTGTGCTTAATTTAGTTGTTTATCCAAACACAACCTTGGTTGTTGCTAGTTGAGCTACAAGCTTTTTGGCACAATTGCTTGTTGTGCGTTCTAAATTCAGTTGTTTGACTGTTTCCTGAGAATAACTTTAAAGATTTGACCTATACTTTGATGACTCATGGTGTTTAGGGCGGAATATGATTATCTAATTGAATAAAATTCCTCGCATGAAAATAAAATAGGTTCTTTCCTCATCCTTTCTTGTTTTATTTACTTTTAGTTCATTATTATATGAGGAATCTATAGACTAACTATGAACATTTGTGCACACTCTCAGCATGTTTTTGCTTTTGGATGCTGCAGGAATGTTATATAGTTTGTGGCTAGACTACAATGCTAGGAATTTGTGTTTGTGAGTACTAGATTGAATTTCTAGCATCCATGAAAATCAAGGGTACCTGTGATTAATTGATTGAATGGAACATTTGATAAATATAGGCAGATCATTTTGGAGGTATCTGATCTGAGGCGTTGTCCATTCTGGTTTGGGGCATGTGATGATGGGTTCAAAATAGCACCTCATGAATTAACCTTTTATACTCTTTCTTCCGTCATCCCCAGAATTTTTCTGAGATATGTTTTTACTCGGATTACCAAGCTACCTGTTAGGCTATGTTGTGTTTCTGTCCCAACAAGAGTATCTGTTTGAATTGTTTCTTTTCCACAGAGTCCCCCCCCGAGAGAGAGAGACTTCCCTCGAGCAATATGCTTATATTATTTCCTGAAGTGGTGTTGGTTATGAAGTTTATAAAAATTACATTTTCATGACCAAAGATTGATGTCAATATGTATTATATAGTCTCTTGACTCTCTTCCTGATTTTTGTGAGCAAACATGGGCATAGCCTTAGCTTATTTTAATGTGTATTTTTTTTCAGAGAAAAAAAAAAACGGATCTGCTATATGCTTCAAGATTTTATTCCATAAAACCATCCCAAATATGTCTCTGCCAATATTATGCAAGATCTGTTGTATTTATAACTCAGTAGCTGGTTTTAGGGGTCAAAATCTAGACAACAATGCTTTACAAAATGAAATAATTTTTTATCGTACATTTGTCTTCAATGAACCCCTCTCCTTGGCCAGTCAGAATATTTTATTGAGTTCTTATCATACTTGAAGTTTTAGCTGTTCTGGGACTGGCAAAAAGTGATGAATACAAATACAAAGAAACACAGCAGTGCATATATATAAGATACTGCAGAACTGTGGTGTGTGTGGTAATGTCTGATGTGCGAGGAACCAGGGATATTATTGGAAATGACATTGCAAATATGTACAGACCATTACATCAAGTTTTAGTATAGCTGAACCATTTTTTATGCATCTCAACGATTCTAATGTCATTGAGATGAAAACTATCATTCTAAAATATGCATGATTTTAATGTCTCAAGGTGCTTGGATCTATTGTTGGAGTTAAACTTCTTATTTATACAATTCCTGAAGTAGGACGGGGACCAAGTCTGAATATTGACATTCATCGGGGTGCACTGACAGAAGGATTGCTAACATTCGCAATTGTAACCATTTCACTTGGACTTGCCACAAAAATTCGTGAAAATTTCTTCATGAAGACTTGGATCTCCAGCCTCTCCAAGCTAACACTTCATATACTTGGCTCTGATCTGACTGGTGGTTGTATGAACCCTGCATCTGTAAGTACTTTTGTATCTTTTTTTACCTGTCATTACTTTGATCACAGAACTTTGGTTATGTGAACCATAGAAGAAACCTTTGTTGTTTTCTTCCTACGAAACCTTTTTCTTTGTTCAGGTAATGGGATGGGCTTATGCTAGAGGGGATCACATAACAAAGGAACACATCCTTGTATACTGGCTTGCCCCCATAGAAGCAACTATTTTAGCAGTGTGGACGTTCAAATTGCTAGTCCAGCCTGTAAAAGAAGATAGAACAGCCTCAAAAAGAAAATCGGATTGA |
| >CrXIP1;1  ATGGATTTCGCTGATTCCCCAGTAGTTGACATTGATAAACAATTTCCGAGGCCTGTTCAAAATCATGAGGCTAATAAGAAATTTTTAGACTCCAAACTTCTTGACTCAATCGGTGCCCATGAAATTTTCACAAAAGAGGTAATGAAAACAAGTCCTTATAATTTCTATAGTAGGTGACAGATGCAACTAAAAGTTGTTCAATTTTCTTATCACTAACTTAGTGTAAGAAAGAATAACATTTGTTTTAAATTTTCTTCTTTCAGATGTGGAAAGCAGCTCTAACAGAGTTAACAGCAACTACATTTCTAGTGTTCACCTTAACAACTTCCATTATTGCATGCTTGGACTCAAATGAGGTTGATCCTAAGCTTCTTGTTCCCTTTGCAGTCTTCATCATTGCTTTCTTGTTCCTAATTGTGACAGTTCCTCTATCTGGGGGTCATATGAGTCCTGTTTTCACAATCATTGCTGCTTTAAAGGGTGTTGTGACTCTTGCACGTGCTCTTATTTACATATTAGCACAATGTATTGGCTCAATAATTGGTTTCTTAATACTTAACAGTGTAATGGACCCAAAATTAGCTGATACATATTCCTTGGGAGGCTGTGCCATTAGTGGCAAAGATGTGAATTCTGGTATAAAGCCTATGGATGCTTTGATATTGGAATTCACTTGCACATTTGTGGTACTCTTTGTGGGTGTCACATTGGCATTTGATAAGAAAAGGTCAAAGGATTTGGGCTTACCAATTGTATGTTTGGTGGTGGCAGGGGCCATGGCACTGGCAGTTTTTGTGTCCATAACTGTAACTGGGCGGGCTGGCTATGCAGGTGTTGGGTTGAACCCAGCAAGATGTTTAGGCCCAGCATTGTTTCGTGGAGGCCCATTGTGGGTAGGGCATTGGGTTTTTTGGGTTGGGCCTTTCTTGGCTTGTGTAGTCTATTATGCTCTGTCTATCAATCTACCAAAGGAGGGTGTGGTTTGGGTGGATGGACAATATGATGTGTTAAAGTTGGCTTTCGGTTCCAGTGGGACCATCCATAATAACAATGGTGTTACAAATGATCAAACAGAATGCCAAGCTCAAGTATGA |
| **Promoter (ATG upstream2000)** |
| >CrPIP1;1-Pro2000  CAACAAAAGAGTGATAAATGAAATAATATATTTTATTTCGAAAATTCTAATCGTGAAAAATCTTATTGAAGTCAAGAAGCCAACAAAGAAGTGAAATTCACCTCACTAAAATGTCTTTAATCTTCGGACAAGAGTAATTAGGTAAAGCCCTTTTCATTGTAGTAACCCTAAAATTCAAACCTTTGACATTGTAAGCAAAATTGGTCCAAAGTAATAATTCACTTTCATTGTTAACAATAACTATTGACTTAATAATTTTTTCATTGAATAAATAAATAACTATTTAAGAGAATGTTACAAAAAAATTTAGAATTTTTTTTCCGGCATCACTTTGCTTCATCATCATGTTCCTTAAAGTCGCTTTTTTCTTAAAGGAAACTTGCTTTGCGGTGCCCACTTTCTACTGTTTCACCAAACCAAAGCCGTTACAATAAGTTCAACGGTTACTTGTAGTAGTTTTAAGTGAAGAAAGTCATCCTCATAAGCGATTAATGCCTACCCAAAAACAAAAGCTTAATCTTATTTATATTGCTCTTGACTCTTCCTTTCAATTTTAGCCGTTACATAATAACATACTCAGTTGCATCTTGTGATATGAGAGGATGCAACTATGCTCCTCGTGATGTTTTGGCGCACATATTGTAATTCTCAAGGTAGTCCTTAAAACTATATAGATACAATATAGTTTTTAATTTTTAATTTTATTACAATAAATTTTTAAAATATTTTTTAATTTTTATTTAAAACATAATATTAAAATAAATGTTATTTTATAACACTAGAATTAATGAAATTTTTTTAAAAAAATATATATACTAAAATATATAAACTCAAAATTTTAATGACTAAATCAAGAATTATTCTTTTTATTTATAAGTAGATAATTTTTTTGTTGTATATCCAAAAATAAACAGAAAAAATTTACAAAATATTAAAAATATATATTAAAATAAAAGACAAATAATATTTACATTTTCCAAAATTCATTAAAAAACATACAATAAATCTTTTTTTTTTAAACTATTATAATTTTCACTCTTATCCTTACCGGTATTAAATATGTCAAAATTAAATATAAAATGATTTGATTACCATTATTCTCCTATGCATCTCCCGCTCCAAACCGTTATCTTTTTTATAACTTAGTTTACTAAAGAATCACAGTGAGATAACTCTAGGAAAAAAAAATTCAATAACAAAAATTCTTTTGAAAGAAGCACAAAATTTACTTTTCGAAAAGAGGAAAGGATTAAAAATAAAAAACAAGGAGGTATCCAAATCCACCTAATAGAGCAAACTCTCCAAACCCCCACCATCACTGAAGTTCCAAATCCTCATACCTAATCCATAGGCTTGAGTGTGGTCACAGCAGCTAGCAAAGCAACCACCAAGAGATACAAGGCTTAAATCTCCCACTTTCCCCATGAGAGACTCCAACTGCTCCTTAACCATCTTAAGTTTCCAACCAACACAGTTTTTAGCCAATAGAACCTTCATTCATATATATATATATAGCCCTTGTAATAAAGTTATGTTCTTTAGGATCATGTTGAGTGAGAAAATACAAGTAAAAATTTAGTGTACAGTAGGAAGGGTAAGTTTTGGAAAAAAGATTTCATAAAAATCATATTAAAAGTCACGTAACACAAAATTCTATTAGGTTAGATATCAATTTCCGTAAGGATATCTTATGTAAAAAAGATAAATAAATTAAAAGTTTGTAGATCTATAAAAAAAATACCATTATTTTTTTAATAAATTTTAGAGAATGTGAAATATTAATGTAATTTGTTCGAATAAATATACTACTAATATGGAAGTGAATTAGAAAAATGAAATAAATCATGTATTTTAGGCTGTTAAATAAACCAAGTGATGCTCTTAAAACTAAGAATAATAAAAAAGTGCACTTTTCCATGGTGACGGCAAGAGAATCAAATTTGTGAAGGCCCCATATTGTGGTTGGACGGTGGCAATGCACACTTTCATCATGCGCTTTT |
| >CrPIP1;2-Pro2000  AAACTAGGCCCATCTGGGTTAGTCTTTTTGTTTTGTAACATTGAATTGTTGTTGGAAGAGATATTCATAAAGCCCAGAGAGTCCGTCAATCGTCGTTATCGACAATAGGAGTTGGATTATTTTTGGTTGAAGAGGAACATAAGGTGGGCTTTAGTGGACATTGGTTTGGGCTTCAAATGGGTCAGGTCTATAAATTGAGATGCTTTTTAAGTAATCAATTTTTTTAATGTGTCCAAAGTTAGAAATAAGAAATCAATAAATAGTAAGATTTTTAGAAAAATATTAAATTTAATGCCATCAATATATTAGATTCTATTATTTTCAATAAAAACTTATTAAAATTTACTATTTTTAAATTTTTATTGTCTATCTTTGTACATGCATTAAACAGTTATTAATTATTTATTTTCATAATTATCCGGCGGACAGTCTTTAAAAACAAAAAAAAAAACCTGCAACCCCATAAATTTATTTGGAAAATATCACATTAATTTTTTTAATGAAATTAGTGATTATCATTAATTTTGGAATATTCGTAGAATTGTGTTTCCTTAACCAGAACCTGGCATCTTTAGCTATGATCACATAATTGTAATTCATCCATATGAAATGCAATAAATAAGGCCGTCTGGTGTGGCCGTGTGGGTCAGGTAAGTTTTCATTTCATGTATCTTATCATAATATCACCAGCTAGACCATTTGAGCTCAAATTCATAAAATTGCATGAAAAAGAGATTTTTTTACCTTTAACAGTTGCAAAATTTTATATATTAAACAATAATTATTTTTTATATTCAGAATTTTGATAATTATTTAATCTGTAGGAAGGGTGTTAGTTAAAAAAAAATCCAATTTCAAAATTTTGTAAGCACATACAAATAAGTAGTTTACGCAATTCGAAAAGGTAATTATGATTGGATGGATATTAGTACAATTAAAAAATACCTACATTCTCTATATATATATTGGTTGTTGTATCGCAGTAAAGTTTTTTTTTTTTTTTATAAATTTATAATACATCCATATCTATAAAATTTAGAATTATTTTTATTTTAAATAATGTAAAATTTTAATCCTTTTAAAACACAAATGTTTTCAATATTACTAATTCATACTAAAATTATTTTATTTAATAACTCTTTTAAACAGTTGGATTAATTATGCTTTATAAAAAAATTTGATTCATAAAATTATTATTAAAATATATAATACTCAGTATTTATATGATAAAAAAAATGAATGAATTATGTAATGATGATAGTTTATAAAGAAATAAATTAACCGAGGATCGTATCATAAATTTTTTACTAAAACAATTAATTTTTTCAATGCAAAATATAGTTCTACAGAACATTTTGCATGATCAAAACAATAACGACACATAAAATTATTTATTTTTAAAAAATCAAATATTTTCCATATAAATGCCTTAATGATTATTAGCTTGTTTTTACTATCATTGACAGCTACCTCAAACAACTTTTTCTAGTTGGGATCTAAAATTGTCTTTGCCAAAAGTGAGCGAAGACTAGTGTAAACTGTTTGGAGCTTCTGAACACTAAGGTAAACATTCAATATATAAACTCGTTCCAAATAATTATTTATGCAATTAAATGAATGTGGAGTGTAATCACAGGGATCAACTAAATAATTTCATCTTTAAAAAAAGGCTAAAAGAATATTCAAATAGTATGCAAACTTAAAAGCTAAGTTCAAATGTTATAGTTGTTATTGGGTACTTCGGGGGCTATTAGCTCCATTCTATCCCTAAAGGGAGCATTTTGTGATGCATAAAATAATTTGGTACAATAATATGTTGAGGGAATTTTGCGAACTTGGTTTGAAAGTGAAGAGTAAAAGGAATGGTAAAGAAGCAAAGTGATGCTGTCAAGAAAAGAAATGAACCAAGACAAGTGAACTTTCAATTTCATACTATGTGTCACAACAATGCACAATTTCACATTCTTTCAATTTACTCACAAGACAGTCACCACTCACCA |
| >CrPIP1;3-Pro2000  CAGCAACTAATATTTTGATGTATAATAAAAACAATAAAATTTAAATTGAACTCTTTAAATCTTTCATTATTATGAACAATAATAATATATTATCAGCGTATTTAGACATATTATAAAATGTTGCTTTTAAGTAATTTTATTCATATGTATAAGTAATTAAGCCGAAAATTAATGATTATTGTTATAAAAATTATAACTTATTTCATTGTAGAAAGCCTAAGGTTGATATTTACACTATGGTGGTAGGGTGGTTAAAAAATTATTCATTTTTGACAGGAAGGTATGTAGACTAGAAAGTTTTAATTTTTTATAACGTTTTTTAATATGATTTTTTAAACTTGGATCACAATTAATTAAAGACCAAATTTTTATACTACTTAATTATACAATAAGATATTATAAAATATATAAAAATACATAAATAATTTTCTATTATTAACATAATCTTGATCCACATGTAGACGCAGCTCCAACAGGGCATCCTTTGAGTATTAGCTGTCATGCACAACATAAAAAGCTTGCCCTGACTCTGCTTTCCATATAAATATGTATGATAATAAAGACACCGATGATTAGGCCCACTATAATAAAATTTGGTGGAATATTTGAGACACAGCTAAAACTAGAGCATACAACAGCCCCACAACATTTATTATTCATCACTCTTCATGTTCGTGAAATAATACAAATGTAAGATCAAAGACAAGAATCCCTAAAATCATTCAAAATCTTGAAAATAAATATATGCAATTGTAGATTATGAGTTATGAAGCAAACTACACTCCCCTCTAATACTATACTATAAATTGTTTTAAGTGTTTACTCTTCTGACATGTTTGAATTATGGTTTGAAAATTACAGTTAGATCCAATAAAAAGTATTTTAATTATTTTATCTGTACAGAAATCATAATGTGAGGCTCAAAATCATGTTACAGAATAATTGAACGATGATTTGTAGACATTATTTATAAATAAAACTCAATTTGTGTTGTCGAAACCAAGGTTTGGGGGTTCTCCAACTGCAAAAGGAAAGCTTGTTGACGTGACAATTAACCGAGGCGTGTTCAATCAAGAATAATGTAATGCTATTTTTTTATTACAGGGATGATCCTAATCCACAAAGGTTACTAAAAGTAAAGTTTAATTAATGGAAGAATGACGGGTAAAGGGCCCCAATTAGATATAGTGTAACCGCAACAGAAACGTGAACTGTTGCAACTACAAACTATAGTTTGATTATGAAGGACCACAGAACTGAAAACAAAACTTGTTGCCTATGTCCATGTGCTAGGTAAAAAGCTGTCACTTTCCCAACGGTCATTTATTCTAGTCCATTCGATTTGTTTCTTAGTCCTGTGGCTGTGATTTTCCTACCGTTAATCTGATTATGACCTTTTTGGAGTCAAGAAAGATGAAATTGAAGAGGATATATATACCATTCACATTTCACGCTTACTACTAACCGGGGACAACCACTTTTGGTACAATTTCAAATACTTAATAATTTAATGCCCCATGTCATGTAACATGTAATTAATTAATTGTTGATCATCAAATAAATATAACAATGCCCAGAGAATGCAAAAATAGTTTTTTAGTGACAGACTCATTCACATATATGTAAATTATTCGGTAGAGCATGTCGCTAATAACTACGTAATGAAAAGAATCATAAAAAGGACAGAAGATTAGAGAAGATGACATGCTACAGTGTGCGGCGGATAACAAGACAAAGGAACCACAGTTTAATTTCCACAAAAAATTAGTTCCCTTTTTAATCATACATACCATCATTGAACACCTTAACCCACATCTTATTAATTTTTTAATACTGAAAGTACTATAATAATCAAATACCAAAGCATAAGAGCATACAGTACAGTAGAAGTTTGCTAAAAACAAAAAAACAGCCACACTTGACTTAACTTTTTTATTTCCAGCAAAATATAATATAATTATTAGCAAAAGGACAGCAATAATAGAGTTGCTCGGTATTTT |
| >CrPIP1;4-Pro2000  TATCAATTTAACCATTAAATTGATAGATATTATTATGTAAATCATATTTACAAAATTTTAAAAATTTAATATTTTATTATATAATTTTATTTTAATAAATATTAAAAATATCATTTAAATAAAATATAACAACACTCAATTTTTGGTATAATTTCGTAAAATTGAAATTTGATTTTAGTGTAATAATTTATTGAGTGTACTCTGTAACGTAATGTGTTGCAACGTAATGGTAGAACTAGAAGCTGTCACTTTCCCAACGGTCACCAAACGGCCAATTGTCTGTGCCCATTCGATTTGTTTCTTGCCACTCCCACGCTATCTATCACTGCGACTCTCATTATACAACCGTTAAAAACTTGAAATCACCTTTGCATTTGCCAGCTCAGGAATTATTCAAAGCTTGTGGGGTTAACTCTTTGACCAAAGTTTTTCCTAAGTTCTATCCATAATTTGAACTCTTATGTTTTATTTAGATGAAATATAAAATAGGTTACGAATAAATTAAAAAAAAAAGATAAAAATTAGAATGAAAATAAAATAAAAAAATAAATTGTTTGAATAGAATAAAAAAATATTAATAGTATAACTTTTTATTTATATAAATTTTTAAAAAATAAATTATAATTTTAAAAAATTTAATTTTTTATTATATTTTTTATTGACATAGTATTTATTTTTTAAAATTTTAAAAAAATTATTTAATAATTTAAAATAAAAAAATTAACCTCTCTTCACTTTCTTTTTTTTAAAGAAGAAAATCATTTTCAAAATTTCAATTACTTTTTTTATATAAAATATATAAATACAAACAAATAGAGTTAAAGAAATAAAATCTATACAAATTTTAAAAATAGATTTTTTTCTCCTAAATTAAAAATTACATTCAATTTCTCCTATTATGAATATACCGTTGGATTTCAAGGTTAACAATTAAATTATTTGTTTGGTTAAAAAATATTTTTTTACAAATAATCATAAAATATCATTATTTTTTTTTATTTTTATTTTAAATATTTATATAAAAATAGTTAAAAATAATTGTTACTATTTTTATGCATCTTTCTTTTTAGAAAGATTTAACAAATGATATATGTTACTTAACAAAAAAATTTCTTGTAATACATGGTTAAAGATCTCTTCGATTAATGAAAAAGTTTATTTGAAGAAACAAATAAAAAAATGTATATATATATGATTTAATGTATATGTAAAAATAAAAAATAAAAATAATCTTTATATGTAACACATGCATAGTGAAGTCAATAAAAAGTATCTTTTTATTGACTTGTATTATATACTCCAACAGGTAACCATTAATGCTACGCATAGATACATCTTTGTCGAGGCATTGGAATCGGAGCCAAATCCTCTCCACCTAATGTACCTTATAAAAGATAAGAGATTGATTATACGTTAGATCTTATGTATACTTAATAGAGTTAAAAATAAAAAATAAAAAAATAGAAAGAATTTTTTCTTTAGAATAAAAATTATTTTCATCGGAGCAGTCGATAAAGTATTTACACAATTTATGAGATAATATGATTAAGCTACATAGGTTATTGAATCACTTTGACAAGTTATTAATATAATTTGACGCAATTAAACTTGGAGAAGCACTTTGTATTTGATAACACATTTTTAAGTTTTTGTTGTTGCCACTCTATACGTCTTTTATTCAGTGCGCGTGACTGAACTAACTTTATTCTGAAAAATGGCTCGAAACAGCTAATCACAAGACATATAAAAATATTCCTTTTTTCAAAAAATAATTTATAACACCCTGTTTGAGTGCGGCGGATAAGATAAGAACAAGGGAAAGGTACCAACCAAAACCTGTCAACATCGTCTGGCCGTTTCCAGAACAATCTTTCAGTAAGAATGCAATTATCTTTAGGCTAAGCATAATTTAATTATGATTAACACTTCTAACCTCCAATGTCCTAGAACAGGCGGTATCCAAAGCAAAAAAGTAAGAGATTCTTAAATGAAAAGAAAAA |
| >CrPIP1;5-Pro2000  AATTGCAAAAGATTCCAAAATTTTGGCTCACAACTCAAAACTCATTATGATCAAGAGCCGTTCGTTTTATTTATGTTATCCTGACAAGTCATTCAGACAAATCATGGCGTACTAAACTAATGCATATTAGGTAACGCAAATGATATAATAACACTCCCAGAGTATTCAAATGGTTTATTTTGCTTTTTGGCTAACGACTTTTGTATCTCTACGTATTACTAAAGAAAAGCTGCTATTATATATCCAACTAAAAAAATAAATAAAAGCTACAGTTATAGGCTAGGCTTGGCCCCTTAACAGTATTTAGACTTGATCATTGTCTTATCCATGAGATAGAAACAAAACATATAAAAAGGCTCATTATGCTTAGCTCAAGAAGAGAGGAAAATGAGTATATTCAATTAGCATTTAATAATGGACGTGAGATTAGTTAATGGCTGACAGCCATGTGCCGACCATGTTTTTATAAATGAAAAGAAATAAATGTTGGTAAATATTACAATAACGTTGTTTATATCTTTTTCTTTTGTTGAAAAAACCTTGTTTATCTCATCATTATTTATTTCTAATCCCTAGTCTCTAGACGCCTCACCGTGCAGTCAGAAGTCGACTTCATATTAACATTGGCAATTACAATATCATCAAGTCCTGAGCTAAATATTCTTGGATTTACAAATATGAACAAACAAAAATTTTCTCATACACATTCAATGATTCCTACCATATGTTACTTGTTTTGAAACACCGTGACAGATCTGTGCACCCTGTCATTTATTACTTATTAATTTTGCATATGTACTTTTTATTTGTTTAAATTCAACACGTTCATTTGTCTAAATAATAATAATAACACATTAACTTGAATTTATATCCAATTACCTTTGGTATGTGATACTTTATATTTGGCTTGTTAAAAATATTTAAAACTATTATCGTAGGTTTAGAATTGAGTATGAAATTAAAGTTTGGAAATTAGAATTCATCAAATTTCATTCTTAATTTTATATTTTGAAATTAGTTTTAAAATTTAAAACCATAATGCCATAACAATTCCGTGTAATTTAAAATAACCGACTTGAATTATTTCCTAATTCTTAATTAATATATTAAAAATATTATAAACAAACATTTTTTTTATTGTTATACTTTATTTAATTCTACATAATTATTACTATCATTATTATTGATAATTTTTTTCCCACTATTATGACAATCTCAGCCCCTTCCATTGTCGCTAGCAACCATCAGTATTCAGTGTTGATCCTTTTATATGCATCATTACCACATATCAATATTCTATCATCACATTTTCTAGATAATAAAATTAAAAATTCCAATTTTATATATCAACAATTCATATAATCTAAACGTATAAGTTATATTTAAAAAAAAACTATAATTAAAAGTTAGAACTGTAAACTGTAATTTCAATTACATCACTGAACATAAAGTTAGAAAATAAAATTGATTACAAATTCAACATAAAATGGCATGTGTTTGAATTGAGGGAAATTCTGAAAAAGCAAAAGAAAAAGAGAGAAAAAACTGTTGACGCCTGACAAGAGCATGTAAAATAGTAATAGCGAAGTAGGAGCAGGAGTATTTGCGTGTTGGTTTCCAATTCTAATTCTGGTTGCAGAATGGACGTGTAATATGGAGTGACACGTGTCGTTTTTGAGAGGCGAGTTGAACGTGATATTTGAACATCTTGAGAACGTGGTGCACCGCCCCAGCAGGAAAGGGAGGCAAGGATGATGTGGAATTTGGTCCCAGCAATACAAATATGCAGTGCATCGGTTAATAGTAGATTTTTATTTACCCTTCCCCTTCTTTCCCTTTTGGAAACTAGAGTAATAAAATCCACGTTCCAGTGATGCGCGGAACATGGTCGGTCCTGGAGTCTTGGTAGGAGTGGTTAGAATCACGCCAGGTAGGACACGGTCCTCCATTAATGATTGGTCTCTATCAAGAAAACAATAAAAAAAATTCAACATATTACG |
| >CrPIP2;1-Pro2000  TAAATGATTCTCGCAATGGATACCTATATTGCTACTACCAAATAATAAATAGTATTCAAGTTAATTTGTCCATCATAGATTTATGCAAGATATTTAAGTTCATGAACTGCATTTAGATTATGCGAAGAACCTTATTTACACTCTTTGAATGCTCTAGCTGATTTAGATTCAGCACGTGCACTACTCGCTTTACTCTGAAACTATTGACTTGTTAGCTCGCATTTTTATCTTGAACCTCGGTGCAGTTTAGATTTGTTAATTCTTTGGCACGACACTGTAATTTGATATTGTGATCTTCATCGTTGATTTAATATGTCTTCTTTACATTTTAGCTACACGAAGAAAGTAACATAATAGACTTTATTTTTTATTTTTTTTATCTTTTACATAGTATAAGCTTCAACTTCATCCTTCGCTAAAAATTGGCGGAAATTAAAAGGACAGCCAGATTATATATAATAGATTGATGCATATGCATATCAAACTCAAATTCTATAGTAAGGTTTCCATGGCATTTTAAATATCAAAGAGTACGTTGTTCGTCTCCCTGCAAGGCTATTTAACTCTAATTATTATTATTATTATTATTATTTCTGGTCGCAATTTAACTCTAATTCAAATAAATGAAATAGCCAAATGGACTTTCCTCATGAAACATCAGCTTCAAAATGACCATGATAGACTAAAAAAATAAATAATCTCTTGATGAGAATGATCGGTCATTACAATCGTTCGTACCTGTATGAGAAATATTCGTCCCCTTTGGTTTTGGGAAAGCTCCAGTTGCTATCTGATTTAGAGCAACGGAATTCGGGCGTACATATATGGAGAAGACAAAGGAGATTATGCTAATAAATGGCTCAGCAAAAATGTCAAAAAATAACGGTCGTTTTCATCGATTCAAAAGCTTAAAAAGTATTATTTAAATATGTTTGACTAATATTTTGTAATAAAATTGAAATCATATTACTTCCTCTATTTTTATTCATTTTTGAAAAAAATTATTCCTCTTTACCCGTTTAAGTAAAGTTTAAGATAATATTAATTAATATTTTTTACTTATAAGAATAAAATAAAAAAAGAAACAAAGAGAGATAGTGCAATTAAAAAAAATAAAGGATAGATTAAAATTTTATCTAATAATTATAATACAATGACAATTTTTTGGTTTATTTATTTTTTTATTTGAATAATAAATAGGGACAAATATAGTATTTGTGTTTACAAAGAAAAAACTTTTTTACAAGCAAAATTTTGAAGTTTATAATTAAAAAATACCAGTTTATTTTTTAATAATTTTTATTTGTATCCTCAATAATTATTTATTTTTAAATATGAAACTAACTACTATAAACAATTTCAATCATATTCACTTTTATAATTTTATTAAAAAAAATTATACAATTTAAACAAGATAAATTATTTAAAATCAAGTTTCATCCAAGATATCAAATATAAAATTGATTTTACACAATCGGGTTTGGGAATGATCCATTTTTCAATTAATATAAACACACACTTAATTATTGGTTAAATGAATATATTAAATTTAAAAATATTTGAATGTTTGATTTATATATACGGCCCCTAGCACGTGATGCCCTTTCCATTGTACGAATTCTCGGAGAAATCTTGTACTTTCTAATTAATAAAGTCTAATTTGGTGGAAAATAAAAAGTTGTGCACGTGATGCAATGCCTCGTATAGTCGTATTCCCGTGAAAAAATTTGTACAATACATACACATAGTGCCACTAGGCTTGAGGGGACCCCTGTCTCTCCTAACTTTACTAATCCAATTTCAAATGTGTAGTATCGAAAAAGTTTCCAACATTGCGAAAAACATGAGCTAAACGAATCCTTCGTTCTAGTACTCGTAGTTGAAAACGTAATTGGTGTTGGTATTCAACAATCCCAAGGGACCCAATCAGCTGGCGCCACGCTGTCAAAGACAATAAGCAGCATAAGCATATTCCCAGCTTCTCATGCCCTCGCTTCTCT |
| >CrPIP2;2-Pro2000  TAATTTTTATTTAAAATATTTTATTTATCAATATTTTAACCGTTATCCTTATAACAAAGATTAATAAATTATATGTTTTTTAAAAAAACAATTAACATATTCTTTAAATTACACTTTTTATTATTAGATAAAATACATGAGTTCCATATTTTAAAAATAAATTCCCTAACTTTAAAAGTAAATCTCACTCTAATTTAATGATATCCATATAGATTTCATTTAATAATAAATAATATATTACAAAATGTATTACCGACGTTCCTCTATATTTTATCTACTCCTTTAAAATGTCATACAACTTCGTGTCTAACAAACTGTTAGGTATGAATCTGTGAATAATATGTACATTTATTTAAAGTCTTAAAACTACTGCATCAATATTACATCCATTGGAGTAAATGTATTTTTTATAAATATTCGTTTATAGAATAAATTTATATTTGTAGGATAATATATAAATAAAATTATTTGTTTGATATACTGAAAATTAAATTAAAAACCTTCCTAGTTCCCCACCCCATTTAAACACGCTAAAGTTAAATATAGATGACAACACGATGGAAATTTGAACTATTAAAAAAAATAAAATAAAATACAGGACATAAAAACAAGGGTAGAGACTTTTTATTTTTCTTTTGTTTTCCATAAAAACTTGTCAAATACTACAAATAATTTGCATATTCTTTTGGTTTAAATATTTGTGTTTATATTTGTTGTGAAAGTGGGTCGAAGTCGACCTACGAATGGTGCAAAAAATAAATATTAGCCATTAACTTAAAATAGGATTTTCAAATCCGATTGAAATGTAGTTATGACGGATAACTGCTCAATTAATTATTAAATTTTATAATCAATTAAAATAAGTGATCTTAAACTTGTGGCGAGAATCACTTGTACCCTCACTGTGTTCAAAACCCTTTGATTTTAGGTGCGTCCAGCCATATATGTTTTTCTGGCTCGTCTTCGTCATCTAGCAAAATAACAACTACCTCTACACTTATTTTAATAAATTTCATAATAATTGCACTATTGAAAATAATTATTGATAACTTTCTTCTGTTTAATTTCAGGATAATATATCTTAATCTCTTGCTTATTTTTTTTATGCAAATATTTCTTAGACACTAACTTTGTGAAATAGTACCAGAGTATAGTCAACATTTTTGAAACAACAAACTTCAGGTAATTCAAATGCAACTTCATAATCTAGAGAATTCTAAATTTTCAAGGAGAAATTATAACAACATATTTTAAAGTATGATCAAATAATGAGCTAACTACCCCCCTCCCCCTCTCCCTCTCTCACAAAGACATTATAACATATATTTGATATTTACTGTTGTGTACATTACAAAATTCCCCTCTACTGTAAAAAGGTACTTTTCTATGTAATCTTATATTGAGAAATAAATATAAGTACTTATAAAATAATTTATTTAATAAATATAAAAATATTAGTCGGACTTAAAAAAAACATTTTAAATAAAAATATATCCATTCTTATAACAAAATTTGAAGAGTATCATTTTGATTTAAATGGCTACACATTATAAAATTAATAAAATATGTACGTGACAGAGAAGCTTAAATACATGAATTTACCACATTGAAACAAGAATTGATGGATGGAGAATTAACAGGAAGTACACATGAATATTGTTGTCTCTATTTATTTAGCTCCATGTGCTATGAAACTAAGGAAGATAGAGTTAAGTGCGTGTAAAGAGTAAAGAGGACCCATCAACAGAACATTGGGGACCAAAGCCATTAATTAGCCTTTGCCAAAGGAGTGACAGACAGGGCAGCAAAAACAGTAGACCCTGTTGTTGCTGTTTAATTTGCCATTCTAATAATTCTTAATTTCTGATGCTATCTACATGGCAATGCCTTTTAGCAATTTTGTGCACTTCCCCATAACCACATGTAGCCGTTGATACACAAGTGCCCTTGGTTGTGGGGCCATGCAAGCCAAATCAAGCTAATAGGGGCGGCAAAAACAT |
| >CrPIP2;3-Pro2000  GTTTAAAGTTAAAAATTATATAAATTATTTCAAATTCTCATTAACTCTAATAACCTACACACAAGAGTAATATGCATAAGGAAGAAGTGTTAATTTCTTACACATTTTGTTATAACAGTTTTTTTTTTTTTTTTTTTTTTGGAAATGGAGAAGGTAAAGGAAATGAGGTAGTACATTGAGTTATTTTTTTAAATAATTAATAAGTTTAATAAAAATATTATACAATTGACATTTGTTCCATATGTACAGAAACCATGATATGTTATTTAATCTACTAGATTAGATATTGTTTTAAAAATATGTTAAACAATATTAATGCGTTTTAGTCAAATAATTCATCTAAAGAAAAGGCCAAAAAAATTATTACGTTTTTTTTTATTAATCTAGACCACGGTTCCTAAAAATAGTTATGTTTTATGACTTGGTGTGTGTATTTCATATATTATTTTCTGTTACCTTTACCTCAAACTGGTTATTAAAATATTAATAATAATTTTGGTATGAATGTTTTTGTAATTTGTACCAAGATGTGTGTGATTTTTATATTTTTATGTTTGGATGGAATTTTTTTTAAAATTAAATAAAATAAATAATTATTAAAAGAAAAGTGAATATAAGAAAAATTATGAATAATAATTTAAGAAGAAAATAATACAATAAAAAATATTTTTAAAAATAATTATCATTTGAAATTACTTCTATTTCATATAAAAATTATTTAGAGAAATTTTTTTTTTCGTTATCATCTCAATACATAAATCTAGTAAAATTAAGTTATATTCTCTCTAAATGTAAGGTTATATAATAATATGCATTTTTTTTTTAAAAAAAGAAAGTATAGAAAAGTATTTCTATGTACTGAACTTGAAAGTTGATTAGAACTCTTTCCAGCTCTTCCCAGCCATCCAAATTCATTTTATTCTTTCCTTTTTCTTTTCTAAAATACATTATAACAACTTGTCAAATAAGACAGAAATGAAACTTGGAATATTCTTTGTTTTATGGTGTAATCTTCTTCTTTTTTTTGTGGGAGGATTGAAGTAGATGTAATATAATATTAGAACTGGAACCCAAAACCCATTTGAAAATAGTTATTGATTAAAACTTTGCATAATTAATTACAGACAGGGCCTCATAATTGTGAGAAGGATAAGGTGAATCGCCCATTTGTTCCGAACCTATTTGGTTTTAGGTGCGTCCAGATAAGTTCATCTGGCAAGCCTTCGTCACGCTACAAAAGTGACAACTACTTTTCTTCTTAAATTAATCCTATCAACAATTACATAATACTGAAAATGATTAATGATAGATAGCTTTCTTTTATTTCCCAGTATAAGGTACCTTAATCTCGTGCTATTTTTTTATTCTAATATTCCTTAGCCACTAACTTGCATGGAGTGGTAGAAAGTTCTAGCAGGGTCATATTTTTAGAAATGGCAAAGTTCAGGCAAATTAAAGATAACTACACTTTATCGTTAAAAAAAGAAGATAACTTCACTTTCTTGAGCAAAATTAAATTAAATAACTCCAGTTTTCTCTGTGCCATGAGATCAAGCGAGATTAAAGTTAGATTCATTTCTGGGATGAAGTTCAATAGATTTAACTTATCCAAAAATCTCTCCAAAATCGGTGGACAGAAAAGCTTCTGACCGTTGTATGGCTAGCTGCTTAACTATCTAATTCAATTATATTTTATGGATTTAAACGTCTTATTTTACTTATGTCTCCCTTTTATTAATTGTTCCTTTAGCAAAAAAATTGTACTTTTAAAATATTTGGCTTTAAAATAATTTATTATTATATATTGAAATGTAATTTTTAATCAAAGAAATGTAAAATACAGGTGATTGATTTTGTTATTTTACAAATTACAGAGTTGTGCACAGCCAGATATTTTAAAAAAAATTTATTTTAATATATCTTATATTTTGATATGTCTATTTATTATCATATATTATTTTTTTATTTGATATTTATAATAGTATAAAATATAATTGGTG |
| >CrPIP2;4-Pro2000  TATTTTAAAATGTTTATTACGTTTTTTATGTTTAGTAGAGTTTCTAACATCTTTTCTCTTTGTTTTCAACAGGTACATGGCTGATTATTACATTTCTTTTCAACTTTCAATACTTGTTTGATGTAATTTTAAGTTATTTCATTTGTAAATTAAAGTTTGATAATAAATAATGATGTATTTAGTTTAAATATATAGATTATGTGAATTCTGTATTGGCTGTCTGGTGCTTCTCTATTGTCTGCCTGGATTCTGTGTGTATATGCAGGTATGTGAATAAATAATACGGTAGTATACCTAAACAAAATGAACATTAAAAAAAAAAGGACTTTCTACCTCGGTTACGACCATAACCGATATAGAAAGTACACTTTTTACATCAGTTTCGGGCAGAACCGATGTAAAAAGTATGACTTTTTACATCGGTTCTGCCCGCAACCGATGTAAAAAGTGTACTTTCTACATCGGTTCTTTGCCCAACCGATGTAGAAAGTCATACTTTTTACGTCGGTTGCAGAACCGATGTAAAAAGTCTTATTTTCTATATCGCCCGCTTCAACACCGCCTGAATAACCGATGTAAAATGTGCTTAACTACCGATGTAAAAAGCTTATTTTCCACTAGTGATTGTAAGAAACACTTTGAGAAGGATAAAAGATAAAGAGATGAAGCAATGATATGATAAAGTCATGGAAGAGGAAGAAAAAAAAATGAATATGAAAGCACTTAAGATGATGACAAAATCTTGTCTAAGGTTGTTTGGCGGTCCTTCAAAGTCAGGTCCAATGGTTGCAGTCCATAGGTTTTGAAGAAAGGAAAAAATAAAAAAAGAATGAGTGTCCGTCGTTGAAGGATTGGAAGCAAACTTCACACTTTCTACCATGGAAATTGCATCGTTAACTCCACAAGAATTCTAAGAACATTCTTTCTTTTCTAACACATCTCAATACAATAAGGGTACCAAGATTTTTTAAAATTTAGATCAGTTATTTAACTCGTTAAATTACTGATGGAAGATTTAATGGTTTAATCATAATTAATTAGTAATTTAAAGATATTTATAATATATAAAATATTAAAATATATAATATAACATATTAATATTATAATTTTTATTATTAAAAATTACATATCATTTTAAATTTTTAAATTTAAGCTATATACATAAGATTAATTAAAATATAACAATTCTATTTTACAAATAATTAAAATAATATTATTTTATATAAAGAATAATTTTTTATATTTTTTACTTGTCTGTTTACTAATTTTACTGATTTATCATCAGTTTCTTTTTTTTAAAATTATTTTATGATTTAAATGATTTAAAGATATGATGAATTTTCAAATCAACTAATCCAATTAATTGTGTCCAATCCCAATACTTGAACATATACATACTATCCACATTCATATCCATCTTAAAATTTAAAATATTGATCGATTAATAAATATTTATAATGGATAATTTAAGTTATATAATTAATTATATAAAATTAATTATAATTTTTTTAATCAATTACCATTCTATTAATAAATATTAATTGATTAAAAAATTATAATTAATTATATTATAATTTATTTTATATAAAATTTTAATTATGTAATTAATTATATATTTTTATTAATCAATTAACATTTCAAATTTTAGGATATATATTGATTTGAATACTTTCCATATGCATTTTTGTTAAAACAATGATAAGAAGTATATGATATATAAAGAGAAGAGTATTTATAATAATATGGATTAAACCAAAGGGACGTAATCTGTTAAAAAAGTATATTTTTCCCTTGTTTTTAATACAGAATTTCATTAGTTTTTATAATCTGTGAAAGGGTGGTATGTCATAAAATGGCACATATTTTTCATTTCAATTGTTTTGTTCTAGACACAAACAGAAACAGTGATTTCACACATGTCTCTGAGTAAAACAAATCTTAAACTGAACGGATGAAGTGGAAAAGACAATGACCAAGTTGCTAAACTTAGAAGATGAAGA |
| >CrPIP2;5-Pro2000  TGGATCTAGTCATTTTTCCTTAAACGGGAAAATAAGTTTCTTAATCTACAACCACTTGTTTTCTTTAATCTAGGTCCTTAGCCTGAACATCTTGAGAATCAAAAAGGACAAGTTTAAACTCTTTTTAGTATGATTCCCTCAAGCAGAAAACGAATACCACAACTTATTAATTGATTTGGAAAGCATAAACAGAGAAGAAACTTAATCATTCTATAATTTTTCATAATTAATTCTAGAAACTTAATCATTCTATAATTTTTCATAATTAATTCTAGAGTTCCACAGGAAGAAGGTGCTTTTTACATTTTTTCTATTAAACATTGATGTATAAATCATTAGAAAATATATTTCCATAAAAGGAAAATAAGATTTTTTTTCAGCATGTCAACTTTTTATAATATTAATTGGATCGTTACAGATAATATTTAAGATGGGTTAAAATTCTTAAATAGTTCATTATACATATTACTTCATTTTCAGACACAGTAAAACAAATCAATAAAACTTACGCTGTTATTATTGCCAACAAATTTTGAATTTATATAGCATAATTTAATTAAAAATAAATTACTTCAATTTCTAATTAAATACAAACCTTATATGTATATATATTTGTTCACTTAGAAAAGGCGAGTCATTCTTATGATATAAGTCAAATCATAAAGCTTAGTATCCCCATATTTGGATAGTTAGTGTAACGTTATTTTTGTGCAAAACATAATTTGTAGCCACTGGAATTAGTTTGAATGATGAAAGATGCATGAAGTTCAAGTGGGTGGGGGGATCTAAATTCATTCTGTTTTACTAAATTTAAGATTTATTTGTTTTGGAATTTAATTTTTGCATCTTTTAATACAATATATAGTTCAAGAAAATTATGCGGCTTAAATTTATTTATATATAATTTTTAAATTAATTTTTTATTAAAATCAAGTCATTTTAACTTCAAATCAAATAGTCATTTCAAATGTATTTGCTTATAAATTGTAAAAAAAATCCTTCAAAAACTAATTCAGAAAACTATTTTTGAAAAAAAAAATGTTTTAAGCATTAAAAATAATAAATTGTTAAAAAACATTTATAATTTTATAGTTTCGTTCTTTTTTATCTGTTTGAAAAGAGAAAAAACACGTATGTTAATACATGCAATTAACATTATAAGCTTTTTTAAAAATGTTATTAACTTTTTTAATTTATTTTTTATACATACATTATATTTTTCATTTATTATAATTGATAAATACAAAAATATATATAATAAAAAATAATTAATACTTTCTTAAAAATTAAAGTGGATAAATAAATAAATAAAAACAAAATTTGTACTATCTTTCTTGACATATAAAAAAAGGATTTTTTATAGTAAAAATTACTATTTTTATTTTCTTAAATCAATATTGATGTTAATATTTTTCTTAAAAATATTCATCCATGATAATACTAGATACCCAAATGAGGAAAAATAGTAACTTCTTTCACATGATAAAATTTATTTTGGAAATAATAGTTACAAAAACAACTTAATTAACCTTCGAAATTGGGTTTAAAGTCGACAATTTATGTTCTACATCCGAAATTACTTTTGAATGTTATTTAATGATTCTAAACATCTAAAAGAAAAACACAATTATCCATTAAAAATGTTGATTCCCCTCTAATTAAGTTCCAGAAATGTCTAACAAACTAACCAGGACAAAATAAAACACAAAAGCCCTGGAATAAAAAGATTAAAGCGGTAGAATAATGTGCAAGTTGTAGAAGTTTGGAGAAAGTTGAAAACTAAACCCGTGATTGCAAGACAACACCCAACCACTCTACTTGTGTAGATTCTGAAGAAACAAAAAATGACAAAAAAATAAAGAGAAATAAAAATGCAAATGAAAAGGAAGGGAGGGGGTGGGGGAGGGGGTGGTGTGGCAAGGGTCGGTGAAAACTAAAACCAAACCAAACCCAATTTTTTTTTTATAATTTAGTTTTATGTTGCTTAGTGCCAAATTGCA |
| >CrPIP2;6-Pro2000  TGGATCTAGTCATTTTTCCTTAAACGGGAAAATAAGTTTCTTAATCTACAACCACTTGTTTTCTTTAATCTAGGTCCTTAGCCTGAACATCTTGAGAATCAAAAAGGACAAGTTTAAACTCTTTTTAGTATGATTCCCTCAAGCAGAAAACGAATACCACAACTTATTAATTGATTTGGAAAGCATAAACAGAGAAGAAACTTAATCATTCTATAATTTTTCATAATTAATTCTAGAAACTTAATCATTCTATAATTTTTCATAATTAATTCTAGAGTTCCACAGGAAGAAGGTGCTTTTTACATTTTTTCTATTAAACATTGATGTATAAATCATTAGAAAATATATTTCCATAAAAGGAAAATAAGATTTTTTTTCAGCATGTCAACTTTTTATAATATTAATTGGATCGTTACAGATAATATTTAAGATGGGTTAAAATTCTTAAATAGTTCATTATACATATTACTTCATTTTCAGACACAGTAAAACAAATCAATAAAACTTACGCTGTTATTATTGCCAACAAATTTTGAATTTATATAGCATAATTTAATTAAAAATAAATTACTTCAATTTCTAATTAAATACAAACCTTATATGTATATATATTTGTTCACTTAGAAAAGGCGAGTCATTCTTATGATATAAGTCAAATCATAAAGCTTAGTATCCCCATATTTGGATAGTTAGTGTAACGTTATTTTTGTGCAAAACATAATTTGTAGCCACTGGAATTAGTTTGAATGATGAAAGATGCATGAAGTTCAAGTGGGTGGGGGGATCTAAATTCATTCTGTTTTACTAAATTTAAGATTTATTTGTTTTGGAATTTAATTTTTGCATCTTTTAATACAATATATAGTTCAAGAAAATTATGCGGCTTAAATTTATTTATATATAATTTTTAAATTAATTTTTTATTAAAATCAAGTCATTTTAACTTCAAATCAAATAGTCATTTCAAATGTATTTGCTTATAAATTGTAAAAAAAATCCTTCAAAAACTAATTCAGAAAACTATTTTTGAAAAAAAAAATGTTTTAAGCATTAAAAATAATAAATTGTTAAAAAACATTTATAATTTTATAGTTTCGTTCTTTTTTATCTGTTTGAAAAGAGAAAAAACACGTATGTTAATACATGCAATTAACATTATAAGCTTTTTTAAAAATGTTATTAACTTTTTTAATTTATTTTTTATACATACATTATATTTTTCATTTATTATAATTGATAAATACAAAAATATATATAATAAAAAATAATTAATACTTTCTTAAAAATTAAAGTGGATAAATAAATAAATAAAAACAAAATTTGTACTATCTTTCTTGACATATAAAAAAAGGATTTTTTATAGTAAAAATTACTATTTTTATTTTCTTAAATCAATATTGATGTTAATATTTTTCTTAAAAATATTCATCCATGATAATACTAGATACCCAAATGAGGAAAAATAGTAACTTCTTTCACATGATAAAATTTATTTTGGAAATAATAGTTACAAAAACAACTTAATTAACCTTCGAAATTGGGTTTAAAGTCGACAATTTATGTTCTACATCCGAAATTACTTTTGAATGTTATTTAATGATTCTAAACATCTAAAAGAAAAACACAATTATCCATTAAAAATGTTGATTCCCCTCTAATTAAGTTCCAGAAATGTCTAACAAACTAACCAGGACAAAATAAAACACAAAAGCCCTGGAATAAAAAGATTAAAGCGGTAGAATAATGTGCAAGTTGTAGAAGTTTGGAGAAAGTTGAAAACTAAACCCGTGATTGCAAGACAACACCCAACCACTCTACTTGTGTAGATTCTGAAGAAACAAAAAATGACAAAAAAATAAAGAGAAATAAAAATGCAAATGAAAAGGAAGGGAGGGGGTGGGGGAGGGGGTGGTGTGGCAAGGGTCGGTGAAAACTAAAACCAAACCAAACCCAATTTTTTTTTTATAATTTAGTTTTATGTTGCTTAGTGCCAAATTGCA |
| >CrTIP1;1-Pro2000  CGGAAACTGAATCAAACAGAATAGAAATTGAATACCTCTGCGTAGTTTTGCGATTGAGCATGCGAATTCGAAGAGAGTATAAATTGAATCGAAAAATGTAAATCTAAAATAGAAATGGAATGAGTACCAAGTGCTGCGGCAGAGATCGCGACTTTACTTAGATTGACTAATTATGCAAAATCAGCAAAATATTGGGATTAGGGTTTTTTGCTCTAGGTAGAACACTGGTGACGCTGCTGGTCTTTAGCTTGGACTTGCACCAAATGAAAAAACCGAATGATACCAGCTCGGATTTTTCAGGGTCAGGGTTGACCCATGAATTAGCCCACCGGATCCAAATATTTTAGGCCTTCTGATCAGGTTTTTTTAGATCAAGGATGGGTGGGTTAAAAAAATAAATGGGTAGGGCTGGCTCAGGTTAAAGCTCAGTTTGAGAGTCTACTTAATTATTATGTAGGAAAGTGACTAGTCATTTAAAGTTGAAAAAATTAATAATTTGATATTTTAATTTTATCATATTTATCAATTATGATAAATTTTTTTACTAAAAGCACTAATTTTAGACCTAATTAAAAAATTATACAAATTATAATTAAAAACTAAGTATTAATTTTATCATTAAAACTTGTTCAATAACTAATTGTTACAAATAAAAGGTTACAATATACTTTTGATTTTTATTGTTGCACTCGTTTTAGTGTGCATTGTGTTTAATTTATTATAAGAATATAAGTGTTAATATTTATAAATATTTGAGTAATTTTTTAAATCAAACTATTATTTTTGTTTAAAAAATTATTTCAACTATATATTTATCTTGTATTTAGATGAGAAATTTTAGCATTGTAAGAAATTTTAAATGCTTTTAATTAACATATTTTTATTTTAAAAATACTATATATAAAGACATTTAAATCTTTTATATTTTAAATTTCTTACTTGGTTGGAGGATTTTAAATTTTAAACTTAATTCTTTAAATAAAATTTTATTTTTTTCTATATGAAACATTTTATTTTTGAATATATATTTAAAAATTAAAATAATACTAAAAAATAAAAAATGTTAAAATTATTTTATTAATATTATTAGTATTTATGTTGATTTAAGTATATTTTTTTAAGTATTTTAAAAGTTTCTATTTATTAATTATTAACATTAATCATTATAAAAAATTACATTGTATTTATCAATCTAGATATTGCATACACTTTTTACAACATCAATTTTTTTTTTAAACAATTACATTGTATTCGAAGTATATATATTTACACAATATTAATTGGATAAACTTTTTTACTATATAATAAATTAGTAGGATTATTTTTTAATTTCACTGTAACATAATTCTTCTTAAACATTTTTAATCATTCTAAACTTTTGAGTTAAAAAAAAAAGAAGGCATTTGACTGTGATTCAAATATTAACAAGAATTATATTTTGTAAATTTTTTCTTAACTACAATCCTATTTATTAAAAAAACGATTTATGTATACAATGTGTAGAAAGTGAAAAAAAATAGAGATGTATAAAAACTCTTAAACTGCATAATAAAGTCTCTTTAATTTTTTTATTATAAAAAATTTAGATAAATTAGATTTTGAAAGCTCGTCAAAATCAGATTCTGGCATTGAAATCAGAATTTGAATCCATGAATTTATGAATCACGAATCCAATTTAGTTCAATTGTAAGGCAGAACAATTAATTAATTATTTTTTAGGTTTGAAAAAAATTAGGAAAAATGTATAAGGCAGCACTTTTGATTCGCTGGTGCTGAAAATACAGCGCATCATCAAGTAAAACCATTTGATGTCCCACTTAGTTGTAGCTAAAAATCACATCACAGCCACATATATGGCAAAAATATGACCAAGTCACGCACTTACCTACAAGTGGCTCCACTAACACTTTACCCCTTAGTCCTTATAAGGTGCATGCTTGCTTGCATTATTGTACACTACACACTCTCTCTCACTGACCGTTTCCTTGGCCGTTAATTTT |
| >CrTIP1;2-Pro2000  ATATTTTTTTTATGTATTTATTGGTTCATAATTAAAATTAAAATAATAAAATTTTAAGATGAAATAAAATTAAAAATAATGAATGTAAAGTAACTTGTTGAATCCATTTAGGAATTTATTAAAAATGAAAAAAATAATATATATATATATATGTATGAAATTATATAATATATATATATATTATTTAAATAATTGTAAGCAATGATGCTTCATTGAAGTTCTTAGAAATCAAGTTAGAATTAAGAAAGGAAATGAAAATATATGGACAAAAATTAGGGGGAAAATTATCGAAATTTAATTCTTACATGCAATGTTTATTACCCCTTTTTATTATTACTCTTATTCAAATAAATTTTCTTTAAATATACTTCTTTTCTTTATTTATCAAAACTAATAATTTTAAAAAATATTTAAATTTTAAATAAATAAAAAGATAATAAACACATCAAAATGACAAAGAAAAGAAATTTGCAGGATCACAAGGAAATTATATTCAAATTCAATTGCATGTTGAGGGTATACAATATGGATAAAAATCTTTGATGTACAATACACTTCTTACAACGTGATGGCACTATTAAAGCTCTGATTTTTCGAACTTACATATGGCTTGAAATAATTATGTGAATTCCTTTCATTTATGCTGTTTTACTTGTGGCTTGAAATAATTCCCTGAGATATATTAATTTACATTGGTTTTTGGCTCTATTGGAGGTCAAGCTGTGTTTTATTCATAGCATTTCAATATCAAAACAAGAGACATGCAACTTGGTTTAAAGTATTCGTTCTTTAAGCCGTCCATAATAAAGAAATGAAGCAGATAAGCTTACAAATTAAGACAATTTTGAAGGTGTCAGAAGAAGGGAGGAAGAGATGAAGCACTTTGTAATTTCCTGTGGCTCTTGATCCACTATATTTGTTTGTAGACAAATATATATATGTAGCAGTTAGTATTATTATTTTGTATCTATATTAGAGGCTTAGGTTTAATCGATAAACCATAGCATTATTATTAATACTATAAACAAGTTGAATGGCAAGTTCGACGCATGAGTACCACATATCACAGATGCTTAGGTGTATTCAAATTGCACTCACCAAATCCCGTCTTCTCCTAAAGCTACCTACCTTTGTTTTGGGCATCTACTTTTTCAAAATTGGCTTAAAGTCTTAAATAATGATTTTAGCTTTTCTATGAAGAAACAAAGCTCAAGTTCAGTCCGACCCATAAGAAAACCTCTGAAACGCATTAACTTGGAACTAATGTAACATAATGATCAATGTTAATTGACACCCAACGAGTCAATGACCCTTTGCACACAAGGCAATAAACATTACAAATTTACGTTCAAAATATACATGTCACAACAGGCCATTCATTCATTTCGTAGGCCACATTTACCAGGGCTATTGATGCTGACTTCGTTAAAACAACTAGTCAACCATCATGGATCTCCTTGTTCGTGGGAATGTGGGATCATTAGACCTTCTCCACCAAGCACATTAATATTTTAAAATCACAGGAAAAATAAACATATATTTTAACCGTATTTATATTTAATATATTTTTATATCACACCATGTATTATATTATATTTTTTAATCTTTCACTTTTTTATCATTTCATACATCTCATTTAAGATGTATCAGAAATATTTGGTTTGGTATTTATTAAACTTATCTTGCAAAACAAACAATAATTAAGGCAACACTGAACAAATAAACGACGTTGGCAATGTCCAGTCCACGTCACCGCCATGTAAACAATCCGAAGTCGGTTGGAGGTAAAGCTAAGAAGGACCCACCATCACGAATTGGTCATCGGTCGGTGATCCAGCCTCATAACATCACATCCACCGTCCACGTGTCTTCACGGGAACAAAACGGAGGCCCCTAACCTGCTTTTTCGAAAATTCTCTTTTTCCCATTTTTCCCTCGTGTCTGTTTGTGCAATTGTGCTACAGCGTTAATGCAACAGCACTTTCTTGTTATTTATAGCC |
| >CrTIP1;3-Pro2000  CATTAATAGTATCAAAATTAAACTATTTAATTATGAAAAAAAACTTCAATTAAATTATTAAAAGTATTACTGCTTCTTAAGTAATACGGTAATTAGATGGTTACAAAGTAATTTTACTATGAAACAAGTTCGGTTTACATACTGTGCACATGGGTTTTTTATATATTAAAAAAACTGACATAGAAAATAGTTTAAGAATTTGATTTTTGAAAGAAAACTAAACATCCAAATTGAGCAAAAGCCATTTTTCACGGATACAAATCCTCTAAGTAATAGAGAGAAAAAATATATATAAACAAGGATATTTTTCGGAAAAAAATGAAATCAAGTTTTACCCAGTTTAACTTGAGAAAGTTCCGAACGTAATTCCTCAGCAAAATAAAATAAAAAGAAACAAAATCTTTTTTGATGGCCGTATATTTGGCAGAATCTAATTGACGTGACTGGTGAATAAGGAAAAGAGAAAATACGGGCATGACCAAAAAAAGTAATAATAGATTAAAATCAACATGGAATCAGGGGTGTGTTGTGGCGCTCTTAAGCCAGAGAGAACGAGACTATGTTGTTGAAAATCATGACCAAAAATATCAGATATAGCTACACGTTTGGGGACAAATAACTCTCCTCACGACATCATGCTCATCAGCGCAGAAACTTGGTGTCTCCTTCTTGTATTTGTCTCAGGAAAAGTTCGCAAGATCCATTACGTAATATTTGTATGGGTTTTCTGTTTATGAGAATTCTATTTCCACTGTTATTCTCATTATTGCAAGATTTAAAAAAAATGCAACCTGGGTTTAAAGTTTTGGTCCTATAGGCCGTCCATAAGACAGGAGTATTTGAAAAAAGTTGTGGCTCCTGATGCATCACATTCGTGGACGAGTAAGCAATTGAAGATTATATTAGAGGCTCAGATTAAATTGATAAGCTAGGGCAGTTTTATTATTGTCGTTATTACCATTATTTTAAGCAACTCAAATGACCAGTTAGGCACATGATTACAAGCAATCACCATGCTTAGGAATTCAGATCCAACCTCCTGAATCTCATCTTTCTCTCACATTATCTGCGGAATTTTCAATCGACGTCAAGCGGCCTCTATCTATGCACACAGCGATCAATTTGCCTTTTGGTAACAAAATAAACATGACAATTTTACAGCGCGGGCTCTTGGCATTTTTCCTACTTGGCAGCAATGTTTTAAAATATTAATGTTCAACAAGTCATCACATTCACGTCGACCGTACTCCACGAGTTGTAATGAAGCTGGCACGCCTCCCATCATTTCGTGGGCCAGGCCTTACCTTTTAAATCTGGACCTTAGAAAACAAATCTCCACTATCTCATATTTATTAAAATCATGACTCTCTCAGACTCTCACTCTCATTGTCTCCACCAAACACATACACTCTTCATTACAATTTTTTTTGTTAACGAAAAATTAGAGAAGGTAAATTAGTATTTGAATCATTTGGATTTTCTTCAAAACATGAAATTATCATTCTACTCAACTAAAGAGTATATGCGTCAAACTCTAAATAAACTTATTAAACTACATTTGATACACTGGTAAAAGGGTATAATAAAACCAATTGTTTTATTTTAAGAAAAGTATAAGAGGAAAGAAGTCATTGTGAAGTCGGTTACATGTATGAATGAAACTTGTTTGGAAAACAAATATATGCGGAATAGGGGCATGCGACAAAAACAAATAATAATGAATCCAACACAAACAACCCTATTGGGCCGGTCCACAGCACCGGCCAATGAAAATTCAAAGGAGTTGGTTGGGTCCAAAAGGTACGACATTGATGGAATCATGAAGAAGGGGTCGGTGATGATTAGTGATTAGTGAATAGCGAGCAGTCCACATCATATGGTACACGTGGCATCACAGAAACGCCCCGGAGGCTGGGCTCTGGACCACCCTAACAATAGACAGGTGGCAAAATGTTTGAATTTTCTCGGTTGCTTGTTTTGCCTTTGTCCCTCGTGCCTTA |
| >CrTIP1;4-Pro2000  AATAAAATTTAAAAGTTAATAAGCTGCTAATTAAATTAAAAATATTTAATAAAATTAACTAATAAATATAAAATGATAAATAAAAACATAGTTAAATAATAATTAAAACATAATAAATATAACATATTTTTTAACAAATAATATATTTAAATTTAATTAAATAAAATTTTATTTATTTTTAAATTTTAAATGAAAATTTTTAAAATTATTTATTTTTAAAATTTTAATTTTTTATCAAACATTTTAATATTATTAAAATAATATAATTAAAATAAATAACATATATTAAAATAGTAATTTAAAATAGGATTAAATATATTTTTTGTCCATTTAAAATTTAAAAGTATTTTGATTTTATTACATAATTTTAAAAATGTTTGATTTTGGTCCTCCATTTTTTAAAATAGATTAGTTTTAATTTAAAATAACTCATGTTTAATATGTGATATGTATTGATGTTTAACATGTGATATATGCTCAATAATGAGAAATTAAAATAAATTTACTTTGAAAAATGAAGAATCAAAATTAAATATTTTAAAAATAAAGTACTGAAATTGAAATTTTAAAATTTTAGAAAGATAAAAAAACATATTTAATTTCTTAAAATATAATATTTTATGATAATATAAAAATAAATTTAACAAAATAATATTATTATAAAAAAATTAATAATATTAATTTTATAAATATATAAATAAATAAAATACTAAAATATAAAAATACATTAAGCATAAAATAAACAACATATTTCAGTTAAAAATTAAAAAATATACAAATAATTTAAAATAATTTTTCTAAAAATACTAATAATTATATAAAAAATATTATTAACTATTAAAATAAATTATTTAATTTAATAAAATTTTAAATAATAATTTAAAATTTTAACTAGCTGAAATATTTTATCAAACATTTTTATAATAATAAATCATATAATAGTTTAATATATAATATATTTATAGTATATATTGATATTATGTATTAATAAAAAAATTTATTGGTATAATATAATTTATTATTAACATAAATTCATGGTATCTACATTAGCTAACAATTTTTTTAAAATAATATACTAATAAGAGTTAATCTAACATTAAAGTATTAATGAATGAAAAGAACATTTTATCTATATTTAATTTGTCCAATAATTTGAATACAAAGTAGCAATGAATTAGTTTAAGAGGGGATTTGATTTTTTTAAATGATTGGTTAAAAAATAAATAATTATTTCTCATAAGAAAAACTCTCCCAGACACGCAGAAACGAAAACTACGATGATTAATAAGAAAATAAAATAAAATAAAATAAAAAATCATTTTTGCATTGATGTATAAAATATTTCGATGATAAGGTATTTAAATGGAAGATAATACAATCATTAACAACCAGTGATTGTGACTAAGCTGGCAAATCATATGCATAATATATACTCTAAATTCGAATGCACATTTTTCTTTCCAATAGGGGAACTCATGATAAACTCAATTTAATGCTTTGAATGCTTACTATGTAAGTTATTATTTTTATTACGATTTGACTTTTCACTTTGATGCTTTGAACTTCTAGGTTTGGTTTGAAGGAAATGGATTTAAAGATGTGTTTAGTTACGTATTACAAAATTATTGTGGATATTGCAAGGAAAGAAAATGTGAACGAATTTTATTTTATACATTAATCCTCACATTCATTAGACTTAATTTTAACGGAAAAGTTAAATACACACTTAAAAAAATTACAATGATTAAATTGTTGAAGTTGTTGGAGGTACATGACAAACTTCGAAACAGAATATATAGGTGATTCCAGATGCACGGCGTACCTATTTGCTTATAATAACTCACTAAATAATTGAGGGGACCAGCTAAGCTAATCCAAAGATAGATCATGGAGGGAAATCAATTTTAATTAAGACCCTTTTGCATAAAGGTGAACTTTATGCCCAAACCCATATATATATATATATATATTTTTAAATAAAAGAAGAAGCTACATGCAAC |
| >CrTIP2;1-Pro2000  ATTTTAATTTTTTTTATAAGTTTAGTTTAGTTTATTTTACTTTTATATTTTTTTCTATAGGTGTTTATTAATAAGTTTTTTTGAATTAAATAAATTTTTAATATTTTATTATTAAATAAATAAAATAAACAATATAATGACTAAAATAACTAACTTAATATTAAAATATATAAAATGTAATTCTCATAAAATATAAAATAAATAAATTAATAAATCAAACTTATTAATATTATTAAAAATTAAAATAATATTAAATTTATGAAAATAAATAGATAATAAAATATCAAATTATAAAAATATATCAATGATAAAAGATTTATTTCTAATCAAATTTGAAAATTTTGAACGTTACTTAGAATAGTATTTTAAAAAATATATTAACTATTCAAAAATATTAATAATTACTAAAAAAATTTAATTAACAAAGTTAAACTTTTTCAACTAGCTTAAGTTAATTTTAATTTTCAGTTAGTTAAAATATGTTATCGCATACATTGAAAACGAATCAAAATTCTTTTTAAAAGTTAAAATCAACTAATGGCGTGTCACTTTTTATAAAATTTTCTTTCATCTAATTTATTTAAAAATTAAGGTAGATAAGTTAATTTTTATTTTTTAAAAAGTTAAATTTAATTTTTTATTCCTTTTCTTAAAAATATTTATTAAGAAAACTTTCTAAACTACATAGTTTGATAATATTTGCTTTTTTAAATAAGACAAAGTACATATTAGCACTTATGGTCGTCAATCTTCCACTATTTTTTAAAAAAACACATTAAGATACAGCTTGAATATGCTAAACTACTTCTAATTAAAGGTAAAATTGTTAATATACTTATTAAAAAAATATAAACAAGTCACAACTTGTCACGTAATATTAACAAAAAATATTAATTATAAATGATTTTTGATGTAAAATAATTGATTAACAATTATACTACAACTGGTATTCATTGATGCTACCAGAGAGAGGGTTGTGGGACAAGAGAAGCTACCGTATTGACTTTAATAAGCAGAAGGTTGCTCTCATAAAATTGTCTACAAAAGGAGAATGCAAAACCAAGAGACAAAGACAACATATAGAAGAGCTATTCCACTACACCGCCCAACCACTAGGGATCAGTTAAATTACAATAATCATGGTATTAAACAATTCCAAATTGCCAATGAAATAACAACATAATTTCTTAAGATATTGATTAAAAAATTAAAACAATACTTTTTTATCAAAACATATTATTAATGTATAATGTAATTTTTAATATTTTTTATTCTTTAATTAATATTTTTAGAGTACTAATCAACGAAGCTATTCAAATTAATTTAACAATAAAAAATTGAGAATTATAATAATTTTACATGAGAATTCAGAATTTTTTGTATTATACTAAAAATAATACTGATTAATGAAAAAAAAAAACTGACCTTATTAATAGTCAATGAATAAACGTGAGTAAGGGTTGGACAATCATTGGAATTTATGAATTATGAGGCAGGAATGTGAGAAGGAGACCTGTTCTTTTTCTCTCTTCTTCATAGTGGAAATAGAATGCCGGTAACTATATTTAATGCCACCCTTGTTATCATCATCATTAAATTTAAGCCTCTTTTTGTTCATTTAGCTACCTGGCAAATGGCGACTCACATAGATTTTTTTAATGTAAATGGAGTTGAGTATGATAATATTCGCTTAGGACATGCTTATCCACCCAGTGGTTTTTTAAAAGCTCCTCGTTTATTGTTCCATTGATTACTAGTTAACTTAATTGTCATTGAGCTCACAATGAATAAAAGAGATACCAATACAAATCATAGAATTTTTTAAAATAAAGAAAAGAGGGAAGCTAGGAGGTTTAGTTAGGCCCTCCCTTATTAATCAAGAATTATCCAACCACTCATGTGGGCCCATGGTCGGTGTCACTGACAGCCAACGATGGCCACCCTTTCACAATCACATGTATTATATTTATGTAAACTCTTGTTATATAAACAACCTAGAC |
| >CrTIP2;2-Pro2000  TTGTCTGCTACTAAGATCCATTATTGCATTGGTAATTCCAATCCTAAGCATCCAAAAGGAGATTTCTCAAGTCGAATTCTAATTTCTAAAAATATTCACGGATTTTATTCAAAAACAATACTTCTAATGAAACAATATTCTTGCTGGTATTTCCATGTCAAATAGCATGACATTTTAAACGTGAAAGTGATACTTTTATAACATATGGGACATCAAAATTGTTATTTTGAAATTGAAAGTAGATATCTCTATATCATTTTGATTTTGACGTGGCTCTAAAATCATATCTGGCAATAGAGAATAGCATACAGAGAAAGTGAATTTATCAGAAACTAGGATCGGAAATTTCGGCACAGGAGAAAAACGTTATAACCAATTTTATTATGATGTGGGGTTCATTTTTTTTTAACTTTATCTTTGCAAAATTTGGAAGTGAGGTAAACGCTGTACGTTTTACTGAATTGGGTTCTGATGTATGTACTATGTACAAGTGAGATAATTACGAAATTAAAGAATTGCTCTATGGCCTAAGTTATATTCATGTTTCTTATTATGTAGTCCAGTGGCTTTATTTTATATGACATTAGTTGGTAGATAAGGATTCCTATTCAATAGTAACTCGGAACCATCAATTTACACCAAACATGTGCAGCTGCAATGGCATCACAAAAGGACATCAGGCACAAAACTACTTTTCATCAAATTACAAATTACATAAACATTTATTGAGATTCATTAAAAAAATATTTGATACCTTTCTTTTCTTTATCCAACCAAAATTTTTGGACATAATATTTTATATTTCTCTTAATTATAACTTAATACAAATCTCTCCTATTTTTTTATTATATTAAAAAATTAAAGAAATATTAAAAAAAGTATTAAATATTTTTTCGATATATTTTAAGAAATATTCATTTATTTATCCAAACTACTTTGATTAGCATCCTAGTTCTGTGACTAATTCTCTTGGTTTAAACAGTCACTTTAATTATATTTTTTTTATAGATTTGTTGAAAGAATATTCTTTTAGCAATCTCTTTCTCCTTTCACCCATTGGGGACATCCCTTATGCACTATCACTTTAATAGTGGAACCTCTCTTACATCCTGGTGATATGTATGGTTATTTTTTTAAAAAACAAAAACTGCTGAACATGAAAAGAAATCTACAAGAAAGTAGATACTATAGGAATACCAAAAATTTACTTGGTTATGCCTTATCTAAATGAAACATTTTGGGAAATTTCAGTGTAAAAAGGACAATGGCAATTTAAACACTGTTGAAAACCAAAGGCCTGTGCATGGACCTTTCACTAACCAACTCACTTTCAATTTTTGGCCAATACCAAGGGACCGAAATTCACATATAGATATCCAAAGTATCTTTTTTTTTTTCTCTCATTGATAACATCAAATAATTAGTAATGGGTCTGTTGGTGCATATAAATTAATGATGATATAATCATAATTAACCAAACCCCTTTTTTTTTTTTCAAAAGAAACTTTTAACAATCAGAAGTGCCAAAAAGTCCAAAAGATTTCTTTGCCTAGCACCGATTAGCCGACCACTTTTTCCATAATATAGACTTAAACAACCAGTATGTGACACATTAATTACTTAAAATTATAAACACCTCGAATTTCTATTCATTCATTTTGAAATTCCACAACCTCTTTGCCATTTTTTAGAGCATGGCCGGTGCAATTTGTGGCCCAAAGCTATTCATGCTCTCCACTCCTACTTTTCTTTTATGATTATTTCCTTCACATTCTACCCCACCAAACACCACTCCCTCTCTCCTACTCACATGGAGGAATCATTCATTGGCTTCCGGACACTTCTAATAAAGCACACCAATGACAGCTAAGTTTGCCACTAAAAGACCTGATTGGATATGCATGGAAATGTTGCACATAACCATATTCCCTTTTATTATGCAAAACAAACATACATTAAAATAATCTTATAACTGCAATTAAATAAGTCTTAATACTACT |
| >CrTIP3;1-Pro2000  AGGTCTTGCTTTCCTTCATCATTCTTTGCCTTCTCACAAGGTCCCTCATGCTAACCTCAAATCAACCAATGTCCTAATCCATCAAGATAGCAAAGGTTACCATTCCAAGCTCACAAACTATGGCTTCTTGGCTCTACTCCCAGCTAGAAAGAATGCAGAAAAATTAGCCATAAGAAGGTCACCAGAATTTGTTAAAGGGAAGAAGCTGACACACAAAGCTGATGTGTATTGCTTTGGTATCATTATGCTAGAGATAATAACTGGCAAAATTCCTGGTCAAATCATAGGGGGAATTGAAGATACAGCCAATGATCTTTCAGATTGGGTAAGAACAGTGGTGAACAATGATTGGTCCACAGATATATTGGATTTAGAAATACTAGCAGAAAGGGAAGGACATGATGCAATGTTGAAGCTAACGGAGTTAGCTCTAGAGTGTACAGATATGACACCAGAGAAGCGGCCTAAAATGAGTGTAGTATTGATGAGAATAGAAGAGATAGAGCAAATGAGAAAAGATAATGACTGAAACAACTTAAGTCACTGCAGCAGGATGCATTATTGTGTTGCACGAAGAGTACCACAAGAATGTTTCTTGTATTCAATTGAACACTTGATTCATGTGACACATCATCCAAATCAGAAATTGCTTAGTAACTGCATATAACAATATGTACGGAGACAAAATTTTTTTTCCAGAAGAGATAAAAATCTTTGACATTGAGAAATTCTTTCCTTTGTTTTATTTTTCTTTATGTTTTCTTTTTACTTTAGAAAAGCACACTAGTGAGGATTAGAAGATGTGGTGTTGTACCAAACAGAAACAAAGTACCTCTTTATTTACAGTTTTGATTTGAGATTGAAAATATGCCTCATTCAAATCATCTGGAATCTCCTTTCCAGTTGTACCACTTCTATTATTCCTAACCTTTGCTCTACGTTCAGATTTTAGGATATGAGAAAATCACAGTTAAGTTGACCTATTTATTGAATGAAAGAATGTTTGTCCTCTGCATTATATAGAGTCATTTGAAAGGATATTATAGACACTCAGTTAAGCATTTGAGTTTTTTGTTATAGTTCACAAGTACTCTAAAGGAGACAATTTCATTTGAACCAAGAATATCTACAGCATAATAAGACTAGCTCTTTCTATATAAATTTTTATTTATAAAACTTGAACCGAAAACTTTGTTTAAAAAAATAGAACGTATACTACTTAAACAAACAGTTAGGCATTTAAATTTAAGTGAACGGGGTAATTTCTAATAGTTAATAATAAATGTAATATTAGCACATAAAATTTTATAATATGTCAGAATTAACCAACCCAAGAACTTGTCAGAAAATATTTATGTACTTGAACTGGGTCAAAGATTTTGGACCAGTCAAGCCTTTTCACATGGGGTAACCTCCACAAGCAAAGTTAGGGATGGACACGTCCACGTCATCCACAGGAATAATTGCAAGCTCTGCTTTTGACAGATGAGTAGGATGCAGCATAATAATAAATGAGGGAAAATATTGGACAGATAATTTTTGGAAAATTATAAAATCAATTAATAAGTAAAATTTATTAAATTCTATATGTAGTTAACACACACAAGTTGGTGATATAATCATAAACAGATCTAATGTTTAGATATAATTATACTAAAATAAAATATAATTATTTTGAATAATGTAACATTAATAAAAATAACTCAAACTTCAATACATGAATCAGATAAGCCTCTTAAACCGCAACCGCAAGGCATACACTGTGGTTTTTGTCCTTGTTGAGAAAATGAGGATCAACATTGACACTGCAACCAAACACAGGTTATTGTTGATGTAGATATGTGTAGGAAATAAAGAATGCTCAATTCGTAATAAGTAGCCAACACATACCACCGCGTGTCCACCATTTAGCATTTCAGAGACATAGGTGTCTCATCTAAGTTAAACACCTAGTTTCCCCATATAAGCACAATCACTCCACTTTTCAACCATGCAAAAGG |
| >CrTIP3;2-Pro2000  GGCTTATTAAAAAATTTTTTTAGATAAACAAGTCAAGCCTACGCTTATAAAAAATCTTATTTAACATATTTATGGGCCTATTTATATATTATATATTATATATTTATATTTATTTTAATATATTATATTTATATAATATTTATTTTATATAATATTAATATATATATATATTTTAGGTCTATTTAATGACCTTTTTTATCTTGTAAACTTTAAATTTGCAAGCTTTTTTAATTAATTATTTATATAGACTTTAAATTTATATATTAATTTTTTTTGTAACGTAAACTTTTAAACAGATTTTTGTAGCTTTAAATCAGACCAAACAGAAAATAAAAATAAAAATCTTTAACAGGTAACAGGCCACTCTGCCTAACTCGGCTCAGCCTACTTCCATCCCTAAATTGGACTAATCATTGAACCTTTGTTCTAGTTTGAAATTCAATAATCTAACTATAGTTAAATCGTAATTGATATTAACTAAATATTATTTTTTAAATATTATAATATATAATATATTAAAAAATATAAAATATATTAATATCATAATTTTATGTAATTAAAAATTAAAAATTAGTTTAAATTTTTAAAATTTAATCTATACATATAAAACTAAATTAAATAAAATATAATTTTATCTTTTCTTTAAAAAATAAAAAAATATTTTATATATAAATCGATTATGTTGATTTGCCGTTTTTTATTTTATTGTTTTGCTACCAATTTTTTACTAAAACAGTTTTATAGTTCAGATTAATATTTTTAAAATTGGATTAGTCAATATTGAATGGTTTAATTAAATTGTGATTAAATTGGTATTAATTAAATATTATTTTTAAAATATTATAATATATATTATATTAAATATATATAATAATAAATATATTAAAATTATAATTTTATATGATTAAAAATAAAAAATTACATATATTTTAATATTTTAAATTTAAATTATATAAATAAAATAAAATAATTTTTTTATTTAAAAAATTAAAATAATATTATTTTATATAAAACTGGTTTAATCGGTTGACTGCCGATTTATAAGTTTATTACTAATTTTTTATTAAAATAATTTTATGGTTTAATTAGTCTAAAGAGAAAATTAGCTCCTAAATTCAACAAATCTGACGGTCCGATTTTTAAATGACAAAAAAAAACCGGTTCAACCGGTCCAACATGTGGATTTGATCCGATTTTTATGTAACTTACTATGTATTAGATAGAAATATATATATATATATATATATATATATATATATATATATATATATATAATGCTGATACAGTATATAAAAGAACAGCCCAAATAATATAAGGATGTGGATCTTTGTGATATCATGTTATAACCTCTTGATCCAATAGATAGGAAATTATTTTAGTTGTCTCATTTTTCATCATGATCATTTTATAGTAAGTTAACCGTGTCATGTATGAGCAAAACAATTTGAATACCCCTTTTCAATATTTCTCCCAAATGGTTCCTTTCCATAAATTTGGCACATTACCTTTCTCACTTCATCGGGAAACCACCTTTTCTTTGGTCAAGCAGGAAATCACCCTTGAACAAAGAACATGTGGGTATTACAGGACCTTTCAGCTCCAATTTGAGTTTTCATTCTATGAAGTTGGACCATGCCAAAGATTTTAGGCTAAATAAGGTTGCCTTCACATGGCCAAACACCTCATGAGCATAGGTAAATATTTTTTGAAAGATTAAATAATATAGTTAGTATGAAAAGTTAATTTGGAATTGCACATGAATTATATTATATAGTTAAAAAATAATTGAATTACTGTTTCACCGATTTTAACGAGTGTAACATTAATAGAAAGTAACCGCAGCTAGAGTGCAAACTATTAAGTTGGAATTTGTTCTTATTGGGAAAAAAGGATAAACATAATAAATGGAACCAACCAGAAAGAGGTATAGTGCGGATATAGACGGATGTATAGAAGAAATAGAAGATCTGAATTTGAAAAAATTTGTGAACACATTTCAC |
| >CrTIP4;1-Pro2000  TAATTCAACCATTAAAATAATATATTATATTATATATATTATTTTTATAAATTTTTAAAATTAAATATTTAATTAAATAAATTTAAATTAACAAATATTATAAAAAAATATATTATCCGTTAATTTAAATTTATGTATTAAAATATCAAAATTTTTAAATTAAAAATAAAATACAATAATTTATATAATATAATATATTAATTTAATAATTAAATTAATAAAATTATTACATAACGTAACTAATTAAAATTATTTTAAAAATAATGTATTCTTTATATATATATATATATATATATATATATATATATATTAAGAGGATATATATACACACGTATGCGTAAATTAATCACATATATTAATAATTTATTGTTATTTTTATTTATAAACTAATTGATGAAAGTTTATTTTTAAAAAATAAAAATTCAATTTTTGTTAATAAATATATTTAGAGATATAATTTTAATATTTTTTTATTTGCTGATAGATAGAGTTTTAGTGTGCTGTGTGGATTGATAAGGCAATGAGTATATAGTATGTATACTAAAAGAATTTTACTAAAGTAATTAGTATATTTAGAGTGCAGATAATTGGTATAACATGAGAATTATATTATATTATATATATTTTTTAGTATTTATTTAACTGGTAAGATATAATAACTTTATCACATTATTTATCCTATTATATTATTATCCTTTCTTATTGTTCTAGTATAATTGTTACAGGAAAGCTGAATTAGAAAATAATAAAATAATGATATATTTATTTTTAATAATAATAATATAATTAATTATATCAATATTATATTAAAATAATAATATTAATAATAAAAACTAATTTTAATAAATTAGATATTAATATTAATAATAATTATAATGTTGATAATAATAATATTTAATTTTAATATATTTTTTCTCACAGTATATTATAAATTACCCTATCATAATATTTAACAAAAATATAAAATAAAATAAATTATATCGTATTTTATTCTATCATCATATTTTACCATGTTCATCAAACGAATCTTTATATGTATTCTTCGAAAAGGAGACGAATGTGTAGATTAACTTGAGATTCTTATCCTCTGTAGTTAGATATTGTTTTAGCTGATGTTATAGATGTTATTAAATTAAAATCTGTTTTTTTCTCTGTTATTTTTTATTTTAAATAAATTTAATTCTAACATAATATTAGTTATTTTATTTTATTAATTAATTAAAAATTATTTTATATATTTTTAAAATAATTATTAAAAAAATTATATCTTTCAACTATATATATGATTATCCATTTCTTTTGTCATAAAATCATAGATATTTTTAATAAGAAATAAAATTTTCAAATTGAATAGAATAATTATAAATAATAATTTTCTTCTATTTAACGCAGTTATATATTCAAACTCCAAACTTGAGGTGATTGACCAATTTAATAAATTTTCATTTCACTATTCGTGATAAGTATAGAATAACCCATAATTGGAATTAGAATATAAAATATATTTCAATTAAATTATATTAAAAAGATACTAATGAATTTTATTTGAAAAGTATATATGAATAAAGTAGTTGTGGATTTTTTAATAATTGAATTTATAACTTTCGTGTAGGATTATTTAACTTAACCAAGTAACATAGAAGTGAATAATAATAATAATAATAATAATACTAATAATAATTTATGCTGTTTGTCAATGGGGGAATGTAAGCGCCCAATGCGCATTCTAGCATGTTCCTTTGTCCTAATGCATTTATTCACGCAGCCAGCTTTTTATCATTTATAATAAATAATTAAAAACTATTAGTCAACGGGGGAATGTGAGGACCAAGTAAACCACCCGGTGCAGTTGCATTCTAGCATAACAGTACCAACTTGCTTTTGTTTTGCATATAGAATTAGCAGCCTGCATTGTCTCACTTATAATAATAATAACAATAAAAAATACATAAATGGTATTAGTCAACGAGGGAAAGTAAGCATCCAGTGCGCAAGGTAGC |
| >CrTIP5;1-Pro2000  TCTTTGGTGCTACTGCTGCTACAGTTTCATATGTTTTGTAGCCCCATTAGATTGGATAGAGGTGAAGAAGTTATTTCACTTAAATAATATTTTATAGTCTGTGTTTGATATACATTGATTTCATCTTTATTTTCTTCTTGGGCTGTTATGTTCCTGGTGCTCTCTATTCTCTTTCTACATGAGATGTCCGGTAGTAATGTGTGCAGATGTTGTCATGTGACAGATCTATTGATTTGATTGTTGCTCCATGAGTATTCTCCTCTGCTTGTAAATTTAAGATATTTCCATCTCTAACCTGAATGAGGGCTTTGCTTTACTGCTTCAGAAAGAACTTGGAGTTCTGCTGACCCCACCAAGTGTAAAAGAAAAGGCTGAGAATTGGAAAGGACTTGAATCAGAACGTAAATTGCAAATAAAATTTGAAGATGTGGAAAGGTATCATTCCAAGTTAATTCTCTTCTCTTATATTCGACCTAAAAAAGAATTTTATTTTGGCGTTTGAATTTTGAAGCCTTATTTAGCTTATTCTGTGCTTATTGTTGAGTCATCTCGGCCAAACATTGTAGCATTTGTGAATAGTAGTTAAATATGAAATTTCTTGTTTTTCTCTTTGGGCAGGCCATCATGTGATATAGCTATATCAGGTCTAGGATGGTTTTCCGTTGAGCCAGTTAGTAGGTCACTCAAAATCTCACAATCAAAACCTATAGAGACTGCTGGGGAATTGCTTTTGACTGTGCATGTCCCAAAACCTGTTGAGATTTTCGTGAGGCCACCAATACCAGTAGGCAAGGCTGGAGCAGAGTGGTACCAGTACAGAGAGTTAACAGAGAAAGAAGAGGAAACGAGACCAAAATGGTACTTTTGAATTCTGGTATGTGCTATGTATTTTTTTCTTTTTGGGGGGTTGATTCACTTCCATTGTCTGACGAATATTTGACCTTTACCCAAATCCCCGGCCAACCAATTTTCGGTGTATTATTAATGTGATTGATTCCCAAGTTGTACAACGATAACATTATTTCAAAAGATGAGGGAAAGTTAAAGATAGCAAAGAAATGATGGCATTTTCTCTCAACGCGGAATGTTGTGATTCTTGTTTTCTTCTGTAAAATTTCTCACTACAAAGAACGAACAGCGCACAGCTTCAGCGAAGGGATTAGATGGTTCATAACCTTTCTAATTCGAGTCTAATAAAAAAGCATTCTTTAATTAAAATTTAAATGAAAGGTTCATGCCTTACAATACGTGAACTTCCCTCGGAATCATGAGCAAATGATGATTCGCGTGTCCAACCTTTATTTTTAATTTGAAAAATAATATCTCATATAAAGTGGATGTTGCTATTTTTAGAGAAAGAAATATGTATTTCTATAACATTAAAAAAAACTTTCGTTGTGATTAGAGAAAATAGAAAATTAAATTTTTTTATCTCCGTAAATAGGTTTACCAGTCTCAATTCAAATCAATAGAAATTTAAAAACAACAAAAAAATTTCTTTCTTTAGGCCTTTTTTACCCGAATCTGCTTCTTTGTTTCTTGGTATAAAAGTGAAGTGATACCAAATAGTAATTTTTTTAAAAAAAAAATCTATTTATTGAAATCGGATTGTTTGGGTAAAGGTAAATTTTATAATGTTTTGTCACATGTACAAGAATATTCACAAAACTCTTGACAATAATCTGCCTTGGATTTATGCCATCATTTTTTTAAAAAATAAAAAATGCCCCATGTTTTATAATTAATTTTAGCTGATTTTAATTGACCGTTTAATAAAGAGATACTCCTTTGGAGTAACAATATGACAGTTTCCTGCACGAACAAAAAAATGATCCCAGTAAGCAGCCACATTGGTTGGCGGTTATAAGTTAGTTTTTATTTTGAAATGCATACGTGCCTCCTTTGGGAAACGGCGTGCCTTTATCTCCAACTGCTTATTTATAAACGGACTTGTTCATTTCTTTGTCTCCCTCTTTCTACCGTTCTTTGTCTCTACTACA |
| >CrNIP1;1-Pro2000  TTGGAGTCATACCAAGTGTGATAGAGTACATGGCTTCGCAAGTACGCAATAAAATATGTCCCATTAGACAACCGTTTCAGATAAGGTTGGTATTTGATAACCTTTTTAATTGTAATTTTTAATTATTATAATTCGATTTTAAATATTTCTAATTGGAAGTGGCATTCTAAATTTCTGTTTTTCTAAATTAAGGGATTGTACCAAAAATTTGATATTTATTCTTTTATTATTTTAAAAAATTTTGTGACGATTTAATCGCTATTTTGCTATTTTTAATAGTTCTATGAGCAGTTATCAAAAAAGTAATAGAAAAATTTATTCCTTTTAAAAAATAATATTTATAAAAAAATTATAAAAATTAATCAAGTAATTTTGTAAAAAAAAAAGTCTACCTCAAATTCTTTCTCTTTATATCTATAAAAATTTCATATATATTTTTTTATAATAAGTGTATGCTAAATTGTTGAACAAGTGGTTTTTGAGGATTTCCATTTTTAAAATTAAAAATTATAAAAACATTTATAAATATATTAAGTGAATTGGTCTAAATGACCTTTTTCTATTTTTCTGTTATTAATTTCTAAAATTTTTATTTATTATATTTATTGTTTGTCGTTTAAAAAATAATAAATAATATTAATTATGAAGTTTAAATTAAAAATGTATAGCAGAAAAATTTAAAGATACTACAAATAGGGATGGAAGTAAATAAGGTCATTCGATATGGGCCTACGATTCGAACTCGTTTAAAGCCTAATTAGTTCGGCTCGTTTAATAAATAAATAGACTCGTTAGAAAACTTTTTTAGATAAACAAACCAGGTCTAGACTTATAAAAAAAGTCTATTTAACCTATTCAAGTGTCTATTTATATTTATATATTATATATTTATTTTAATATATTATATTTATATAATATTTATTTTATATTTTATTTTACATAATATTATTATTATATATATATATTAGACCTATTTAATGGTCTTTTTATCTTGTAAGTTTTAAGTTTACAAATTTTTTTAATTAATTATTTATATAAATTGTAGACTTTAAATTAGGTCAGACCTTAATAAAAGAAAAATTTTTAATAAATAACAAGCTCGGATTAAGACCTCAAAATTTTTCTAAACTTCAACACTCAAAATCTAACTCGGCCCTACTTTCATCTCTTACTACAAAATATATTATATTATGTTACACATTAAAACAAGAGAAGTAATTGACACAAAATATATTAATGTAACAAAAAAAAAAGAAAAATACTTGACTGCAATAATTACCGAAGTAAATAATAGAGGCGGGTGAATTTACTTTGCAACTTTAAAAAGTGAGAGTTTTACTGTTGTACTGTGGTACAAAAGAGGTTAGGAGAAAACAATGAAGGATAAAAATATTATAATAAATAACTTTCAATGCTAATTAATAATTGTTACACGTCTAGGATAATGTTCTTAACCGATAAAATAGGAAAGTAAAAAGTTATCTTCTTTGACTTTTAGACAAGAAGACAGGAAATAAAAAGTTGTCTTATTTGATTTTAGTATGTATTAAAGGGGAAATATATATATATGTATATATGTTTAATTTTTAAAATCTGATAATAATATATTTTAATAATATTTTTACCTTATATTTTTATATTATATCTTCTTTTTCTTTTTTATCTCTATACTTTTTTCATCCTATTACATCTATTTATGATGTATATCAATAATTTGCATCTCCATTTCCCAGATTGGTTTTTCATTTGGCATAAATAAGAGACATTGTCCCAAAAAATAAGACTCAATTCCGTCCAAAATTAATGTGTGGAGTGGTATTCAGGCTCTTCTCTTAATTTCTGATGATGGGTAGCTGAACAAGCTTTCAATGAATGCTATTCTCAGATACAAATCACACAAAGGCTACAAAGATCAGTTTCCATGGATATATCGTATGCAAGAAAGGAATTCCTTTATCTTAAAACTACATACCAAGTGGAGCATAAATGAGTTTAATTGG |
| >CrNIP1;2-Pro2000  CAGTGTCTTCTCCTACATTAATTATTATTTTTTACAATTAATTTTTTAAAATTGTTAATTTGAATTAATTTTTGAATACTAATACATTAATAAATTTAAAGTTTTGTTTCTTTTGTTTAGATAAATCAAAATATTATTGTAATTTTATTCAATTTTAATTTAAATATAAATTTTAATTTTTAATATATCATATCAATAAAGATTATATGTCAAGATTTTAATAATTTTAATTTTATCCCTAGTGTTAAATTTTTAATTTTTTAATTTATTACTAATATAATATTAGCAACTGAAAACTATTATTATTTCAAATTTTTATTTATAAATTTATTTAAAATTAAAAATATCCATACAATATATGTATAATAATATGCTAATTTATTTAAGACGAAATTATTTTTAACTTCTTGGAAACTTTTAAATTTTTTTTTAAAAATAATTTCTAACAAAATAGGCACTATAAAATTGTAGGCCTTTGCAATATCATACACCAGCGTTCACACGTATCATTTATAATATTTTGAATACAATAACTTGTATAATTTCATGATATTAAAATTACGTACAGGTATGTTACTTTTACTTTTCAAATAATTTTATATCTAATTTTGTTATTAATCTTATAGTACTCTTTTTCTATATATCTACAATATGGTTTTGCGGTGTTTTTCAAATATTGATAGAGATATAAACTTATTGGTTTCGTGAATCTCAGTAATTATGTTCGATTTTAATTCTTACAAAATAATAAATTTGATAAGAAAATAAAATAGTAAGAAAGTAAATTATTAAGAGATGTGTTGTTCGTATTTTAAATGCATATAATAATTTTTATAATATATATTATCATATTTTTGAATTATATTTTAATTTTTTTCGCATTAATTTTTTTAAGAAAAAATAACATTTCTTAAACATTGATGGTAAGAAGGTTTATCTTTGTTTAAATTGTTTTTATGTTCTTTTATCAATTCAATTAAGCCGCTCATTCCGTTATCATTGGCTTATCTGACTTTTGTTGACGTAGCACTTTGTCACGATGGCTGAAATTTACGAGTGACGAGTCCAACGTAACGATTAAAAAATGTTACATGTTTTTATCAGAATAAAAAATCTAAAAAAGACTTGTTTTGTTTTTTAAATGGTATGTGTATAGCAATCATGAAAAACAAAAGAAAGAAATTAAATTAAACATTTAGAGAATGTTTAAAAATTATTTATAAAATTATTTTAAAATGAAAATAAAGAAGAATAAATAAAAAAGTAAAATAATATGTAGAAGAATAGTAACAATAAAAAACTAAAAATTGAAAATTAAAAAATAAAAAATAATTTAATTTAATTTTAAAAATAAAATTATTATTTTTAAAAACTTTACTCATCAAATATATATTTTTAATTTTTTAATTTTCGAAAACAAAAACAACACACAAACCATATTCGAAGGCTTTATGCATGCTCTTTATAAAATTAGAGTTGTATTCGCGTGATCTTATCCAACTTTTACGACAAACGAAAGTTATTCAAAGTTTAGATTGTTTTGTTTTCAATTTTGGTTGGGCGACTCCTCCATTTACCAAGTAAAATAGGAGACATTGACCCAAAAACAAGCCTAGGTCTTGTCCAAAATTGAGGTCATGTGCCATTCATGCTCCCTTCTGCATTTCCGGTGTTGGACAACCTTTCAATGCTTTCTTCATATCACAACACATAACCCACAAATATCACAGCTTTCGTCATATATCCTATGTAAGGTAAAAAGAAAAAACTTTATTTAAGAATTTTGATACAGAGCGTTGAGAAATAAGAATTTAAAAGGAGTGTGTACAAGTAGGAAAATAAAAAAGAAAAGTAAAAAGGGGTGTGTATCCAAAAACATGTTGACACATTAAAATTAAAGCGGTTGATAGATCCAAATCCCATCCGTGGAAAAATTGATAAAAGTCCAATCTCCGAATCTCTCTCTCTCTCTCTGCATGCTCCCTGAACTTTTGGGTGCC |
| >CrNIP1;3-Pro2000  TCCAACTTTGTTAGTCCAATTTTGACTTGAGATCCAACTTTATACTGATTGCATGATTACCGCAGCGGAAAACAAGTCGTCAAATAGGATAGAATATGCGATACTGAACAAAGGGACGGAATTTAAAAATTATGTAGCATTTTTGAGAAATTCTCCAGCGAATTACGCCTATCATATAAAATGAATTATCTAATTAAAATTTGATAATCTAATTTTTTAATTAATTGAAAAATAATAAAGAAACAAATCTTGAATCACATTTTTTAAAAATTAATATTTCCAATGAATTAAAGAATAAATTATTAAAATTTAATTAAATAATTCATAATCCTATAAATCCAATCCACTCACTAAAAAAGGTTTTCGCATTCTTATTCTTGTCTTTTACGGTAAGAAAGGGTCTAAATGTTTCTTTACATCTGCTTAAGTTTCTAAATTTGGGTTCACTCACACCCGTACATTCTTTTCATGGCATAGAAACAGTTTGAACTACTGATAATATTTATTTCTTTTTTTTTTGTAAAATTTTAAAAAGAAACAGTTTTTTGTATCTATTTGATCTTCACAAGGTTGCCCTTGTGTTCTTAGGCAGACTGATCATACATTTTGAATTCAATTTTAATATGTTATGAATATAAATTGTTAATCAATTAAACATACGATAAATATAGTTTTTGAAGCAATTATATAAAAAATTAATAATTCATATTAATATGATAATTTATAATTAGATAATAGTATAAAATATTTTTATATTGTCTGTATATTTAAATTAAATTTATATATTTTTAATATCTTTATTGATAGACAAAATTTCATACTAGTTAAAATAACTTAAATTTATTTTATTTAGTAGAGTTAGTACAGAATGTTATTTAATGATTCAAAATTAAAAAAATATCAAAAGACAAGTAAGATAGACTTTCTGTTATATGAAAGTTGTAACGGCCTAGATTGAAAAGAAAAGAAAAGGATTTAGGAGTTGTTTGTCTTAAATTTAACAAATTAATTTAAATGAAATTTGTTTTAAATCATTAAACATAATTAATTATGATATCTATTCAAATTAATTTTAACCATAAAATTTTATTAAAATAATTATGTTTGTTAAAAAATATAAAAATAAGCCTTTTAAATATAAATAAAAAAATATAATTCATTATGATTAGATTAATAATAAAAGGTCAAAATTAAAATAGTTCACATAAAATTCTATATTGATTTCATTAATTTTTTGTTATAGAAAAAAGAAGAAGCACTTAAGTCTCCTCAAGATGGCTGTGTCCAGTGCTGCATTCCTCTAGCCCCCACACAAAGAAAGAAAAAGGGTTATATCTGAAGACCTATTCATGATGCTAACTGTATATCCGGTGGGTAGGTGGTTCAGCCTTCAATGCATTTTCAGGGGCAAATTAACCACTTCAAATGCTCCATAAGCCATTTTGAATAATGATTAATATCAACACACGGAACACCAAAATCATAATTGATAACTCACCTTACTTTGTAATTTATACTGTCAACCACTCAAGGAGTTTATAAAGGATTAGGATGCTTTTATAATAATAGTAAATATTACGTTATAAGAGATAAAAATATAAAAATTAAATTAATAAATCTTATATAAATAATAATAAGTTTTACTAGAAAAATTCTGTATATTTTTCTCATAAAAACTAATACCATAAGATAGTACGATACAAATCCAATTAATAATCCTTAATATTTTCAAACTACATATGACCCAAAGGGGTTGTTTTGTCTTCTCTCTTGCTAGAATGATAAGTCCAAGCTCTAAGACTTTTTGTTCCCTCTGTATATTCCTAATTTTTTTTTATTTGGCAGATCTGTATATTCCTCTTTGGCTAATGTGATAACCTCTTAAGACATCTTGTTAATCATCGTCTTAATATTTAAAAGCGAAAAATATCAATCATATAAAAATAAATCTATTTATTTTAACATATTAAAAATTAAAAAAAAACTTTTATTATTTAATA |
| >CrNIP2;1-Pro2000  AATATTTTTTATTTTAAAAATATTTTATTTAAATAAAAAAATTTAAATTATTTATATTTTAAAAAAATTATTTTTAAATAATCATATATTTTTAAATTTTTTTCTATTTTTTAAAAAATATAAATATATAAATTTTTTATTAAAATGTTGTAGAACATATTCAAGTAACGTAATATAGAATAAAAATCTTCGTATTCAAACACAAGGCTAATTATTGCATATTGGATAACTTTAAGTGTTATCATCAATTTTTAGCAAAGAAGAGTGAATTGTAAGAAGTATTGAGATTGGATGCAAGACTATATTCATAGCCGTGCACATGAGGAAAGTGCACACCTTGAACGACGAAACAAACACTGTCAAGTAGAATTTTACACATCTCGACATCAGAATTTAGTTTAGGGTTTTATATATAAATTTGATACGGATGAGAGGATCTTTTATCCAAGTCTGGCTGTGTTTCTTTGTAAAGAGAGTTTTTAGAATCAAACATCTGCACATCATTTAGATTCATATATATTTTAAAATTTTAATTAATGTTGGTAGTAGATTATATAATTAATTACATAATTAAATTAATTATAATTTAAAAATAATTATAATATTTTTTAATTGATTACTACTCTATTAATATACGGTAGTTGATTATAAAAAATGTATAATTTATTATAAAATATGTTATGATTATTCCTGTATAAAATAATGATTAATTTAGTTATATAATTAGTTAGGTGATAAATATCTATATTAATTAGTGTACATATAAGTATAATTAATTAATATTTTAAATTTTAATATATATATATATATATATATATATATATGAATGTAATTGGAGTGCATGTTTGATAATTATTTTCTTTGGGCATGTTGGAATATGAAACGGGGGATTTTCTTTACACAAAATACTCGTACCCTTTGATATTCTCTCTACACTACACCTATTACTGCCGGTCAGTGCCTCCGCGGAGGTTCAAAGTGCCTTCTTGCAGAAGTTCTTCTACTTCAATTTTTTGTCGTTTGTATAGGATCATCATAAAAAAATAAGGAAATTGGATAATATCTTAATAATTGTATAGGTAAAATCAAATGATTTAAATTAATCTATATTGAATTTTTGTCTGCCATATTACAATTTTTTACTATTCTTTTTTTAGTTGTTTTTTATTTTATTTTATTTTTTTTACTCAAACCACTCTCTATCTTTACTATGATTTTTTTAGTTGTTTTTTCATTTTCATTTCTTATTTTTCTTAAATGCTTTGTACTCAAACAACAATCTCTTTTATCGTCTGCGTAGTCAAGATTTTGATTAAACTTAGTTGTTTGCTATAGATGTGGAAAGATATGAAATGAAAATATACCAAATTTTATATCAACAATTTAACATAATTTTCGTCAAATATCCAACTTAATTTTTACTTTTTAATTTATTACTATCTTCTTTTTTTGAAAATCACCTACTATCGTATAACAAGATAGGATCACATCTCATTTGTCACTATTTGTAATAGAATTACTTGTTTAATAAAAAAATATTTACACCAATCTTATTCAAAGGAATGCATTAATCAATGACTAACATGGTTATATATGAAAGACAAGTGATCAAATGAATTAGATAAAACAAAAATAGTATAAAATTCTAAGAAAAACCACAAAGAATTAAATAAAATTTAAAAGATGTGATTGTATTTATCTTTCTCAAGATAAGTAGCAACCCTAATATGATAATTTATTGGATGCGTGTTTAAATTTGCTTTACGATCACAATTAACCAAGAAAAAATTTGTTGGCAATTATTTTTTCAAAAATAGAAAAAAAAATATTCCTGTTATAAATTAGACTTTAACTTGAGCAAGGCCAGCAAAGACACATAAAATAAAAAGGTAAGTTTATGATTTTGACCAAAAAGCATGCAATGATACATGATAGAATGATACAAGGCCACAAAAACAACAGAAATTTATGTGTGATGATGAAAATTTTACATTCTAATT |
| >CrNIP2;2-Pro2000  AAGTTTTTTATCATTTTCTTCTTTTCAAAAATTATTATTCCTTTCACATCGAACACTGATGCAAGCAACAAATAAAATAAAAGCTTGTAACGAAAATGCAACAATAAAAGAAACTCAACAAAGCAAAACAGATGTGTGGATCAAATTAAAATAGAACCATTGCATGCATCAAAGCACAATATAGAAGAAGCTACCTTAACAAATCTTTGATCAAATCTATAACGGTCTCTTTTGATTTTCGATGAATGTCTATTTTATGGATCTACTGTGAAATGGTTGTCGTGAGAGAGATGATCGCATGCTTGTGGATGGTCGTTAATATGGGTATAACTATAGTTTAGTGTCCCACATTTGCATAGTACAATGAACAATGAATTTTTCTTTTGGTTGTGAAACCTGGACTGTGCTTACTATTGACATTCATCAAAATACAAATGTTTATGAATAAGGTAACAAAGATTGACACCAACCTAACATCACGATAAAGAGTCTTTTAGCTTGTAACGTAAATACTTACAATGTTCAATAAAATAGAAATGTTGGGAGCACACATATTTAATACTCATCTCTTTGTTATTATCTCTATTATTAATATGTAAATTATATATATGTTTCATTAAAAGTTTAAATATATTATTTATTTTTGTAAATATAATATTAATTTTCTAAAATAAACTTGATTGTTTCAATTTTTATAAAATTTAAAATTTAGAATAATTTGTATAATTATTTAGATCCCATTAGGATAGTTTAGTTATTTATTTTTTTAATTTTTAAATTAAATTTAAAAATAAAACTGTTCGATTAAAATTATTTTTTAATAGAATTTTAACATATTTTAAAAGAGACTTCTTAATCATTATTTTTAATTTAAATTTTTATCTCTACTTTAGATTTATCTCTTAAAATGCACCATCATCTCACTACTATTATTATTACTATCATCATTGACCGTTACCACTACTAATATCGTTATCATCTAAACATTACTACAACCGTTACATATAAAGATTTTTTCAATTTTTTTTATATTTTTATATTTTTATATTTATTTTTCTTTTACATGCATATTTACACACTCACAATGGGTATTTACCTCATTTTCAATGAACTTCTCCATTTACGGAAGAAATCTTCGATCCATAGTCCACAACTTTAGTAACTACAATCATCGCTAATAATAATAATAATATTTATTATGAAAAACTAATGAAGAAAAGGATAATCAAATTTATCTTGGATTTTTTTAAAAAGAAAAATTAAACGATGAAAAAAATAACCAAATTATTAATCAACCTTTGATAATGCATTTAGATGAAAAGAATTAGTTGCCATTATTTAAATTTTAGTAAGGATTTTTTAAAATATAATAAAGTGTAACGATATATTCTCAGGTCACGACTAAACTTTAAATCTGATACTTTCTATTTTCTAACCTAATATATTTTGTTTCCCATGTTTTTATAAGCAAAAGATATTATTAAATTTTTTTAGTACTAGGGGTGCTCATCCTTCTACAGAGCTGTTGCACGAGTTCTCATTTTGAACCTTGTTGCACCTAATATATTTTGTAATAATTGAGATGTAATTCTGTGACTTAAACTTTGAAGCGCGTTCACACACAAAGTAACTTTCTACCATTCTTTACATTCCAAGATTAATTAAAACAAGAAATTAAATGGTGGGATTTTGGGTGAACTACACGGTTACACCCAATGGTTCTTATTTATTTTTAGCTATAGTAGCATGTGTACAATATTGAATGAGGATGAAGTTGTGGTAGATATTTTCACTTAAAACACCATAGTGAGTATAAGCGTTTTTACTCCAATGGTTGCTCCTGAAATAAGCTAAACCATCAATATCCACATTTATTTCCTATTTATAAGTATGTTTCGATTGCAAACAAACTTTCAACCATATTTCGAGCTAAGAAGCTCGTCTTAATTATCAAAAGGAAAGGAAAGAAAAATAAAAATAAAAATAAAAAATACCAAAAA |
| >CrNIP3;1-Pro2000  GCATAGTTATAATTTTTCATCATTATTAATTAAGAAGTTTGTTTATATTAAAAATACTAAAATATAAATCACAATTTTTTTTTAAAAAGTGCATGTTATACTTTAAAATAAAAAAGAAAAAAGATATTTGTTTATATTAAAGATAAATGTTGAATTAAAAAATGAAGAATAATATAATAATTCTAAAAACTTAATCAACTATAATAAAATAATAGTGTGGATGATTATGAGGAATTAGGATCTCATAATCCATAATCTTTCTATAGAACCTCATAAATTTTAAAGGTGAATATACTATTTTTATATTAGTATTACTAATTTAGATTATTTATATTTTAGATGAGTTAAAATCTATATAAATAATTTAGGAAATATTACATTTAAAATTTTTATTAATAAAAATAAAATATTTTAAATTATTTAAATAAAATATTATAATATTAAATAATAATATAAAAAATAATATTTTATAATAAATAAAAAATTAATCAATATATGAAAGATAAAATAAATAGTTAAAATCTTGAAAGTTAGTGAATATATTGAAACATATGTTAGTTACTTCATTTACTTGTTAAAACAGATATTATGTTAAACAAAATTAAAATTATTCTAAAATTATTTTAGTTAGATTAAATATACTAAAAAAAGGAAAAAATATGTTTGGAACTAATTAAAAGTTTATTCTGTATGTTGACACTCCATCTCACTTATTTTTAACATGAATTTGATACATCGGGTATTAAAATATTCAACAGGAAATCATTTGAATATTCCATGGTAGTTTTATCGAGGTAATTTTTTCGGTGAAATAGCTCTCAAATTAAAAGGGGGTAAAAGTAAAACCAGGATTGATTTTTCATTGCTTACCTGAAAAAAAAAAGCCTAACGATCTGTAGAAAGTGTGTTCATACAAAAAAAATGGTAGACATATATAAATACAAAGAAAACAATGACATAAAATAATTGATGCGTGATAAAAAAAAAAGAAAAATATTTTGCAAAAAAATATTATATAAATTTATTGTATAAATAATATATCTTTACAAATTTGAAAATAATATCACTTCATAGCATGTTAAAAGGTAATATGATATACTATCGAGGATATGATCACGTGATATAATGTTAATTCATCTTAATTAAAAATTTTAGATTTGAATTTTAGATATGTAATAATATTGAAAATAAAAAAAATAACTTGATTATCTATATTAGCCCTATTCAATTTAGGCAAAATTATTTCAATAAAAAGTATATGATTTTCAAAATATGATATGATAATAATTAATTATTAATCACATAACATGCATAGACAAAATATTAGTTGGAATTTCATAAACAAAGGTGAAGAAAATCTGATGATCTCATGCAAAAAAAAGTGTAAACAGAAATAGAAAAAAAAGAGTAAAAATATATATAAAATAAAATAATTGATATACGATAAAAAAATATTAATAAATTTATTGTAAAATAATATGACTCTACAAATTTATCCATAACCAAGCATATAAATTTTTAGCAGATTAAAATGCGATATAATGTACTATTGATACATAAATCAATTTGTAAAATATTGATTAAGCTTAATTAAAAATTTTAAATTTAAATTTTAGATATGCAATAATGTTAAAAATTCAGAAGAAAAACTTTACCACCTATATTAGTTAGGCATGAAGAAGATACTTGTTGAAATTTTATAAGAAATAAAATTGAAATACTATATTTTTTTAGTAATGTTGTCCAATAAAAACTAGAATATGACATAATATCCTATACTAAAATTTGAATGTGGAAGTTAAGGAGGAGAAGAAGGTGGAATTTTAACTGGAGGAGAAAGGAAAGGAAGTTATGATAGTGCATCACTTGAAGTGATTGGTAAAAGAGATGGAGTTAATATGGAATGAAGAAGGGGGGATCTGGGGAAGAAAGAAAGGGAAATAAAATAATGGAAATGAAAAGCATGCATCATGCATCATGCATCATTGATGGATGCCTAA |
| >CrNIP3;2-Pro2000  TAAATTAATAAAAATATAAATATGAGTAAAAAAAATTATTATTTGATTAAAAGTCATTAATAAACTGTAAGTATCTATTTTGATTAAGAATAAAAATAGTGTAAGCTGAGTTAGATTTTAAATGTTTAAATTTTGGTTATTTAAAAATTTTGAGATATAAAAGTGAATTTGTTATTTTTTGTTTTTTTTAAAACTTGATTTGATTTAAAATTTATAGAATTTTTCAAAAGTTTATGTTAAAAAATAAATAAAATTAAAGTATACGTAAATAATGGATTAAAAAAGTTTACAAATTTAAAGTTTACAAAATAAAAAGATGATTAAATATAAATATAAATAGATCTGAATAGGTTAATAGATTTTTTTTAAGCTTAAACTTTATTTATTTATGTTTTTTAATAAATTCGAGCCTATTCATTTATTAAGATAACAGAATCTTATGCTTTAAATAAGTTAAATCATAAATTTCTATTACAAGATTTTTAACATCAACACATTGAAAATATATTATAAAAGCTTATTTATATATAGTGAGATATATTTATATTAAATATTAAATTTTATCATTTACATTAATTTAATAATTTTATATAAAATATAAAATTTATAAATTTAATTATTAATTTAAAAATTATTATTAAACAAGTTATTTATATAAAATTTTATATAAAATAAAAATTAATAAGTATTTTATAATTTTATTTTAACAATTTATATATTGTTTAAATATATCTAATAAATTTTATATTTTTCATAAAATTATTAAATTGATATAATATTAAAATTTAATATAATTTATATATATATATATATAGATAGGTAGATAATATAATATAATATAATGAAACGTGTTTATATCTTGGAATGAAAATTTTAATTATTAAATATTTTAAAATTTAGAATACCAATTACACGAGTCTTGACCATCAGTTCTCTCTTTCTATATATAAAAGTATATGAAATATACCTTTTTGATTATATGTTTATATAAATCTCACATATGATTATTAATTCAAATGTTTGACTAATGATATTAATAAATGAAGCACGCACATATAAATTGAACCGAGCCTCAATTGGTCTGAACTCAAAACAAATATATTAACAAAATTACCCTTAAAATTTTATTTATTTACAAATCTTCTTTATTAATATTTATAAAAAATATTATTATTATTATTATTATTATTATTATTATTATCCAAACAAATTTATTTTTATTGCTTCAACTGAAATAAATAAGAAACTTTTTCTTTTCTTTCTTCCATGAAATTATCCTCCTTTCTTCTCTTTTAACCAAGCAGAAAAGTGTGTAATTTTCCGGTTTTATATTATAGCAGGAAAAGAAATTGGATAAATTTAAGTATATATTCAATGAGGAGGATACACCATCCCCTTCAAATGTAAAATAATAAGTAATAAAATAGAAAAAAGAAAAGAGAGTAAAGAATAAAATGGAAAAGAACGTTAATATATGTTTATCATTTTGGTGGGAAAGAAAACTTATTGACCACTGTTGAACACTCTTCATGAACAGCATCTTCCAAAGATGCAATATACATAAAGTCTTGTGTTTAATTAGTATTAGCAACATTACATGGTATATTACAAACAAGACAAGATGTGTTTGGTGGCAGTTTCCTCCATGCTGATTGCTGAGACAAACGCACCTGTTACAGTATTGAGGAGGAAGGAACTGAGAAAAAAAAGGGATGGCCAAGAAGAAATTGAAATGGAAATGGAAAAGAAAATAAGAAAAAACTGAATGCACATTGAATGGTACATATGGAAAACCCATGATTGATGTCTGACGTTTAGGGTCTCTGCATTTGTCCCACCCTCCACTTCCAGAACCAACTGCTACCTCATCTCTTCTTTTCTCACTCCCATCACCTTACTGCACTACTTTCCTTTCTCTTTCTTTTATTTGGATTCTCTAGTCAACTATCTCCCTCCTATATAAATAACATGCCTCATTGGTTTCACTCTCACCACAAATA |
| >CrNIP3;3-Pro2000  TTACAACTTTTTTAAAATAAAATAAATATTTATATTTTCAAATTTAATGTCATTTTTACCATATTTATAATTAGATAAGAGATATATTCTTAAATTTAATTAAAGCTAATTTTTTCGTTAAGGCCAAAGATAAAGTTGAATAGATAATTATGGTTTTGAATTTTATTGCCCTATCTCTTTGTGTATCACATTACCTGTTAGAACTAGCATAAGAAAAATGGTATAATTTAAATAACCATCACTTGAGAGAACTAGGTTAGGAAAATTTTCATCACAAGTCAAACTACATACTGCATCTCAAAACTTGTTACTAAAGTCATATATATATTTTTAAATATTAATATAGTTTAAAAATTATAAATAATTATTTATTTAATAATTATAAGAGTCAATTTAAAATTTATAATTTTAGAAAATATTTAAAAATAAATATATGAAAAGAAATAAAATTATTGATGTATTAATTATATTTTTTATAACATCAATTACAAGTATTATATATCACATAAATACATATTAAATCACATCAACTTAGATATTGTAACTCAGATTTACAAATATAGCAGAAAAATACCTTCATTAAAACTTAAGAAACAGTAACATGCGTCCGTCGTTTCTGAGTAATTTTAAATTAGAAAAAAAACTTGTTTAAATAATAAATTATAAAAATAATTATTTCACATAAATACAATAAAATTATTTATTTAAATTTTTAATTTTATTTATAGTAATTAATTATATATTTTTAATAATTAATTACTTATTAAATATATAATTGAATTAACCAATATTTTTTATATATTAATTATAATATCTATTATAATCAATTATATATTTTTAATAATTGATTATCAATATTAAAATTTAATATATAAATAAAAAATTACTGTTATTACGTGAAAAAATATTTTTAAATAAATTATTAATCAAAAGAACATGCATTTTGAAGTTGAGATTTTAAAGTGTTTGTAGTTTTTAAAATTTTTTAATTTTAAATTAAAAATTCAGTAACTTCATTAACATTATAACAATATATATAGTTATTTTATAAATGACGCACGATGCTTAAAACTATCGTGTCGGAATGAACTTCAATTATAATTAAAAACAATATTTGCGATTAAAAATATGGAAAGGCGTAACTAGTATGTGCTGGAAAAGAAAACAAACAACTAACATCGTATAATATAATATAACGTAAGAATAAAATGCTTATTACCAATATAAAAAACATATAGTATACAATCATTACCTGAAGAGGAAAGTAAAAGCAATAATGCGAAACCTTTGACTTTTTTATTATGATATGCGCTGTATTAATCTGATAATAATATATATATATATATATATATAATATCTTTTCATAAGTAAATTATTATTTATTTTTTTAATTTTTTTGACTTAATTAGTTAATTTTTAAAAGAATAAAATTATTATTTTTATCTCTAAAAACATAAAACATATTTTATTTATTCACATTGATTTACTTTTATTAAAAAAATTAAAAAAATATATGATCAATTTTATTTTTTTAAAACCAAAATGCTATTTTATTTTATTTACTAAAATTATATATATTATTTATCACAAGCTTTTTATTTCTACGATATTTTTTAAAAATATATAGATAATAAAATTATTGAGATTAATTTAATAATAAAAATTCGAATAGTTTATATATTACAAGTATGAAAAAAATTAAAGAGACTAAACTCTAACTACCGTTTATGTATTATTTGTATTTAATATAGTTAATCTTATTATTTAATTATTGTTTAAGTTAAATAATAAAATAATGATAACTTTATTAAATAATCAGGATTTCTATTATCAAATTTTAAAAAATATCAAAAAACCAATTTTTTTAATCATTTTCTTATACCTCACCCGATACATTCACCTTATGAATGTATAGTGACGATTTTCCTTCGTGTATTATGCAAGGAACGCGCCGACTCGTTGTTCTTAGGACTTCATAAATTGATTATTAAAATCTATAT |
| >CrNIP4;1-Pro2000  TTTTAAAAATATAGAAAATAAATCTACAGTTACTTGAGTAAGTTTTTATAATAATTTCTTAAATTGTTATATTGATTGTAATTTTCAATTGATTAATAATATAGTTTTTATATTAATAATAAATTAAAATTATACTTTTTAAAATTACGTTTTAATGTTATATGGATAATATTAACAATTTATATAGTCGGTAAAAAAATAATATATGCTAATAAGTTATAATTGGTAATTTATATTACTATATCAAAATTTAATCATTTTATTGTTCATCACGTATAATTCAATTAAAAAAAAAACATTTTTTAAACTTGAGTTGGAGTTCGATATATTGTTATTAACTAGATTTGATATGATTTACGGGTTTATATGTTCTTTATTTTTATATTATTAATAATTCAGAATATATAGTTTAAGTATTAACAAATTATGACATTAAATAATATTAAAATGATTATTTTATTAAATTCTAAAACAAAATAAAAATTATTATTATTTATATATTGTTAATATTTAAGGAATTTAATATTATAAATATTTAGAATACAAAATTAATATATAGTATAAATTTTTAAATGATTAAAAAAGTTAAATATTAAATTTTGATTTATAAACTTATTTGTATATATCATATTTTTGTTTTATATTATAGTAATAATTAAGCATATATAACAATTGATTAATAAAATAACTTACATTGACCTAAAGTTATAAAATAACTAATTCACCAATTCCTTTTCTCATTGAGAAATCCTATTTTTACTATAAATGGATACAACAAAATAATCTAATAAAGAGAAATAGGAATAGAAAAGAGAGAAACATGTGATATGATCCATGAATGATGTGATAAAAAACAAAAACAAAAACATATTATACAGATTGTAATAGAAGTTTGTAGTCTCTCTCGGCTGGAGTAGTTGCAATGCTCTTAACAATGACTTAAAGTTTGTGTTGGTCTACTTCAAAGTTCAAACATTAACATGTAACTTTTCCAAGAATAGGAAACAGAACATGGTTGTATTAGAATGAGGAAGTGGTTGTTGAGATCTAGCTTTTAGGTTGGATTCTTCATAATAAAAGATGAGCAAAAAGTTAGAGAAGAGAGTGATAAAAGGTTTACATTTGTAGGGAAGTAATAGTTTGCTTTCTAAGCTGTAGCAGTTAATAAGCACCAAATTGGTCTTCTCTACCTCACTAACAGCAACACATGAATCGTGTGTTGAAGCCTTTCAAACAATCTTTAAGAAGCCAAAGACGTGTGAGCAACAAAAGGTTCCAACTTCCAACCATAGTTAAACTTCCCCCACATTTGTGCATCATGACTGTTCCATGGAATGCTAGAACTCTACTTTTTCTACACAACGTGTTCTTTGCACTTACTATACATATATGAGACCTTATTGAAGATTGTGTTCATCACAAGTTTGATGATGACTGACATATTTGAAAAACAACCATCTTCTGATTCTTCAAACTATGCATCAAGCAGTGGCCTAACTGGTGATGATAAGGAGAATGGATATAGAGCTTCAACATCAAAGCATCGATATCTTTTGGCCAACAACTTTTCTCTTCACTTCTTATCCATCAAAATTGACCTCAATTTTGCTCGCATGGTAAGAGTATATTCAACCTTTCTAAGTGTTATTTTGTCAAGAGTTTTATTATCTGATTTTAGAATACATTTCAACCTAGCTACAAGCTAGCAATGCTTGATACTTTACATTATAATTCTAGATATGTAAGGATGGAAAAACAAAATAAAATAAAAAAGTAAAGTGAATAGCAGCATCATATGGAATGTATGTAGGTAGAAATTTGGACTATTACTAATGCAAGAATGGTAAGTGTGATCAAGAAGCAAAACCTTGTAGAAGCTATAAAAGAATTTAAACATGACCCTAACAAAAACCAAATATTTTTTGCTATTATTTTCACTTGGAAAAACATTATAAATGCTTGATAATATCAGAAATATAAGTTGTTTTGAAATATAGGTG |
| >CrNIP5;1-Pro2000  TGTTGCATACACTTTACTGTAATATAAATAAGATAAATTATATTGATATTTTTAAAATTTATAAAAAAATATTTATATATTATTGTATAATTAAAAAAATAATATTATATATTTATATAAATTAAAAAATTACATTAAATAAAAATAGAAAGGATATAAAATAATTTTTTATAAATATCAAGCAAAAAATCAATCACCGTAAGAGGAATGAAAAAACTCTTCTTAGAGTTAATGAAGGTAATTCATCTCATAAGGTAAGGATAAGAAAAGTGAAAAATACTACAGCTAACGAAGTTGGTTAATCCATTTAAAATAAATAAATTTTTTTAAGAAATTAATGTTATTTTTATCCATTTTATTTGTGATTCCTGAATTTGTAAAATTTTTAAAAGAATTAAATTTTAATAAAAATTATAAAATAAAAAAATAAGGATATAATAAAAAAATTTAAAAGTTATAAGAAAAAAAAACTTTTTAACCTTTTATATATATATATATATATATATATATATATATATATATATATATATATATATATGATAACGTTATTTGAAAAAACTTTTTAACCTTATATATATACAGGAGCAACGTTATTTGAAAAAATTAATCCTAAGTAAAAATTTTATTAGAAATCAAATTTTGAAAATTTTAAGTTAACTAAAAATATATTTACCAATTTTATTATTATCTCATACTTTTCATTTATTTAATAATTAAAAAATAATTTGTAAGATTTAAAATAGCATTTAATTAGCGAATATGTCAATATCAATTCTTTTTACAGTTATGTAAGAATCTAAACTATTCGATCCCTTTGTCTTAATATTAATAATTTAATGCTATCACATTGTTATATAAAAAAATATTCTCACGTACATATAGAGGAGGAGTTCTATATATATATATATATATATATATATATAAACTTGTATTGTAAGCGTCACTAACCAATAAAAAGCAAAAAGTATACATAGATTTATTTGTTTTTAATTTCATGGAAGTCATTGTCGCGAGACTCAAATAACTAAATTCAAAATTATACTTATTGTCCCCAATATTTGTTAATATATCATTAAAAATAAAATCATATTAACCCGTGGTTTCAGTTGACGACGTATACACTAAGTCGTATATATCTCTAGCCAATGACTTGCCCTATTTCGTATTAAAAGTCACGTCAAATAAAATGTCATGAGATTCAATTATAGGGTAATAATTATGATTTTTTTTTTACTATATATACTCCAAATTTTAGACCCATCTTACATGTCTATTTTTTTTAATTTACTTCTGTCATCTCATCGTTTAAAAAAAAATTTACTTATAAAAAAATTATAGATATTTTAATAATATATTTTTAATTAACGTTTTATATAGATTCACCGTGCAAATTTGGTAAAATTCCAGGAACAACATCGCGCCCCAGCATCCACTACAGGCCGCATCATACCCACCGTCAACTTCCTCTCCATCACGAGCTACTGAAATCATCATCATTTCGGAAGGACAAACGCACTTTGAAATTGCGCTTTAGATATATAACATTTTAGGGTTTGATTTATTAAAGGAACCGAATGTCTTTGTTTCAAAGTTGGGTTTGAATTTAACTTACAAAATTAGAATTGACGAAATTTTTATAAAATTTACTTTCACCATATATGTAATTAAGGTGTGACTGACTTGTCAACTTCGGCAAACTTGAACCATTTTAAAAAATAAATATAAAATTTCGTCAAATTAAAAAAATACGAGAGTCAGACTAATAATGCTTATATTTTATACCTCAAAAATTTGATACTCATTGCACATGTCTAGTTTTATCTTTTATTTTTTTCCTCTTCGCACTATTTATAGACAAAGCTTATATACAGAATCATAAGTAATATTGTCAAATAGTATCCTTTGGATTAAAAATATAATATCAAAATATCTATAATACAACACATAAATTTTGTCCTTATATATGCACATATCATTTTCTCTTTTCTTGTATCGTATCT |
| >CrNIP6;1-Pro2000  AAATTTTCAACTAACCCGATTTTGTTAAAGTATTGTTTATTTTTTAATTTAAAAACCTAAAAATACCCCCTTTGTCAATAGAATGATGAAATATGCGCGTTTCTTCCTAATTCCCTTCATTCGCCATATTTTAGGTTTCAAACCCTATTGCTTAGACCCACCCACCTTGTTCACTATTTTCTTTATCATCATCATCCTTGATTGCAATTGATTATGATTTTGGGTTGTAGGATTTGAAATTTATCTTATTTTATTTTTGAAACTGTAATACCATTTATTATAAAAGATATTATTTATTAATAAATAAGAATTATTAAGAGTTAAAAAATATTTTATTTGTATAGATGTTATCTAATAAAATTAATGCGTTAGTATAGTTTTTGAAACTTTATCCTAATAATATTTTACATTAACATTTAATATTTTAATAATGTACATTAATGTTATATACTAATGAAATTATTTATTAATACAATATATTATACTATTAGTATAAATTTATAATGTTTATATGATAATTTGATTTTTGTTAAAAAAAATATATTAACAAAGATCAATCTAATATAAAATTATTAGTAAATAATTTATTTGCAATGACTCCTATCCTATCATATACATTTATCAAATCATTTTTTTACTCTTAAAAATCTACTTTAAACTACCACAACAAGCTATTAAATATCTAATAAGAAAGTATTTAGATACTTTATATGAAATTTTATATTTAAATTTTATTATTTTTATTATAAAAAAAACACTACGATCGAGATCATAACTCATGAATAGGTACACCTCCACGTATTAGAAGCTGGTTTTAAATCAATGAAAATATAACTTAATTGTAATTGTAATGTATTTATAAAATAAGAAGTGTAAAATATAGAATTAAAAAATGTTAGGATAATTTACACTTAATTCTAATTATAATTGTTTCTGCTGAAACTATAAAATTAAAATGGTTAACAATATTTATACCATGCATTTAGAAAAGGATTCATCTTTTCTTAATTAAAAATTTAATATTTCAAAACGAGGATTTCATTTCTACTTTTATAAACTTAAGAAGTTGGTAGAGTACCGTAAGATAACTCTACATATTTTTTTATCATAAAAATAATAAAATTTGAATTTAAAATCTTAAGTAAACTATCCTAACATTTTACTATTAGATTAATCTTAATAATTTTTTTCCTATTTAATAGATCACTAGTTTTAATATTTTAACAGAAAAAATAAGTAAAAATGCATGTAACTTTAATTTTTTATTAATAATTAAAGGAATGCTCAAACGGAAATTTGGTGTAGGATTAGGCGCCACATAATTAAGTTTATATAATAATTTTTTATAAAATAAAAATTTAATAAATTTTACCGATAATATTTATCTATATTTTAAAAGATACATTATATATTAAATTAAATTTATTTGATGAATATCAAATAAATTTTATATTTTATTACATTCTATAAACAATTTACATAATAATTTTTTATCAAATAGGTACATAATTATTAAAATTTAATATTTAAAAAGTTATGAAATAAAATAAATTTAATAAGAATTACTTTCTAAATAAGTTAATGTGTATAATTTTTTTAAATAGAAAGAATTATTAAAATAAAATAATTCAATACCTTGACGCTAGCTAGTGTCGCCATAGTACTGTACTTTCTATACGGCCAATATATCAAACAATTGATCTGCATTATAATACCATAGTACAAGTACTAAATTCAAAAACACCCATAACGGAATCACCCCTCTCATAAAATCTTTTCTACAGATTCAGATTGTTCGGTTATATACGAATGGTCCCTGTGGAATGTTTTCAGCTCAACATTACATTATTTGCCGCCCAAAGATTTGGCTTTCTCTCTCCTCTTCCTGTTGCCAATTATCAAACCTTAATTATGTGCCACGTTATTACTTTTAACTTTTTCCCCTAACATTACAAATGGAACTCTCATATTGATTGAAGCTAATGAACCATAGATCATTG |
| >CrSIP1;1-Pro2000  GATGTTGAGTTTGTAAGGAGAACCTTAAAACATCCTTTCATAAAAGCTTGACCATGTTCAACACTCTACTTAGGCATGAATGCATTATGGGATATTACTATTGTTGAGTAGCTATGTGATTATGTGAACATATGTTTCCATAATCCACCATTTAATAAACTAATTTTAAAAAAATATGTTTTTCTAAGCTTCCACCATTTTCTAACGTTGGACATTACAACAGTGTAATTCATCTTCGTGGATACTGCATTCTATCAGCTACAAATCAATTTCTACACTTTTGGTTAATTTACAAAAATAAGATTCAAATTATGGAGATGTTGTTAGTTACGGAAAGAGGACATTGCTCTGTAGAGAACAGAGGAAGGAGCGGCGGATGGAAGTGAGTGTTGAAGGAATAAAGTGGTTGTTGACGGTGGAGGCACAATTTGAGAACAATGGTTTCTGAAAACAAGATGGCTGTATTTAAATCTCAGCCCTTGAATATTTTCAAGCCACGTGGCTGCCGATCCCGTTCCTACTAGTTTGCCTCCTTCATTTCGGATCAATTCTACTTTAGGTAGTTGACTGATCTATGGAAATATATAACATCGATTTTTTTTTTATAACATGTAAAGAGAATTGTATTTTCGATCCTTATATTTTAATTTTTAATTTTTATTATTAGATCGATTTTAGTGATTTATAAATTGAAAAGTAAAATAAAGCTTTAATCTTTTTCAGTAATTTAATTTTGATTTGTGTATAATTTTAATTTTATTCCTTTTAAATATTAATCAATTTAATTTTTTAAATTATTATTTTTATCTAAATTTTTGGAAAATTTTATAATAACACTGGAATAGAATATAATTTTAAAAATCTAGAAATAAAAAATTTGATAAAGTTTGAATGATTTTATAAAATGTAAGATTAAAATTTGAAGAAGAAAAAAAAATGAGACTTTTTTTTTAATTTTCGAAGAAATTCAACATTTTGGTTGTTGTATTTTTTTTTAAATAATTCAGTCATCTAATTTGTAAAAATTTTAAGTAGAAAATTAAAATTATAAATTTTAAAGTAAAAAAAATACAAATAATTAGATTTACTAAATTTTAAAAATAAAAGTAAAATTATTAAAATTAAAAAATTTAAACCTATACATATTAAATTAAGAAGAAAAAATACAATTAAACGTAAAATAAAAGACTTGAAATTTAAATTACAGATAACAACATAGCACAAAAAAAAAGTAAATCAGTATCTTAGTGATTGAATATTTTTTTTTATCTCACATTAATTTTTAATTTCCTTAAAATATTTGTACCAAAAAAAATTCTTTAAACTCATAATTAATGATTGTGTGCGAAGCGTTTAATGCTGTAACACATTAATGCTATGTTCGGTGGAAAGAAGTATAAGTGAGATAAAAGTAAATTAAACTTTAAATTAGAAAGAAAATGAAAAAATATTTAATTTTTTGTACCTTCTGATCAATCAAATAAAAATTATTATTATTATTATTATTATTATTATTATTATTATTATTATTATTTCCTTCTCATTCTTTTCATTTCAAACTAATCAAAGCATAAATATGTTGCCTAAAAAAATGTAAAAAAAGGAAATTTGATGGGAATTCACGCAAGTGATGGAACATGTAATTTAAATTATGCCAACTAACATTCTTTAAGATTTAACATGCGAAGCTTGAATATCTATTTTATAAGTACTATTTGATAATTTAGATTTAATTATATCTTATAATTTTAATAGTTATTTTGTTTTTATAATTAGAAAAATATGAAATTCAAACTCATTTGAATACATGTTTAAAAATATATGATAAATAATAAGTTTTTATTAAAAAATATAAAACAATATGAATTAGAAGGATTAAAATTTTAATTTAACCTTATTAATTATTATTTTAAAAAAAAAAAAAAACCGGCTATCACCCGTAAGTAGAGGTTTGTTAGGTTGAGCTTATCTGCTTATGCTTATGCTGTATGCAAGGTGCC |
| >CrSIP1;2-Pro2000  TATAATCATAATAAATATCATAATTATCATGATGATGATAATAAAAATTCATTTTTTTAATTTATCTAATTTTATAGATTAATAATACAATATATATTATATTTAAATTTATAATATTATAAAATTATTTTATATTGTATAATTATTTATAATATATTATAATATCTATATATATTATTTTGTATAAAATAAATATTGAATAATATAAAAATATTATAATAATAATTTATAAAATTATTTATCTTATATATTATAATATTAATTACCAAATAATATAAAAATAAAAATTTATATAATTATATAATATTTTTATATTATAACGATCATATTATTGTCATTATCTCACAAAAGTAATAATAATAATAATATTTTATTTTAATAAATTTTTTTATATAAATTTTTAGTAAAATAATAAATACTAAATTTAAAAAATTTAAATTAAAAGTGAATTTATTTTCTGAAAAATATTTAAATTAAGAATTAAAAATAATCCTAAATGATCCAGAATAAATGCATAGTTTAAATCACTCCTATAAAATCAAACCATAAATTTATTTTAATAAAAAACAATAGTAAACCAACAAAACTGTAAACTGATTTTAAAAAATATAAAAAATTTATGTATATATAAAGTAATTTTTTAATAAATATAAATTAAATTATGTTAAATTTTATTTAATTTTATATTAATTTAAATTTAAAAATTAAAATATATCATTTTTATTTTTAAATTTTATAAAATTATGATATTAATATATTTTATTGTTATATATACATATTTTTATATTTTTAAATATAATATATTTATAAATATTTAAAAAAATATTTAAAATATTAGTTAAACCATTAAATCTGTATTTTTAATTTAATTACCAATCCGATTTTAAATAGTTTGATCCTACGTTTAAAGTAACTTTGTTCATGTGCCACCATGTGATCGGTGAGTTCCTTTGAGAAGCAACTGCAATTCCCGTTCTGGAGATGCTTTAAAAGACATTATATACGAATAATTTATTACCCACGTTCTAATATCCAATGCATATCTCTCACACAATTCACCCCAAAAATATTTTATGTTGCATTGATCCATCATGTGGCTTAGTTTCCACCTGTAAATTATATTATGATTTTTTTTTTCGCTGCTTAGGTTTTGTAGAGATCCCATCCTTTAATACGTATTTTAAAAAACCAATATTTCAACCCAACTCACTTCTATTTCATGTTAATTTTAATTTATATGTTTTTATTAAAGATAACTCCTTTTTTATTTTTTGTTATTTCAATTTTTTAAAATTTAAGAAAAGTGATGTAAAGTAAAATAATTTAAATTTAATTTTTCATTATTTCATGTTTCTTTTTTAACACTTAATTTTATATTTATAAAAAAATATTTTTTTTTTCTTTTTGCTGTCTATCACATGGTTTGAGATTAAAAAAGTGTAAAATAAATTAAAATGTCTCGTTAACAACTTTTTCACGTCGTTTTATGTTTGTAGTGTAATATAACATGTCACTTTCACTAAAAGAAATCTCATTTTATAATAAAAATAATGAGTGTAACTGATGTAAAAATTATCAAACTTGAAGGGAATTTTTTTTGTGAAGAACAGAACAAAAGAAATAAAAAGTTGCCTCTTCTAAGTTTAAATATATTTTTACTCTCTGTATATATACACATACAAACAAATAAAAATAATTATTAATATCTAAATAATGAAATTAAAATTTTAATTTTTTTTTAAAGAAAAGTGACATATTTCTTCTCAATTATGATAGGGTACCCCACAAAGGACACAACCAGCTAATTGTAGTGTCTGAATGTTTTGTCGTTTTACCCACCAAAGGAGTTATTCACTTGCATCCACTGATTCTCCTTCTTATTCTCTCTCTATTCACTCTTGTTATTAGCCATTAACATTATAGCCAAAGTGTGTGAGAGTTGCAACTCCAAGCAATTAGGACATAGAGAGAAAAAGTTCCTAAAAAAAATTAAGGAGGAC |
| >CrSIP1;3-Pro2000  TTTCGGACACAGTGACCCCTAACTAATGCACCATTTAATTCTACACGTTCATTGCATGCCTCACCCAATCTATTCCCAAATGCTCTCACGTTCTTTTAGTAAAAATTGCTTATTATTACGATTAGTGGTAAAATTGTTTATACTTTGTTTCTTCAGAAATGGAAAAAAAGAAAGAAAATGAAAACAAAAACAAATAGAAGAAATTAAAATATTTTTTAATATTTAAATGTACATTCATATGAGAGGAAAGAAAGTATTTTTATAGGATATATTTAAAATTTAATCTCTCATGAATAGGAAAGAAAAATGGAGGAAAAAAGTTTTTTTTTAAGGTTTTAATTATGATATAATTTTTTTAAAAAATAAACTAAAAATTAAAATATTGAATTAATAATAAATAATATAATAAAATTAAAAAAACTTAAGTTAAATGTAAATTTAATTAATATAAAATAAAATTTATATTATTAATTTAATATTTTTCATTCATCTATTTTTTTTATTAATTTAAATAATTTATTTTCTTATTTTATTGATCATTCAAACAAAACATTATTTTTTAAAAAAATTATATGTTAAATACAAAAGTAAATCGGATATTTTATTTAAGCCAATAAAATTATTTAATAATGAGAATTTGAGATAATTTACTTGAATTTTAAGTTAAAACCTTATCCGTTTCACTATTATACACTACAAATAATGATTTTTTTTTTATCTTTTTGCAAAACTTAAATATTTACTATTGCATTTCATTTACCATCGATTTTAATTATTAAAATAAAAGCTATTTTACCGAAATAAATATTATTTTTTATTATAAAATAAAATTAATAATATTATAATTTAAGTTTTTTATTTAATACATAAAACATGGTATATTCCATAATTGGTATATATTTCAAAGATAAAATATATTTTTAATTTTTATACATATTAGTAAATTTAGTTTTAATTTTTTAAAATATATTTAGATTAGTTTATATAATTTTTTAATTTTAATATTATTTTTCAAATAAATCCAATTAAAATCACAACAAATTCTTTTTAATTATTAGATTAAATAATATTTTATCTTAAAGTTGATTCAAATTGAACCACAGACAGCTTTAACATTTATCATCAGAAAAAAAATCAAGAACCAATGTGGTTCGTTTTTTTCCTTTTTAAAATAAATAAAGATAATTGATTTTTTTTGTGTGTGTGTTTTACTTATTTATCATTATTGAATCTTAAAATAAGGAGAAATTAAAACAAGCAACTGAAATAATTATAAAAAAATGAACATAACCGAGTCTGATTTTATTTTGTATGTGTCTAGCCAATATGTCCAAGAAAACTGTTTGATAAACTATTCTTACGTTTTTGAAGATTTATTTTTACTGTATAAACTTTATATTTCTTTATTGCGTTCATTAATGGAACCTCTAGCATAGCCAACAAGACCCATTCAAAGTTTTCATTTTCCACCAGTTTCGTTTGACCCCGGAATCAAGAGATTGCAAATTTGACAAAGATTTCCATTCTCCAACATCACCCTTGTTCTTCTTCTTCTTTACCCTTGTTCTTCTTCTTCTTTTTTCTTTGGGTCAACTATCCCCACTGGTCTTGGGATGCTTCAAAGAGACATTATGATTAATCGGAGTTTACTGAAATAAGAGACTCAACAAAGACTAAAATTATTGACTGCAAAAATTAGTATCTAAATTTAAATAATCATATAATTTTAATTAATAATTACAATTCTTTATTTTAATCCTTAAAGTGACTCATTAACTTAAATGAGTTAAATGTGGTTAATCTATTATCTATAATATATATATATATATATATCTGCATTAAAAAACTAAAAGAGAAAAAATTATTATTTAAGTATAATTATTTTTAATGAAAACATTTGAGATTAAAAAGAAACTATTTATCCACATTGTAACTGGCCCAAATTGGATTATGAATTTTTCTTGTGTCAACGCATAGTTTAAGCATAGATCCATACATTT |
| >CrSIP2;1-Pro2000  ATAATAAATATAAAAATTTAAAACTAATAATCATAATTTAAATCTAATAATAAAACCACCATATAAATTTAGCAATAAAAAATCATTTCAAATAGTAGTTCCATATTGTTATCAATTAATTTGAAATTACAAAAATTTTAAAAATTCATAATATTTTTATACAAAAAATTACCATCAACTAATTAAGGTTTATTATATATAATTTTATATTAAAAATATAAAAATCTTAAAATTAATTATAGTATAATATTAAATATTTAAATGAAAAATAGTAAATATTGAAAATTATGATCATCTAATGGAAATTATGTTGTTAATTTTAAATTTAAAAAGGATAATAAGATTTTATTTTTAAAGTAATAAAATACTTTTTAATTAAAATAATTATTTAATTATTAATTATTAGAAAATTAAATTTATAAATATAAATAAAATTGTTAACTATATTAATTACATTCTAATAACATCAATTACAAATATGATGCATAATATAAATATATTAAATCCTATCATCTTAGATACTATATATTCAAAAAAAAATTTAAAAATCAATTTATAAATATAAACAAAAGTATTAATTGTATTAATTATATTCTAAAAATAATTATATTATAAATATGGTATATAATATAAATATATTGAATCATATTATTTTAGTTATTATATATTAATATTTTAAAAAAGATAAAATATTTTTTATTTTATTTTATGAATGTATTTGATAAAATAATGAATAAATTAGATAAAAGTTAAAAAATGAATTAAAATAATATTTGATAAAATTATATATTAAATTAATTGATAAAGATAAAATGATAAATAAAAATATATTAATTAAACCTGAATTTAAATATTATAATATATTAATTATTAATTTTATTATTTAAATTTAATTATTATATTAAAAGTTAGGATAATATTTTTTAAATTTATTCTTTTTTATATAATTATTTCTATTACAAAATATTATATAAAGAAATGTTCAATTATTTTATACTTATATATTATAAAATAAAAAATTTAATGATTATATTTATTTGTAATATATTTTTAAATAATTTTTTAACATTTTATAAATATATTTTAAATAATATTTCTATAAAAATATTGATTTATTTTAAAAAATTTAGCCTCAAAAGTTAAATATTTTATTATAATTAATTTAAATAAAATATTTAATAATTAAACTAAATACTATAATATTAAATAATAATATAAAAAAATTTAATAAAATATTTAACCTTTTTTTTATAAAGAATAATTTTCAGATTCTAATTTTTCTATAATAATTATATATTTTTTTACTTAAATTTTTTCAAAATAATTAATATTCAAATTATAAAATATTTTTAATAATATATTTAACATTTTTTTTAAAAAATAACTTTTATATTCTGATTTTTTAAAAAAATTATATTTTGAATTTTAATATTAACATTTATATTTTTTCTTTTTACTTAAATTTTTTTAATGTACTTTTCAAAAATAATTAATATTAAAATTTTAGAATTTTTTGCAATGTATTTTTCAAAAAAAATTATTAAACTTTATCTTTAATATTTTTAAATCTTTACTTTTTCTCTTTTCTTTTTTCAAGTTAAAGAATTTAAAACCAAAAATCAATTCAAATCTTAATTATAAACTATACTTCTAAGTAATGTTCTAAATTGTAAAAAAAAATTCTCTCATACATTCAGGATATATTATCATAAAACAAGAAAAAATATGTAGGATCAAATAAAATAATCCTTATATTTATATAATAAAACATTAAATTTTTTTAAAGTTATAATATATAAATGTATACGTCACATAACATATAAATTTATGTATTATATAATTAAAAATAATTTAAAAATTAACTGTAGATATAAATTTATGCGTCTGGATAAATATATATTTTTTTTAACGTAAATATTTTTTATTTGAACATGACTTTGGTAAAAAACCAACATATTGTATATAACTGGTATCCATTGAATTAGTTTTCAAAAACTGGC |
| >CrXIP1;1-Pro2000  TATGTTAACTGAGGTAAGAATTAAATATTTAAATCAAGATTGATTTTATTTAATCGTATTCTGCTTTAATTTAATGTTCTTTGATAAATTAAAGACCATGTTCATCACATACACAAAAAAAAAATGTTAAATTTATTAGCGGCTAAACATTGTGGTCAAGATGCCAAGTATTTGAACAAATCAATGCCATGCAAAAAAGTTAAAATTGCGCATATTTCCGATCTTTGAAATTGCAGAGATTTCTGTTGATTGATCCTGTCAAGGCTAATCTTTTCAGTATATACCTCAATTATGCTCTCTAAATTCGATGCCGTGGCCTTGAATAAATTGTGAAATACGTGCACAACAATGTGTGTATTCATGCAATCATGCAAATTAAGGTTGGACAAATTTTTACAAAAAACGTTTCCTATTTTTATTTTTATTTATAAAAATATAATTTTGTTTTTATTTTATGTTCTGTTTTTAAAACTTTGTGTTGAATACTGTAAAAATAGAAAATAAATATTTTCTATTTTCACATTTTCTTATCTAAAATATAAAATAAAATAAAATTTTATAAATAAAAAATATTTTCATAAACAAATATCACTTAAGTGTGTGATCTAAGTCTGGTTTTCAAGAGTGAAATTTGAACTTTCCTAATTTCATTCCAATGACTACAATGTACGTGAATGTTTGAATGCACGAATTAGTGGTAATGATGCAATTAAATACCATTGGATTTCAAAGATAAAGATTTGAAATTTATTATTTAATATTATTTCATGTATGATGAGAACTTTAATATAATATTATTTTGAATTAAATGATTAAATTTTTTATAAAATTTATTATTTTGTTTGCAATAAAAAATATTAATAGAAAAATCATTGTAATTTAATAATTACACTTTTGTCAAAAACATTCTTTAAATGATATATTTTTAAAATAAAAATGAAACTTAAAATTGGATTCAACATATCTATTTGTTTTCAAAGTATAAATCACAAAATAATGTTTAGGTTAAACATATTTTTAATCTTTCAAAAAATTTGAAACTTTTATTTTTTTATTTTTTTTTTAAATTAATTGATTTTATTCCTATTTTTTAAATTAAATTGCTTTTAAACTTTTATTTTTAAATTAATTGATTTTATCCCTATTTTTTAAAATAAAATTGCTTTTATCCTTAAAGTTATTAATGTTTAATACGGGTATATAACAATATTTTATAGAAGAGTGACTGAAAGTTAATTATTTTAAAAAATAAAAGATCAAAATTAATCATTTTAAAATAAAAAACTAAAATCAAATTTTAAAATTTTTTTAAAAATTAAAAACATATTTAACTCATAATATATATTTTTTAACATGAGAGAGATATGACTTTCAAATTAAAAGAACAAATTTGATAAAGTTTATAAGAATCAGATTCATGATTTAGAAGGTTAGACACTCAAATCACATTGTCTAACAATTTTGTTGACAAGGTAATTGACAAATATCTTTATAAACACCCCTTTAAATCATGAAGCATATCTTGGCCCAAAGAAATCTTGAGGAGCTATCCCATCTAAATTTTTCCATACAAAGAATCCCTCGTTGTTGGAATGGTGAACTTGCATAGGTCCAAAATTTGGCTTTGTTCACAGCGAAAAAGTTGGAGAGAGCTAGAAATCATATCTCCTTGTTTCGTACGTAGAAGGCGAATGTCAAATGAGCACAAACTTATCCATGTCTTAGACATTCACGCAGCTACGCTATCCCATCACTCCATTAAGAAGGACGAACACCATAGCTTATTTTGGCAGCTATACCTCAAACTATAACCATCTTCAAATGCTAGCAAAATCACTATAAAAATACCATTTTGTTACAAGCTTTCAATCACCTACACCCTTAAGGGTGGCCTAATTTTAGCATCAATCACAAAAACCAAATAATTATCCTTAAATGCTTCGAAAAAGGTAGTGCTAGAAATAGAATCTAGCAATCCCTTTTCTTAAAATCCAGCACTC |

Or see the online file at ResearchGate (http://doi.org/10.13140/RG.2.2.21213.74727).
