# Supplementary material for: Genome-wide identification and expression analysis of aquaporin family in Canavalia rosea and their roles in the adaptation to saline-alkaline soils and drought stress
Source: BMC Plant Biol. 2021 Jul 13;21:333. doi: 10.1186/s12870-021-03034-1 (PMC8278772; doi:10.1186/s12870-021-03034-1)
Supplement: Supplementary file 8 — Additional file 8: Table S2. Primers’ information used in this study. [file 12870_2021_3034_MOESM8_ESM.docx]

**Table S2**

| **Primer ID** | **Sequence (from 5′ to 3′)** | **Purpose** |
| --- | --- | --- |
| CrPIP1;1RTF | TGTGAGAGTTGGTGCCAATAG | Primer pair for qRT-PCR of *CrPIP1;1* in *C. rosea* |
| CrPIP1;1RTR | CTGAAGGTGGCTCTTTGTAGTC |  |
| CrPIP1;2RTF | GTTCTTGGCACGCAAGTTATC | Primer pair for qRT-PCR of *CrPIP1;2* in *C. rosea* |
| CrPIP1;2RTR | CACCACCAAGCCTCTCATATT |  |
| CrPIP1;3RTF | CGGCATTAACCCAGCTAGAA | Primer pair for qRT-PCR of *CrPIP1;3* in *C. rosea* |
| CrPIP1;3RTR | AAGGGCAGCTCCAACAAA |  |
| CrPIP1;4RTF | AGGAGTCTAGGTGCTGCTATAA | Primer pair for qRT-PCR of *CrPIP1;4* in *C. rosea* |
| CrPIP1;4RTR | CGGATCACGATCTGGTGATATAAA |  |
| CrPIP1;5RTF | AGGGGTTAACCTTGTGAGCG | Primer pair for qRT-PCR of *CrPIP1;5* in *C. rosea* |
| CrPIP1;5RTR | TGGTCATCCCAGGCTTTGTC |  |
| CrPIP2;1RTF | CCGTCATAGGCCACAAGAAA | Primer pair for qRT-PCR of *CrPIP2;1* in *C. rosea* |
| CrPIP2;1RTR | GTGCAGTAGACGAGGACAAAG |  |
| CrPIP2;2RTF | CTGTGGCTGATGGCTACAATA | Primer pair for qRT-PCR of *CrPIP2;2* in *C. rosea* |
| CrPIP2;2RTR | CTTAGGATCAGTGGCAGAGAAG |  |
| CrPIP2;3RTF | AGCAACCCTTCTCTTCCTTTAC | Primer pair for qRT-PCR of *CrPIP2;3* in *C. rosea* |
| CrPIP2;3RTR | CGTCACATTCGGTGTTACCT |  |
| CrPIP2;4RTF | TGCCACACTTCTCTTCCTTTAC | Primer pair for qRT-PCR of *CrPIP2;4* in *C. rosea* |
| CrPIP2;4RTR | CAACACCACCACAAACATCAC |  |
| CrPIP2;5RTF | GCAGTGACATTTGGGTTGTTT | Primer pair for qRT-PCR of *CrPIP2;5* in *C. rosea* |
| CrPIP2;5RTR | CCAACCCAACTCCACAGATAG |  |
| CrPIP2;6RTF | GGAAAGTGTCGCTGGTTAGA | Primer pair for qRT-PCR of *CrPIP2;6* in *C. rosea* |
| CrPIP2;6RTR | CTCCTGCGTATCTGTTGTAGTAG |  |
| CrEF-αRTF | GACCTTCTTCGTTTCTCGCA | Primer pair for qRT-PCR of reference gene CrEF-α in *C. rosea* |
| CrEF-αRTR | CGAACCTCTCAATCACACGC |  |
| CrPIP1;5OXF | GGCAGCGGCCGAATTC ATGGAGGGGAAGGAACAGGA | Primer pair for cloning the full-length CDS of *CrPIP2;3* and construction of *CrPIP1;5*-pEGAD, *Eco*RI and *Bam*HI sites were underlined |
| CrPIP1;5OXR | CAGTTATCTAGGATCC TTACTTGGACTTGAAAGGAATGG |  |
| AtACT2RTF | GGTAACATTGTGCTCAGTGGTGG | Primer pair for qRT-PCR of reference gene *AtActin2* (*At3g18780*) in Arabidopsis |
| AtACT2RTF | AACGACCTTAATCTTCATGCTGC |  |
