# Supplementary material for: Genome-wide identification and expression analysis of aquaporin family in Canavalia rosea and their roles in the adaptation to saline-alkaline soils and drought stress
Source: BMC Plant Biol. 2021 Jul 13;21:333. doi: 10.1186/s12870-021-03034-1 (PMC8278772; doi:10.1186/s12870-021-03034-1)
Supplement: Supplementary file 9 — Additional file 9: Table S3. The categories of cis-acting elements identified in the CrAQPs’ promoter regions. [file 12870_2021_3034_MOESM9_ESM.docx]

**Table S3**

|  | **LRE** | **GRE** | **JARE** | **AuRE** | **SARE** | **ABRE** | **ERE** | **MYC** | **MYB** | **MBS** | **TC-rich repeats** | **LTR** |
| --- | --- | --- | --- | --- | --- | --- | --- | --- | --- | --- | --- | --- |
| CrPIP1;1 | 7 | 0 | 2 | 0 | 0 | 1 | 2 | 0 | 4 | 2 | 1 | 0 |
| CrPIP1;2 | 11 | 0 | 2 | 2 | 0 | 0 | 3 | 4 | 3 | 2 | 0 | 0 |
| CrPIP1;3 | 16 | 3 | 4 | 0 | 1 | 2 | 0 | 4 | 6 | 1 | 1 | 1 |
| CrPIP1;4 | 19 | 0 | 2 | 0 | 0 | 0 | 7 | 4 | 6 | 0 | 0 | 0 |
| CrPIP1;5 | 18 | 1 | 2 | 2 | 1 | 9 | 3 | 5 | 4 | 0 | 1 | 0 |
| CrPIP2;1 | 16 | 0 | 0 | 1 | 0 | 6 | 2 | 2 | 1 | 1 | 0 | 0 |
| CrPIP2;2 | 11 | 1 | 4 | 0 | 0 | 1 | 6 | 4 | 5 | 0 | 1 | 0 |
| CrPIP2;3 | 16 | 0 | 2 | 0 | 0 | 0 | 6 | 5 | 1 | 0 | 0 | 0 |
| CrPIP2;4 | 16 | 0 | 0 | 1 | 0 | 0 | 6 | 3 | 2 | 0 | 0 | 1 |
| CrPIP2;5 | 18 | 0 | 0 | 0 | 0 | 1 | 0 | 4 | 3 | 0 | 0 | 1 |
| CrPIP2;6 | 15 | 1 | 0 | 0 | 2 | 2 | 4 | 2 | 1 | 1 | 1 | 1 |
| CrTIP1;1 | 16 | 0 | 4 | 0 | 1 | 0 | 6 | 4 | 1 | 0 | 0 | 0 |
| CrTIP1;2 | 15 | 0 | 2 | 1 | 1 | 5 | 6 | 2 | 3 | 0 | 1 | 0 |
| CrTIP1;3 | 15 | 2 | 6 | 1 | 0 | 8 | 1 | 7 | 2 | 0 | 1 | 1 |
| CrTIP1;4 | 12 | 1 | 0 | 0 | 0 | 0 | 15 | 1 | 2 | 0 | 0 | 0 |
| CrTIP2;1 | 17 | 1 | 2 | 0 | 0 | 2 | 2 | 3 | 2 | 1 | 1 | 0 |
| CrTIP2;2 | 13 | 3 | 2 | 1 | 0 | 2 | 1 | 2 | 10 | 0 | 0 | 2 |
| CrTIP3;1 | 10 | 1 | 2 | 0 | 0 | 2 | 0 | 7 | 4 | 1 | 0 | 1 |
| CrTIP3;2 | 11 | 0 | 2 | 0 | 0 | 0 | 4 | 5 | 1 | 0 | 0 | 0 |
| CrTIP4;1 | 18 | 0 | 0 | 0 | 1 | 1 | 4 | 0 | 2 | 1 | 0 | 0 |
| CrTIP5;1 | 10 | 0 | 2 | 0 | 1 | 2 | 1 | 3 | 0 | 1 | 0 | 1 |
| CrNIP1;1 | 15 | 0 | 2 | 1 | 0 | 1 | 5 | 1 | 1 | 0 | 1 | 0 |
| CrNIP1;2 | 12 | 0 | 6 | 0 | 0 | 1 | 6 | 1 | 1 | 1 | 1 | 0 |
| CrNIP1;3 | 24 | 0 | 2 | 0 | 0 | 0 | 2 | 1 | 3 | 0 | 0 | 0 |
| CrNIP2;1 | 14 | 1 | 2 | 2 | 0 | 0 | 6 | 4 | 3 | 0 | 0 | 0 |
| CrNIP2;2 | 11 | 1 | 0 | 1 | 1 | 0 | 3 | 3 | 6 | 0 | 1 | 0 |
| CrNIP3;1 | 10 | 0 | 0 | 0 | 2 | 2 | 6 | 1 | 2 | 0 | 1 | 1 |
| CrNIP3;2 | 14 | 0 | 2 | 0 | 1 | 1 | 5 | 4 | 3 | 2 | 1 | 0 |
| CrNIP3;3 | 18 | 0 | 4 | 1 | 1 | 1 | 7 | 0 | 2 | 0 | 0 | 0 |
| CrNIP4;1 | 12 | 1 | 0 | 0 | 1 | 3 | 1 | 6 | 4 | 0 | 1 | 0 |
| CrNIP5;1 | 16 | 0 | 12 | 0 | 0 | 2 | 4 | 1 | 2 | 1 | 0 | 1 |
| CrNIP6;1 | 20 | 0 | 2 | 0 | 0 | 2 | 4 | 3 | 2 | 0 | 1 | 0 |
| CrSIP1;1 | 16 | 1 | 2 | 0 | 1 | 3 | 6 | 1 | 4 | 0 | 1 | 1 |
| CrSIP1;2 | 11 | 0 | 0 | 2 | 1 | 2 | 5 | 4 | 1 | 1 | 0 | 0 |
| CrSIP1;3 | 20 | 0 | 0 | 0 | 0 | 2 | 3 | 2 | 1 | 1 | 1 | 2 |
| CrSIP2;1 | 23 | 0 | 2 | 0 | 0 | 0 | 5 | 0 | 0 | 0 | 1 | 0 |
| CrXIP1;1 | 12 | 3 | 0 | 0 | 1 | 2 | 7 | 2 | 2 | 0 | 1 | 0 |

The *cis*-acting elements identified in the *CrAQP*s’ promoter regions was conducted using the PlantCARE (<http://bioinformatics.psb.ugent.be/webtools/plantcare/html/>). LRE: Light responsive element; GRE: Gibberellin-responsive element; JARE: MeJA-responsiveness element; AuRE: Auxin-responsive element; SARE: Salicylic acid responsiveness; ABRE: *Cis*-acting element involved in the abscisic acid responsiveness; ERE: Ethylene-responsive element; MYC: MYC recognition site; MYB: MYB recognition site; MBS: MYB recognition site involved in drought tolerance; TC-rich repeats: *Cis*-acting element involved in the stress response; LTR: Low-temperature responsive element. The sequence information and the possible biological functions were listed at the web page of PlantCARE.
