# Supplementary material for: Genome-wide identification and expression analysis of aquaporin family in Canavalia rosea and their roles in the adaptation to saline-alkaline soils and drought stress
Source: BMC Plant Biol. 2021 Jul 13;21:333. doi: 10.1186/s12870-021-03034-1 (PMC8278772; doi:10.1186/s12870-021-03034-1)
Supplement: Supplementary file 10 — Additional file 10: Table S4. The conserved motif sequences of CrAQPs identified by the MEME web server. [file 12870_2021_3034_MOESM10_ESM.docx]

**Table S4**

| **Group** | **Motif** | **E-value** | **Consensus sequence** |
| --- | --- | --- | --- |
| CrNIP | 8 | 1.1e-149 | 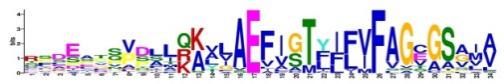  RSDEATSVDLLQKVLAEFIGTYIFVFAGCGSAIA |
|  | 2 | 9.6e-335 | 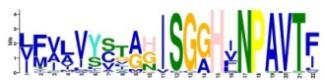  LFVLVYSTAHISGGHINPAVTF |
|  | 6 | 1.1e-276 | 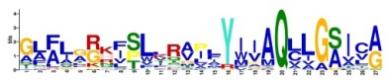  GLFLGRKFSLLRAILYIIAQLLGSICA |
|  | 7 | 5.1e-181 | 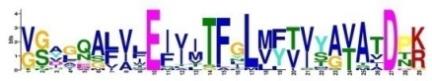  VGAGQALVLEIIITFGLMFTVYAVATDPK |
|  | 1 | 1.3e-365 | 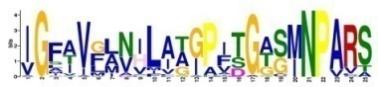  IGFAVFLNILAAGPITGASMNPARS |
|  | 4 | 1.4e-370 | 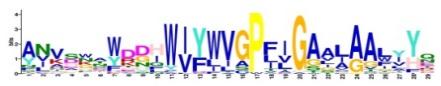  ANVSNAWDDHWIYWVGPFIGAALAALYYQ |
| CrPIP | 3 | 1.1e-312 | 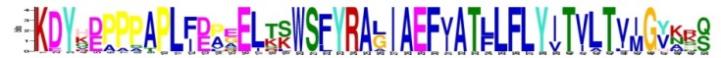  KDYHDPPPAPLFDPEELTSWSFYRAGIAEFVATLLFLYITVLTVIGYKKQ |
|  | 10 | 5.9e-061 | 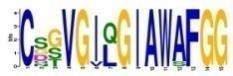  CSGVGILGIAWAFGG |
|  | 2 | 9.6e-335 | 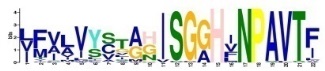  LFVLVYSTAHISGGHINPAVTF |
|  | 6 | 1.1e-276 | 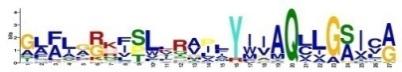  GLFLGRKFSLLRAILYIIAQLLGSICA |
|  | 9 | 2.7e-079 | 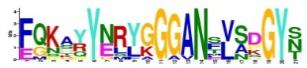  FQKAYYNRYGGGANSVSDGYS |
|  | 5 | 2.5e-284 | 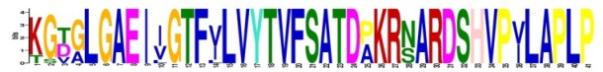  KGTGLGAEIIGTFVLVYTVFSATDPKRNARDSHVPVLAPLP |
|  | 1 | 1.3e-365 | 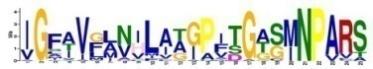  IGFAVFLNILAAGPITGASMNPARS |
|  | 4 | 1.4e-370 | 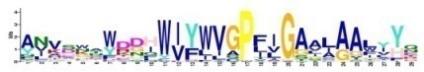  ANVSNAWDDHWIYWVGPFIGAALAALYYQ |
| CrTIP | 8 | 1.1e-149 | 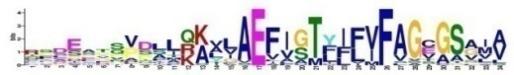  RSDEATSVDLLQKVLAEFIGTYIFVFAGCGSAIA |
|  | 2 | 9.6e-335 | 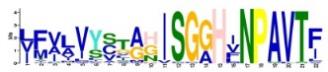  LFVLVYSTAHISGGHINPAVTF |
|  | 6 | 1.1e-276 | 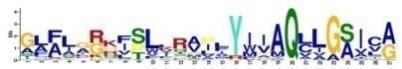  GLFLGRKFSLLRAILYIIAQLLGSICA |
|  | 7 | 5.1e-181 | 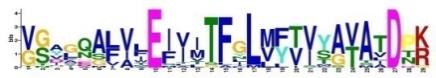  VGAGQALVLEIIITFGLMFTVYAVATDPK |
|  | 1 | 1.3e-365 | 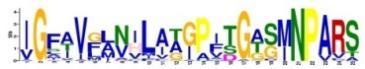  IGFAVFLNILAAGPITGASMNPARS |
|  | 4 | 1.4e-370 | 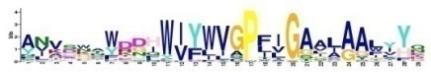  ANVSNAWDDHWIYWVGPFIGAALAALYYQ |
| CrXIP | 6 | 1.1e-276 | 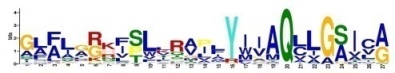  GLFLGRKFSLLRAILYIIAQLLGSICA |
| CrSIP | 6 | 1.1e-276 | 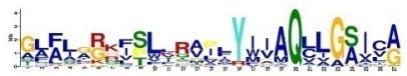  GLFLGRKFSLLRAILYIIAQLLGSICA |
|  | 4 | 1.4e-370 | 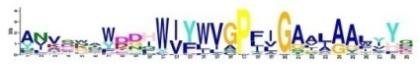  ANVSNAWDDHWIYWVGPFIGAALAALYYQ |
